# Incidence of severe acute respiratory syndrome coronavirus 2 (SARS-CoV-2) infection in North Carolina from December 2020 – February 2022

**DOI:** 10.1371/journal.pone.0332645

**Published:** 2025-10-08

**Authors:** Elyse M. Miller, Ross M. Boyce, Aaron M. Kipp, L Kristin Newby, Christopher W. Woods, Caitlin A. Cassidy, Annika K. Gunderson, C Suzanne Lea, Coralei E. Neighbors, Bonnie E. Shook-Sa, Richard Sloane, Anne P. Starling, David Wambui, Douglas Wixted, Aaron Fleischauer, Allison E. Aiello

**Affiliations:** 1 Department of Epidemiology, Gillings School of Global Public Health, University of North Carolina, Chapel Hill, North Carolina, United States of America; 2 Institute of Global Health and Infectious Diseases, University of North Carolina, Chapel Hill, North Carolina, United States of America; 3 Department of Public Health, Brody School of Medicine, East Carolina University, Greenville, North Carolina, United States of America; 4 Division of Cardiology, Duke University, Durham, North Carolina, United States of America; 5 Duke Clinical Research Institute, Duke University School of Medicine, Durham, North Carolina, United States of America; 6 Duke Clinical and Translational Science Institute, Duke University, Durham, North Carolina, United States of America; 7 Department of Medicine, Duke University Medical Center, Durham, North Carolina, United States of America; 8 Department of Pathology, Duke University Medical Center, Durham, North Carolina, United States of America; 9 Hubert-Yeargan Center for Global Health, Duke University, Durham, North Carolina, United States of America; 10 Department of Population Health Sciences, Duke University School of Medicine, Durham, North Carolina, United States of America; 11 Department of Biostatistics, Gillings School of Global Public Health, University of North Carolina, Chapel Hill, North Carolina, United States of America; 12 Center for the Study of Aging and Human Development, Duke University Medical Center, Durham, North Carolina, United States of America; 13 Career Epidemiology Field Officer, Centers for Disease Control and Prevention, Atlanta, Georgia, United States of America; 14 Department of Epidemiology, Robert N. Butler Columbia Aging Center, Mailman School of Public Health, Columbia University, New York, New York, United States of America; Retired-United States Environmental Protection Agency, UNITED STATES OF AMERICA

## Abstract

**Background:**

Surveillance estimates of SARS-CoV-2 infections over time have relied on mandatory clinician and laboratory reporting. These estimates increasingly underestimated true viral incidence due to asymptomatic infections, variable access to testing, and self-administered diagnostics. To overcome these limitations, the North Carolina Department of Health and Human Services partnered with academic researchers to conduct three concurrent population-based longitudinal cohort studies in three distinct North Carolina counties to offer more accurate estimates of the incidence, prevalence, and vaccination rates for SARS-CoV-2.

**Methods:**

We enrolled and followed adult residents of three North Carolina counties from August 2020-February 2022. Demographic and health information was collected in biweekly surveys. Nasal swabs were collected biweekly and tested for SARS-CoV-2 using PCR testing. Blood samples were collected monthly and tested for antibodies to the SARS-CoV-2 nucleocapsid and spike proteins. We calculated monthly seroprevalence, sero-incidence, PCR test positivity, and vaccination uptake.

**Results:**

We enrolled 646 participants. Routine blood samples and nasal swab samples were contributed by 639 and 642 participants, respectively. By February 2022, 98% (95% CI: 97.4–98.2) had antibodies to the SARS-CoV-2 spike protein, and 13% (95% CI: 12.4–14.2) had antibodies to the nucleocapsid protein, indicating viral exposure. PCR testing detected infection among 14% (95% CI: 13.1–15.0) of participants, but cumulative PCR test positivity was only 1.3% (95% CI: 1.2–1.4). Over half of PCR-detected infections were asymptomatic. By February 2022, 97% of participants had completed the primary vaccine series, and 52% had received a booster dose.

**Conclusions:**

Nearly all participants had anti-SARS-CoV-2 antibodies by the end of follow-up, primarily through vaccination. The incidence of PCR-detected infections was similar to antibody testing, but PCR test positivity substantially underestimated incident infections. These findings emphasize the importance of prospective infection monitoring via antibody testing in a comprehensive approach to tracking viral infections in the community setting.

## Introduction

The severe acute respiratory syndrome coronavirus 2 (SARS CoV-2) pandemic profoundly reshaped morbidity and mortality patterns in the United States (US), becoming the third leading cause of death after heart disease and cancer [1]. In North Carolina (NC) alone, approximately 3.5 million infections and 29,000 fatalities due to coronavirus disease 2019 (COVID-19) were reported between March 2020 and May 2023 when the NC Department of Health and Human Services (NC DHHS) State dashboard was discontinued [2]. Like most of the US, NC experienced substantial racial disparities in COVID-19 infection rates, with infection rates of 7.6 per 100 Black residents compared to 5.9 infections per 100 White residents [2,3]. In addition to high morbidity and mortality, rates of COVID-19 vaccination in NC lagged behind many other states, placing 23^rd^ nationally for coverage [4]. As of May 2023, only 63% of North Carolinians had received the initial full vaccine series, with vaccination rates particularly low among most minoritized groups [5].

**Fig 1:**
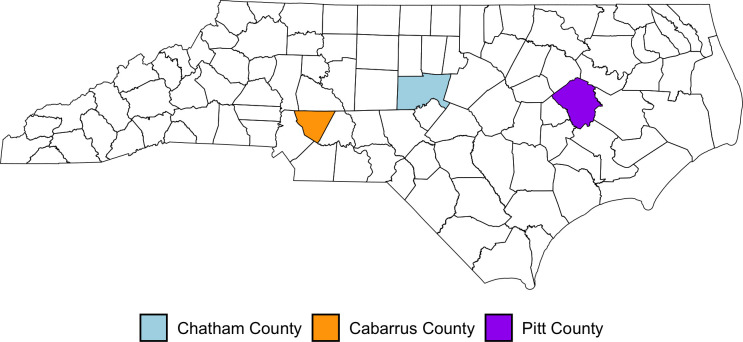
Map of North Carolina displaying county locations of study cohorts. Created by authors in the R package ‘tigris’ (Walker K (2022). tigris: Load Census TIGER/Line Shapefiles, version 2.0 [cited 2023 Sep 16]: https://CRAN.R-project.org/package=tigris) using US Census Bureau TIGER/Line Shapefiles (2022): https://www.census.gov/geo/maps-data/data/tiger-line.html.

Early in the pandemic, estimates of SARS-CoV-2 incidence and seroprevalence in NC were primarily derived from the NC Department of Health and Human Services (NC DHHS) surveillance systems along with a small number of research studies. The NC DHHS estimates were based on mandatory reporting from clinical and public health testing during clinical encounters and at testing sites and pharmacies [6]. However, depending solely on these sources likely resulted in a significant underestimation of the true disease incidence, particularly for those with mild or asymptomatic illness and those who used at-home tests [7]. Research studies conducted early in the pandemic also had limitations. Most studies were carried out among distinct populations that may not accurately reflect the wider population in the state [8–11] or utilized a cross-sectional design [3], which provides only a snapshot of the disease [12,13]. Consequently, estimates of COVID-19 cases in NC increasingly did not depict the actual transmission of disease in the state [14,15]. In response to these limitations, the NC DHHS brought together investigators at three academic centers across the state with the goal of providing refined estimates of SARS-CoV-2 seroprevalence, incidence, and vaccination uptake in NC, with a particular focus on rural areas with less access to testing [16]. To accomplish this, population-based cohort studies were launched in three counties across the state (Fig 1). Herein, we report county-level and pooled data from these studies to estimate the monthly and cumulative incidence and seroprevalence of SARS-CoV-2 infection, assessed via monthly polymerase chain reaction (PCR) testing and immunological assays, over approximately two years of follow-up. Furthermore, we evaluated vaccination status and immunity to SARS-CoV-2 in these counties over the same period.

## Materials and methods

### Study design and population

The study protocol was derived from the methods employed in the Chatham County COVID-19 Cohort Study (RMB, AEA, and BES) at the University of North Carolina at Chapel Hill [17]. This protocol was then shared with two other community-based prospective cohort studies, the Community Prevention and COVID-19 Testing (ComPACT) Study in Pitt County, NC, led by East Carolina University (ECU) and the Cabarrus County COVID-19 Prevalence and Immunity Study in Cabarrus County, NC, led by Duke University [18]. The NC DHHS selected Chatham, Pitt, and Cabarrus Counties as study sites because of their socio-demographic similarities to the state as a whole, proximity to major academic research centers, and characteristics unique to each site. For example, Chatham County’s primarily rural population facilitated the NC DHHS’s focus on rural communities with less access to testing, while Cabarrus County was the location of an ongoing study from which a study population representative of the county could be expeditiously recruited early in the pandemic [18,19]. Data collection methods across the three studies were kept largely consistent to allow for meaningful comparisons, but slight variations in sampling and recruitment methods were permitted to accommodate study-specific objectives and logistical needs, as described below.

Follow-up for the present analysis took place from December 2020 through February 2022. In the Cabarrus County study, data collection ended in December 2021; therefore, no data were included for this site for the final two months of our analysis period (January and February 2022). Eligibility criteria included age of at least 18 years, ability to provide informed consent, and continuous residency for the duration of the study in Chatham County, Cabarrus County, or Pitt County, respectively. The Cabarrus County study also required that participants have access to email. Household members of participants already enrolled in the studies were ineligible for participation.

### Sampling, recruitment and enrollment

Recruitment in UNC’s Chatham County study took place between July 29, 2020 and February 28, 2022. Participants were selected from a pre-existing community cohort supplemented with a new two-stage stratified cluster sample. Selected individuals were initially contacted by phone and postcard to determine their interest in study participation [17]. Door-to-door recruitment was later implemented to reach subgroups that were underrepresented in the study population compared to the Chatham County population and those historically underrepresented in research, including Black and Latinx individuals [20]. Individuals who expressed interest in study participation were invited to provide their contact information via an online survey. In the ECU-led Pitt County study, the first wave of recruitment occurred from July 3, 2020 to January 16, 2021. Participants were recruited via email listservs, fliers, website/Facebook pages, and local media and invited to take an anonymous public survey. Upon survey completion, respondents were asked to provide their contact information if they were interested in participating in a follow-up study. All interested respondents were approached for enrollment. This convenience sampling approach was necessary as the number of interested respondents was insufficient to perform random sampling stratified by sociodemographic characteristics. Following the first year of the study, a second wave of targeted recruitment was conducted in Pitt County to increase enrollment of key groups underrepresented in the cohort. This supplemental recruitment period began December 21, 2021 and concluded January 28, 2022. In Duke University’s Cabarrus County study, participants were selected and recruited between June 9, 2020 and September 4, 2020 from the ongoing Measurement to Understand the Reclassification of Disease of Cabarrus and Kannapolis (MURDOCK) Study cohort using weighted, random sampling across age, sex, and self-identified race and ethnicity, described in detail in Neighbors et al. and elsewhere [18,21,22].

Written informed consent was obtained from all study participants with IRB approval granted from each of the respective study institutions.

### Survey data collection

Participants in all three studies completed a longer electronic survey at baseline and shorter biweekly surveys in English or Spanish using Research Electronic Data Capture (REDCap) (S1 Appendix) [23]. In Pitt County, the baseline survey was readministered annually. Pitt County participants without internet access could opt to complete surveys electronically during clinic visits with assistance from study staff. Participants without internet access in Chatham County could opt for a paper-based version of the surveys or to complete surveys electronically with staff assistance during clinic visits. The surveys collected data on participant demographics and COVID-19 vaccination status, infection status, and symptoms and were consistent across sites other than slight modifications made to accommodate local context. Participant race was collected by self-report and coded as a categorical variable that allowed participants to select more than one option. Participant ethnicity and biological sex were also collected by self-identification but were coded dichotomously (Hispanic or Latino vs. non-Hispanic or non-Latino and male vs. female). Alternatively, participants could decline to report their race, ethnicity, and sex. Age was collected by self-report and coded as a continuous variable. Where age was missing, we calculated it using the participant’s self-reported birth year.

### Clinic visits

Participants in all three studies attended monthly in-person clinic visits to collect biological samples and anthropometric measurements. Alternatively, Chatham County participants could opt to do at-home sample collection for the duration of the study and forgo any in-person clinic visits.

### Nasal swab testing and collection

Nasal swabs (self-collected mid-turbinate nasal swabs [MTNS] in Chatham and Pitt Counties and self-collected anterior nasal swabs in Cabarrus County) were collected bimonthly at each site. For all three studies, baseline swabs were collected at an in-person clinic visit under the supervision of a study-employed healthcare worker. In Cabarrus County, all other nasal swabs were collected at home by the participant. In Chatham and Pitt Counties, one swab was self-collected at a monthly in-person clinic visit under the supervision of a study healthcare worker, and a second swab was self-collected by the participant at home and brought to the participant’s next monthly clinic visit (Chatham County) or mailed directly to the NC State Lab of Public Health (Pitt County). No in-person clinic visits were held in Pitt County from May through September 2021. During this period, both swabs were self-collected at home and mailed in except for in August and September, during which no swabs were collected due to logistical issues. All swabs collected in the Pitt and Cabarrus County studies and MTNS collected at clinic visits in the Chatham County study were sent to the North Carolina State Lab of Public Health (SLPH) for PCR testing using either the CDC Influenza SARS-CoV-2 (Flu SC2) Multiplex Assay (sensitivity: 100.0%; specificity: ≥ 99.8%) [24,25] or the Thermo Fisher TaqPath™ COVID-19 Combo Kit assay (sensitivity: 85.3–100.0%; specificity: 100.0%) [26–28]. Samples sent to the SLPH were not tested if they arrived 72 hours or more after collection, had incomplete specimen labeling or documentation, or had an insufficient amount of fluid in the submitted cryovial. Swabs collected at home in Chatham County were tested on-site at the University of North Carolina (UNC) using the TaqPath™ COVID19 Combo Kit assay.

### Blood sample collection and testing

Detailed methods of blood sample collection, processing and serological testing were previously published for the Chatham and Cabarrus County studies [29,30]. In brief, all three counties collected monthly blood samples at in-person clinic visits using routine phlebotomy. In Chatham County only, participants could opt for at-home collection with a Tasso serum self-collection device [31] in lieu of in-person sample collection. Tasso-collected samples were mailed back to the study staff at UNC for processing in a sealed biohazard bag. In Pitt County, samples were not collected from May through September due to not having in-person clinic visits.

Blood samples in the Cabarrus County study were initially collected every other month (June 2020-April 2021) then monthly thereafter (May-December 2021) to align with monthly sample collection in Chatham and Pitt Counties. After 12 months in the Chatham County study, participants transitioned to having clinic visits with sample collection every other month. If a participant tested positive for COVID-19 within the study or via external testing, in-person sample collection was delayed by 14 days in Pitt County and by 28 days in Cabarrus County. In Chatham County, COVID-19 positive participants were mailed Tasso kits for self-sample collection.

**Fig 2:**
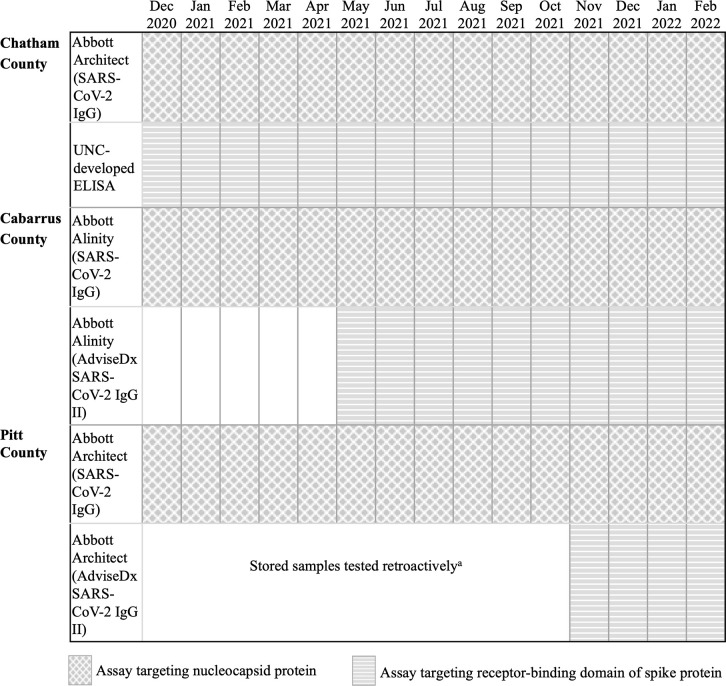
Gantt chart depicting period of use for each serological assay during follow-up. ^a^All samples taken after September 2021 were tested. All samples taken at baseline and the last study visit before vaccination from December 2020-September 2021 were tested retrospectively beginning in October 2021. If the baseline or last pre-vaccination sample was positive or the participant tested positive on a nucleocapsid assay or PCR test between the baseline and last pre-vaccination study visits, all samples taken between these visits were also tested retrospectively.

Samples were processed and tested by study staff at UNC, Duke University, and East Carolina University (ECU) for the Chatham, Cabarrus, and Pitt County studies, respectively, using assays to detect IgG to the SARS-CoV-2 nucleocapsid protein or spike protein (Fig 2). In the Chatham County study, enzyme-linked immunosorbent assays (ELISA) (sensitivity: 98.0%; specificity: 100.0%) developed and validated at UNC were used to test for total IgG to the receptor-binding domain of the SARS-CoV-2 spike protein, and the Abbott SARS-CoV-2 IgG assay (sensitivity: 87.8–100.0%; specificity: 99.6–100.0%) was used with the Abbott Architect system to test for total IgG to the nucleocapsid protein [32–36]. In the Cabarrus County study, all samples were tested for total IgG to the nucleocapsid protein using the Abbott SARS-CoV-2 IgG assay on the Abbott Alinity system. From May through December 2021, samples were also tested for IgG to spike protein using the Abbott AdviseDx SARS-CoV-2 IgG II assay (sensitivity: 95.6%; specificity: 98.7–100.0%) on the Alinity system [37,38]. The Pitt County study used the Abbott Architect system to test samples for IgG antibodies to the nucleocapsid protein with the Abbott SARS-CoV-2 IgG assay, and beginning in October 2021, for IgG antibodies to the spike protein using the Abbott AdviseDx SARS-CoV-2 IgG II assay. Once the spike protein antibody assay became available, Pitt County study staff retrospectively tested all stored serum samples from the first year of the study that were taken at baseline and at a participant’s last study visit prior to vaccination (‘pre-vaccination’). Stored samples taken between these two timepoints were only tested if the baseline or pre-vaccination sample was positive or the participant had a positive result on any nucleocapsid assay or PCR test administered between baseline and vaccination. Otherwise, the samples between baseline and pre-vaccination were not tested but were presumed to be negative for antibodies to the spike protein.

For all three studies, a positive result for IgG to the SARS-CoV-2 nucleocapsid protein at the manufacturer-recommended threshold was interpreted as evidence of natural infection among both vaccinated and unvaccinated participants. A positive result for IgG to the receptor-binding domain of the spike protein at the manufacturer-recommended threshold was interpreted as evidence of natural infection among unvaccinated participants and of natural infection or vaccination among vaccinated participants. Given the rarity of reinfection within the first 90 days after a previous infection and the complexities of differentiating between preexisting infections and reinfections during this period, we considered a positive test result to be evidence of reinfection only if the participant did not have a positive result within the 90 days preceding the test [39].

Due to the nature of the unfolding pandemic, participants were notified if they had a positive PCR test by the SLPH or study personnel. All participants were also provided with regular individual reports of their PCR and serological test results.

### Statistical analyses

Descriptive statistics were calculated for the combined cohort as well as separately for each study. We estimated monthly seroprevalence, defined as the number of participants who tested positive for antibody to spike protein, indicating seropositivity from infection or vaccination, or nucleocapsid protein, indicating seropositivity from infection, in each month divided by the number of participants with a valid test result in the same month, and cumulative seroprevalence, defined as the number of participants who tested positive for antibody to spike or nucleocapsid protein during follow-up divided by the total number of participants in the study population. We also estimated monthly sero-incidence, defined as the number of participants who tested positive for antibodies to nucleocapsid protein in each month divided by the number of participants who had a valid test result in that month, among participants who were seronegative in the previous month.

Monthly test positivity was calculated as the number of positive PCR tests in each month divided by the number of PCR tests with valid results in that month. Cumulative test positivity was calculated as the total number of positive PCR tests during follow-up divided by the total number of PCR tests with valid results during follow-up. We estimated the cumulative incidence of PCR-confirmed infections as the number of participants who had at least one positive PCR result during follow-up but had not tested positive within the 90 days preceding the positive test, divided by the total number of participants with a valid PCR test result during follow-up. Among PCR-confirmed infections, we estimated the proportion of reinfections and the proportion of asymptomatic infections. The proportion of reinfections was defined as the number of PCR-confirmed infections among participants who had been previously infected with SARS-CoV-2 but had not had a positive PCR result in the preceding 90 days divided by the total number of PCR-confirmed infections. The proportion of asymptomatic infections was defined as the number of infections confirmed by PCR in participants who did not report experiencing cough, shortness of breath, difficulty breathing, fever, chills, rigors, myalgia, headache, sore throat, or new olfactory or taste disorder in the two weeks before or after their test date divided by the total number of PCR-confirmed infections. These symptoms were selected based on their clinical consistency with SARS-CoV-2 infection during our study period [40].

Prevalence of vaccination to SARS-CoV-2 was calculated for the initial vaccine series and the booster vaccine. In the case of the initial vaccine series, “at least partial vaccination“ was defined as having reported receipt of at least one dose of either the Pfizer messenger RNA (mRNA) vaccine (BNT162b2) or Moderna mRNA vaccine (mRNA-1273), and “at least full vaccination” was defined as having received at least two doses of the Pfizer or Moderna vaccines or one dose of the Johnson and Johnson vaccine (Ad26.COV2.S). “At least boosted” was defined as having received a booster dose of either the Pfizer or Moderna vaccine. Vaccination status was assessed monthly using data self-reported by participants on the last biweekly survey in each month. For participants with no history of SARS-CoV-2 infection, self-reported vaccination status was confirmed through testing for antibodies to the spike protein.

Estimates and corresponding 95% confidence intervals were generated for all analyses using R software (version 3.6.1) [41]. For all estimates of infection frequency, we calculated 95% Clopper-Pearson confidence intervals due to small numbers of monthly infections. For estimates of vaccination and booster prevalence, we calculated 95% Wald confidence intervals.

## Results

### Study population

**Fig 3:**
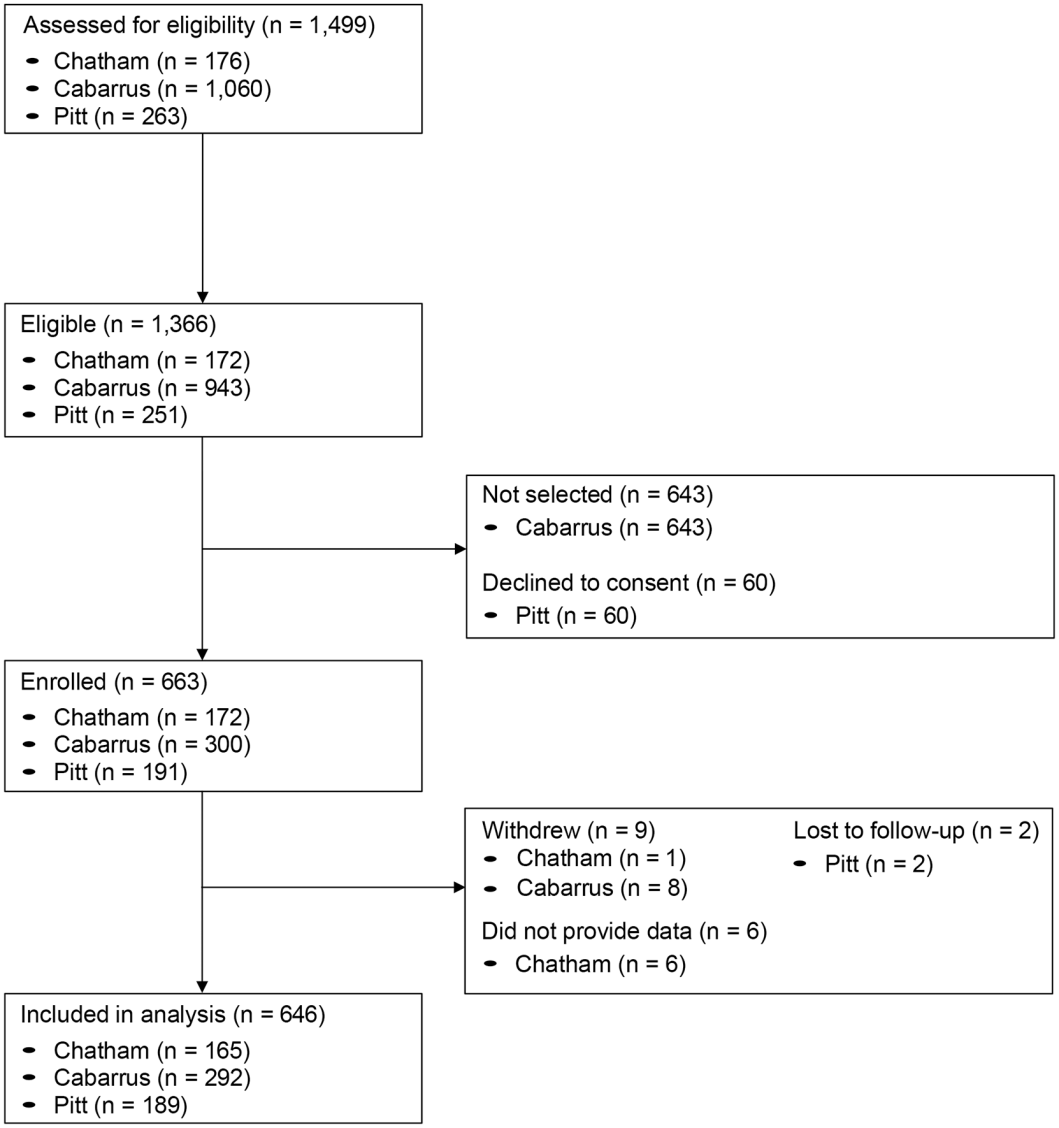
Participant flow diagram.

A total of 646 participants were included in the present analysis (165 (25.5%) from Chatham County, 189 (29.3%) from Pitt County, and 292 (45.2%) from Cabarrus County (Fig 3). Another 1,499 individuals were screened for inclusion but did not meet our eligibility criteria, including 4 from Chatham County, 12 from Pitt County, and 117 from Cabarrus County. During the analysis period, 14 participants withdrew from the study (1 from Chatham County, 2 from Pitt County, and 11 from Cabarrus County), 36 had their participation discontinued as a result of not meeting one or more study conditions (6 Chatham County participants did not provide any data to the study, 4 Pitt County participants moved out of the county, and 8 Cabarrus County and 18 Pitt County participants did not provide consent to participate in the respective extension studies), and 31 Pitt County participants were lost to follow up.

Study participants were older and more likely to be white and non-Hispanic compared to state averages (Table 1). Participant age ranged from 19 to 89 years, with a mean of 56 years (60 (range 20–88) in the Chatham cohort, 50 (range 19–84) in the Pitt cohort, and 57 (range 25–89) in the Cabarrus cohort), whereas the state average is 39 years. More than 60% of participants were female (63.7% overall, 62.8% in Chatham, 68.3% in Pitt, and 61.3% of Cabarrus), and the vast majority identified as white (82.6% overall, 86.7% in Chatham, 88.4% in Pitt, and 76.7% in Cabarrus) and non-Hispanic (91.7% overall, 88.8% in Chatham, 98.4% in Pitt, and 89.0% in Cabarrus).

**Table 1:** Demographic characteristics of participants in the Chatham County, Pitt County, and Cabarrus County study cohorts and the overall study population compared to the combined general population of the three counties and of the state as a whole.

Demographic Characteristic	Chatham County Cohort n = 165	Pitt County Cohort n = 189	Cabarrus County Cohort n = 292	Total Study Population N = 646	Chatham/Pitt/Cabarrus Combined General Population^a^ N = 472,332	North Carolina^a^ N = 10,439,388
	N (%)	N (%)	N (%)	N (%)	N (%)	N (%)
**Age**						
18-24	3 (1.8)	13 (6.9)	0 (0.0)	16 (2.5)	31,354^b^ (9.1)	701,089^b^ (8.9)
25-34	7 (4.2)	28 (14.8)	9 (3.1)	44 (6.8)	58,059 (16.8)	1,362,100 (17.3)
35-44	17 (10.3)	31 (16.4)	41 (14.0)	89 (13.8)	61,411 (17.8)	1,300,738 (16.5)
45-54	27 (16.4)	28 (14.8)	78 (26.7)	133 (20.6)	61,649 (17.9)	1,340,415 (17.1)
55-64	32 (19.4)	45 (23.8)	76 (26.0)	153 (23.7)	58,138 (16.8)	1,365,767 (17.4)
≥65	79 (47.9)	44 (23.3)	88 (30.1)	211 (32.7)	74,735 (21.6)	1,789,448 (22.8)
**Sex**						
Female	103 (62.8)	129 (68.3)	179 (61.3)	411 (63.7)	246,320 (52.1)	5,372,038 (51.5)
Male	60 (36.6)	58 (30.7)	113 (38.7)	231 (35.8)	226,012 (47.9)	5,067,350 (48.5)
Unknown^c^	1 (0.6)	2 (1.1)	0 (0.0)	3 (0.5)	0 (0.0)	0 (0.0)
Missing	1	0	0	1	0	0
**Race**						
American Indian/Alaska Native	1 (0.6)	0 (0.0)	1 (0.3)	2 (0.3)	2,315 (0.5)	130,032 (1.2)
Asian	1 (0.6)	1 (0.5)	0 (0.0)	2 (0.3)	16,689 (3.5)	343,051 (3.3)
Black or African American	10 (6.3)	17 (9.0)	50 (17.1)	77 (12.1)	110,950 (23.5)	2,140,217 (20.5)
Native Hawaiian/ Pacific Islander	1 (0.6)	0 (0.0)	0 (0.0)	1 (0.2)	280 (0.1)	8,518 (0.1)
White	137 (86.7)	167 (88.4)	224 (76.7)	528 (82.6)	280,977 (59.5)	6,488,459 (62.2)
Unknown^d^	6 (3.8)	4 (2.1)	17 (5.8)	29 (4.5)	61,121 (12.9)	1,329,111 (12.7)
Missing	7	0	0	7	0	0
**Ethnicity**						
Hispanic	6 (3.7)	3 (1.6)	31 (10.6)	40 (6.3)	50,603 (10.7)	1,118,596 (10.7)
Not Hispanic	143 (88.8)	184 (98.4)	260 (89.0)	587 (91.7)	421,729 (89.3)	9,320,792 (89.3)
Unknown^e^	12 (7.5)	0 (0.0)	1 (0.3)	13 (2.0)	0 (0.0)	0 (0.0)
Missing	4	2	0	6	0	0

a Estimates from 2020 United States Census. Percent estimates for age are among adult residents (≥18 years) only.

b Includes ages 20–24.

c Includes participants who did not identify as male or female and participants who declined to report their biological sex.

d Includes participants who did not identify as American Indian/Alaska Native, Asian, Black or African American, Native Hawaiian/Pacific Islander, or White, participants who identified as 2 or more races, and participants who declined to report their race.

e Includes participants who did not identify as Hispanic or non-Hispanic and participants who declined to report their ethnicity.

### Seroprevalence via natural infection or vaccination

**Fig 4:**
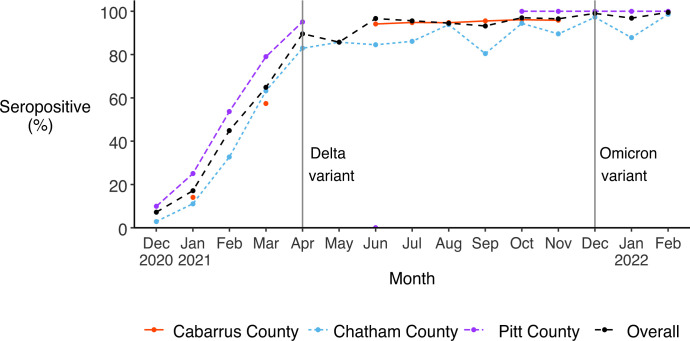
Monthly seroprevalence via vaccination or natural infection, overall and by study cohort. Vertical lines indicate when the Delta and Omicron variants of SARS-CoV-2 were first detected in North Carolina, in April and December 2021, respectively [42].

A total of 4,708 serum samples (27.5% from Chatham County participants, 28.4% from Pitt County participants, and 44.1% from Cabarrus County participants) and 5,025 serum samples (25.7% from Chatham County participants, 31.4% from Pitt County participants, and 43.0% from Cabarrus County participants) from 639 unique participants were tested for antibodies to the SARS-CoV-2 spike protein and nucleocapsid protein, respectively. Seropositivity increased substantially during our follow-up period across all three study cohorts (Fig 4). Of the three cohorts, Chatham County experienced the largest increase in the proportion of seropositive participants, from 2.9% (95% CI 1.7, 4.9) in December 2020 to 98.6% (95% CI 96.4, 99.6) in February 2022. The Pitt County cohort experienced the second largest increase, from 9.9% (95% CI 8.2, 11.9) in December 2020 to 100% (95% CI 98.8, 100.0) from October 2021 onward and was the only cohort to attain complete seroconversion. In the Cabarrus County study, which had a shorter period of sample collection, the proportion of seropositive participants increased from 14.1% (95% CI 12.5, 15.9) in January 2021 to 95.8% (95% CI 94.7, 96.7) in November 2021.

### Sero-incidence via natural infection

**Fig 5:**
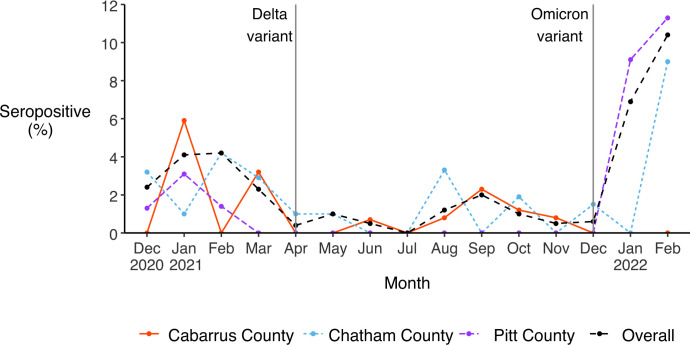
Monthly sero-incidence via natural infection, overall and by study cohort. Vertical lines indicate when the Delta and Omicron variants of SARS-CoV-2 were first detected in North Carolina, in April and December 2021 respectively [42].

Monthly sero-incidence was highest in February 2022 for the Chatham and Pitt County cohorts at 9.0% (95% CI 3.4, 18.5) and 11.3% (95% CI 6.2, 18.6), respectively) (Fig 5). The Cabarrus County cohort experienced its highest monthly sero-incidence in January 2021 at 5.9% (95% CI 4.8, 7.1). Monthly sero-incidence was lowest in months with no seroconversions, including July 2021 for Cabarrus County, June, July, September, and November 2021 and January 2022 for Chatham County, and March, April, October, November, and December 2021 for Pitt County. However, in the Pitt County study, which did not offer at-home blood sample collection, the month of seroconversion was unknown for 7 participants who tested positive for nucleocapsid protein after missing monthly clinic visits for two or more consecutive months. Across the three sites, 13.3% of participants were infected with SARS-CoV-2 during the study period. Cumulative sero-incidence was highest in Cabarrus County at 14.4%, followed by Pitt County at 14.0%, and Chatham County at 11.8%.

### PCR-confirmed SARS-CoV-2 infection by nasal swab

A total of 10,136 nasal swab samples (20.0% from the Chatham cohort, 30.5% from the Pitt cohort, and 49.5% from the Cabarrus cohort) from 642 unique participants underwent PCR testing for SARS-CoV-2. A small proportion (1.5%) of these samples produced an invalid result, including 43 in the Chatham County study and 108 in the Cabarrus County study. Of the samples with valid PCR results, 97 (1.0%) were positive for SARS-CoV-2. Among participants who contributed at least one nasal swab sample, 90 (14.0%) were infected once (11 from Chatham, 39 from Pitt, and 40 from Cabarrus) and 7 (1.1%) were infected two or more times (1 from Chatham, 3 from Pitt, and 3 from Cabarrus) during our study.

### PCR test positivity

**Fig 6:**
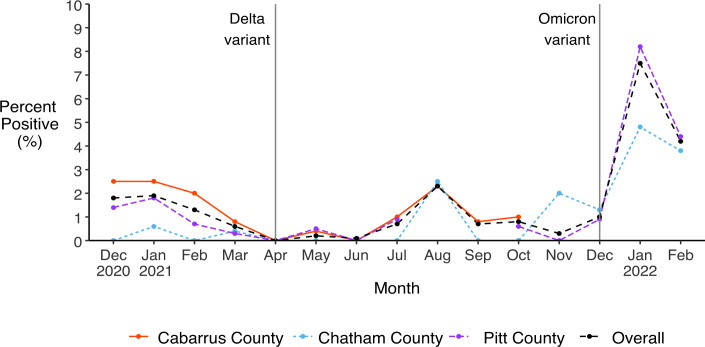
Monthly PCR test positivity. Positive PCR tests as a percentage of all PCR tests, overall and by study cohort. Vertical lines indicate when the Delta and Omicron variants of SARS-CoV-2 were first detected in North Carolina, in April and December 2021 respectively [42].

Monthly test positivity peaked in January 2022 at 4.8% (95% CI: 1.0, 13.3) in the Chatham County cohort and at 8.2% (95% CI: 5.2, 12.3) in the Pitt County cohort, then decreased slightly in February 2022 to 3.8% (95% CI: 0.8, 10.7) and 4.4% (95% CI: 2.2, 7.7), respectively (Fig 6). Among Cabarrus County participants, monthly test positivity was highest in December 2020 and January 2021 (both 2.5%, 95% CI: 2.0, 3.1). Monthly PCR test positivity was at its lowest in the months during which no SARS-CoV-2 infections were identified by PCR testing, including April and June for all three cohorts as well as December 2020 and February, May, July, September, and October 2021 for Chatham County, and November 2021 for Pitt County. Cumulative PCR test positivity ranged from 0.6% (95% CI: 0.3, 1.0) among Chatham participants to 1.4% (95% CI: 1.3, 1.5) among Cabarrus participants and 1.6% (95% CI: 1.2, 2.1) among Pitt participants over study follow-up.

### Asymptomatic PCR-confirmed SARS-CoV-2 infection

**Fig 7:**
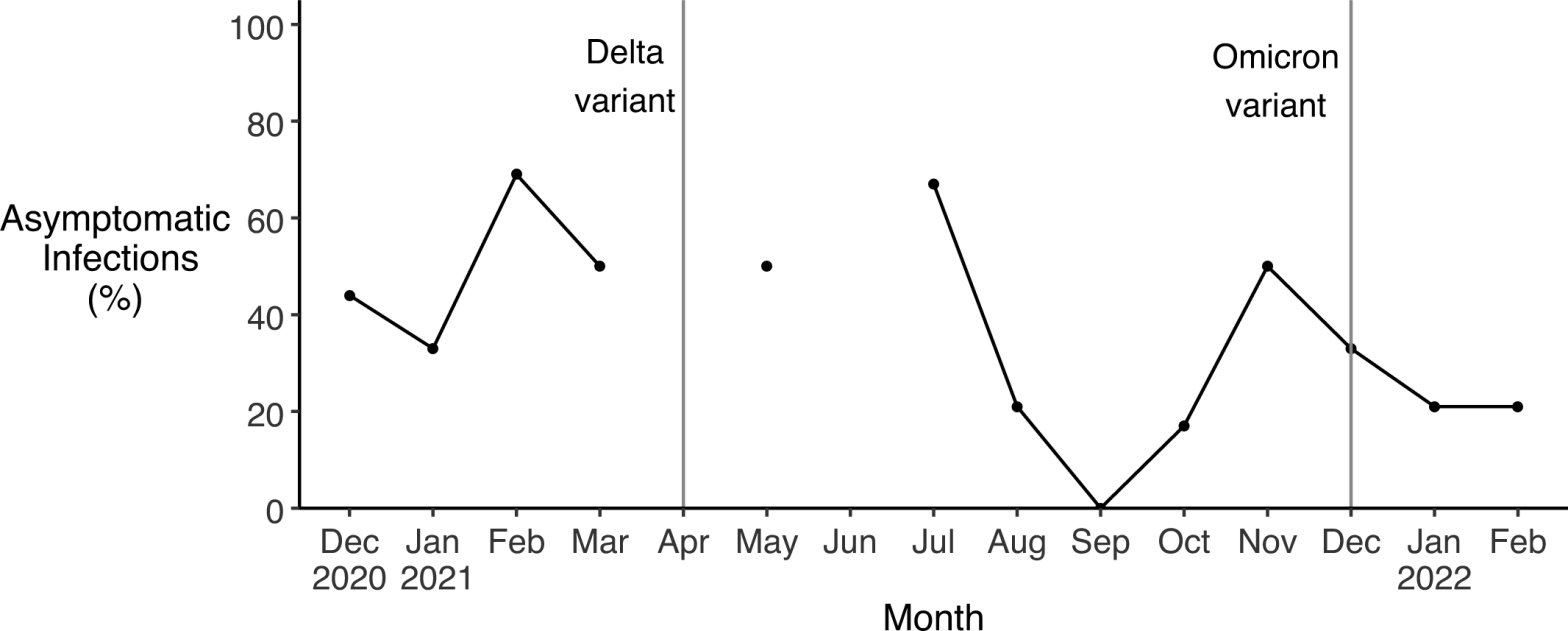
Asymptomatic SARS-CoV-2 infections. Positive PCR tests associated with asymptomatic SARS-CoV-2 infections as a percentage of all positive PCR tests across all study cohorts. No data is shown for months when there were no positive tests (April, June). Vertical lines indicate when the Delta and Omicron variants of SARS-CoV-2 were first detected in North Carolina, in April and December 2021 respectively [42].

Of the 97 SARS-CoV-2 infections identified by PCR testing, 50 (51.5% [95% CI: 48.1, 55.0]) were in participants who reported experiencing no symptoms (2 in the Chatham cohort, 7 in the Pitt cohort, and 41 in the Cabarrus cohort). Asymptomatic infections were more common among those who reported receiving at least one dose of any vaccine against SARS-CoV-2 than among known unvaccinated participants (26.6% [95% CI: 23.0, 30.4] compared with 18.8% [95% CI: 13.0, 26.2]), but this did not reach statistical significance. We found that the proportion of asymptomatic infections varied throughout the pandemic but did not follow a clear temporal trend (Fig 7).

### Prevalence of vaccination against SARS-CoV-2

**Fig 8:**
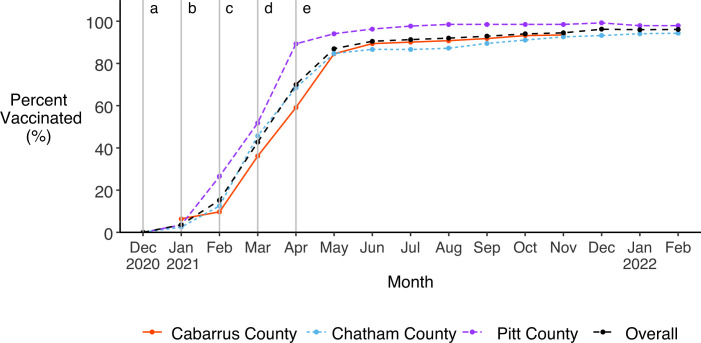
Uptake of SARS-CoV-2 primary vaccination series among all participants over study follow-up. Monthly prevalence of primary series vaccines, overall and by study cohort. Vertical lines indicate when a) frontline healthcare workers and long-term care residents and employees (Group 1) [43], b) adults ≥65 years and remaining healthcare workers (Group 2) [44], c) frontline essential workers (Group 3) [45], d) adults <65 years with comorbidities and non-frontline essential workers (Group 4) [46], and e) the general population (Group 5) were eligible for vaccination in NC [47].

**Fig 9:**
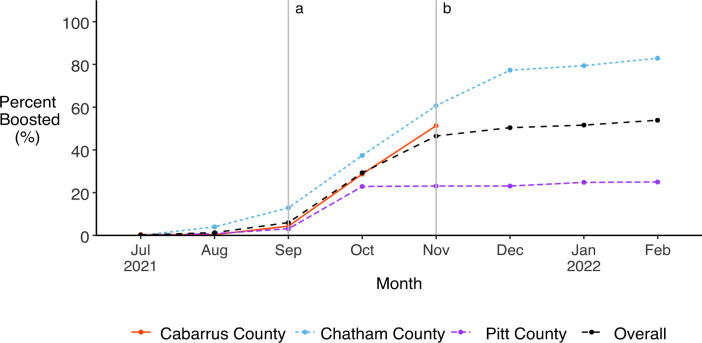
Uptake of SARS-CoV-2 booster vaccines among all participants over study follow-up. Monthly prevalence of booster vaccines overall and by study cohort, with vertical lines indicating when boosters became available to a) adults ≥65 years, adults <65 with comorbidities, and frontline workers [48] and b) all adults [49].

Vaccine uptake was very high in all three study cohorts (Fig 8). Once vaccines against SARS-CoV-2 became available in NC in December 2020, vaccine coverage of our study population rose rapidly. By June 2021, 90.5% of all participants had been fully vaccinated with the initial vaccine series. The proportion of fully vaccinated participants continued to rise over the remaining eight months but increased more gradually. By the end of our analysis period in February 2022, 97.4% of all participants had been fully vaccinated with the initial vaccine series, including 93.5% of participants in Cabarrus County (where data collection ended in December 2021), 93.2% of participants in Chatham County, and 99.3% of participants in Pitt County. The prevalence of booster doses among our full study population was much lower compared to the initial vaccine series, and the difference between cohorts was greater (Fig 9). By the end of our analysis period, 51.7% of all participants (55.5% of vaccinated participants) had received a booster dose, including 80.3% of Chatham participants (87.9% of vaccinated participants) and 51.3% of Cabarrus participants (54.9% of vaccinated participants) but only 25.0% of Pitt participants (25.5% of vaccinated participants).

## Discussion

Through this unique partnership between the state public health agency and three academic institutions, we observed sero-conversion among nearly all study participants in our three cohorts by the end of follow-up in February 2022. This suggests that most individuals living in these three populous counties in NC have some level of antibodies against SARS-CoV-2 from either natural infection or vaccination. Antibodies indicating infection with SARS-CoV-2 during the study period were detected in 13.3% of participants across the three sites. Cumulative sero-incidence was highest in Cabarrus County at 14.4%, followed by Pitt County at 14.0%, and Chatham County at 11.8%. Our sero-incidence estimates were similar to our PCR results from nasal swabs, which identified incident infection among 14.0% of participants across all three sites over our follow-up period. Our results show monthly sero-incidences as much as 10.2 percentage points greater than NC DHHS-reported cases by month over the same period [50]. In most months, our estimates based on serological analyses reflect a higher proportion of identified infections than NC DHHS estimates, which were primarily derived from PCR test results submitted through electronic laboratory reporting. There are several possible explanations for this discrepancy. Our study was able to capture sub-clinical and asymptomatic infections through routine sample collection whereas NC DHHS estimates were derived from mandatory reporting of clinical and public health testing typically initiated in response to symptomatic illness. Additionally, some of the PCR tests that informed the NC DHHS estimates may have been administered outside of the window during which PCR tests are most reliable [51], a limitation that we addressed through routine sample collection and sero-testing.

In two of the three counties (Chatham and Pitt), sero-incidence and PCR percent positivity were highest in January and February 2022, when the Omicron variant was predominant in NC [42]. Statewide estimates show a similar trend, with infections peaking in early 2022 [2]. The increased transmissibility of the Omicron variant relative to earlier SARS-Cov-2 variants has been well-documented [52]. Infections peaked earlier among Cabarrus County participants, likely because data collection ceased in November 2021 before the arrival of the Omicron variant in NC in December 2021. In contrast, monthly PCR test positivity was lowest in April and June of 2021 when no SARS-CoV-2 infections were identified. This coincides with a period in the pandemic during which the less transmissible Alpha variant was dominant and warmer weather made outdoor interactions more common in NC [42].

Asymptomatic infections accounted for more than 50% of PCR-detected infections in our study. In two large meta-analyses of 95 studies and 170 studies, prevalence estimates for asymptomatic infection among confirmed cases were 38% and 41% for cohort studies and 34% and 40% for research conducted in community settings [53,54]. In our study, asymptomatic infections were slightly more common among vaccinated participants than among unvaccinated participants. We observed no apparent trend in the proportion of asymptomatic infections over time, consistent with other studies [53,54].

Reinfections were uncommon in our study, representing only 5.3% of all infections across the three counties. Among participants infected during follow-up, the reinfection rate was 7.8%. Another study reported comparable reinfection rates ranging from 0.46% for the Alpha variant and 1.16% for the Delta variant to 13.0% for the Omicron variant [55]. These results are consistent with our findings given the timeframe of our study, which spanned the Alpha wave, the Delta wave, and the beginning of the Omicron wave in NC. However, reinfections would very likely have increased with a longer follow-up period.

We found that PCR test positivity underestimated sero-incidence via nucleocapsid testing. For example, cumulative sero-incidence across the three study sites was between 11.2 and 13.0 percentage points higher than PCR test positivity over our study period. Such underestimation may be due to misalignment between PCR testing frequency and the timing of infections [51], or to participants missing or rescheduling clinic visits, particularly if the absence or rescheduling was related to infectious symptoms not reported to study staff. Participants may also have undergone unreported testing outside of our study. Our results suggest that nucleocapsid testing is a useful and important marker of sub-clinical transmission. In future pandemics, population-based testing should be used as an adjunct to PCR testing in clinics and other settings. It would be helpful to create population-based sampling frames to quickly develop and deploy repeated monthly testing of a large population. One of the unique features of our study was the use of a Tasso device for at-home self-sample collection [29–31]. Our use of the Tasso device provided results comparable to traditional phlebotomy and allowed for efficient and convenient sample collection among participants who were unwilling or unable to visit an in-person clinic (e.g., due to concerns about risk of infection, transportation challenges, etc.). Creating a sampling frame of eligible participants and mailing them a Tasso or similar device in future pandemics will enable public health agencies to more efficiently and accurately monitor emerging diseases and identify the populations most likely to benefit from targeted interventions and resources to combat infection.

Reported completion of the primary vaccine series was high across all three study cohorts and indicated nearly complete coverage of our study population (97.4%) by February 2022. Our higher seroprevalence estimates from spike protein testing, indicating either vaccination or natural infection, and lower seroprevalence estimates from nucleocapsid testing, indicating natural infection, supported the high prevalence of vaccination self-reported in participant surveys. Completion of the primary vaccine series was substantially higher in our study than among the general adult population in NC, which had only 74% coverage by April 2023. Uptake was even lower in our target counties where only 62% (Chatham County), 56% (Cabarrus County), and 55% (Pitt County) of adults had received a complete primary vaccine series by April 2023 [5]. The high uptake of vaccines among our study population compared to our target populations is very likely due to differences in demographic and socioeconomic characteristics (Table 1), or health-seeking behavior associated with both study participation and vaccination. Booster uptake was lower than uptake of the primary vaccine series and more heterogeneous across sites. By the end of our analysis period, just over half of fully vaccinated participants had received at least one booster dose. However, booster uptake among Cabarrus County participants, and thus overall uptake, would likely have been higher if the site’s data collection had continued for the full 14 months observed in Chatham and Pitt Counties. Notably, Pitt County had both the highest prevalence of primary vaccine uptake and the lowest prevalence of booster uptake. Factors contributing to this apparent discrepancy may include the younger age composition of the Pitt County cohort. This is because adults under 65 years old in the general population (non-essential workers with no comorbidities) were not eligible to receive a first booster dose until late November 2021, shortly before the end of our study period [49].

Our study has many strengths, including its extended duration and breadth of data collection. We used three different testing methods (nucleocapsid- and spike protein-based sero-testing and PCR testing) to assess the prevalence and incidence of COVID-19 over 14 months. To our knowledge, this is one of the longest follow-up periods of any sero-study of SARS-CoV-2 published to date [56]. Because of this, we were uniquely able to capture changes over the extended course of the pandemic and through multiple viral variants. Despite our long follow-up time, we observed low attrition (10%) compared to similar studies [56]. Another key strength of our study is its inclusion of participants from three geographically and socio-demographically diverse counties, including rural communities overlooked in previous studies. This enabled more accurate and representative estimates of SARS-CoV-2 infection in NC and allowed for valid comparisons within the state. Additionally, our use of multiple testing methods minimized the possibility of missed infections to produce more robust and accurate estimates.

The results of our analysis should be considered in the context of several limitations. Although we implemented weighting techniques to minimize the potential for selection bias by certain demographic variables, many of those sampled either did not respond or declined to participate. In particular, younger individuals and those identifying as members of minoritized racial or ethnic groups, both of whom accounted for a disproportionate number of infections in NC [2], were underrepresented in our study. Moreover, our results do not account for differential study participation based on health-seeking behaviors such as vaccination, social distancing, and mask-wearing, further contributing to the risk of selection bias. A more representative study population would likely have yielded even higher estimates of SARS-CoV-2 incidence and prevalence, and lower estimates of vaccination uptake, than we observed. While there is likely residual selection bias in our estimates of overall infection incidence and prevalence and vaccination uptake, we do not expect it to have influenced our estimates of asymptomatic infection. This is particularly important, as asymptomatic infections are not well captured by traditional case-based reporting used in public health surveillance [6,7,57]. An additional consideration is that participation in the study itself may have induced greater engagement in health-seeking behavior among participants [58].

We note that our estimates are not adjusted for test performance and may underestimate actual viral incidence and prevalence in our study population as a result. Additionally, there is evidence that SARS-CoV-2 infection induces relatively low levels of anti-nucleocapsid IgG antibodies that may wane more rapidly compared to other antibodies (e.g., anti-spike and neutralizing) or may be less reliably detected by our nucleocapsid-based assays [38,59–61]. Thus, our nucleocapsid-based testing may not have captured natural infections with longer intervals between viral exposure and testing if the anti-nucleocapsid IgG levels had fallen below the assay’s positivity threshold. Given our use of multiple testing modalities, however, we would not expect this to meaningfully alter our overall findings. Missed clinic visits also may have contributed minimally to an underestimation of infections in our study. Although all participants attended at least one study visit, a minority of participants missed one or more visits. While some incident infections may have been missed as a result, these infections were likely detected in a subsequent month. The requirement for participants in the Cabarrus County cohort to have an email address may limit the generalizability of our Cabarrus County results to residents with email access. Finally, although data collection was largely identical across our sites, differences in recruitment and sampling between counties should be considered when comparing estimates. For these reasons, we were unable to combine weights across sites to provide estimates that are generalizable in aggregate nor were we able to examine seroprevalence by individual-level characteristics such as race, ethnicity, age and sex.

We have documented the progression of SARS-CoV-2 infection during the pandemic across three areas of NC that are demographically similar to the state. Throughout the study period, there were notable and rapid increases in infection rates, particularly when new viral strains emerged. Our research revealed a significant disparity between results obtained through serial nasal swab PCR testing using test positivity as a measure of viral spread versus serial serology testing for antibodies to infection, which offered a much broader scope for identifying individuals who had been infected throughout the study’s duration. By employing a dual testing approach, we were able to identify a considerably higher number of infections than those reported through routine public health surveillance. These findings emphasize not only the importance of prospective infection monitoring coupled with sero-epidemiology as crucial tools in a comprehensive approach to tracking SARS-CoV-2 infections, but the benefits of partnerships between public health agencies and academic research institutions. Ultimately, the implementation of studies like ours by state health departments will facilitate more informed decision-making and more efficient resource utilization, reducing morbidity and mortality in future pandemics.

## Supporting information

S1 Appendix Baseline and biweekly surveys. (S1 Appendix.PDF)

S1 Table SARS-CoV-2 seroprevalence by month and study site. (S1 Table.PDF)

S2 Table SARS-CoV-2 sero-incidence from nucleocapsid, by month and study site. (S2 Table.PDF)

S3 Table PCR test positivity by month and study site. (S3 Table.PDF)

S4 Table SARS-CoV-2 vaccine uptake by month and study site. (S4 Table.PDF)

S5 Table NC DHHS estimates vs. study estimates of SARS-CoV-2 incidence by month and county. (S5 Table.PDF)
