# Supplementary material for: Incidence of severe acute respiratory syndrome coronavirus 2 (SARS-CoV-2) infection in North Carolina from December 2020 – February 2022
Source: PLoS One. 2025 Oct 8;20(10):e0332645. doi: 10.1371/journal.pone.0332645 (PMC12507194; doi:10.1371/journal.pone.0332645)
Supplement: S1 Appendix — (S1 Appendix.PDF) [file pone.0332645.s001.pdf]

Chatham County COVID-19 Cohort Study

Baseline Survey

Baseline Survey

This Chatham County COVID-19 Cohort Study is being conducted by researchers from the University of North Carolina at Chapel Hill Schools of Medicine and Public Health. The purpose of this study is to learn how the new coronavirus disease, called COVID-19, is spreading and how it affects health. The results may help uncover new ways to prevent COVID-19 in the future.

Please complete the survey below.

Thank you!

What is your first name? / Cual es su nombre preferido?

____

What is your last name? / Cual es su appelido?

____

What is your date of birth? / Cual es su fecha del nacimiento?

____

What is your sex? / Cual es su sexo?

- ◯ Female / Mujer
- ◯ Male / Hombre
- ◯ Prefer not to answer / Prefiero no responder

Would you prefer to take this survey in English or Spanish? / Preferie responder esta encuesta en ingles o espanol?

- ◯ English / ingles
- ◯ Spanish / espanol

What is your race?

- ☐ American Indian or Alaska Native
- ☐ Asian
- ☐ Black or African American
- ☐ Native Hawaiian or Pacific Islander
- ☐ White
- ☐ Other
- ☐ Prefer not to answer

(Select all that apply)

What is your ethnicity?

- ◯ Hispanic or Latino
- ◯ Not Hispanic or Latino
- ◯ Other
- ◯ Prefer not to answer

What is the highest level of education or schooling you have completed?

- ◯ never attended school
- ◯ kindergarten - 8th grade
- ◯ some high school
- ◯ high school equivalency (GED)
- ◯ high school graduate
- ◯ some college
- ◯ college graduate
- ◯ graduate school or more

Do you currently smoke cigarettes, cigars, or a pipe on a daily basis?

- ◯ Yes
- ◯ No

How old were you when you first started to smoke fairly regularly?

____

What is the average number of cigarettes, cigars, and/or pipes smoked per day since you began smoking?	◯ none<br>◯ 0-10<br>◯ 11-20<br>◯ 21-30<br>◯ 31-40<br>◯ 41-50<br>◯ 51-60<br>◯ 61 or more
Did you previously smoke cigarettes, cigars, or a pipe on a daily basis?	◯ Yes<br>◯ No
How old were you when you first started to smoke fairly regularly?	__________________________
How many years has it been since you quit smoking?	__________________________
What was the average number of cigarettes, cigars, and/or pipes smoked per day when you were smoking?	◯ none<br>◯ 0-10<br>◯ 11-20<br>◯ 21-30<br>◯ 31-40<br>◯ 41-50<br>◯ 51-60<br>◯ 61 or more
Do you currently use electronic cigarettes (e-cigarettes, vaping)?	◯ Yes<br>◯ No
How old were you when you first started to use electronic cigarettes fairly regularly?	__________________________
What is the average number of e-cigarette (or other vaping product) puffs you inhale per day?	◯ 0-25<br>◯ 26-50<br>◯ 51-75<br>◯ 76-100<br>◯ 101-125<br>◯ 126-150<br>◯ 151-175<br>◯ 176-200<br>◯ 201-225<br>◯ 226-250<br>◯ 251 or more
Did you previously use electronic cigarettes (e-cigarettes, vaping)?	◯ Yes<br>◯ No
How old were you when you first started to use electronic cigarettes fairly regularly?	__________________________
How many years has it been since you quit using electronic cigarettes?	__________________________

What was the average number of e-cigarette (or other vaping product) puffs you inhale per day?	◯ 0-25<br>◯ 26-50<br>◯ 51-75<br>◯ 76-100<br>◯ 101-125<br>◯ 126-150<br>◯ 151-175<br>◯ 176-200<br>◯ 201-225<br>◯ 226-250<br>◯ 251 or more
Do you currently drink alcohol at least once a week?	◯ Yes<br>◯ No
How old were you when you first started to drink alcohol fairly regularly?	__________________________________
Think specifically about the last 30 days, including today. During the last 30 days, on how many days did you drink one or more drinks of an alcoholic beverage?	__________________________________
On the days that you drank during the past 30 days, how many drinks did you usually have each day?	__________________________________<br>(One drink is equal to 5 ounces of wine (one glass of wine), 12 ounces of beer (one can or bottle of beer), or 1.5 ounces of liquor (one shot or mixed drink))
Did you previously drink alcohol at least once a week?	◯ Yes<br>◯ No
How old were you when you first started to drink alcohol fairly regularly?	__________________________________
How many years has it been since you quit drinking alcohol?	__________________________________
During a normal 30 day period, on how many days did you drink one or more drinks of an alcoholic beverage?	__________________________________
On the days that you drank, how many drinks did you usually have each day?	__________________________________<br>(One drink is equal to 5 ounces of wine (one glass of wine), 12 ounces of beer (one can or bottle of beer), or 1.5 ounces of liquor (one shot or mixed drink))
At least once a week, do you engage in regular activity like brisk walking, jogging, bicycling, swimming, etc. long enough to work up a sweat, get your heart thumping, or get out of breath?	◯ Yes<br>◯ No

On average, how many days per week do you engage in this kind of exercise?	◯ 1<br>◯ 2<br>◯ 3<br>◯ 4<br>◯ 5<br>◯ 6<br>◯ 7
On average, how many minutes per day do you engage in this kind of exercise?	◯ 0-20<br>◯ 21-40<br>◯ 41-60<br>◯ 61 or more
When you are exercising in your usual fashion, how would you rate your average level of exertion (degree of effort)?	◯ Easy / Warm-up<br>◯ Medium (can hold a conversation) / Aerobic Development<br>◯ Hard (but you can push yourself to continue) / Aerobic Endurance<br>◯ Very Hard (cannot hold a conversation) / Anaerobic Endurance<br>◯ Extremely Hard (out of breath, your body wants to stop) / Speed, Power
Before the COVID-19 pandemic began in North Carolina, which of the following best fit your work situation?	◯ worked full time<br>◯ worked part time<br>◯ was looking for work/employment<br>◯ retired<br>◯ homemaker<br>◯ student<br>◯ on maternity/paternity leave<br>◯ on illness/sick leave<br>◯ on disability<br>◯ other
Before the COVID-19 pandemic began in North Carolina, did you consider yourself self-employed (including as an independent contractor or gig-economy worker)?	◯ yes<br>◯ no<br>◯ don't know
Of the job (or jobs) that you held before the COVID-19 pandemic in North Carolina, which description best described your main job (i.e. the job you spent the most hours at, or the job at which you had worked the longest)?	◯ managerial<br>◯ professional<br>◯ administrative support<br>◯ service<br>◯ farming/forestry/fishing<br>◯ precision production/craft/repair<br>◯ operators/fabricators/laborers<br>◯ military<br>◯ student<br>◯ not working<br>◯ other
How many years had you spent at your main job?	__________________________________
In your main job before the COVID-19 pandemic, did your employer offer you any of the following benefits?	☐ paid sick leave<br>☐ paid vacation/personal leave<br>☐ health insurance<br>☐ disability insurance<br>☐ retirement plan<br>☐ other<br>(Select all that apply.)

Has your work situation changed since the COVID-19 pandemic began in North Carolina?	◯ Yes<br>◯ No
Which of the following best fits your current work situation?	◯ works full time<br>◯ works part time<br>◯ is looking for work/employment<br>◯ retired<br>◯ homemaker<br>◯ student<br>◯ on maternity/paternity leave<br>◯ on illness/sick leave<br>◯ on disability<br>◯ other
Do you currently consider yourself self-employed (including as an independent contractor or gig-economy worker)?	◯ yes<br>◯ no<br>◯ don't know
Of the job (or jobs) that you currently hold, which description best describes your main job (i.e. the job you spend the most hours at, or the job at which you have worked the longest)?	◯ managerial<br>◯ professional<br>◯ administrative support<br>◯ service<br>◯ farming/forestry/fishing<br>◯ precision production/craft/repair<br>◯ operators/fabricators/laborers<br>◯ military<br>◯ student<br>◯ not working<br>◯ other
How many months have you spent at your current main job?	______________________
In your current main job, does your employer offer you any of the following benefits?	◯ paid sick leave<br>◯ paid vacation/personal leave<br>◯ health insurance<br>◯ disability insurance<br>◯ retirement plan<br>◯ other<br>(Select all that apply.)
On a scale of 0 (definitely not going to happen) to 10 (definitely going to happen), how likely is it that you will lose your job because of the COVID-19 pandemic?	______________________
On a scale of 0 (definitely not going to happen) to 10 (definitely going to happen), how likely is it that you will receive fewer work hours at your job because of the COVID-19 pandemic?	______________________

	always (100%)	most of the time (75%)	half of the time (50%)	less than half of the time (25%)	never (0%)

How often were you required to work from outside of the home before the COVID-19 pandemic in North Carolina?	○	○	○	○	○
Before the COVID-19 pandemic, how regularly were you in close physical contact with co-workers during your work outside of the home?	○	○	○	○	○
Before the COVID-19 pandemic, how regularly were you in close physical contact with clients during your work outside of the home?	○	○	○	○	○
Before the COVID-19 pandemic, how often did you have access to disposable gloves during your work outside of the home?	○	○	○	○	○
Before the COVID-19 pandemic, how often did you have access to a face mask during your work outside of the home?	○	○	○	○	○
Before the COVID-19 pandemic, how often did you use disposable gloves during your work outside of the home?	○	○	○	○	○
Before the COVID-19 pandemic, how often did you use a face mask during your work outside of the home?	○	○	○	○	○
Before the COVID-19 pandemic, how often did you wash your hands with soap and water during your work outside of the home?	○	○	○	○	○
Before the COVID-19 pandemic, how often did you sanitize your hands with hand sanitizer during your work outside of the home?	○	○	○	○	○
Before the COVID-19 pandemic, how worried were you that you would be exposed to COVID-19 during your work outside of the home?	○	○	○	○	○

	always (100%)	most of the time (75%)	half of the time (50%)	less than half of the time (25%)	never (0%)
How often are you required to work from outside of the home currently?	○	○	○	○	○
How regularly are you in close physical contact with co-workers during your work outside of the home currently?	○	○	○	○	○
How regularly are you in close physical contact with clients during your work outside of the home currently?	○	○	○	○	○
How often do you have access to disposable gloves during your work outside of the home currently?	○	○	○	○	○
How often do you have access to a face mask during your work outside of the home currently?	○	○	○	○	○
How often do you use disposable gloves during your work outside of the home currently?	○	○	○	○	○
How often do you use a face mask during your work outside of the home currently?	○	○	○	○	○
How often do you wash your hands with soap and water during your work outside of the home currently?	○	○	○	○	○
How often do you sanitize your hands with hand sanitizer during your work outside of the home currently?	○	○	○	○	○
How worried are you that you will be exposed to COVID-19 during your work outside of the home currently?	○	○	○	○	○

Do you currently work in any of the following high-risk settings for COVID-19 transmission?

- ☐ healthcare setting (hospital, clinic, urgent care, etc.)
- ☐ dense residential setting (nursing home, other long-term care facility)
- ☐ prison or jail
- ☐ meatpacking facility
- ☐ shipping or distribution facility
- ☐ high-volume retail facility (grocery store, etc.)

What is your height?

____
(Record your height in feet and inches (example: 5'10))

What is your weight?

____
(Record your weight in pounds (example: 145))

Are you covered by any type of medical or health insurance (including private insurance, insurance your purchased, Medicare, Medicaid, or any other health insurance program)?

- ◯ yes
- ◯ no
- ◯ don't know

What is the primary health insurance coverage that you have?

- ◯ Private health insurance through a job or school
- ◯ Insurance purchased through a state or federal health insurance exchange, such as healthcare.gov
- ◯ Insurance purchased directly through a health plan or insurance company
- ◯ Medicare
- ◯ Medi-Gap
- ◯ Medicaid
- ◯ Military health care (TRICARE, VA, CHAMP-VA, etc.)
- ◯ Indian Health Service
- ◯ Other

(Select one (your primary insurance).)

Please specify your other source of health insurance

____

Have you ever been given a diagnosis of any of the following?

	yes	no
seasonal allergies	◯	◯
asthma	◯	◯
diabetes	◯	◯
hypertension	◯	◯
cardiovascular disease (heart attack, heart failure, angina, etc.)	◯	◯
cancer	◯	◯
chronic lung or respiratory disease (COPD, emphysema, bronchitis, etc.)	◯	◯
chronic kidney disease	◯	◯
chronic liver disease (cirrhosis, etc.)	◯	◯
weakened immune system (HIV, chronic corticosteroid treatment, organ transplant, etc.)	◯	◯

other chronic condition ◯ ◯

Please identify what other chronic medical condition you have been diagnosed with. ________________________

Are you currently pregnant?
- ◯ Yes
- ◯ No

Have you previously been diagnosed with COVID-19?
- ◯ Yes
- ◯ No

When were you previously diagnosed with COVID-19?

____
(Please provide your best guess as to your date of testing, or if not tested for COVID-19, then your best guess as to the date when you were diagnosed by a clinician.)

	excellent	very good	good	fair	poor
In general, how would you have rated your health before the COVID-19 pandemic?	◯	◯	◯	◯	◯
In general, how would you rate your health over the last two weeks?	◯	◯	◯	◯	◯

Did you receive a flu vaccine this flu season (2019-2020)?
- ◯ yes
- ◯ no
- ◯ don't know

How often do you get a flu vaccine?
- ◯ every flu season
- ◯ most flu seasons
- ◯ half of the flu seasons
- ◯ less than half of the flu seasons
- ◯ never

How often have you done the following things to protect yourself from infection since the beginning of the COVID-19 pandemic in North Carolina?

	always (100%)	most of the time (75%)	half of the time (50%)	less than half of the time (25%)	never (0%)
Worn a face mask	◯	◯	◯	◯	◯
Washed hands and/or used sanitizer frequently	◯	◯	◯	◯	◯
Stayed at least 6 feet away from others	◯	◯	◯	◯	◯
Avoided large gatherings, public spaces, or crowds	◯	◯	◯	◯	◯

Avoided contact with people who could be high risk	○	○	○	○	○
Avoided food from restaurants, including takeout	○	○	○	○	○
Worked or studied at home instead of going into an office/classroom	○	○	○	○	○
Avoided shaking hands or touching people	○	○	○	○	○
Stayed home when you were sick	○	○	○	○	○
Wiped down surfaces with disinfectant	○	○	○	○	○
Cancelled or postponed planned travel for work	○	○	○	○	○
Cancelled or postponed travel for pleasure	○	○	○	○	○
Cancelled or postponed personal or social activities	○	○	○	○	○
Cancelled a doctor's appointment	○	○	○	○	○
Stockpiled food or water	○	○	○	○	○
Followed government guidelines or rules to shelter in place (staying at home, limiting contacts with other people, etc.)	○	○	○	○	○

Have you received a COVID vaccine outside of a clinical trial?

○ Yes
○ No

Where did you receive the COVID vaccine?

○ Doctors Office
○ Work/Employment
○ Retail (e.g. Walgreens, CVS)
○ Vaccine site
○ Other

Please specify where you received the COVID vaccine.

____

Please specify the city/town in NC of the vaccine site:

____

Please specify who was the hosting organization/institution of the vaccine site:

____

Which COVID vaccine did you receive?

○ Pfizer
○ Moderna
○ AstraZeneca
○ Novavax
○ Johnson & Johnson
○ Other
○ Unsure/Unknown

Please specify which other COVID vaccine you received. __________________________________

How many doses of the vaccine have you received?
○ 1
○ 2

What day did you receive the first dose of the vaccine? __________________________________

What day did you receive the second dose of the vaccine? __________________________________

Did you experience any of the following side effects after vaccination?

		No	Mild (you notice symptoms, but they aren't a problem)	Moderate (symptoms limit your normal daily activities)	Severe (symptoms make normal daily activities difficult or impossible)
1	pain at or around the injection site	○	○	○	○
2	redness at or around the injection site	○	○	○	○
3	swelling at or around the injection site	○	○	○	○
4	rash at or around the injection site	○	○	○	○
5	headache	○	○	○	○
6	fatigue	○	○	○	○
7	fever (temperature >100.4°F or >38°C)	○	○	○	○
8	chills	○	○	○	○
9	joint pain	○	○	○	○
10	muscle pain	○	○	○	○
11	nausea	○	○	○	○

How long did these side effects last?
○ Less than 12 hours
○ 12 to 24 hours
○ more than 24 hours

Did you take any medication for these side effects?
○ Yes
○ No

What medication(s) did you take for the side effects? __________________________________
(Please list all medications.)

Did you consult a physician or other health care provider for the side effects?
○ Yes
○ No

How did you experience the side effects after the second dose of the vaccination as compared to those after the first dose of the vaccination?	◯ More severe<br>◯ Less severe<br>◯ Equally severe<br>◯ Not applicable/Haven't received second dose yet
Do you plan to get a vaccine for COVID-19 if one becomes available?	◯ yes<br>◯ no<br>◯ don't know
Which of the following, if any, are reasons that you answered "no" or "don't know" about getting a COVID-19 vaccine?	☐ I am concerned about the side effects and safety of the COVID-19 vaccine<br>☐ I have an underlying condition and there is not enough research to make me feel comfortable getting a vaccine at this time<br>☐ I am concerned that the COVID-19 vaccine is being developed too fast<br>☐ I plan to wait and see if it is safe and may get it later<br>☐ I do not trust the government<br>☐ I plan to use masks and other precautions instead<br>☐ I am not a member of any group that is at high risk from COVID-19<br>☐ I do not believe COVID-19 is a serious illness<br>☐ I believe the vaccine can give me COVID-19<br>☐ I do not think the COVID-19 vaccine will work<br>☐ I do not like needles<br>☐ I already had COVID-19 and believe that I should be immune<br>☐ My doctor has not recommended a COVID-19 vaccine to me<br>☐ I did not know I needed a vaccine against COVID-19<br>☐ I am concerned about the costs associated with the vaccine (such as office visit costs or vaccine administration fees)<br>☐ I am not yet eligible (under NC phase guidelines or due to other health conditions/procedures)<br>☐ I do not have access<br>☐ Something else<br>(select all that apply)
Please specify what other reason you may or may not be receiving a COVID-19 vaccine:	__________________________

During the last two weeks, have you experienced any of the following symptoms?

	yes	no
Fever (measured by thermometer or self-diagnosed)	◯	◯
Cough (new or worsening)	◯	◯
Shortness of breath (new or worsening)	◯	◯
Fatigue (new tiredness doing normal activities)	◯	◯
Body aches	◯	◯

Headache	○	○
Diarrhea	○	○
Sore throat	○	○
Itchy, pink, or painful eyes	○	○
Runny nose or congestion	○	○
Changes in your sense of smell or taste	○	○
New rash	○	○
Repeated shaking with chills	○	○

When did the symptoms reported above first start?

____

Were you worried that you may have COVID-19 due to the symptoms you reported?

○ yes
○ no
○ don't know

Did you experience any bias or discrimination due to the symptoms you reported?

○ yes
○ no
○ don't know

Which of the following did you do to protect your friends and family after your symptoms began?

☐ wore a mask more frequently
☐ washed your hands with soap and water more frequently
☐ used hand sanitizer more frequently
☐ isolated yourself in your home more frequently
☐ stayed home more frequently
☐ wore disposable gloves more frequently

What did you do in response to the symptoms reported above?

☐ nothing
☐ took over the counter medication (ibuprofen, acetaminophen, etc.)
☐ communicated with a health care provider over the phone
☐ visited a health care provider's office
☐ visited a retail clinic or pharmacy
☐ visited urgent care (FASTMed, etc.)
☐ visited the emergency room
☐ was admitted to the hospital
☐ other
(Select all that apply.)

Please specify what other action you took in response to your symptoms.

____

If you were able to talk with a health care provider, were you told that you may have COVID-19?

○ yes
○ no
○ don't know

If you received a COVID-19 test due to the symptoms you reported, what was the result?

○ pending
○ positive
○ negative
○ inconclusive
○ did not receive a test

How many days were you admitted to the hospital?

____

Did you receive the following interventions during your hospital admission?

- ☐ extra oxygen in your nose
- ☐ treatment in the intensive care unit (ICU)
- ☐ mechanical ventilation (intubation or a breathing tube)

Have you returned to your normal health at this time?

- ◯ yes
- ◯ no
- ◯ don't know

Please provide the following information about your household.

What is your permanent address?

____

How long have you lived at this address?

- ◯ 0-3 years
- ◯ 4-6 years
- ◯ 7-10 years
- ◯ more than 10 years

How many additional people (not including yourself) live or spend a significant amount of time (greater than 40 hours a week) in this household?

- ◯ 0
- ◯ 1
- ◯ 2
- ◯ 3
- ◯ 4
- ◯ 5
- ◯ 6
- ◯ 7
- ◯ 8
- ◯ 9
- ◯ 10
- ◯ 11
- ◯ 12

How many of the people in your household are below the age of 18?

- ◯ 0
- ◯ 1
- ◯ 2
- ◯ 3
- ◯ 4
- ◯ 5
- ◯ 6
- ◯ 7
- ◯ 8
- ◯ 9
- ◯ 10
- ◯ 11
- ◯ 12

How difficult has it been for your household to adjust to changes in child care or having to home school?

- ◯ not difficult
- ◯ somewhat difficult
- ◯ very difficult
- ◯ extremely difficult

What is the primary language spoken in your household?

- ◯ English
- ◯ Spanish
- ◯ Other

What other language is the primary language of your household?	__________________________
Before the COVID-19 pandemic, did you have regular caregiving responsibilities for someone who didn't live in your household (ex. elderly parent or sibling who you regularly visited and supported, etc.)?	◯ Yes<br>◯ No
How difficult has it been to continue your caregiving responsibilities due to the COVID-19 pandemic?	◯ not difficult<br>◯ somewhat difficult<br>◯ very difficult<br>◯ extremely difficult
If someone in the household became sick with COVID-19, how well would the household be able to isolate them from other household members (let them stay in their own room and limit contact with them)?	◯ not very well<br>◯ pretty well<br>◯ very well<br>◯ extremely well
What was your approximate total household income last year from all sources, before taxes?	◯ less than $10,000<br>◯ $10,000-$19,999<br>◯ $20,000-$29,999<br>◯ $30,000-$49,999<br>◯ $50,000-$74,999<br>◯ $75,000 or more
How do you think your total household income will change this year due to the COVID-19 crisis?	◯ decrease significantly<br>◯ decrease slightly<br>◯ stay the same<br>◯ increase slightly<br>◯ increase significantly
On a scale of 0 (definitely not going to happen) to 10 (definitely going to happen), how likely do you think it is that your household will run out of money in the next 3 months?	__________________________
How often are you or your household getting help with running necessary errands, such as getting groceries or medications?	◯ always (100%)<br>◯ most of the time (75%)<br>◯ half of the time (50%)<br>◯ less than half of the time (25%)<br>◯ never (0%)
How often have you or your household been staying at home and avoiding interacting with others, aside from getting groceries?	◯ always (100%)<br>◯ most of the time (75%)<br>◯ half of the time (50%)<br>◯ less than half of the time (25%)<br>◯ never (0%)

For each additional person in your household, please provide the following information.

Person 1: What is your relationship to this person?

- ◯ partner or spouse
- ◯ child
- ◯ parent
- ◯ sibling
- ◯ other family member
- ◯ in-home childcare provider or other caregiver
- ◯ other

Person 1: Please specify your relationship with this person.

____

Person 1: What is this person's age?

____
(Please specify their age in years)

Person 1: What is this person's sex?

- ◯ Female
- ◯ Male
- ◯ Other

Person 1: What is this person's race?

- ☐ American Indian or Alaska Native
- ☐ Asian
- ☐ Black or African American
- ☐ Native Hawaiian or Pacific Islander
- ☐ White
- ☐ Other
- ☐ don't know

(Select all that apply.)

Person 1: What is this person's ethnicity?

- ◯ Hispanic or Latino
- ◯ Not Hispanic or Latino
- ◯ Other
- ◯ don't know

Person 1: What is the highest level of education or schooling this person has completed?

- ◯ never attended school
- ◯ kindergarten - 8th grade
- ◯ some high school
- ◯ high school equivalency (GED)
- ◯ high school graduate
- ◯ some college
- ◯ college graduate
- ◯ graduate school or more
- ◯ don't know

Person 1: Which of the following best fit this person's current work situation?

- ◯ works full time
- ◯ works part time
- ◯ is looking for work/employment
- ◯ retired
- ◯ homemaker
- ◯ student
- ◯ on maternity/paternity leave
- ◯ on illness/sick leave
- ◯ on disability
- ◯ other
- ◯ don't know

Person 1: Does this person currently consider themselves self-employed (including as an independent contractor or gig-economy worker)?

- ◯ yes
- ◯ no
- ◯ don't know

Person 1: Does this person currently work in any of the following high-risk settings for COVID-19 transmission?

- ☐ healthcare setting (hospital, clinic, urgent care, etc.)
- ☐ dense residential setting (nursing home, other long-term care facility)
- ☐ prison or jail
- ☐ meatpacking facility
- ☐ shipping or distribution facility
- ☐ high-volume retail facility (grocery store, etc.)
- ☐ don't know

Person 1: Does this person's employer offer them any of the following benefits at their current main job?

- ☐ paid sick leave
- ☐ paid vacation/personal leave
- ☐ health insurance
- ☐ disability insurance
- ☐ retirement plan
- ☐ other
- ☐ don't know

(Select all that apply.)

Person 1: On a scale of 0 (definitely not going to happen) to 10 (definitely going to happen), how likely is it that this person will lose their job because of the COVID-19 pandemic?

____

Person 1: On a scale of 0 (definitely not going to happen) to 10 (definitely going to happen), how likely is it that this person will receive fewer work hours at their job because of the COVID-19 pandemic?

____

	always (100%)	most of the time (75%)	half of the time (50%)	less than half of the time (25%)	never (0%)	don't know
Person 1: How often is this person required to work from outside of the home currently?	○	○	○	○	○	○
Person 1: How regularly is this person in close physical contact with co-workers during their work outside of the home currently?	○	○	○	○	○	○
Person 1: How regularly is this person in close physical contact with clients during their work outside of the home currently?	○	○	○	○	○	○

Person 1: Does this person plan to get a vaccine for COVID-19 when one becomes available?

- ○ yes
- ○ no
- ○ don't know

Person 1: Has this person had any symptoms (cough, fever, difficulty breathing, fatigue, body aches, diarrhea, runny nose, loss of smell or taste) consistent with COVID-19 in the last two weeks?

- ○ yes
- ○ no
- ○ don't know

Person 1: When did this person's symptoms begin?

____

Person 1: Is this person worried that they may have had COVID-19 because of their symptoms?	◯ yes<br>◯ no<br>◯ don't know
Person 1: Did this person experience any bias or discrimination because of their symptoms?	◯ yes<br>◯ no<br>◯ don't know
Person 1: What did this person do in response to their symptoms?	☐ nothing<br>☐ took over the counter medication (ibuprofen, acetaminophen, etc.)<br>☐ communicated with a health care provider over the phone<br>☐ visited a health care provider's office<br>☐ visited a retail clinic or pharmacy<br>☐ visited urgent care (FASTMed, etc.)<br>☐ visited the emergency room<br>☐ was admitted to the hospital<br>☐ other<br>☐ don't know<br>(Select all that apply.)
Person 1: Please specify what other action this person took in response to their symptoms.	__________________________________
Person 1: Did a health care provider tell this person that they may have COVID-19?	◯ yes<br>◯ no<br>◯ don't know
Person 1: If this person received a COVID-19 test due to their symptoms, what was the result?	◯ pending<br>◯ positive<br>◯ negative<br>◯ inconclusive<br>◯ did not receive a test<br>◯ don't know
Person 1: How many days was this person admitted to the hospital?	__________________________________
Person 1: Did this person receive any of the following interventions during their hospital admission?	☐ extra oxygen in your nose<br>☐ treatment in the intensive care unit (ICU)<br>☐ mechanical ventilation (intubation or a breathing tube)<br>☐ don't know
Person 1: Has this person returned to their normal health at this time?	◯ yes<br>◯ no<br>◯ don't know
Person 1: Which of the following did this person do to protect their friends and family after their symptoms began?	☐ wore a mask more frequently<br>☐ washed your hands with soap and water more frequently<br>☐ used hand sanitizer more frequently<br>☐ isolated yourself in your home more frequently<br>☐ stayed home more frequently<br>☐ wore disposable gloves more frequently<br>☐ don't know

For each additional person in the your household, please provide the following information.

Person 2: What is your relationship to this person?	◯ partner or spouse<br>◯ child<br>◯ parent<br>◯ sibling<br>◯ other family member<br>◯ in-home childcare provider or other caregiver<br>◯ other
Person 2: Please specify your relationship with this person.	__________________________
Person 2: What is this person's age?	__________________________<br>(Please specify their age in years)
Person 2: What is this person's sex?	◯ Female<br>◯ Male<br>◯ Other
Person 2: What is this person's race?	☐ American Indian or Alaska Native<br>☐ Asian<br>☐ Black or African American<br>☐ Native Hawaiian or Pacific Islander<br>☐ White<br>☐ Other<br>☐ don't know<br>(Select all that apply.)
Person 2: What is this person's ethnicity?	◯ Hispanic or Latino<br>◯ Not Hispanic or Latino<br>◯ Other<br>◯ don't know
Person 2: What is the highest level of education or schooling this person has completed?	◯ never attended school<br>◯ kindergarten - 8th grade<br>◯ some high school<br>◯ high school equivalency (GED)<br>◯ high school graduate<br>◯ some college<br>◯ college graduate<br>◯ graduate school or more<br>◯ don't know
Person 2: Which of the following best fit this person's current work situation?	◯ works full time<br>◯ works part time<br>◯ is looking for work/employment<br>◯ retired<br>◯ homemaker<br>◯ student<br>◯ on maternity/paternity leave<br>◯ on illness/sick leave<br>◯ on disability<br>◯ other<br>◯ don't know
Person 2: Does this person currently consider themselves self-employed (including as an independent contractor or gig-economy worker)?	◯ yes<br>◯ no<br>◯ don't know

Person 2: Does this person currently work in any of the following high-risk settings for COVID-19 transmission?	☐ healthcare setting (hospital, clinic, urgent care, etc.)<br>☐ dense residential setting (nursing home, other long-term care facility)<br>☐ prison or jail<br>☐ meatpacking facility<br>☐ shipping or distribution facility<br>☐ high-volume retail facility (grocery store, etc.)<br>☐ don't know
Person 2: Does this person's employer offer them any of the following benefits at their current main job?	☐ paid sick leave<br>☐ paid vacation/personal leave<br>☐ health insurance<br>☐ disability insurance<br>☐ retirement plan<br>☐ other<br>☐ don't know<br>(Select all that apply.)
Person 2: On a scale of 0 (definitely not going to happen) to 10 (definitely going to happen), how likely is it that this person will lose their job because of the COVID-19 pandemic?	____________________________
Person 2: On a scale of 0 (definitely not going to happen) to 10 (definitely going to happen), how likely is it that this person will receive fewer work hours at their job because of the COVID-19 pandemic?	____________________________

	always (100%)	most of the time (75%)	half of the time (50%)	less than half of the time (25%)	never (0%)	don't know
Person 2: How often is this person required to work from outside of the home currently?	◯	◯	◯	◯	◯	◯
Person 2: How regularly is this person in close physical contact with co-workers during their work outside of the home currently?	◯	◯	◯	◯	◯	◯
Person 2: How regularly is this person in close physical contact with clients during their work outside of the home currently?	◯	◯	◯	◯	◯	◯

Person 2: Does this person plan to get a vaccine for COVID-19 when one becomes available?	◯ yes<br>◯ no<br>◯ don't know
Person 2: Has this person had any symptoms (cough, fever, difficulty breathing, fatigue, body aches, diarrhea, runny nose, loss of smell or taste) consistent with COVID-19 in the last two weeks?	◯ yes<br>◯ no<br>◯ don't know
Person 2: When did this person's symptoms begin?	____________________________

Person 2: Is this person worried that they may have had COVID-19 because of their symptoms?	◯ yes<br>◯ no<br>◯ don't know
Person 2: Did this person experience any bias or discrimination because of their symptoms?	◯ yes<br>◯ no<br>◯ don't know
Person 2: What did this person do in response to their symptoms?	☐ nothing<br>☐ took over the counter medication (ibuprofen, acetaminophen, etc.)<br>☐ communicated with a health care provider over the phone<br>☐ visited a health care provider's office<br>☐ visited a retail clinic or pharmacy<br>☐ visited urgent care (FASTMed, etc.)<br>☐ visited the emergency room<br>☐ was admitted to the hospital<br>☐ other<br>☐ don't know<br>(Select all that apply.)
Person 2: Please specify what other action this person took in response to their symptoms.	________________________________
Person 2: Did a health care provider tell this person that they may have COVID-19?	◯ yes<br>◯ no<br>◯ don't know
Person 2: If this person received a COVID-19 test due to their symptoms, what was the result?	◯ pending<br>◯ positive<br>◯ negative<br>◯ inconclusive<br>◯ did not receive a test<br>◯ don't know
Person 2: How many days was this person admitted to the hospital?	________________________________
Person 2: Did this person receive any of the following interventions during their hospital admission?	☐ extra oxygen in your nose<br>☐ treatment in the intensive care unit (ICU)<br>☐ mechanical ventilation (intubation or a breathing tube)<br>☐ don't know
Person 2: Has this person returned to their normal health at this time?	◯ yes<br>◯ no<br>◯ don't know
Person 2: Which of the following did this person do to protect their friends and family after their symptoms began?	☐ wore a mask more frequently<br>☐ washed your hands with soap and water more frequently<br>☐ used hand sanitizer more frequently<br>☐ isolated yourself in your home more frequently<br>☐ stayed home more frequently<br>☐ wore disposable gloves more frequently<br>☐ don't know

For each additional person in the your household, please provide the following information.

Person 3: What is your relationship to this person?	◯ partner or spouse<br>◯ child<br>◯ parent<br>◯ sibling<br>◯ other family member<br>◯ in-home childcare provider or other caregiver<br>◯ other
Person 3: Please specify your relationship with this person.	__________________________
Person 3: What is this person's age?	__________________________<br>(Please specify their age in years)
Person 3: What is this person's sex?	◯ Female<br>◯ Male<br>◯ Other
Person 3: What is this person's race?	☐ American Indian or Alaska Native<br>☐ Asian<br>☐ Black or African American<br>☐ Native Hawaiian or Pacific Islander<br>☐ White<br>☐ Other<br>☐ don't know<br>(Select all that apply.)
Person 3: What is this person's ethnicity?	◯ Hispanic or Latino<br>◯ Not Hispanic or Latino<br>◯ Other<br>◯ don't know
Person 3: What is the highest level of education or schooling this person has completed?	◯ never attended school<br>◯ kindergarten - 8th grade<br>◯ some high school<br>◯ high school equivalency (GED)<br>◯ high school graduate<br>◯ some college<br>◯ college graduate<br>◯ graduate school or more<br>◯ don't know
Person 3: Which of the following best fit this person's current work situation?	◯ works full time<br>◯ works part time<br>◯ is looking for work/employment<br>◯ retired<br>◯ homemaker<br>◯ student<br>◯ on maternity/paternity leave<br>◯ on illness/sick leave<br>◯ on disability<br>◯ other<br>◯ don't know
Person 3: Does this person currently consider themselves self-employed (including as an independent contractor or gig-economy worker)?	◯ yes<br>◯ no<br>◯ don't know

Person 3: Does this person currently work in any of the following high-risk settings for COVID-19 transmission?	☐ healthcare setting (hospital, clinic, urgent care, etc.)<br>☐ dense residential setting (nursing home, other long-term care facility)<br>☐ prison or jail<br>☐ meatpacking facility<br>☐ shipping or distribution facility<br>☐ high-volume retail facility (grocery store, etc.)<br>☐ don't know
Person 3: Does this person's employer offer them any of the following benefits at their current main job?	☐ paid sick leave<br>☐ paid vacation/personal leave<br>☐ health insurance<br>☐ disability insurance<br>☐ retirement plan<br>☐ other<br>☐ don't know<br>(Select all that apply.)
Person 3: On a scale of 0 (definitely not going to happen) to 10 (definitely going to happen), how likely is it that this person will lose their job because of the COVID-19 pandemic?	___________________________________
Person 3: On a scale of 0 (definitely not going to happen) to 10 (definitely going to happen), how likely is it that this person will receive fewer work hours at their job because of the COVID-19 pandemic?	___________________________________

	always (100%)	most of the time (75%)	half of the time (50%)	less than half of the time (25%)	never (0%)	don't know
Person 3: How often is this person required to work from outside of the home currently?	○	○	○	○	○	○
Person 3: How regularly is this person in close physical contact with co-workers during their work outside of the home currently?	○	○	○	○	○	○
Person 3: How regularly is this person in close physical contact with clients during their work outside of the home currently?	○	○	○	○	○	○

Person 3: Does this person plan to get a vaccine for COVID-19 when one becomes available?	○ yes<br>○ no<br>○ don't know
Person 3: Has this person had any symptoms (cough, fever, difficulty breathing, fatigue, body aches, diarrhea, runny nose, loss of smell or taste) consistent with COVID-19 in the last two weeks?	○ yes<br>○ no<br>○ don't know
Person 3: When did this person's symptoms begin?	___________________________________

Person 3: Is this person worried that they may have had COVID-19 because of their symptoms?	◯ yes<br>◯ no<br>◯ don't know
Person 3: Did this person experience any bias or discrimination because of their symptoms?	◯ yes<br>◯ no<br>◯ don't know
Person 3: What did this person do in response to their symptoms?	☐ nothing<br>☐ took over the counter medication (ibuprofen, acetaminophen, etc.)<br>☐ communicated with a health care provider over the phone<br>☐ visited a health care provider's office<br>☐ visited a retail clinic or pharmacy<br>☐ visited urgent care (FASTMed, etc.)<br>☐ visited the emergency room<br>☐ was admitted to the hospital<br>☐ other<br>☐ don't know<br>(Select all that apply.)
Person 3: Please specify what other action this person took in response to their symptoms.	__________________________________
Person 3: Did a health care provider tell this person that they may have COVID-19?	◯ yes<br>◯ no<br>◯ don't know
Person 3: If this person received a COVID-19 test due to their symptoms, what was the result?	◯ pending<br>◯ positive<br>◯ negative<br>◯ inconclusive<br>◯ did not receive a test<br>◯ don't know
Person 3: How many days was this person admitted to the hospital?	__________________________________
Person 3: Did this person receive any of the following interventions during their hospital admission?	☐ extra oxygen in your nose<br>☐ treatment in the intensive care unit (ICU)<br>☐ mechanical ventilation (intubation or a breathing tube)<br>☐ don't know
Person 3: Has this person returned to their normal health at this time?	◯ yes<br>◯ no<br>◯ don't know
Person 3: Which of the following did this person do to protect their friends and family after their symptoms began?	☐ wore a mask more frequently<br>☐ washed your hands with soap and water more frequently<br>☐ used hand sanitizer more frequently<br>☐ isolated yourself in your home more frequently<br>☐ stayed home more frequently<br>☐ wore disposable gloves more frequently<br>☐ don't know

For each additional person in the your household, please provide the following information.

Person 4: What is your relationship to this person?	◯ partner or spouse<br>◯ child<br>◯ parent<br>◯ sibling<br>◯ other family member<br>◯ in-home childcare provider or other caregiver<br>◯ other
Person 4: Please specify your relationship with this person.	__________________________
Person 4: What is this person's age?	__________________________<br>(Please specify their age in years)
Person 4: What is this person's sex?	◯ Female<br>◯ Male<br>◯ Other
Person 4: What is this person's race?	☐ American Indian or Alaska Native<br>☐ Asian<br>☐ Black or African American<br>☐ Native Hawaiian or Pacific Islander<br>☐ White<br>☐ Other<br>☐ don't know<br>(Select all that apply.)
Person 4: What is this person's ethnicity?	◯ Hispanic or Latino<br>◯ Not Hispanic or Latino<br>◯ Other<br>◯ don't know
Person 4: What is the highest level of education or schooling this person has completed?	◯ never attended school<br>◯ kindergarten - 8th grade<br>◯ some high school<br>◯ high school equivalency (GED)<br>◯ high school graduate<br>◯ some college<br>◯ college graduate<br>◯ graduate school or more<br>◯ don't know
Person 4: Which of the following best fit this person's current work situation?	◯ works full time<br>◯ works part time<br>◯ is looking for work/employment<br>◯ retired<br>◯ homemaker<br>◯ student<br>◯ on maternity/paternity leave<br>◯ on illness/sick leave<br>◯ on disability<br>◯ other<br>◯ don't know
Person 4: Does this person currently consider themselves self-employed (including as an independent contractor or gig-economy worker)?	◯ yes<br>◯ no<br>◯ don't know

Person 4: Does this person currently work in any of the following high-risk settings for COVID-19 transmission?	☐ healthcare setting (hospital, clinic, urgent care, etc.)<br>☐ dense residential setting (nursing home, other long-term care facility)<br>☐ prison or jail<br>☐ meatpacking facility<br>☐ shipping or distribution facility<br>☐ high-volume retail facility (grocery store, etc.)<br>☐ don't know
Person 4: Does this person's employer offer them any of the following benefits at their current main job?	☐ paid sick leave<br>☐ paid vacation/personal leave<br>☐ health insurance<br>☐ disability insurance<br>☐ retirement plan<br>☐ other<br>☐ don't know<br>(Select all that apply.)
Person 4: On a scale of 0 (definitely not going to happen) to 10 (definitely going to happen), how likely is it that this person will lose their job because of the COVID-19 pandemic?	______________________________
Person 4: On a scale of 0 (definitely not going to happen) to 10 (definitely going to happen), how likely is it that this person will receive fewer work hours at their job because of the COVID-19 pandemic?	______________________________

	always (100%)	most of the time (75%)	half of the time (50%)	less than half of the time (25%)	never (0%)	don't know
Person 4: How often is this person required to work from outside of the home currently?	◯	◯	◯	◯	◯	◯
Person 4: How regularly is this person in close physical contact with co-workers during their work outside of the home currently?	◯	◯	◯	◯	◯	◯
Person 4: How regularly is this person in close physical contact with clients during their work outside of the home currently?	◯	◯	◯	◯	◯	◯

Person 4: Does this person plan to get a vaccine for COVID-19 when one becomes available?	◯ yes<br>◯ no<br>◯ don't know
Person 4: Has this person had any symptoms (cough, fever, difficulty breathing, fatigue, body aches, diarrhea, runny nose, loss of smell or taste) consistent with COVID-19 in the last two weeks?	◯ yes<br>◯ no<br>◯ don't know
Person 4: When did this person's symptoms begin?	______________________________

Person 4: Is this person worried that they may have had COVID-19 because of their symptoms?	◯ yes<br>◯ no<br>◯ don't know
Person 4: Did this person experience any bias or discrimination because of their symptoms?	◯ yes<br>◯ no<br>◯ don't know
Person 4: What did this person do in response to their symptoms?	☐ nothing<br>☐ took over the counter medication (ibuprofen, acetaminophen, etc.)<br>☐ communicated with a health care provider over the phone<br>☐ visited a health care provider's office<br>☐ visited a retail clinic or pharmacy<br>☐ visited urgent care (FASTMed, etc.)<br>☐ visited the emergency room<br>☐ was admitted to the hospital<br>☐ other<br>☐ don't know<br>(Select all that apply.)
Person 4: Please specify what other action this person took in response to their symptoms.	__________________________________
Person 4: Did a health care provider tell this person that they may have COVID-19?	◯ yes<br>◯ no<br>◯ don't know
Person 4: If this person received a COVID-19 test due to their symptoms, what was the result?	◯ pending<br>◯ positive<br>◯ negative<br>◯ inconclusive<br>◯ did not receive a test<br>◯ don't know
Person 4: How many days was this person admitted to the hospital?	__________________________________
Person 4: Did this person receive any of the following interventions during their hospital admission?	☐ extra oxygen in your nose<br>☐ treatment in the intensive care unit (ICU)<br>☐ mechanical ventilation (intubation or a breathing tube)<br>☐ don't know
Person 4: Has this person returned to their normal health at this time?	◯ yes<br>◯ no<br>◯ don't know
Person 4: Which of the following did this person do to protect their friends and family after their symptoms began?	☐ wore a mask more frequently<br>☐ washed your hands with soap and water more frequently<br>☐ used hand sanitizer more frequently<br>☐ isolated yourself in your home more frequently<br>☐ stayed home more frequently<br>☐ wore disposable gloves more frequently<br>☐ don't know

For each additional person in the your household, please provide the following information.

Person 5: What is your relationship to this person?

- ◯ partner or spouse
- ◯ child
- ◯ parent
- ◯ sibling
- ◯ other family member
- ◯ in-home childcare provider or other caregiver
- ◯ other

Person 5: Please specify your relationship with this person.

____

Person 5: What is this person's age?

____
(Please specify their age in years)

Person 5: What is this person's sex?

- ◯ Female
- ◯ Male
- ◯ Other

Person 5: What is this person's race?

- ☐ American Indian or Alaska Native
- ☐ Asian
- ☐ Black or African American
- ☐ Native Hawaiian or Pacific Islander
- ☐ White
- ☐ Other
- ☐ don't know

(Select all that apply.)

Person 5: What is this person's ethnicity?

- ◯ Hispanic or Latino
- ◯ Not Hispanic or Latino
- ◯ Other
- ◯ don't know

Person 5: What is the highest level of education or schooling this person has completed?

- ◯ never attended school
- ◯ kindergarten - 8th grade
- ◯ some high school
- ◯ high school equivalency (GED)
- ◯ high school graduate
- ◯ some college
- ◯ college graduate
- ◯ graduate school or more
- ◯ don't know

Person 5: Which of the following best fit this person's current work situation?

- ◯ works full time
- ◯ works part time
- ◯ is looking for work/employment
- ◯ retired
- ◯ homemaker
- ◯ student
- ◯ on maternity/paternity leave
- ◯ on illness/sick leave
- ◯ on disability
- ◯ other
- ◯ don't know

Person 5: Does this person currently consider themselves self-employed (including as an independent contractor or gig-economy worker)?

- ◯ yes
- ◯ no
- ◯ don't know

Person 5: Does this person currently work in any of the following high-risk settings for COVID-19 transmission?	☐ healthcare setting (hospital, clinic, urgent care, etc.)<br>☐ dense residential setting (nursing home, other long-term care facility)<br>☐ prison or jail<br>☐ meatpacking facility<br>☐ shipping or distribution facility<br>☐ high-volume retail facility (grocery store, etc.)<br>☐ don't know
Person 5: Does this person's employer offer them any of the following benefits at their current main job?	☐ paid sick leave<br>☐ paid vacation/personal leave<br>☐ health insurance<br>☐ disability insurance<br>☐ retirement plan<br>☐ other<br>☐ don't know<br>(Select all that apply.)
Person 5: On a scale of 0 (definitely not going to happen) to 10 (definitely going to happen), how likely is it that this person will lose their job because of the COVID-19 pandemic?	________________________
Person 5: On a scale of 0 (definitely not going to happen) to 10 (definitely going to happen), how likely is it that this person will receive fewer work hours at their job because of the COVID-19 pandemic?	________________________

	always (100%)	most of the time (75%)	half of the time (50%)	less than half of the time (25%)	never (0%)	don't know
Person 5: How often is this person required to work from outside of the home currently?	◯	◯	◯	◯	◯	◯
Person 5: How regularly is this person in close physical contact with co-workers during their work outside of the home currently?	◯	◯	◯	◯	◯	◯
Person 5: How regularly is this person in close physical contact with clients during their work outside of the home currently?	◯	◯	◯	◯	◯	◯

Person 5: Does this person plan to get a vaccine for COVID-19 when one becomes available?	◯ yes<br>◯ no<br>◯ don't know
Person 5: Has this person had any symptoms (cough, fever, difficulty breathing, fatigue, body aches, diarrhea, runny nose, loss of smell or taste) consistent with COVID-19 in the last two weeks?	◯ yes<br>◯ no<br>◯ don't know
Person 5: When did this person's symptoms begin?	________________________

Person 5: Is this person worried that they may have had COVID-19 because of their symptoms?	◯ yes<br>◯ no<br>◯ don't know
Person 5: Did this person experience any bias or discrimination because of their symptoms?	◯ yes<br>◯ no<br>◯ don't know
Person 5: What did this person do in response to their symptoms?	☐ nothing<br>☐ took over the counter medication (ibuprofen, acetaminophen, etc.)<br>☐ communicated with a health care provider over the phone<br>☐ visited a health care provider's office<br>☐ visited a retail clinic or pharmacy<br>☐ visited urgent care (FASTMed, etc.)<br>☐ visited the emergency room<br>☐ was admitted to the hospital<br>☐ other<br>☐ don't know<br>(Select all that apply.)
Person 5: Please specify what other action this person took in response to their symptoms.	__________________________________
Person 5: Did a health care provider tell this person that they may have COVID-19?	◯ yes<br>◯ no<br>◯ don't know
Person 5: If this person received a COVID-19 test due to their symptoms, what was the result?	◯ pending<br>◯ positive<br>◯ negative<br>◯ inconclusive<br>◯ did not receive a test<br>◯ don't know
Person 5: How many days was this person admitted to the hospital?	__________________________________
Person 5: Did this person receive any of the following interventions during their hospital admission?	☐ extra oxygen in your nose<br>☐ treatment in the intensive care unit (ICU)<br>☐ mechanical ventilation (intubation or a breathing tube)<br>☐ don't know
Person 5: Has this person returned to their normal health at this time?	◯ yes<br>◯ no<br>◯ don't know
Person 5: Which of the following did this person do to protect their friends and family after their symptoms began?	☐ wore a mask more frequently<br>☐ washed your hands with soap and water more frequently<br>☐ used hand sanitizer more frequently<br>☐ isolated yourself in your home more frequently<br>☐ stayed home more frequently<br>☐ wore disposable gloves more frequently<br>☐ don't know

For each additional person in the your household, please provide the following information.

Person 6: What is your relationship to this person?

- ◯ partner or spouse
- ◯ child
- ◯ parent
- ◯ sibling
- ◯ other family member
- ◯ in-home childcare provider or other caregiver
- ◯ other

Person 6: Please specify your relationship with this person.

____

Person 6: What is this person's age?

____
(Please specify their age in years)

Person 6: What is this person's sex?

- ◯ Female
- ◯ Male
- ◯ Other

Person 6: What is this person's race?

- ☐ American Indian or Alaska Native
- ☐ Asian
- ☐ Black or African American
- ☐ Native Hawaiian or Pacific Islander
- ☐ White
- ☐ Other
- ☐ don't know

(Select all that apply.)

Person 6: What is this person's ethnicity?

- ◯ Hispanic or Latino
- ◯ Not Hispanic or Latino
- ◯ Other
- ◯ don't know

Person 6: What is the highest level of education or schooling this person has completed?

- ◯ never attended school
- ◯ kindergarten - 8th grade
- ◯ some high school
- ◯ high school equivalency (GED)
- ◯ high school graduate
- ◯ some college
- ◯ college graduate
- ◯ graduate school or more
- ◯ don't know

Person 6: Which of the following best fit this person's current work situation?

- ◯ works full time
- ◯ works part time
- ◯ is looking for work/employment
- ◯ retired
- ◯ homemaker
- ◯ student
- ◯ on maternity/paternity leave
- ◯ on illness/sick leave
- ◯ on disability
- ◯ other
- ◯ don't know

Person 6: Does this person currently consider themselves self-employed (including as an independent contractor or gig-economy worker)?

- ◯ yes
- ◯ no
- ◯ don't know

Person 6: Does this person currently work in any of the following high-risk settings for COVID-19 transmission?

- ☐ healthcare setting (hospital, clinic, urgent care, etc.)
- ☐ dense residential setting (nursing home, other long-term care facility)
- ☐ prison or jail
- ☐ meatpacking facility
- ☐ shipping or distribution facility
- ☐ high-volume retail facility (grocery store, etc.)
- ☐ don't know

Person 6: Does this person's employer offer them any of the following benefits at their current main job?

- ☐ paid sick leave
- ☐ paid vacation/personal leave
- ☐ health insurance
- ☐ disability insurance
- ☐ retirement plan
- ☐ other
- ☐ don't know

(Select all that apply.)

Person 6: On a scale of 0 (definitely not going to happen) to 10 (definitely going to happen), how likely is it that this person will lose their job because of the COVID-19 pandemic?

____

Person 6: On a scale of 0 (definitely not going to happen) to 10 (definitely going to happen), how likely is it that this person will receive fewer work hours at their job because of the COVID-19 pandemic?

____

---

	always (100%)	most of the time (75%)	half of the time (50%)	less than half of the time (25%)	never (0%)	don't know
Person 6: How often is this person required to work from outside of the home currently?	○	○	○	○	○	○
Person 6: How regularly is this person in close physical contact with co-workers during their work outside of the home currently?	○	○	○	○	○	○
Person 6: How regularly is this person in close physical contact with clients during their work outside of the home currently?	○	○	○	○	○	○

Person 6: Does this person plan to get a vaccine for COVID-19 when one becomes available?

- ○ yes
- ○ no
- ○ don't know

Person 6: Has this person had any symptoms (cough, fever, difficulty breathing, fatigue, body aches, diarrhea, runny nose, loss of smell or taste) consistent with COVID-19 in the last two weeks?

- ○ yes
- ○ no
- ○ don't know

Person 6: When did this person's symptoms begin?

____

Person 6: Is this person worried that they may have had COVID-19 because of their symptoms?	◯ yes<br>◯ no<br>◯ don't know
Person 6: Did this person experience any bias or discrimination because of their symptoms?	◯ yes<br>◯ no<br>◯ don't know
Person 6: What did this person do in response to their symptoms?	☐ nothing<br>☐ took over the counter medication (ibuprofen, acetaminophen, etc.)<br>☐ communicated with a health care provider over the phone<br>☐ visited a health care provider's office<br>☐ visited a retail clinic or pharmacy<br>☐ visited urgent care (FASTMed, etc.)<br>☐ visited the emergency room<br>☐ was admitted to the hospital<br>☐ other<br>☐ don't know<br>(Select all that apply.)
Person 6: Please specify what other action this person took in response to their symptoms.	__________________________________
Person 6: Did a health care provider tell this person that they may have COVID-19?	◯ yes<br>◯ no<br>◯ don't know
Person 6: If this person received a COVID-19 test due to their symptoms, what was the result?	◯ pending<br>◯ positive<br>◯ negative<br>◯ inconclusive<br>◯ did not receive a test<br>◯ don't know
Person 6: How many days was this person admitted to the hospital?	__________________________________
Person 6: Did this person receive any of the following interventions during their hospital admission?	☐ extra oxygen in your nose<br>☐ treatment in the intensive care unit (ICU)<br>☐ mechanical ventilation (intubation or a breathing tube)<br>☐ don't know
Person 6: Has this person returned to their normal health at this time?	◯ yes<br>◯ no<br>◯ don't know
Person 6: Which of the following did this person do to protect their friends and family after their symptoms began?	☐ wore a mask more frequently<br>☐ washed your hands with soap and water more frequently<br>☐ used hand sanitizer more frequently<br>☐ isolated yourself in your home more frequently<br>☐ stayed home more frequently<br>☐ wore disposable gloves more frequently<br>☐ don't know

For each additional person in the your household, please provide the following information.

Person 7: What is your relationship to this person?	◯ partner or spouse<br>◯ child<br>◯ parent<br>◯ sibling<br>◯ other family member<br>◯ in-home childcare provider or other caregiver<br>◯ other
Person 7: Please specify your relationship with this person.	__________________________
Person 7: What is this person's age?	__________________________<br>(Please specify their age in years)
Person 7: What is this person's sex?	◯ Female<br>◯ Male<br>◯ Other
Person 7: What is this person's race?	☐ American Indian or Alaska Native<br>☐ Asian<br>☐ Black or African American<br>☐ Native Hawaiian or Pacific Islander<br>☐ White<br>☐ Other<br>☐ don't know<br>(Select all that apply.)
Person 7: What is this person's ethnicity?	◯ Hispanic or Latino<br>◯ Not Hispanic or Latino<br>◯ Other<br>◯ don't know
Person 7: What is the highest level of education or schooling this person has completed?	◯ never attended school<br>◯ kindergarten - 8th grade<br>◯ some high school<br>◯ high school equivalency (GED)<br>◯ high school graduate<br>◯ some college<br>◯ college graduate<br>◯ graduate school or more<br>◯ don't know
Person 7: Which of the following best fit this person's current work situation?	◯ works full time<br>◯ works part time<br>◯ is looking for work/employment<br>◯ retired<br>◯ homemaker<br>◯ student<br>◯ on maternity/paternity leave<br>◯ on illness/sick leave<br>◯ on disability<br>◯ other<br>◯ don't know
Person 7: Does this person currently consider themselves self-employed (including as an independent contractor or gig-economy worker)?	◯ yes<br>◯ no<br>◯ don't know

Person 7: Does this person currently work in any of the following high-risk settings for COVID-19 transmission?

- ☐ healthcare setting (hospital, clinic, urgent care, etc.)
- ☐ dense residential setting (nursing home, other long-term care facility)
- ☐ prison or jail
- ☐ meatpacking facility
- ☐ shipping or distribution facility
- ☐ high-volume retail facility (grocery store, etc.)
- ☐ don't know

Person 7: Does this person's employer offer them any of the following benefits at their current main job?

- ☐ paid sick leave
- ☐ paid vacation/personal leave
- ☐ health insurance
- ☐ disability insurance
- ☐ retirement plan
- ☐ other
- ☐ don't know

(Select all that apply.)

Person 7: On a scale of 0 (definitely not going to happen) to 10 (definitely going to happen), how likely is it that this person will lose their job because of the COVID-19 pandemic?

____

Person 7: On a scale of 0 (definitely not going to happen) to 10 (definitely going to happen), how likely is it that this person will receive fewer work hours at their job because of the COVID-19 pandemic?

____

	always (100%)	most of the time (75%)	half of the time (50%)	less than half of the time (25%)	never (0%)	don't know
Person 7: How often is this person required to work from outside of the home currently?	○	○	○	○	○	○
Person 7: How regularly is this person in close physical contact with co-workers during their work outside of the home currently?	○	○	○	○	○	○
Person 7: How regularly is this person in close physical contact with clients during their work outside of the home currently?	○	○	○	○	○	○

Person 7: Does this person plan to get a vaccine for COVID-19 when one becomes available?

- ○ yes
- ○ no
- ○ don't know

Person 7: Has this person had any symptoms (cough, fever, difficulty breathing, fatigue, body aches, diarrhea, runny nose, loss of smell or taste) consistent with COVID-19 in the last two weeks?

- ○ yes
- ○ no
- ○ don't know

Person 7: When did this person's symptoms begin?

____

Person 7: Is this person worried that they may have had COVID-19 because of their symptoms?	◯ yes<br>◯ no<br>◯ don't know
Person 7: Did this person experience any bias or discrimination because of their symptoms?	◯ yes<br>◯ no<br>◯ don't know
Person 7: What did this person do in response to their symptoms?	☐ nothing<br>☐ took over the counter medication (ibuprofen, acetaminophen, etc.)<br>☐ communicated with a health care provider over the phone<br>☐ visited a health care provider's office<br>☐ visited a retail clinic or pharmacy<br>☐ visited urgent care (FASTMed, etc.)<br>☐ visited the emergency room<br>☐ was admitted to the hospital<br>☐ other<br>☐ don't know<br>(Select all that apply.)
Person 7: Please specify what other action this person took in response to their symptoms.	__________________________________
Person 7: Did a health care provider tell this person that they may have COVID-19?	◯ yes<br>◯ no<br>◯ don't know
Person 7: If this person received a COVID-19 test due to their symptoms, what was the result?	◯ pending<br>◯ positive<br>◯ negative<br>◯ inconclusive<br>◯ did not receive a test<br>◯ don't know
Person 7: How many days was this person admitted to the hospital?	__________________________________
Person 7: Did this person receive any of the following interventions during their hospital admission?	☐ extra oxygen in your nose<br>☐ treatment in the intensive care unit (ICU)<br>☐ mechanical ventilation (intubation or a breathing tube)<br>☐ don't know
Person 7: Has this person returned to their normal health at this time?	◯ yes<br>◯ no<br>◯ don't know
Person 7: Which of the following did this person do to protect their friends and family after their symptoms began?	☐ wore a mask more frequently<br>☐ washed your hands with soap and water more frequently<br>☐ used hand sanitizer more frequently<br>☐ isolated yourself in your home more frequently<br>☐ stayed home more frequently<br>☐ wore disposable gloves more frequently<br>☐ don't know

For each additional person in the your household, please provide the following information.

Person 8: What is your relationship to this person?	◯ partner or spouse<br>◯ child<br>◯ parent<br>◯ sibling<br>◯ other family member<br>◯ in-home childcare provider or other caregiver<br>◯ other
Person 8: Please specify your relationship with this person.	__________________________
Person 8: What is this person's age?	__________________________<br>(Please specify their age in years)
Person 8: What is this person's sex?	◯ Female<br>◯ Male<br>◯ Other
Person 8: What is this person's race?	☐ American Indian or Alaska Native<br>☐ Asian<br>☐ Black or African American<br>☐ Native Hawaiian or Pacific Islander<br>☐ White<br>☐ Other<br>☐ don't know<br>(Select all that apply.)
Person 8: What is this person's ethnicity?	◯ Hispanic or Latino<br>◯ Not Hispanic or Latino<br>◯ Other<br>◯ don't know
Person 8: What is the highest level of education or schooling this person has completed?	◯ never attended school<br>◯ kindergarten - 8th grade<br>◯ some high school<br>◯ high school equivalency (GED)<br>◯ high school graduate<br>◯ some college<br>◯ college graduate<br>◯ graduate school or more<br>◯ don't know
Person 8: Which of the following best fit this person's current work situation?	◯ works full time<br>◯ works part time<br>◯ is looking for work/employment<br>◯ retired<br>◯ homemaker<br>◯ student<br>◯ on maternity/paternity leave<br>◯ on illness/sick leave<br>◯ on disability<br>◯ other<br>◯ don't know
Person 8: Does this person currently consider themselves self-employed (including as an independent contractor or gig-economy worker)?	◯ yes<br>◯ no<br>◯ don't know

Person 8: Does this person currently work in any of the following high-risk settings for COVID-19 transmission?	☐ healthcare setting (hospital, clinic, urgent care, etc.)<br>☐ dense residential setting (nursing home, other long-term care facility)<br>☐ prison or jail<br>☐ meatpacking facility<br>☐ shipping or distribution facility<br>☐ high-volume retail facility (grocery store, etc.)<br>☐ don't know
Person 8: Does this person's employer offer them any of the following benefits at their current main job?	☐ paid sick leave<br>☐ paid vacation/personal leave<br>☐ health insurance<br>☐ disability insurance<br>☐ retirement plan<br>☐ other<br>☐ don't know<br>(Select all that apply.)
Person 8: On a scale of 0 (definitely not going to happen) to 10 (definitely going to happen), how likely is it that this person will lose their job because of the COVID-19 pandemic?	______________________________
Person 8: On a scale of 0 (definitely not going to happen) to 10 (definitely going to happen), how likely is it that this person will receive fewer work hours at their job because of the COVID-19 pandemic?	______________________________

	always (100%)	most of the time (75%)	half of the time (50%)	less than half of the time (25%)	never (0%)	don't know
Person 8: How often is this person required to work from outside of the home currently?	◯	◯	◯	◯	◯	◯
Person 8: How regularly is this person in close physical contact with co-workers during their work outside of the home currently?	◯	◯	◯	◯	◯	◯
Person 8: How regularly is this person in close physical contact with clients during their work outside of the home currently?	◯	◯	◯	◯	◯	◯

Person 8: Does this person plan to get a vaccine for COVID-19 when one becomes available?	◯ yes<br>◯ no<br>◯ don't know
Person 8: Has this person had any symptoms (cough, fever, difficulty breathing, fatigue, body aches, diarrhea, runny nose, loss of smell or taste) consistent with COVID-19 in the last two weeks?	◯ yes<br>◯ no<br>◯ don't know
Person 8: When did this person's symptoms begin?	______________________________

Person 8: Is this person worried that they may have had COVID-19 because of their symptoms?	◯ yes<br>◯ no<br>◯ don't know
Person 8: Did this person experience any bias or discrimination because of their symptoms?	◯ yes<br>◯ no<br>◯ don't know
Person 8: What did this person do in response to their symptoms?	☐ nothing<br>☐ took over the counter medication (ibuprofen, acetaminophen, etc.)<br>☐ communicated with a health care provider over the phone<br>☐ visited a health care provider's office<br>☐ visited a retail clinic or pharmacy<br>☐ visited urgent care (FASTMed, etc.)<br>☐ visited the emergency room<br>☐ was admitted to the hospital<br>☐ other<br>☐ don't know<br>(Select all that apply.)
Person 8: Please specify what other action this person took in response to their symptoms.	__________________________________
Person 8: Did a health care provider tell this person that they may have COVID-19?	◯ yes<br>◯ no<br>◯ don't know
Person 8: If this person received a COVID-19 test due to their symptoms, what was the result?	◯ pending<br>◯ positive<br>◯ negative<br>◯ inconclusive<br>◯ did not receive a test<br>◯ don't know
Person 8: How many days was this person admitted to the hospital?	__________________________________
Person 8: Did this person receive any of the following interventions during their hospital admission?	☐ extra oxygen in your nose<br>☐ treatment in the intensive care unit (ICU)<br>☐ mechanical ventilation (intubation or a breathing tube)<br>☐ don't know
Person 8: Has this person returned to their normal health at this time?	◯ yes<br>◯ no<br>◯ don't know
Person 8: Which of the following did this person do to protect their friends and family after their symptoms began?	☐ wore a mask more frequently<br>☐ washed your hands with soap and water more frequently<br>☐ used hand sanitizer more frequently<br>☐ isolated yourself in your home more frequently<br>☐ stayed home more frequently<br>☐ wore disposable gloves more frequently<br>☐ don't know

For each additional person in the your household, please provide the following information.

Person 9: What is your relationship to this person?

- ◯ partner or spouse
- ◯ child
- ◯ parent
- ◯ sibling
- ◯ other family member
- ◯ in-home childcare provider or other caregiver
- ◯ other

Person 9: Please specify your relationship with this person.

____

Person 9: What is this person's age?

____
(Please specify their age in years)

Person 9: What is this person's sex?

- ◯ Female
- ◯ Male
- ◯ Other

Person 9: What is this person's race?

- ☐ American Indian or Alaska Native
- ☐ Asian
- ☐ Black or African American
- ☐ Native Hawaiian or Pacific Islander
- ☐ White
- ☐ Other
- ☐ don't know

(Select all that apply.)

Person 9: What is this person's ethnicity?

- ◯ Hispanic or Latino
- ◯ Not Hispanic or Latino
- ◯ Other
- ◯ don't know

Person 9: What is the highest level of education or schooling this person has completed?

- ◯ never attended school
- ◯ kindergarten - 8th grade
- ◯ some high school
- ◯ high school equivalency (GED)
- ◯ high school graduate
- ◯ some college
- ◯ college graduate
- ◯ graduate school or more
- ◯ don't know

Person 9: Which of the following best fit this person's current work situation?

- ◯ works full time
- ◯ works part time
- ◯ is looking for work/employment
- ◯ retired
- ◯ homemaker
- ◯ student
- ◯ on maternity/paternity leave
- ◯ on illness/sick leave
- ◯ on disability
- ◯ other
- ◯ don't know

Person 9: Does this person currently consider themselves self-employed (including as an independent contractor or gig-economy worker)?

- ◯ yes
- ◯ no
- ◯ don't know

Person 9: Does this person currently work in any of the following high-risk settings for COVID-19 transmission?

- ☐ healthcare setting (hospital, clinic, urgent care, etc.)
- ☐ dense residential setting (nursing home, other long-term care facility)
- ☐ prison or jail
- ☐ meatpacking facility
- ☐ shipping or distribution facility
- ☐ high-volume retail facility (grocery store, etc.)
- ☐ don't know

Person 9: Does this person's employer offer them any of the following benefits at their current main job?

- ☐ paid sick leave
- ☐ paid vacation/personal leave
- ☐ health insurance
- ☐ disability insurance
- ☐ retirement plan
- ☐ other
- ☐ don't know

(Select all that apply.)

Person 9: On a scale of 0 (definitely not going to happen) to 10 (definitely going to happen), how likely is it that this person will lose their job because of the COVID-19 pandemic?

____

Person 9: On a scale of 0 (definitely not going to happen) to 10 (definitely going to happen), how likely is it that this person will receive fewer work hours at their job because of the COVID-19 pandemic?

____

---

	always (100%)	most of the time (75%)	half of the time (50%)	less than half of the time (25%)	never (0%)	don't know
Person 9: How often is this person required to work from outside of the home currently?	◯	◯	◯	◯	◯	◯
Person 9: How regularly is this person in close physical contact with co-workers during their work outside of the home currently?	◯	◯	◯	◯	◯	◯
Person 9: How regularly is this person in close physical contact with clients during their work outside of the home currently?	◯	◯	◯	◯	◯	◯

Person 9: Does this person plan to get a vaccine for COVID-19 when one becomes available?

- ◯ yes
- ◯ no
- ◯ don't know

Person 9: Has this person had any symptoms (cough, fever, difficulty breathing, fatigue, body aches, diarrhea, runny nose, loss of smell or taste) consistent with COVID-19 in the last two weeks?

- ◯ yes
- ◯ no
- ◯ don't know

Person 9: When did this person's symptoms begin?

____

Person 9: Is this person worried that they may have had COVID-19 because of their symptoms?	◯ yes<br>◯ no<br>◯ don't know
Person 9: Did this person experience any bias or discrimination because of their symptoms?	◯ yes<br>◯ no<br>◯ don't know
Person 9: What did this person do in response to their symptoms?	☐ nothing<br>☐ took over the counter medication (ibuprofen, acetaminophen, etc.)<br>☐ communicated with a health care provider over the phone<br>☐ visited a health care provider's office<br>☐ visited a retail clinic or pharmacy<br>☐ visited urgent care (FASTMed, etc.)<br>☐ visited the emergency room<br>☐ was admitted to the hospital<br>☐ other<br>☐ don't know<br>(Select all that apply.)
Person 9: Please specify what other action this person took in response to their symptoms.	__________________________________
Person 9: Did a health care provider tell this person that they may have COVID-19?	◯ yes<br>◯ no<br>◯ don't know
Person 9: If this person received a COVID-19 test due to their symptoms, what was the result?	◯ pending<br>◯ positive<br>◯ negative<br>◯ inconclusive<br>◯ did not receive a test<br>◯ don't know
Person 9: How many days was this person admitted to the hospital?	__________________________________
Person 9: Did this person receive any of the following interventions during their hospital admission?	☐ extra oxygen in your nose<br>☐ treatment in the intensive care unit (ICU)<br>☐ mechanical ventilation (intubation or a breathing tube)<br>☐ don't know
Person 9: Has this person returned to their normal health at this time?	◯ yes<br>◯ no<br>◯ don't know
Person 9: Which of the following did this person do to protect their friends and family after their symptoms began?	☐ wore a mask more frequently<br>☐ washed your hands with soap and water more frequently<br>☐ used hand sanitizer more frequently<br>☐ isolated yourself in your home more frequently<br>☐ stayed home more frequently<br>☐ wore disposable gloves more frequently<br>☐ don't know

For each additional person in the your household, please provide the following information.

Person 10: What is your relationship to this person?	◯ partner or spouse<br>◯ child<br>◯ parent<br>◯ sibling<br>◯ other family member<br>◯ in-home childcare provider or other caregiver<br>◯ other
Person 10: Please specify your relationship with this person.	__________________________________
Person 10: What is this person's age?	__________________________________<br>(Please specify their age in years)
Person 10: What is this person's sex?	◯ Female<br>◯ Male<br>◯ Other
Person 10: What is this person's race?	☐ American Indian or Alaska Native<br>☐ Asian<br>☐ Black or African American<br>☐ Native Hawaiian or Pacific Islander<br>☐ White<br>☐ Other<br>☐ don't know<br>(Select all that apply.)
Person 10: What is this person's ethnicity?	◯ Hispanic or Latino<br>◯ Not Hispanic or Latino<br>◯ Other<br>◯ don't know
Person 10: What is the highest level of education or schooling this person has completed?	◯ never attended school<br>◯ kindergarten - 8th grade<br>◯ some high school<br>◯ high school equivalency (GED)<br>◯ high school graduate<br>◯ some college<br>◯ college graduate<br>◯ graduate school or more<br>◯ don't know
Person 10: Which of the following best fit this person's current work situation?	◯ works full time<br>◯ works part time<br>◯ is looking for work/employment<br>◯ retired<br>◯ homemaker<br>◯ student<br>◯ on maternity/paternity leave<br>◯ on illness/sick leave<br>◯ on disability<br>◯ other<br>◯ don't know
Person 10: Does this person currently consider themselves self-employed (including as an independent contractor or gig-economy worker)?	◯ yes<br>◯ no<br>◯ don't know

Person 10: Does this person currently work in any of the following high-risk settings for COVID-19 transmission?	☐ healthcare setting (hospital, clinic, urgent care, etc.)<br>☐ dense residential setting (nursing home, other long-term care facility)<br>☐ prison or jail<br>☐ meatpacking facility<br>☐ shipping or distribution facility<br>☐ high-volume retail facility (grocery store, etc.)<br>☐ don't know
Person 10: Does this person's employer offer them any of the following benefits at their current main job?	☐ paid sick leave<br>☐ paid vacation/personal leave<br>☐ health insurance<br>☐ disability insurance<br>☐ retirement plan<br>☐ other<br>☐ don't know<br>(Select all that apply.)
Person 10: On a scale of 0 (definitely not going to happen) to 10 (definitely going to happen), how likely is it that this person will lose their job because of the COVID-19 pandemic?	__________________________
Person 10: On a scale of 0 (definitely not going to happen) to 10 (definitely going to happen), how likely is it that this person will receive fewer work hours at their job because of the COVID-19 pandemic?	__________________________

	always (100%)	most of the time (75%)	half of the time (50%)	less than half of the time (25%)	never (0%)	don't know
Person 10: How often is this person required to work from outside of the home currently?	○	○	○	○	○	○
Person 10: How regularly is this person in close physical contact with co-workers during their work outside of the home currently?	○	○	○	○	○	○
Person 10: How regularly is this person in close physical contact with clients during their work outside of the home currently?	○	○	○	○	○	○

Person 10: Does this person plan to get a vaccine for COVID-19 when one becomes available?	○ yes<br>○ no<br>○ don't know
Person 10: Has this person had any symptoms (cough, fever, difficulty breathing, fatigue, body aches, diarrhea, runny nose, loss of smell or taste) consistent with COVID-19 in the last two weeks?	○ yes<br>○ no<br>○ don't know
Person 10: When did this person's symptoms begin?	__________________________

Person 10: Is this person worried that they may have had COVID-19 because of their symptoms?	◯ yes<br>◯ no<br>◯ don't know
Person 10: Did this person experience any bias or discrimination because of their symptoms?	◯ yes<br>◯ no<br>◯ don't know
Person 10: What did this person do in response to their symptoms?	☐ nothing<br>☐ took over the counter medication (ibuprofen, acetaminophen, etc.)<br>☐ communicated with a health care provider over the phone<br>☐ visited a health care provider's office<br>☐ visited a retail clinic or pharmacy<br>☐ visited urgent care (FASTMed, etc.)<br>☐ visited the emergency room<br>☐ was admitted to the hospital<br>☐ other<br>☐ don't know<br>(Select all that apply.)
Person 10: Please specify what other action this person took in response to their symptoms.	__________________________________
Person 10: Did a health care provider tell this person that they may have COVID-19?	◯ yes<br>◯ no<br>◯ don't know
Person 10: If this person received a COVID-19 test due to their symptoms, what was the result?	◯ pending<br>◯ positive<br>◯ negative<br>◯ inconclusive<br>◯ did not receive a test<br>◯ don't know
Person 10: How many days was this person admitted to the hospital?	__________________________________
Person 10: Did this person receive any of the following interventions during their hospital admission?	☐ extra oxygen in your nose<br>☐ treatment in the intensive care unit (ICU)<br>☐ mechanical ventilation (intubation or a breathing tube)<br>☐ don't know
Person 10: Has this person returned to their normal health at this time?	◯ yes<br>◯ no<br>◯ don't know
Person 10: Which of the following did this person do to protect their friends and family after their symptoms began?	☐ wore a mask more frequently<br>☐ washed your hands with soap and water more frequently<br>☐ used hand sanitizer more frequently<br>☐ isolated yourself in your home more frequently<br>☐ stayed home more frequently<br>☐ wore disposable gloves more frequently<br>☐ don't know

For each additional person in the your household, please provide the following information.

Person 11: What is your relationship to this person?	◯ partner or spouse<br>◯ child<br>◯ parent<br>◯ sibling<br>◯ other family member<br>◯ in-home childcare provider or other caregiver<br>◯ other
Person 11: Please specify your relationship with this person.	________________________________
Person 11: What is this person's age?	________________________________<br>(Please specify their age in years)
Person 11: What is this person's sex?	◯ Female<br>◯ Male<br>◯ Other
Person 11: What is this person's race?	☐ American Indian or Alaska Native<br>☐ Asian<br>☐ Black or African American<br>☐ Native Hawaiian or Pacific Islander<br>☐ White<br>☐ Other<br>☐ don't know<br>(Select all that apply.)
Person 11: What is this person's ethnicity?	◯ Hispanic or Latino<br>◯ Not Hispanic or Latino<br>◯ Other<br>◯ don't know
Person 11: What is the highest level of education or schooling this person has completed?	◯ never attended school<br>◯ kindergarten - 8th grade<br>◯ some high school<br>◯ high school equivalency (GED)<br>◯ high school graduate<br>◯ some college<br>◯ college graduate<br>◯ graduate school or more<br>◯ don't know
Person 11: Which of the following best fit this person's current work situation?	◯ works full time<br>◯ works part time<br>◯ is looking for work/employment<br>◯ retired<br>◯ homemaker<br>◯ student<br>◯ on maternity/paternity leave<br>◯ on illness/sick leave<br>◯ on disability<br>◯ other<br>◯ don't know
Person 11: Does this person currently consider themselves self-employed (including as an independent contractor or gig-economy worker)?	◯ yes<br>◯ no<br>◯ don't know

Person 11: Does this person currently work in any of the following high-risk settings for COVID-19 transmission?	☐ healthcare setting (hospital, clinic, urgent care, etc.)<br>☐ dense residential setting (nursing home, other long-term care facility)<br>☐ prison or jail<br>☐ meatpacking facility<br>☐ shipping or distribution facility<br>☐ high-volume retail facility (grocery store, etc.)<br>☐ don't know
Person 11: Does this person's employer offer them any of the following benefits at their current main job?	☐ paid sick leave<br>☐ paid vacation/personal leave<br>☐ health insurance<br>☐ disability insurance<br>☐ retirement plan<br>☐ other<br>☐ don't know<br>(Select all that apply.)
Person 11: On a scale of 0 (definitely not going to happen) to 10 (definitely going to happen), how likely is it that this person will lose their job because of the COVID-19 pandemic?	______________________________
Person 11: On a scale of 0 (definitely not going to happen) to 10 (definitely going to happen), how likely is it that this person will receive fewer work hours at their job because of the COVID-19 pandemic?	______________________________

	always (100%)	most of the time (75%)	half of the time (50%)	less than half of the time (25%)	never (0%)	don't know
Person 11: How often is this person required to work from outside of the home currently?	◯	◯	◯	◯	◯	◯
Person 11: How regularly is this person in close physical contact with co-workers during their work outside of the home currently?	◯	◯	◯	◯	◯	◯
Person 11: How regularly is this person in close physical contact with clients during their work outside of the home currently?	◯	◯	◯	◯	◯	◯

Person 11: Does this person plan to get a vaccine for COVID-19 when one becomes available?	◯ yes<br>◯ no<br>◯ don't know
Person 11: Has this person had any symptoms (cough, fever, difficulty breathing, fatigue, body aches, diarrhea, runny nose, loss of smell or taste) consistent with COVID-19 in the last two weeks?	◯ yes<br>◯ no<br>◯ don't know
Person 11: When did this person's symptoms begin?	______________________________

Person 11: Is this person worried that they may have had COVID-19 because of their symptoms?	◯ yes<br>◯ no<br>◯ don't know
Person 11: Did this person experience any bias or discrimination because of their symptoms?	◯ yes<br>◯ no<br>◯ don't know
Person 11: What did this person do in response to their symptoms?	☐ nothing<br>☐ took over the counter medication (ibuprofen, acetaminophen, etc.)<br>☐ communicated with a health care provider over the phone<br>☐ visited a health care provider's office<br>☐ visited a retail clinic or pharmacy<br>☐ visited urgent care (FASTMed, etc.)<br>☐ visited the emergency room<br>☐ was admitted to the hospital<br>☐ other<br>☐ don't know<br>(Select all that apply.)
Person 11: Please specify what other action this person took in response to their symptoms.	__________________________________
Person 11: Did a health care provider tell this person that they may have COVID-19?	◯ yes<br>◯ no<br>◯ don't know
Person 11: If this person received a COVID-19 test due to their symptoms, what was the result?	◯ pending<br>◯ positive<br>◯ negative<br>◯ inconclusive<br>◯ did not receive a test<br>◯ don't know
Person 11: How many days was this person admitted to the hospital?	__________________________________
Person 11: Did this person receive any of the following interventions during their hospital admission?	☐ extra oxygen in your nose<br>☐ treatment in the intensive care unit (ICU)<br>☐ mechanical ventilation (intubation or a breathing tube)<br>☐ don't know
Person 11: Has this person returned to their normal health at this time?	◯ yes<br>◯ no<br>◯ don't know
Person 11: Which of the following did this person do to protect their friends and family after their symptoms began?	☐ wore a mask more frequently<br>☐ washed your hands with soap and water more frequently<br>☐ used hand sanitizer more frequently<br>☐ isolated yourself in your home more frequently<br>☐ stayed home more frequently<br>☐ wore disposable gloves more frequently<br>☐ don't know

For each additional person in the your household, please provide the following information.

Person 12: What is your relationship to this person?	◯ partner or spouse<br>◯ child<br>◯ parent<br>◯ sibling<br>◯ other family member<br>◯ in-home childcare provider or other caregiver<br>◯ other
Person 12: Please specify your relationship with this person.	________________________________
Person 12: What is this person's age?	________________________________<br>(Please specify their age in years)
Person 12: What is this person's sex?	◯ Female<br>◯ Male<br>◯ Other
Person 12: What is this person's race?	☐ American Indian or Alaska Native<br>☐ Asian<br>☐ Black or African American<br>☐ Native Hawaiian or Pacific Islander<br>☐ White<br>☐ Other<br>☐ don't know<br>(Select all that apply.)
Person 12: What is this person's ethnicity?	◯ Hispanic or Latino<br>◯ Not Hispanic or Latino<br>◯ Other<br>◯ don't know
Person 12: What is the highest level of education or schooling this person has completed?	◯ never attended school<br>◯ kindergarten - 8th grade<br>◯ some high school<br>◯ high school equivalency (GED)<br>◯ high school graduate<br>◯ some college<br>◯ college graduate<br>◯ graduate school or more<br>◯ don't know
Person 12: Which of the following best fit this person's current work situation?	◯ works full time<br>◯ works part time<br>◯ is looking for work/employment<br>◯ retired<br>◯ homemaker<br>◯ student<br>◯ on maternity/paternity leave<br>◯ on illness/sick leave<br>◯ on disability<br>◯ other<br>◯ don't know
Person 12: Does this person currently consider themselves self-employed (including as an independent contractor or gig-economy worker)?	◯ yes<br>◯ no<br>◯ don't know

Person 12: Does this person currently work in any of the following high-risk settings for COVID-19 transmission?	☐ healthcare setting (hospital, clinic, urgent care, etc.)<br>☐ dense residential setting (nursing home, other long-term care facility)<br>☐ prison or jail<br>☐ meatpacking facility<br>☐ shipping or distribution facility<br>☐ high-volume retail facility (grocery store, etc.)<br>☐ don't know
Person 12: Does this person's employer offer them any of the following benefits at their current main job?	☐ paid sick leave<br>☐ paid vacation/personal leave<br>☐ health insurance<br>☐ disability insurance<br>☐ retirement plan<br>☐ other<br>☐ don't know<br>(Select all that apply.)
Person 12: On a scale of 0 (definitely not going to happen) to 10 (definitely going to happen), how likely is it that this person will lose their job because of the COVID-19 pandemic?	______________________
Person 12: On a scale of 0 (definitely not going to happen) to 10 (definitely going to happen), how likely is it that this person will receive fewer work hours at their job because of the COVID-19 pandemic?	______________________

	always (100%)	most of the time (75%)	half of the time (50%)	less than half of the time (25%)	never (0%)	don't know
Person 12: How often is this person required to work from outside of the home currently?	○	○	○	○	○	○
Person 12: How regularly is this person in close physical contact with co-workers during their work outside of the home currently?	○	○	○	○	○	○
Person 12: How regularly is this person in close physical contact with clients during their work outside of the home currently?	○	○	○	○	○	○

Person 12: Does this person plan to get a vaccine for COVID-19 when one becomes available?	○ yes<br>○ no<br>○ don't know
Person 12: Has this person had any symptoms (cough, fever, difficulty breathing, fatigue, body aches, diarrhea, runny nose, loss of smell or taste) consistent with COVID-19 in the last two weeks?	○ yes<br>○ no<br>○ don't know
Person 12: When did this person's symptoms begin?	______________________

Person 12: Is this person worried that they may have had COVID-19 because of their symptoms?	◯ yes<br>◯ no<br>◯ don't know
Person 12: Did this person experience any bias or discrimination because of their symptoms?	◯ yes<br>◯ no<br>◯ don't know
Person 12: What did this person do in response to their symptoms?	☐ nothing<br>☐ took over the counter medication (ibuprofen, acetaminophen, etc.)<br>☐ communicated with a health care provider over the phone<br>☐ visited a health care provider's office<br>☐ visited a retail clinic or pharmacy<br>☐ visited urgent care (FASTMed, etc.)<br>☐ visited the emergency room<br>☐ was admitted to the hospital<br>☐ other<br>☐ don't know<br>(Select all that apply.)
Person 12: Please specify what other action this person took in response to their symptoms.	__________________________________
Person 12: Did a health care provider tell this person that they may have COVID-19?	◯ yes<br>◯ no<br>◯ don't know
Person 12: If this person received a COVID-19 test due to their symptoms, what was the result?	◯ pending<br>◯ positive<br>◯ negative<br>◯ inconclusive<br>◯ did not receive a test<br>◯ don't know
Person 12: How many days was this person admitted to the hospital?	__________________________________
Person 12: Did this person receive any of the following interventions during their hospital admission?	☐ extra oxygen in your nose<br>☐ treatment in the intensive care unit (ICU)<br>☐ mechanical ventilation (intubation or a breathing tube)<br>☐ don't know
Person 12: Has this person returned to their normal health at this time?	◯ yes<br>◯ no<br>◯ don't know
Person 12: Which of the following did this person do to protect their friends and family after their symptoms began?	☐ wore a mask more frequently<br>☐ washed your hands with soap and water more frequently<br>☐ used hand sanitizer more frequently<br>☐ isolated yourself in your home more frequently<br>☐ stayed home more frequently<br>☐ wore disposable gloves more frequently<br>☐ don't know

Please provide the following information about your mental health and wellness.

How serious a problem would you say the COVID-19 pandemic is for you personally right now?

- ◯ very serious
- ◯ somewhat serious
- ◯ not too serious
- ◯ not at all serious

How serious a problem would you say the COVID-19 pandemic is for people in your community right now?

- ◯ very serious
- ◯ somewhat serious
- ◯ not too serious
- ◯ not at all serious

How serious a problem would you say the COVID-19 pandemic is for people in the United States right now?

- ◯ very serious
- ◯ somewhat serious
- ◯ not too serious
- ◯ not at all serious

How serious a problem would you say the COVID-19 pandemic is for people around the world right now?

- ◯ very serious
- ◯ somewhat serious
- ◯ not too serious
- ◯ not at all serious

The COVID-19 pandemic may cause challenges for some people regardless of whether they are infected. How concerned are you about each of the following things?

	not at all concerned	not too concerned	somewhat concerned	very concerned
Getting the healthcare you need (including care for mental health)	◯	◯	◯	◯
Having a place to live	◯	◯	◯	◯
Being able to interact with other people	◯	◯	◯	◯
Getting food, water, and other household supplies	◯	◯	◯	◯
Getting medication	◯	◯	◯	◯
Having transportation to get where you need to go	◯	◯	◯	◯
Caring for your family and friends	◯	◯	◯	◯

During the last two weeks, how often have you been bothered by the following problems?

	not at all	several days	more than half of the days	nearly every day
Feeling nervous, anxious, or on edge	◯	◯	◯	◯
Not being able to stop or control worrying	◯	◯	◯	◯
Worrying too much about different things	◯	◯	◯	◯

Trouble relaxing	◯	◯	◯	◯
Being so restless that it's hard to sit still	◯	◯	◯	◯
Becoming easily annoyed or irritable	◯	◯	◯	◯
Feeling afraid as if something awful might happen	◯	◯	◯	◯

How difficult have these problems made it for you to do your work, take care of things at home, or get along with other people?

◯ not difficult
◯ somewhat difficult
◯ very difficult
◯ extremely difficult

During the last two weeks, how often have you experienced the following behaviors and feelings?

	Rarely or none of the time (less than 1 day)	Some or a little of the time (1-2 days)	Occasionally or a moderate amount of the time (3-4 days)	Most or all of the time (5-7 days)
I was bothered by things that usually don't bother me.	◯	◯	◯	◯
I did not feel like eating; my appetite was poor.	◯	◯	◯	◯
I felt that I could not shake off the blues even with help from my family or friends.	◯	◯	◯	◯
I felt I was just as good as other people.	◯	◯	◯	◯
I had trouble keeping my mind on what I was doing.	◯	◯	◯	◯
I felt depressed.	◯	◯	◯	◯
I felt that everything I did was an effort.	◯	◯	◯	◯
I felt hopeful about the future.	◯	◯	◯	◯
I thought my life had been a failure.	◯	◯	◯	◯
I felt fearful.	◯	◯	◯	◯
My sleep was restless.	◯	◯	◯	◯
I was happy.	◯	◯	◯	◯
I talked less than usual.	◯	◯	◯	◯
I felt lonely.	◯	◯	◯	◯
People were unfriendly.	◯	◯	◯	◯
I enjoyed life.	◯	◯	◯	◯
I had crying spells.	◯	◯	◯	◯

I felt sad.	◯	◯	◯	◯
I felt that people disliked me.	◯	◯	◯	◯
I could not "get going".	◯	◯	◯	◯

Are you willing to provide a painless nasal swab and painless blood sample for this study of COVID-19 in Chatham county?

◯ Yes
◯ No

How did you complete this survey?

◯ on a computer (laptop or desktop)
◯ on a mobile device (cell phone or tablet)
◯ on a phone with an interviewer
◯ other

¿Cuál es su raza?

☐ Indio americano o nativo de Alaska
☐ Asiático
☐ Negro o afroestadounidense
☐ Nativo de Hawái o de las islas del Pacifico
☐ Blanco
☐ Otra
☐ Prefiero no responder
(Seleccione todo lo que corresponda.)

¿Cuál es su identidad étnica?

◯ Hispano o latino
◯ Ni hispano ni latino
◯ Otra
◯ Prefiero no responder

¿Qué nivel de educación tiene usted?

◯ No tiene educación formal
◯ Kinder a 8° grado
◯ Estudios de educación secundaria
◯ Equivalencia de educación secundaria
◯ Diploma de educación secundaria
◯ Estudios de educación superior
◯ Título universitario
◯ Estudios de posgrado o más

¿Fuma usted cigarillos, cigarros o pipa a diario?

◯ Sí
◯ No

¿A qué edad comenzó usted a fumar con regularidad?

____

En promedio ¿cuántos cigarrillos, cigarros o pipa fuma usted al día desde que comenzó a fumar?

◯ 0
◯ 1-10
◯ 11-20
◯ 21-30
◯ 31-40
◯ 41-50
◯ 51-60
◯ 61 o más

¿Fumaba usted cigarrillos, cigarros o pipas a diario antes?

◯ Sí
◯ No

¿A qué edad comenzó usted a fumar con regularidad?

____

¿Hace cuántos años que dejó de fumar?	__________________________________
En promedio ¿cuántos cigarrillos, cigarros o pipas fumaba al día cuando fumaba?	◯ 0<br>◯ 1-10<br>◯ 11-20<br>◯ 21-30<br>◯ 31-40<br>◯ 41-50<br>◯ 51-60<br>◯ 61 o más
¿Fuma usted cigarrillos electrónicos o de vapor?	◯ Sí<br>◯ No
¿A qué edad comenzó usted a fumar cigarrillos electrónicos con regularidad?	__________________________________
En promedio ¿cuántas bocanadas de cigarrillo electrónico o de vapor inhala usted al día?	◯ 0-25<br>◯ 26-50<br>◯ 51-75<br>◯ 76-100<br>◯ 101-125<br>◯ 126-150<br>◯ 151-175<br>◯ 176-200<br>◯ 201-225<br>◯ 226-250<br>◯ 251 o más
¿Fumaba usted cigarrillos electrónicos o de vapor antes?	◯ Sí<br>◯ No
¿A qué edad comenzó usted a fumar cigarrillos electrónicos con regularidad?	__________________________________
¿Hace cuántos años que dejó de fumar cigarrillos electrónicos?	__________________________________
En promedio ¿cuántas bocanadas de cigarrillo electrónico (o de otros productos de vapor) inhalaba usted al día?	◯ 0-25<br>◯ 26-50<br>◯ 51-75<br>◯ 76-100<br>◯ 101-125<br>◯ 126-150<br>◯ 151-175<br>◯ 176-200<br>◯ 201-225<br>◯ 226-250<br>◯ 251 o más
¿Toma usted bebidas alcohólicas al menos una vez a la semana?	◯ Yes<br>◯ No
¿A qué edad comenzó usted a tomar bebidas alcohólicas con regularidad?	__________________________________

En los últimos 30 días, incluido el día de hoy ¿cuántos días tomó usted una o varias bebidas alcohólicas?	__________________
En los últimos 30 días, ¿cuántas bebidas alcohólicas tomó por lo general cada día que bebió?	__________________ ((Una bebida es igual a 5 onzas de vino (una copa de vino), 12 onzas de cerveza (una lata o una botella de cerveza) o 1.5 onzas de licor (un trago o un coctel).))
¿Tomaba usted antes bebidas alcohólicas al menos una vez a la semana?	◯ Sí<br>◯ No
¿A qué edad comenzó usted a tomar bebidas alcohólicas con regularidad?	__________________
¿Hace cuántos años que dejó de tomar bebidas alcohólicas?	__________________
Durante un periodo normal de 30 dias, ¿cuántos días tomaba usted una o varias bebidas alcohólicas?	__________________
Durante un período normal de 30 días, ¿cuántos días tomaba usted una o varias bebidas alcohólicas? ¿Cuántas bebidas alcohólicas tomaba usted por lo general cada día que bebía?	__________________ ((Una bebida es igual a 5 onzas de vino (una copa de vino), 12 onzas de cerveza (una lata o una botella de cerveza) o 1.5 onzas de licor (un trago o un coctel).))
¿Hace usted al menos una vez a la semana actividades físicas tales como caminar rápido, trotar, andar en bicicleta, nadar, etc. hasta sudar, acelerar el pulso o jadear?	◯ Sí<br>◯ No
En promedio, ¿cuántos días a la semana hace usted este tipo de actividades?	◯ 1<br>◯ 2<br>◯ 3<br>◯ 4<br>◯ 5<br>◯ 6<br>◯ 7
En promedio, ¿cuántos minutos al día hace usted este tipo de actividades?	◯ 0-20<br>◯ 21-40<br>◯ 41-60<br>◯ 61 o más
Cuando hace ejercicio normalmente, ¿cómo calificaría usted su nivel promedio de esfuerzo?	◯ Leve / calentamiento<br>◯ Medio (puede conversar) / desarrollo aeróbico<br>◯ Intenso (pero puede esforzarse para continuar) / resistencia aeróbica<br>◯ Muy intenso (no puede conversar) / resistencia anaeróbica<br>◯ ™ Extremadamente intenso (jadeo, su cuerpo quiere parar) / velocidad, potencia

¿Cuál de las siguientes es la mejor descripción de su situación laboral antes de que comenzara la pandemia de COVID-19 en Carolina del Norte?	◯ Empleo a tiempo completo<br>◯ Empleo a tiempo parcial<br>◯ En busca de empleo<br>◯ Jubilado<br>◯ Ama de casa<br>◯ Estudiante<br>◯ Permiso de maternidad o paternidad<br>◯ Permiso por enfermedad<br>◯ Desempleado por discapacidad<br>◯ Otra
Antes de que comenzara la pandemia de COVID-19 en Carolina del Norte, ¿se consideraba usted empleado por cuenta propia (contratista independiente, trabajador esporádico (gig), etc.)?	◯ Sí<br>◯ No<br>◯ No sabe
¿Cuál de las siguientes es la mejor descripción de su empleo principal (empleo en el que pasaba la mayor parte del tiempo o que había tenido por más tiempo) antes de la pandemia de COVID-19 en Carolina del Norte?	◯ Administrativo<br>◯ Profesional<br>◯ Apoyo administrativo<br>◯ Servicio<br>◯ Agricultura, silvicultura o pesca<br>◯ Produccion de precisión, artesania o reparación<br>◯ Operario, fabricante u obrero<br>◯ Militar<br>◯ Estudiante<br>◯ Desempleado<br>◯ Otra
¿Cuántos años llevaba usted en su empleo principal?	__________________________________
En su empleo principal antes de la pandemia de COVID-19, ¿ofrecía su empleador alguno de los siguientes beneficios?	☐ Permiso por enfermedad con goce de sueldo<br>☐ Vacaciones o permiso personal con goce de sueldo<br>☐ Seguro de salud<br>☐ Seguro de discapacidad<br>☐ Plan de jubilación<br>☐ Otro<br>(Seleccione todo lo que corresponda.)
¿Ha cambiado su situación laboral desde que comenzó la pandemia de COVID-19 en Carolina del Norte?	◯ Sí<br>◯ No
¿Cuál de las siguientes es la mejor descripción de su situación laboral actual?	◯ Empleo a tiempo completo<br>◯ Empleo a tiempo parcial<br>◯ En busca de empleo<br>◯ Jubilado<br>◯ Ama de casa<br>◯ Estudiante<br>◯ Permiso de maternidad o paternidad<br>◯ Permiso por enfermedad<br>◯ Desempleado por discapacidad<br>◯ Otra
¿Se considera usted actualmente empleado por cuenta propia (contratista independiente, trabajador esporádico (gig), etc.)?	◯ Sí<br>◯ No<br>◯ no sabe

Del trabajo (o trabajos) que desempena actualmente, cual de las siguientes opciones describe mejor su trabajo principal (el trabajo en que pasa mas horas, o el trabajo en que ha trabajado mas tiempo)?	◯ Administrativo<br>◯ Profesional<br>◯ Apoyo administrativo<br>◯ Servicio<br>◯ Agricultura, silvicultura o pesca<br>◯ Produccion de precisión, artesania o reparación<br>◯ Operario, fabricante u obrero<br>◯ Militar<br>◯ Estudiante<br>◯ Desempleado<br>◯ Otra
¿Cuántos meses lleva usted en su empleo principal actual?	______________________
En su empleo principal actual, ¿ofrece su empleador alguno de los siguientes beneficios?	◯ Permiso por enfermedad con goce de sueldo<br>◯ Vacaciones o permiso personal con goce de sueldo<br>◯ Seguro de salud<br>◯ Seguro de discapacidad<br>◯ Plan de jubilación<br>◯ Otro<br>(Seleccione todo lo que corresponda.)
En una escala de 0 (definativamente no va a suceder) a 10 (definativamente va a suceder), ¿qué tan probable es que usted pierda su empleo debido a la pandemia de COVID-19?	______________________
En una escala de 0 (definativamente no va a suceder) a 10 (definativamente va a suceder), ¿qué tan probable es que le asignen menos horas de trabajo debido a la pandemia de COVID-19?	______________________

	Todo el tiempo (100%)	La mayor parte del tiempo (75%)	La mitad del tiempo (50%)	Menos de la mitad del tiempo (25%)	Nunca (0%)
Antes de la pandemia de COVID-19 en Carolina del Norte, ¿con qué frecuencia se le exigía que trabajara fuera de su residencia?	◯	◯	◯	◯	◯
Antes de la pandemia de COVID-19, ¿con qué frecuencia se encontraba usted físicamente cerca de sus compañeros de trabajo mientras trabajaba fuera de su residencia?	◯	◯	◯	◯	◯
Antes de la pandemia de COVID-19, ¿con qué frecuencia se encontraba usted físicamente cerca de los clientes mientras trabajaba fuera de su residencia?	◯	◯	◯	◯	◯

Antes de la pandemia de COVID-19, ¿con qué frecuencia tenía acceso a guantes desechables mientras trabajaba fuera de su residencia?	○	○	○	○	○
Antes de la pandemia de COVID-19, ¿con qué frecuencia tenía acceso a una mascarilla mientras trabajaba fuera de su residencia?	○	○	○	○	○
Antes de la pandemia de COVID-19, ¿con qué frecuencia llevaba usted guantes desechables mientras trabajaba fuera de su residencia?	○	○	○	○	○
Antes de la pandemia de COVID-19, ¿con qué frecuencia llevaba usted una mascarilla mientras trabajaba fuera de su residencia?	○	○	○	○	○
Antes de la pandemia de COVID-19,¿con qué frecuencia se lavaba las manos con agua y jabón mientras trabajaba fuera de su residencia?	○	○	○	○	○
Antes de la pandemia de COVID-19, ¿con qué frecuencia se desinfectaba las manos con desinfectante para manos mientras trabajaba fuera de su residencia?	○	○	○	○	○
Antes de la pandemia de COVID-19, ¿cuánto le preocupaba exponerse a la COVID-19 mientras trabajaba fuera de su residencia?	○	○	○	○	○

	Todo el tiempo (100%)	La mayor parte del tiempo (75%)	La mitad del tiempo (50%)	Menos de la mitad del tiempo (25%)	Nunca (0%)
Actualmente, ¿con qué frecuencia se le exige que trabaje fuera de su residencia?	○	○	○	○	○

Actualmente, ¿con qué frecuencia se encuentra usted físicamente cerca de sus compañeros de trabajo mientras trabaja fuera de su residencia?	◯	◯	◯	◯	◯
Actualmente, ¿con qué frecuencia se encuentra usted físicamente cerca de los clientes mientras trabaja fuera de su residencia?	◯	◯	◯	◯	◯
Actualmente, ¿con qué frecuencia tiene acceso a guantes desechables mientras trabaja fuera de su residencia?	◯	◯	◯	◯	◯
Actualmente, ¿con qué frecuencia tiene acceso a una mascarilla mientras trabaja fuera de su residencia?	◯	◯	◯	◯	◯
Actualmente, ¿con qué frecuencia lleva usted guantes desechables mientras trabaja fuera de su residencia?	◯	◯	◯	◯	◯
Actualmente, ¿con qué frecuencia lleva usted una mascarilla mientras trabaja fuera de su residencia?	◯	◯	◯	◯	◯
Actualmente, ¿con qué frecuencia se lava las manos con agua y jabón mientras trabaja fuera de su residencia?	◯	◯	◯	◯	◯
Actualmente, ¿con qué frecuencia se desinfecta las manos con desinfectante de manos mientras trabaja fuera de su residencia?	◯	◯	◯	◯	◯
Actualmente, ¿cuánto le preocupa exponerse a la COVID-19 mientras trabaja fuera de su residencia?	◯	◯	◯	◯	◯

¿Trabaja usted actualmente en alguno de los siguientes ambientes de alto riesgo de contagio de COVID-19?

- ☐ Ambiente de asistencia médica (hospital, clínica, centro de urgencias)
- ☐ Ambiente residencial denso (hogar de ancianos, otro centro de asistencia de larga duración)
- ☐ Prisión o cárcel
- ☐ Establecimiento de envasado de carne
- ☐ Establecimiento de envío o distribución
- ☐ Establecimiento minorista de alto volumen (tienda de provisiones, etc.)

¿Cuánto mide usted?

____
((Dé su estatura en pies y pulgadas (p. ej.,5'10)))

¿Cuánto pesa usted?

____
((Dé su peso en libras (p. ej.,145)))

¿Tiene usted seguro médico o de salud (seguro privado, seguro que usted ha comprado, Medicare, Medicaid o algún otro tipo de seguro)?

- ◯ Sí
- ◯ No
- ◯ No sabe

¿Cuál es su principal seguro de salud?

- ◯ Seguro médico privado por empleo o establecimiento educacional
- ◯ Seguro comprado en un intercambio de seguros de salud estatal o federal, tal como healthcare.gov
- ◯ Seguro comprado directamente a una campañia de seguros
- ◯ Medicare
- ◯ Medi-Gap
- ◯ Medicaid
- ◯ Asistencia médica militar (TRICARE, VA, CHAMP-VA, etc.)
- ◯ Servicio de Salud Indígena
- ◯ Otro

(Seleccione uno (su seguro principal).)

Si marcó "Otro", especifique la fuente de seguro médico.

____

Alguna vez le han dado un diagnostico de alguno de los siguientes?

	Sí	No
Alergias estacionales	◯	◯
Asma	◯	◯
Diabetes	◯	◯
Hipertensión	◯	◯
Enfermedad cardiovascular (ataque al corazón, insuficiencia cardíaca, angina, etc.)	◯	◯
Cáncer	◯	◯
Enfermedad pulmonar o respiratoria crónica (COPD enfisema, bronquitis, etc.)	◯	◯
Enfermedad crónica de los riñones	◯	◯
Enfermedad crónica del hígado (cirrosis, etc.)	◯	◯

Debilitamiento del sistema inmunitario (VIH, tratamiento crónico con corticosteroides, trasplante de órganos, etc.)	◯	◯
Otra afección crónica	◯	◯

¿Qué otra afección crónica le han diagnosticado?

____

¿Está usted actualmente embarazada?
- ◯ Sí
- ◯ No

¿Le han diagnosticado COVID-19?
- ◯ Sí
- ◯ No

¿Cuándo le diagnosticaron COVID-19?

____
(Si le hicieron la prueba, dé la fecha exacta o más aproximada posible. Si no le hicieron la prueba, dé la fecha exacta o más aproximada posible en que un médico le diagnosticó la COVID-19.)

	Excelente	Muy buena	Buena	Regular	Mala
En general, ¿cómo habría calificado usted su salud antes de la pandemia de COVID-19?	◯	◯	◯	◯	◯
En general, ¿cómo calificaría usted su salud en las últimas dos semanas?	◯	◯	◯	◯	◯

¿Ha sido usted vacunado contra la influenza esta temporada (2019-2020)?
- ◯ Sí
- ◯ No
- ◯ No sabe

¿Con qué frecuencia se vacuna usted contra la influenza?
- ◯ Cada temporada de influenza
- ◯ La mayoria de las temporadas de influenza
- ◯ La mitad de las temporadas de influenza
- ◯ Menos de la mitad de las temporadas de influenza
- ◯ Nunca

Con que frequencia ha hecho lo siguiente desde el inicio de la pandemia de COVID-19 en Carolina del Norte para protegerse contra la infeccion?

	Siempre (100%)	La mayor parte del tiempo (75%)	La mitad del tiempo (50%)	Menos de la mitad del tiempo (25%)	Nunca (0%)
Llevar mascarilla	◯	◯	◯	◯	◯

Lavarse las manos y/o limpiárselas con desinfectante a menudo	○	○	○	○	○
Mantenerse al menos a 6 pies de distancia de los demas	○	○	○	○	○
Evitar grandes reuniones, espacios públicos o multitudes	○	○	○	○	○
Evitar el contacto con personas de alto riesgo	○	○	○	○	○
No comer en restaurante, ni siquiera comprar comida para llevar	○	○	○	○	○
Trabajar o estudiar en casa en lugar de ir a una oficina o un aula	○	○	○	○	○
No dar la mano ni tocar a otras personas.	○	○	○	○	○
Quedarse en casa cuando se está enfermo	○	○	○	○	○
Limpiar las superficies con desinfectante	○	○	○	○	○
Cancelar o postergar los viajes de trabajo	○	○	○	○	○
Cancelar o postergar los viajes de placer	○	○	○	○	○
Cancelar o postergar las actividades personales o sociales	○	○	○	○	○
Cancelar las consultas médicas en persona	○	○	○	○	○
Almacenar comida o agua	○	○	○	○	○
Seguir las recomendaciones o reglas del gobierno sobre refugiarse donde se esté (es decir, quedarse en casa, limitar el contacto con otras personas, etc.)	○	○	○	○	○

¿Ha recibido una vacuna de COVID-19 fuera de un ensayo clínico?

○ Sí
○ No

¿Dónde recibió la vacuna de COVID-19?

○ Oficina del doctor/medico
○ Trabajo/Empleo
○ Minorista (por ejemplo Walgreens, CVS)
○ Sitio de la vacuna
○ Otro

Especifique donde recibió la vacuna de COVID-19.

____

Especifique la cuidad/el pueblo en Carolina del Norte del sitio de la vacuna:	__________________________________
Especifique quien fue la organización o instituto que organizo el sitio de la vacuna:	__________________________________
¿Cuál vacuna de COVID-19 recibió?	◯ Pfizer<br>◯ Moderna<br>◯ AstraZeneca<br>◯ Novavax<br>◯ Johnson & Johnson<br>◯ Otro<br>◯ No sabe
Especifique que otra vacuna de COVID recibió.	__________________________________
¿Cuántas dosis de la vacuna ha recibido?	◯ 1<br>◯ 2
¿Qué día recibió la primera dosis de la vacuna?	__________________________________
¿Qué día recibió la segunda dosis de la vacuna?	__________________________________

¿Usted sintió alguno de los siguientes efectos secundarios después de la vacunación?

		No	Leve (nota síntomas, pero no son un problema)	Moderado (los síntomas limitan sus actividades diarias normales)	Grave (los síntomas hacen que las actividades normales sean difícil o imposible)
1	dolor en o alrededor del lugar de la inyección	◯	◯	◯	◯
2	enrojecimiento en o alrededor del lugar de la inyección	◯	◯	◯	◯
3	hinchazón en o alrededor del lugar de la inyección	◯	◯	◯	◯
4	erupción en o alrededor del lugar de la inyección	◯	◯	◯	◯
5	dolor de cabeza	◯	◯	◯	◯
6	fatiga/cansancio	◯	◯	◯	◯
7	fiebre (temperatura >100.4°F o >38°C)	◯	◯	◯	◯
8	escalofríos	◯	◯	◯	◯
9	dolor en las articulaciones	◯	◯	◯	◯
10	dolor muscular	◯	◯	◯	◯
11					

nausea ◯ ◯ ◯ ◯

¿Cuánto tiempo duraron estos efectos secundarios?
- ◯ menos de 12 horas
- ◯ 12 a 24 horas
- ◯ más de 24 horas

¿Tomo algún medicamento para estos efectos secundarios?
- ◯ Sí
- ◯ No

¿Que medicamento(s) tomo para los efectos secundarios?

____
(Por favor enumere todos los medicamentos.)

¿Consultó a un médico u otro proveedor de atención médica para los efectos secundarios?
- ◯ Sí
- ◯ No

¿Como experimento los efectos secundarios después de la secunda dosis de la vacuna en comparación con los efectos secundarios después de la primera dosis de la vacuna?
- ◯ más grave
- ◯ menos grave
- ◯ igualmente grave
- ◯ no corresponde/aún no he recibido la segunda dosis

¿Piensa usted vacunarse contra la COVID-19 si se ofrece una vacuna?
- ◯ Sí
- ◯ No
- ◯ No sabe

Cuáles de las siguientes, si alguna, son razones por las que respondió "no" o "no se" acerca de recibir la vacuna de COVID-19?
- ☐ Me preocupan los efectos secundarios y la seguridad de la vacuna de COVID-19
- ☐ Tengo una condición subyacente y no hay suficientes investigaciones que me hagan sentir cómodo al recibir la vacuna de COVID-19 en este momento
- ☐ Me preocupa que la vacuna de COVID-19 se esté desarrollando demasiado rápido
- ☐ Tengo la intención de esperar y ver si la vacuna es segura y recibirlo más adelante
- ☐ No confió en el gobierno
- ☐ Tengo la intención de usar máscaras y otras precauciones en vez de recibir la vacuna
- ☐ No soy miembro de ningún grupo que este en alto riesgo de COVID-19
- ☐ No creo que COVID-19 sea una enfermedad grave
- ☐ Creo que la vacuna podría darme COVID-19
- ☐ No creo que la vacuna de COVID-19 funcione
- ☐ No me gustan las agujas
- ☐ Ya tuve COVID-19 y creo que debería ser inmune
- ☐ Mi doctor no me ha recomendado la vacuna de COVID-19
- ☐ No sabía que necesitaba una vacuna contra COVID-19
- ☐ Me preocupan los costos asociados con la vacuna (como los costos de las visitas al consultorio o las tarifas de administración de la vacuna)
- ☐ Todavía no soy elegible (según las pautas de las fases en NC o debido a otras condiciones/procedimientos de salud)
- ☐ No tengo acceso a la vacuna
- ☐ Otra cosa

(Marque todo lo que corresponda)

Especifique que otro motivo tiene usted para o no recibir la vacuna de COVID-19

____

Durante los ultimos dos semanas, ha tenido alguno de los siguientes sintomas?

	Sí	No
Fiebre (medida por termómetro o autodiagnóstico)	○	○
Tos (nueva o que empeora)	○	○
Dificultad para respirar (nueva o que empeora)	○	○
Fatiga (nuevo cansancio al hacer actividades normales)	○	○
Dolores de cuerpo	○	○
Dolor de cabeza	○	○
Diarrea	○	○
Dolor de garganta	○	○
Picazón, enrojecimiento o dolor de ojos	○	○
Goteo o congestión nasal	○	○
Cambios en el sentido del olfato o del gusto	○	○
Nuevo sarpullido	○	○
Temblores y escalofríos reiterados	○	○

¿Cuándo comenzaron estos síntomas?

____

En vista de los síntomas que declaró, ¿le preocupaba a usted la posibilidad de tener COVID-19?

○ Sí
○ No
○ No sabe

¿Fue usted objeto de prejuicio o discriminación debido a sus síntomas?

○ Sí
○ No
○ No sabe

¿Cuáles de las siguientes medidas tomó usted para proteger a sus amigos y familiares después de que comenzaron sus síntomas?

☐ Ponerse mascarilla con mayor frecuencia
☐ Lavarse las manos con agua y jabón con mayor frecuencia
☐ Limpiarse las manos con desinfectante con mayor frecuencia
☐ Aislarse en su casa con mayor frecuencia
☐ Quedarse en su casa con mayor frecuencia
☐ Ponerse guantes desechables con mayor frecuencia

¿Qué hizo usted en vista de sus síntomas?	☐ Nada<br>☐ Tomó medicamentos sin receta (ibuprofeno, acetaminofén, etc.)<br>☐ Consultó por teléfono a un proveedor de asistencia médica<br>☐ Fue al consultorio de un proveedor de atencion médica<br>☐ Fue a una clínica o una farmacia minorista<br>☐ Fue a un centro de urgencias (FASTMed, etc.)<br>☐ Fue a la sala de emergencias<br>☐ Fue ingresado al hospital<br>☐ Otra cosa<br>☐ No sabe<br>(Seleccione todo lo que corresponda.)
¿Qué otra cosa hizo usted en vista de sus síntomas?	__________________________________
Si pudo hablar con un proveedor de asistencia médica, ¿le dijo el proveedor que era posible que tuviera COVID-19?	◯ Sí<br>◯ No<br>◯ No sabe
Si le hicieron una prueba de COVID-19 en vista de los síntomas que declaró, ¿cuál fue el resultado?	◯ Pendiente<br>◯ Positivo<br>◯ Negativo<br>◯ No concluyente<br>◯ No se hizo la prueba
¿Cuántos días estuvo usted hospitalizado?	__________________________________
¿Le hicieron a usted las siguientes intervenciones durante su hospitalización?	☐ Oxígeno adicional por la nariz<br>☐ Tratamiento en la Unidad de Cuidados Intensivos (Intensive Care Unit, ICU)<br>☐ Ventilación mecánica (intubación o tubo de respiración)
¿Ha vuelto usted a su salud normal?	◯ Sí<br>◯ No<br>◯ No sabe

Proporcione la siguiente informacion sobre su hogar.

¿Cuál es su dirección permanente?	__________________________________
¿Cuanto tiempo ha vivido en esta dirección?	◯ 0-3 años<br>◯ 4-6 años<br>◯ 7-10 años<br>◯ Más de 10 años

¿Cuántas personas (sin contarse usted) viven o pasan una cantidad considerable de tiempo en esta vivienda (más de 40 horas por semana)?	◯ 0<br>◯ 1<br>◯ 2<br>◯ 3<br>◯ 4<br>◯ 5<br>◯ 6<br>◯ 7<br>◯ 8<br>◯ 9<br>◯ 10<br>◯ 11<br>◯ 12
¿Cuántas personas de su grupo familiar son menores de 18 años?	◯ 0<br>◯ 1<br>◯ 2<br>◯ 3<br>◯ 4<br>◯ 5<br>◯ 6<br>◯ 7<br>◯ 8<br>◯ 9<br>◯ 10<br>◯ 11<br>◯ 12
¿Qué tan difícil ha sido para su grupo familiar adaptarse a los cambios en el cuidado infantil o la educación de los niños en casa?	◯ Nada dificil<br>◯ Un poco dificil<br>◯ Muy dificil<br>◯ Extremadamente dificil
¿Cuál es el idioma principal que se habla en su grupo familiar?	◯ Inglés<br>◯ Español<br>◯ Otro
¿Qué otro idioma principal se habla en su grupo familiar?	__________________________________
Antes de la pandemia de COVID-19, ¿tenía usted responsabilidades habituales de cuidado de una persona que no vivía con su grupo familiar (por ejemplo, padre o hermano de edad a quien usted visitaba y apoyaba con regularidad, etc.)?	◯ Sí<br>◯ No
¿Qué tan difícil ha sido continuar con sus responsabilidades de cuidado debido a la pandemia de COVID-19?	◯ Nada dificil<br>◯ Un poco dificil<br>◯ Muy dificil<br>◯ Extremadamente dificil
Si a un integrante del grupo familiar le da COVID-19, ¿qué tan bien podría el grupo familiar aislarlo de otros integrantes del grupo familiar (asignarle su propia habitación y limitar el contacto con el enfermo)?	◯ No muy bien<br>◯ Bastante bien<br>◯ Muy bien<br>◯ Extremadamente bien

Antes de descontar los impuestos, ¿cuál fue el monto total aproximado de todas las fuentes de ingresos del grupo familiar el año pasado?

- ◯ Menos que $10,000
- ◯ $10,000-$19,999
- ◯ $20,000-$29,999
- ◯ $30,000-$49,999
- ◯ $50,000-$74,999
- ◯ $75,000 o más

En su opinión, ¿qué efecto va a tener este año la crisis de COVID-19 en el monto total de ingresos de su grupo familiar?

- ◯ Disminución considerable
- ◯ Disminución ligera
- ◯ Ningún efecto
- ◯ Aumento considerable
- ◯ Aumento ligero

En su opinión ¿qué tan probable es que su grupo familiar se quede sin dinero en los próximos 3 meses? Dé su respuesta en una escala de 0 (definitivamente no va a suceder) a 10 (definitivamente va a suceder).

____

¿Con qué frecuencia recibe usted o su grupo familiar asistencia para hacer mandados tales como comprar provisiones o medicamentos?

- ◯ Todo el tiempo (100%)
- ◯ La mayor parte del tiempo (75%)
- ◯ La mitad del tiempo (50%)
- ◯ Menos de la mitad del tiempo (25%)
- ◯ Nunca (0%)

¿Con qué frecuencia se ha quedado usted o su familia en su casa y ha evitado interactuar con otras personas, exceptuando las salidas para comprar provisiones?

- ◯ Todo el tiempo (100%)
- ◯ La mayor parte del tiempo (75%)
- ◯ La mitad del tiempo (50%)
- ◯ Menos de la mitad del tiempo (25%)
- ◯ Nunca (0%)

Por cada persona adicional en su hogar, proporcione la siguiente informacion.

Persona 1: ¿Cuál es su relación con esta persona?

- ◯ Pareja o cónyuge
- ◯ Hijo o hija
- ◯ Padre o madre
- ◯ Hermano o hermana
- ◯ Otra familiar
- ◯ Proveedor de cuidado infantil u otros cuidados en casa
- ◯ Otra

Persona 1: Especifique su relación con esta persona.

____

Persona 1: ¿Qué edad tiene esta persona?

____
((Especifique la edad en años))

Persona 1: ¿Cuál es el sexo de esta persona?

- ◯ Femenino
- ◯ Masculino

Persona 1: ¿Cuál es la raza de esta persona?	☐ Indio americano o nativo de Alaska<br>☐ Asiático<br>☐ Negro o afroestadounidense<br>☐ Nativo de Hawái o de las islas del Pacífico<br>☐ Blanco<br>☐ Otro<br>☐ No sabe<br>((Seleccione todo lo que corresponda.))
Persona 1: ¿Cuál es la identidad étnica de esta persona?	○ Hispano o latino<br>○ Ni hispano ni latino<br>○ Otra<br>○ No sabe
Persona 1: ¿Cuál es el nivel de educación de esta persona?	○ No tiene educación formal<br>○ Kínder a 8° grado<br>○ Estudios de educación secundaria<br>○ Equivalencia de educación secundaria (GED)<br>○ Diploma de educación secundaria<br>○ Estudios de educación superior<br>○ Título universitario<br>○ Estudios de posgrado o más<br>○ No sabe
Persona 1: ¿Cuál de las siguientes es la mejor descripción de la situación laboral actual de esta persona?	○ Empleo a tiempo completo<br>○ Empleo a tiempo parcial<br>○ En busca de empleo<br>○ Jubilado<br>○ Ama de casa<br>○ Estudiante<br>○ Permiso de maternidad o paternidad<br>○ Permiso por enfermedad<br>○ Desempleado por discapacidad<br>○ Otra<br>○ No sabe
Persona 1: ¿Se considera esta persona actualmente empleado por cuenta propia (contratista independiente, trabajador esporádico (gig), etc.)?	○ Sí<br>○ No<br>○ No sabe
Persona 1: ¿Trabaja esta persona actualmente en alguno de los siguientes ambientes de alto riesgo de contagio de COVID-19?	☐ Ambiente de asistencia médica (hospital, clínica, centro de urgencias, etc.)<br>☐ Ambiente residencial denso (hogar de ancianos, otro centro de asistencia de larga duración)<br>☐ Prisión o cárcel<br>☐ Establecimiento de envasado de carne<br>☐ Establecimiento de envío o distribución<br>☐ Establecimiento minorista de alto volumen (tienda de provisiones, etc.)<br>☐ No sabe
Persona 1: ¿Le ofrece el empleador a esta persona alguno de los siguientes beneficios en su empleo principal actual?	☐ Permiso por enfermedad con goce de sueldo<br>☐ Vacaciones o permiso personal con goce de sueldo<br>☐ Seguro de salud<br>☐ Seguro de discapacidad<br>☐ Plan de jubilación<br>☐ Otro<br>☐ No sabe<br>((Seleccione todo lo que corresponda.))

Persona 1: En una escala de 0 (definitivamente no va a suceder) a 10 (definitivamente va a suceder), ¿qué tan probable es que esta persona pierda su empleo debido a la pandemia de COVID-19? ________________________________

Persona 1: En una escala de 0 (definitivamente no va a suceder) a 10 (definitivamente va a suceder), ¿qué tan probable es que a esta persona se le asignen menos horas de trabajo debido a la pandemia de COVID-19? ________________________________

	Todo el tiempo (100%)	la mayor parte del tiempo (75%)	La mitad del tiempo (50%)	Menos de la mitad del tiempo (25%)	Nunca (0%)
Persona 1: Actualmente ¿con qué frecuencia se le exige a esta persona que trabaje fuera de su residencia?	◯	◯	◯	◯	◯
Persona 1: Actualmente ¿con qué frecuencia se encuentra esta persona físicamente cerca de sus compañeros de trabajo mientras trabaja fuera de su residencia?	◯	◯	◯	◯	◯
Persona 1: Actualmente ¿con qué frecuencia se encuentra esta persona físicamente cerca de sus clientes mientras trabaja fuera de su residencia?	◯	◯	◯	◯	◯

Persona 1: ¿Piensa esta persona vacunarse contra la COVID-19 cuando se ofrezca una vacuna?

◯ Sí
◯ No
◯ No sabe

Persona 1: En las últimas dos semanas, ¿ha tenido esta persona algún síntoma de COVID-19 (tos, fiebre, dificultad para respirar, fatiga, dolores de cuerpo, diarrea, goteo nasal o pérdida del sentido del olfato o del gusto)?

◯ Sí
◯ No
◯ No sabe

Persona 1: ¿Cuándo le comenzaron los síntomas de COVID-19 a esta persona? ________________________________

Persona 1: En vista de sus síntomas, ¿le preocupa a esta persona la posibilidad de tener COVID-19?

◯ Sí
◯ No
◯ No sabe

Persona 1: ¿Fue esta persona objeto de prejuicio o discriminación debido a sus síntomas?

◯ Sí
◯ No
◯ No sabe

Persona 1: ¿Qué hizo esta persona en vista de sus síntomas?	☐ Nada<br>☐ Tomó medicamentos sin receta (ibuprofeno, acetaminofén, etc.)<br>☐ Consultó por teléfono a un proveedor de asistencia médica<br>☐ Fue al consultorio de un proveedor de asistencia médica<br>☐ Fue a una clínica o una farmacia minorista<br>☐ Fue a un centro de urgencias (FASTMed, etc.)<br>☐ Fue a la sala de emergencias<br>☐ Fue ingresado al hospital<br>☐ Otra cosa<br>☐ No sabe<br>((Seleccione todo lo que corresponda.))
Persona 1: ¿Qué otra cosa hizo esta persona en vista de sus síntomas?	__________________________________
Persona 1: ¿Le dijo un proveedor de asistencia médica a esta persona que era posible que tuviera COVID-19?	◯ Sí<br>◯ No<br>◯ No sabe
Persona 1: Si a esta persona le hicieron una prueba de COVID-19 en vista de sus síntomas, ¿cuál fue el resultado?	◯ Pendiente<br>◯ Positivo<br>◯ Negativo<br>◯ No concluyente<br>◯ No se hizo la prueba<br>◯ No sabe
Persona 1: ¿Cuántos días estuvo hospitalizada esta persona?	__________________________________
Persona 1: ¿Se le hicieron a esta persona las siguientes intervenciones durante su hospitalización?	☐ Oxígeno adicional por la nariz<br>☐ Tratamiento en la Unidad de Cuidados Intensivos (Intensive Care Unit, ICU)<br>☐ Ventilación mecánica (intubación o tubo de respiración)<br>☐ No sabe
Persona 1: ¿Ha vuelto esta persona a su salud normal?	◯ Sí<br>◯ No<br>◯ No sabe
Persona 1: ¿Cuáles de las siguientes medidas tomó esta persona para proteger a sus amigos y familiares después de que comenzaron sus síntomas?	☐ Ponerse mascarilla con mayor frecuencia<br>☐ Lavarse las manos con agua y jabón con mayor frecuencia<br>☐ Limpiarse las manos con desinfectante con mayor frecuencia<br>☐ Aislarse en su casa con mayor frecuencia<br>☐ Quedarse en casa con mayor frecuencia<br>☐ Ponerse guantes desechables con mayor frecuencia<br>☐ No sabe

Por cada persona adicional en su hogar, proporcione la siguiente informacion.

Persona 2: ¿Cuál es su relación con esta persona?	◯ Pareja o cónyuge<br>◯ Hijo o hija<br>◯ Padre o madre<br>◯ Hermano o hermana<br>◯ Otra familiar<br>◯ Proveedor de cuidado infantil u otros cuidados en casa<br>◯ Otra
Persona 2: Especifique su relación con esta persona.	__________________________________
Persona 2: ¿Qué edad tiene esta persona?	__________________________________<br>((Especifique la edad en años))
Persona 2: ¿Cuál es el sexo de esta persona?	◯ Femenino<br>◯ Masculino
Persona 2: ¿Cuál es la raza de esta persona?	☐ Indio americano o nativo de Alaska<br>☐ Asiático<br>☐ Negro o afroestadounidense<br>☐ Nativo de Hawái o de las islas del Pacífico<br>☐ Blanco<br>☐ Otro<br>☐ No sabe<br>((Seleccione todo lo que corresponda.))
Persona 2: ¿Cuál es la identidad étnica de esta persona?	◯ Hispano o latino<br>◯ Ni hispano ni latino<br>◯ Otra<br>◯ No sabe
Persona 2: ¿Cuál es el nivel de educación de esta persona?	◯ No tiene educación formal<br>◯ Kínder a 8° grado<br>◯ Estudios de educación secundaria<br>◯ Equivalencia de educación secundaria (GED)<br>◯ Diploma de educación secundaria<br>◯ Estudios de educación superior<br>◯ Título universitario<br>◯ Estudios de posgrado o más<br>◯ No sabe
Persona 2: ¿Cuál de las siguientes es la mejor descripción de la situación laboral actual de esta persona?	◯ Empleo a tiempo completo<br>◯ Empleo a tiempo parcial<br>◯ En busca de empleo<br>◯ Jubilado<br>◯ Ama de casa<br>◯ Estudiante<br>◯ Permiso de maternidad o paternidad<br>◯ Permiso por enfermedad<br>◯ Desempleado por discapacidad<br>◯ Otra<br>◯ No sabe
Persona 2: ¿Se considera esta persona actualmente empleado por cuenta propia (contratista independiente, trabajador esporádico (gig), etc.)?	◯ Sí<br>◯ No<br>◯ No sabe

Persona 2: ¿Trabaja esta persona actualmente en alguno de los siguientes ambientes de alto riesgo de contagio de COVID-19?	☐ Ambiente de asistencia médica (hospital, clínica, centro de urgencias, etc.)<br>☐ Ambiente residencial denso (hogar de ancianos, otro centro de asistencia de larga duración)<br>☐ Prisión o cárcel<br>☐ Establecimiento de envasado de carne<br>☐ Establecimiento de envío o distribución<br>☐ Establecimiento minorista de alto volumen (tienda de provisiones, etc.)<br>☐ No sabe
Persona 2: ¿Le ofrece el empleador a esta persona alguno de los siguientes beneficios en su empleo principal actual?	☐ Permiso por enfermedad con goce de sueldo<br>☐ Vacaciones o permiso personal con goce de sueldo<br>☐ Seguro de salud<br>☐ Seguro de discapacidad<br>☐ Plan de jubilación<br>☐ Otro<br>☐ No sabe<br>((Seleccione todo lo que corresponda.))
Persona 2: En una escala de 0 (definitivamente no va a suceder) a 10 (definitivamente va a suceder), ¿qué tan probable es que esta persona pierda su empleo debido a la pandemia de COVID-19?	______________________
Persona 2: En una escala de 0 (definitivamente no va a suceder) a 10 (definitivamente va a suceder), ¿qué tan probable es que a esta persona se le asignen menos horas de trabajo debido a la pandemia de COVID-19?	______________________

	Todo el tiempo (100%)	la mayor parte del tiempo (75%)	La mitad del tiempo (50%)	Menos de la mitad del tiempo (25%)	Nunca (0%)
Persona 2: Actualmente ¿con qué frecuencia se le exige a esta persona que trabaje fuera de su residencia?	○	○	○	○	○
Persona 2: Actualmente ¿con qué frecuencia se encuentra esta persona físicamente cerca de sus compañeros de trabajo mientras trabaja fuera de su residencia?	○	○	○	○	○
Persona 2: Actualmente ¿con qué frecuencia se encuentra esta persona físicamente cerca de sus clientes mientras trabaja fuera de su residencia?	○	○	○	○	○

Persona 2: ¿Piensa esta persona vacunarse contra la COVID-19 cuando se ofrezca una vacuna?	○ Sí<br>○ No<br>○ No sabe

Persona 2: En las últimas dos semanas, ¿ha tenido esta persona algún síntoma de COVID-19 (tos, fiebre, dificultad para respirar, fatiga, dolores de cuerpo, diarrea, goteo nasal o pérdida del sentido del olfato o del gusto)?	◯ Sí<br>◯ No<br>◯ No sabe
Persona 2: ¿Cuándo le comenzaron los síntomas de COVID-19 a esta persona?	__________________________
Persona 2: En vista de sus síntomas, ¿le preocupa a esta persona la posibilidad de tener COVID-19?	◯ Sí<br>◯ No<br>◯ No sabe
Persona 2: ¿Fue esta persona objeto de prejuicio o discriminación debido a sus síntomas?	◯ Sí<br>◯ No<br>◯ No sabe
Persona 2: ¿Qué hizo esta persona en vista de sus síntomas?	☐ Nada<br>☐ Tomó medicamentos sin receta (ibuprofeno, acetaminofén, etc.)<br>☐ Consultó por teléfono a un proveedor de asistencia médica<br>☐ Fue al consultorio de un proveedor de asistencia médica<br>☐ Fue a una clínica o una farmacia minorista<br>☐ Fue a un centro de urgencias (FASTMed, etc.)<br>☐ Fue a la sala de emergencias<br>☐ Fue ingresado al hospital<br>☐ Otra cosa<br>☐ No sabe<br>((Seleccione todo lo que corresponda.))
Persona 2: ¿Qué otra cosa hizo esta persona en vista de sus síntomas?	__________________________
Persona 2: ¿Le dijo un proveedor de asistencia médica a esta persona que era posible que tuviera COVID-19?	◯ Sí<br>◯ No<br>◯ No sabe
Persona 2: Si a esta persona le hicieron una prueba de COVID-19 en vista de sus síntomas, ¿cuál fue el resultado?	◯ Pendiente<br>◯ Positivo<br>◯ Negativo<br>◯ No concluyente<br>◯ No se hizo la prueba<br>◯ No sabe
Persona 2: ¿Cuántos días estuvo hospitalizada esta persona?	__________________________
Persona 2: ¿Se le hicieron a esta persona las siguientes intervenciones durante su hospitalización?	☐ Oxígeno adicional por la nariz<br>☐ Tratamiento en la Unidad de Cuidados Intensivos (Intensive Care Unit, ICU)<br>☐ Ventilación mecánica (intubación o tubo de respiración)<br>☐ No sabe
Persona 2: ¿Ha vuelto esta persona a su salud normal?	◯ Sí<br>◯ No<br>◯ No sabe

Persona 2: ¿Cuáles de las siguientes medidas tomó esta persona para proteger a sus amigos y familiares después de que comenzaron sus síntomas?

- ☐ Ponerse mascarilla con mayor frecuencia
- ☐ Lavarse las manos con agua y jabón con mayor frecuencia
- ☐ Limpiarse las manos con desinfectante con mayor frecuencia
- ☐ Aislarse en su casa con mayor frecuencia
- ☐ Quedarse en casa con mayor frecuencia
- ☐ Ponerse guantes desechables con mayor frecuencia
- ☐ No sabe

Por cada persona adicional en su hogar, proporcione la siguiente informacion.

Persona 3: ¿Cuál es su relación con esta persona?

- ◯ Pareja o cónyuge
- ◯ Hijo o hija
- ◯ Padre o madre
- ◯ Hermano o hermana
- ◯ Otra familiar
- ◯ Proveedor de cuidado infantil u otros cuidados en casa
- ◯ Otra

Persona 3: Especifique su relación con esta persona.

____

Persona 3: ¿Qué edad tiene esta persona?

____
((Especifique la edad en años))

Persona 3: ¿Cuál es el sexo de esta persona?

- ◯ Femenino
- ◯ Masculino

Persona 3: ¿Cuál es la raza de esta persona?

- ☐ Indio americano o nativo de Alaska
- ☐ Asiático
- ☐ Negro o afroestadounidense
- ☐ Nativo de Hawái o de las islas del Pacífico
- ☐ Blanco
- ☐ Otro
- ☐ No sabe

((Seleccione todo lo que corresponda.))

Persona 3: ¿Cuál es la identidad étnica de esta persona?

- ◯ Hispano o latino
- ◯ Ni hispano ni latino
- ◯ Otra
- ◯ No sabe

Persona 3: ¿Cuál es el nivel de educación de esta persona?

- ◯ No tiene educación formal
- ◯ Kínder a 8° grado
- ◯ Estudios de educación secundaria
- ◯ Equivalencia de educación secundaria (GED)
- ◯ Diploma de educación secundaria
- ◯ Estudios de educación superior
- ◯ Título universitario
- ◯ Estudios de posgrado o más
- ◯ No sabe

Persona 3: ¿Cuál de las siguientes es la mejor descripción de la situación laboral actual de esta persona?	◯ Empleo a tiempo completo<br>◯ Empleo a tiempo parcial<br>◯ En busca de empleo<br>◯ Jubilado<br>◯ Ama de casa<br>◯ Estudiante<br>◯ Permiso de maternidad o paternidad<br>◯ Permiso por enfermedad<br>◯ Desempleado por discapacidad<br>◯ Otra<br>◯ No sabe
Persona 3: ¿Se considera esta persona actualmente empleado por cuenta propia (contratista independiente, trabajador esporádico (gig), etc.)?	◯ Sí<br>◯ No<br>◯ No sabe
Persona 3: ¿Trabaja esta persona actualmente en alguno de los siguientes ambientes de alto riesgo de contagio de COVID-19?	☐ Ambiente de asistencia médica (hospital, clínica, centro de urgencias, etc.)<br>☐ Ambiente residencial denso (hogar de ancianos, otro centro de asistencia de larga duración)<br>☐ Prisión o cárcel<br>☐ Establecimiento de envasado de carne<br>☐ Establecimiento de envío o distribución<br>☐ Establecimiento minorista de alto volumen (tienda de provisiones, etc.)<br>☐ No sabe
Persona 3: ¿Le ofrece el empleador a esta persona alguno de los siguientes beneficios en su empleo principal actual?	☐ Permiso por enfermedad con goce de sueldo<br>☐ Vacaciones o permiso personal con goce de sueldo<br>☐ Seguro de salud<br>☐ Seguro de discapacidad<br>☐ Plan de jubilación<br>☐ Otro<br>☐ No sabe<br>((Seleccione todo lo que corresponda.))
Persona 3: En una escala de 0 (definitivamente no va a suceder) a 10 (definitivamente va a suceder), ¿qué tan probable es que esta persona pierda su empleo debido a la pandemia de COVID-19?	______________________
Persona 3: En una escala de 0 (definitivamente no va a suceder) a 10 (definitivamente va a suceder), ¿qué tan probable es que a esta persona se le asignen menos horas de trabajo debido a la pandemia de COVID-19?	______________________

	Todo el tiempo (100%)	la mayor parte del tiempo (75%)	La mitad del tiempo (50%)	Menos de la mitad del tiempo (25%)	Nunca (0%)
Persona 3: Actualmente ¿con qué frecuencia se le exige a esta persona que trabaje fuera de su residencia?	◯	◯	◯	◯	◯

Persona 3: Actualmente ¿con qué frecuencia se encuentra esta persona físicamente cerca de sus compañeros de trabajo mientras trabaja fuera de su residencia? ◯ ◯ ◯ ◯ ◯

Persona 3: Actualmente ¿con qué frecuencia se encuentra esta persona físicamente cerca de sus clientes mientras trabaja fuera de su residencia? ◯ ◯ ◯ ◯ ◯

Persona 3: ¿Piensa esta persona vacunarse contra la COVID-19 cuando se ofrezca una vacuna?

◯ Sí
◯ No
◯ No sabe

Persona 3: En las últimas dos semanas, ¿ha tenido esta persona algún síntoma de COVID-19 (tos, fiebre, dificultad para respirar, fatiga, dolores de cuerpo, diarrea, goteo nasal o pérdida del sentido del olfato o del gusto)?

◯ Sí
◯ No
◯ No sabe

Persona 3: ¿Cuándo le comenzaron los síntomas de COVID-19 a esta persona?

____

Persona 3: En vista de sus síntomas, ¿le preocupa a esta persona la posibilidad de tener COVID-19?

◯ Sí
◯ No
◯ No sabe

Persona 3: ¿Fue esta persona objeto de prejuicio o discriminación debido a sus síntomas?

◯ Sí
◯ No
◯ No sabe

Persona 3: ¿Qué hizo esta persona en vista de sus síntomas?

☐ Nada
☐ Tomó medicamentos sin receta (ibuprofeno, acetaminofén, etc.)
☐ Consultó por teléfono a un proveedor de asistencia médica
☐ Fue al consultorio de un proveedor de asistencia médica
☐ Fue a una clínica o una farmacia minorista
☐ Fue a un centro de urgencias (FASTMed, etc.)
☐ Fue a la sala de emergencias
☐ Fue ingresado al hospital
☐ Otra cosa
☐ No sabe
((Seleccione todo lo que corresponda.))

Persona 3: ¿Qué otra cosa hizo esta persona en vista de sus síntomas?

____

Persona 3: ¿Le dijo un proveedor de asistencia médica a esta persona que era posible que tuviera COVID-19?

◯ Sí
◯ No
◯ No sabe

Persona 3: Si a esta persona le hicieron una prueba de COVID-19 en vista de sus síntomas, ¿cuál fue el resultado?	◯ Pendiente<br>◯ Positivo<br>◯ Negativo<br>◯ No concluyente<br>◯ No se hizo la prueba<br>◯ No sabe
Persona 3: ¿Cuántos días estuvo hospitalizada esta persona?	__________________
Persona 3: ¿Se le hicieron a esta persona las siguientes intervenciones durante su hospitalización?	☐ Oxígeno adicional por la nariz<br>☐ Tratamiento en la Unidad de Cuidados Intensivos (Intensive Care Unit, ICU)<br>☐ Ventilación mecánica (intubación o tubo de respiración)<br>☐ No sabe
Persona 3: ¿Ha vuelto esta persona a su salud normal?	◯ Sí<br>◯ No<br>◯ No sabe
Persona 3: ¿Cuáles de las siguientes medidas tomó esta persona para proteger a sus amigos y familiares después de que comenzaron sus síntomas?	☐ Ponerse mascarilla con mayor frecuencia<br>☐ Lavarse las manos con agua y jabón con mayor frecuencia<br>☐ Limpiarse las manos con desinfectante con mayor frecuencia<br>☐ Aislarse en su casa con mayor frecuencia<br>☐ Quedarse en casa con mayor frecuencia<br>☐ Ponerse guantes desechables con mayor frecuencia<br>☐ No sabe

Por cada persona adicional en su hogar, proporcione la siguiente informacion.

Persona 4: ¿Cuál es su relación con esta persona?	◯ Pareja o cónyuge<br>◯ Hijo o hija<br>◯ Padre o madre<br>◯ Hermano o hermana<br>◯ Otra familiar<br>◯ Proveedor de cuidado infantil u otros cuidados en casa<br>◯ Otra
Persona 4: Especifique su relación con esta persona.	__________________
Persona 4: ¿Qué edad tiene esta persona?	__________________<br>((Especifique la edad en años))
Persona 4: ¿Cuál es el sexo de esta persona?	◯ Femenino<br>◯ Masculino

Persona 4: ¿Cuál es la raza de esta persona?	☐ Indio americano o nativo de Alaska<br>☐ Asiático<br>☐ Negro o afroestadounidense<br>☐ Nativo de Hawái o de las islas del Pacífico<br>☐ Blanco<br>☐ Otro<br>☐ No sabe<br>((Seleccione todo lo que corresponda.))
Persona 4: ¿Cuál es la identidad étnica de esta persona?	◯ Hispano o latino<br>◯ Ni hispano ni latino<br>◯ Otra<br>◯ No sabe
Persona 4: ¿Cuál es el nivel de educación de esta persona?	◯ No tiene educación formal<br>◯ Kínder a 8° grado<br>◯ Estudios de educación secundaria<br>◯ Equivalencia de educación secundaria (GED)<br>◯ Diploma de educación secundaria<br>◯ Estudios de educación superior<br>◯ Título universitario<br>◯ Estudios de posgrado o más<br>◯ No sabe
Persona 4: ¿Cuál de las siguientes es la mejor descripción de la situación laboral actual de esta persona?	◯ Empleo a tiempo completo<br>◯ Empleo a tiempo parcial<br>◯ En busca de empleo<br>◯ Jubilado<br>◯ Ama de casa<br>◯ Estudiante<br>◯ Permiso de maternidad o paternidad<br>◯ Permiso por enfermedad<br>◯ Desempleado por discapacidad<br>◯ Otra<br>◯ No sabe
Persona 4: ¿Se considera esta persona actualmente empleado por cuenta propia (contratista independiente, trabajador esporádico (gig), etc.)?	◯ Sí<br>◯ No<br>◯ No sabe
Persona 4: ¿Trabaja esta persona actualmente en alguno de los siguientes ambientes de alto riesgo de contagio de COVID-19?	☐ Ambiente de asistencia médica (hospital, clínica, centro de urgencias, etc.)<br>☐ Ambiente residencial denso (hogar de ancianos, otro centro de asistencia de larga duración)<br>☐ Prisión o cárcel<br>☐ Establecimiento de envasado de carne<br>☐ Establecimiento de envío o distribución<br>☐ Establecimiento minorista de alto volumen (tienda de provisiones, etc.)<br>☐ No sabe
Persona 4: ¿Le ofrece el empleador a esta persona alguno de los siguientes beneficios en su empleo principal actual?	☐ Permiso por enfermedad con goce de sueldo<br>☐ Vacaciones o permiso personal con goce de sueldo<br>☐ Seguro de salud<br>☐ Seguro de discapacidad<br>☐ Plan de jubilación<br>☐ Otro<br>☐ No sabe<br>((Seleccione todo lo que corresponda.))

Persona 4: En una escala de 0 (definitivamente no va a suceder) a 10 (definitivamente va a suceder), ¿qué tan probable es que esta persona pierda su empleo debido a la pandemia de COVID-19? ________________________

Persona 4: En una escala de 0 (definitivamente no va a suceder) a 10 (definitivamente va a suceder), ¿qué tan probable es que a esta persona se le asignen menos horas de trabajo debido a la pandemia de COVID-19? ________________________

	Todo el tiempo (100%)	la mayor parte del tiempo (75%)	La mitad del tiempo (50%)	Menos de la mitad del tiempo (25%)	Nunca (0%)
Persona 4: Actualmente ¿con qué frecuencia se le exige a esta persona que trabaje fuera de su residencia?	○	○	○	○	○
Persona 4: Actualmente ¿con qué frecuencia se encuentra esta persona físicamente cerca de sus compañeros de trabajo mientras trabaja fuera de su residencia?	○	○	○	○	○
Persona 4: Actualmente ¿con qué frecuencia se encuentra esta persona físicamente cerca de sus clientes mientras trabaja fuera de su residencia?	○	○	○	○	○

Persona 4: ¿Piensa esta persona vacunarse contra la COVID-19 cuando se ofrezca una vacuna?
- ○ Sí
- ○ No
- ○ No sabe

Persona 4: En las últimas dos semanas, ¿ha tenido esta persona algún síntoma de COVID-19 (tos, fiebre, dificultad para respirar, fatiga, dolores de cuerpo, diarrea, goteo nasal o pérdida del sentido del olfato o del gusto)?
- ○ Sí
- ○ No
- ○ No sabe

Persona 4: ¿Cuándo le comenzaron los síntomas de COVID-19 a esta persona? ________________________

Persona 4: En vista de sus síntomas, ¿le preocupa a esta persona la posibilidad de tener COVID-19?
- ○ Sí
- ○ No
- ○ No sabe

Persona 4: ¿Fue esta persona objeto de prejuicio o discriminación debido a sus síntomas?
- ○ Sí
- ○ No
- ○ No sabe

Persona 4: ¿Qué hizo esta persona en vista de sus síntomas?	☐ Nada<br>☐ Tomó medicamentos sin receta (ibuprofeno, acetaminofén, etc.)<br>☐ Consultó por teléfono a un proveedor de asistencia médica<br>☐ Fue al consultorio de un proveedor de asistencia médica<br>☐ Fue a una clínica o una farmacia minorista<br>☐ Fue a un centro de urgencias (FASTMed, etc.)<br>☐ Fue a la sala de emergencias<br>☐ Fue ingresado al hospital<br>☐ Otra cosa<br>☐ No sabe<br>((Seleccione todo lo que corresponda.))
Persona 4: ¿Qué otra cosa hizo esta persona en vista de sus síntomas?	______________________________
Persona 4: ¿Le dijo un proveedor de asistencia médica a esta persona que era posible que tuviera COVID-19?	◯ Sí<br>◯ No<br>◯ No sabe
Persona 4: Si a esta persona le hicieron una prueba de COVID-19 en vista de sus síntomas, ¿cuál fue el resultado?	◯ Pendiente<br>◯ Positivo<br>◯ Negativo<br>◯ No concluyente<br>◯ No se hizo la prueba<br>◯ No sabe
Persona 4: ¿Cuántos días estuvo hospitalizada esta persona?	______________________________
Persona 4: ¿Se le hicieron a esta persona las siguientes intervenciones durante su hospitalización?	☐ Oxígeno adicional por la nariz<br>☐ Tratamiento en la Unidad de Cuidados Intensivos (Intensive Care Unit, ICU)<br>☐ Ventilación mecánica (intubación o tubo de respiración)<br>☐ No sabe
Persona 4: ¿Ha vuelto esta persona a su salud normal?	◯ Sí<br>◯ No<br>◯ No sabe
Persona 4: ¿Cuáles de las siguientes medidas tomó esta persona para proteger a sus amigos y familiares después de que comenzaron sus síntomas?	☐ Ponerse mascarilla con mayor frecuencia<br>☐ Lavarse las manos con agua y jabón con mayor frecuencia<br>☐ Limpiarse las manos con desinfectante con mayor frecuencia<br>☐ Aislarse en su casa con mayor frecuencia<br>☐ Quedarse en casa con mayor frecuencia<br>☐ Ponerse guantes desechables con mayor frecuencia<br>☐ No sabe

Por cada persona adicional en su hogar, proporcione la siguiente informacion.

Persona 5: ¿Cuál es su relación con esta persona?	◯ Pareja o cónyuge<br>◯ Hijo o hija<br>◯ Padre o madre<br>◯ Hermano o hermana<br>◯ Otra familiar<br>◯ Proveedor de cuidado infantil u otros cuidados en casa<br>◯ Otra
Persona 5: Especifique su relación con esta persona.	__________________________
Persona 5: ¿Qué edad tiene esta persona?	__________________________<br>((Especifique la edad en años))
Persona 5: ¿Cuál es el sexo de esta persona?	◯ Femenino<br>◯ Masculino
Persona 5: ¿Cuál es la raza de esta persona?	☐ Indio americano o nativo de Alaska<br>☐ Asiático<br>☐ Negro o afroestadounidense<br>☐ Nativo de Hawái o de las islas del Pacífico<br>☐ Blanco<br>☐ Otro<br>☐ No sabe<br>((Seleccione todo lo que corresponda.))
Persona 5: ¿Cuál es la identidad étnica de esta persona?	◯ Hispano o latino<br>◯ Ni hispano ni latino<br>◯ Otra<br>◯ No sabe
Persona 5: ¿Cuál es el nivel de educación de esta persona?	◯ No tiene educación formal<br>◯ Kínder a 8° grado<br>◯ Estudios de educación secundaria<br>◯ Equivalencia de educación secundaria (GED)<br>◯ Diploma de educación secundaria<br>◯ Estudios de educación superior<br>◯ Título universitario<br>◯ Estudios de posgrado o más<br>◯ No sabe
Persona 5: ¿Cuál de las siguientes es la mejor descripción de la situación laboral actual de esta persona?	◯ Empleo a tiempo completo<br>◯ Empleo a tiempo parcial<br>◯ En busca de empleo<br>◯ Jubilado<br>◯ Ama de casa<br>◯ Estudiante<br>◯ Permiso de maternidad o paternidad<br>◯ Permiso por enfermedad<br>◯ Desempleado por discapacidad<br>◯ Otra<br>◯ No sabe
Persona 5: ¿Se considera esta persona actualmente empleado por cuenta propia (contratista independiente, trabajador esporádico (gig), etc.)?	◯ Sí<br>◯ No<br>◯ No sabe

Persona 5: ¿Trabaja esta persona actualmente en alguno de los siguientes ambientes de alto riesgo de contagio de COVID-19?

- ☐ Ambiente de asistencia médica (hospital, clínica, centro de urgencias, etc.)
- ☐ Ambiente residencial denso (hogar de ancianos, otro centro de asistencia de larga duración)
- ☐ Prisión o cárcel
- ☐ Establecimiento de envasado de carne
- ☐ Establecimiento de envío o distribución
- ☐ Establecimiento minorista de alto volumen (tienda de provisiones, etc.)
- ☐ No sabe

Persona 5: ¿Le ofrece el empleador a esta persona alguno de los siguientes beneficios en su empleo principal actual?

- ☐ Permiso por enfermedad con goce de sueldo
- ☐ Vacaciones o permiso personal con goce de sueldo
- ☐ Seguro de salud
- ☐ Seguro de discapacidad
- ☐ Plan de jubilación
- ☐ Otro
- ☐ No sabe

((Seleccione todo lo que corresponda.))

Persona 5: En una escala de 0 (definitivamente no va a suceder) a 10 (definitivamente va a suceder), ¿qué tan probable es que esta persona pierda su empleo debido a la pandemia de COVID-19?

____

Persona 5: En una escala de 0 (definitivamente no va a suceder) a 10 (definitivamente va a suceder), ¿qué tan probable es que a esta persona se le asignen menos horas de trabajo debido a la pandemia de COVID-19?

____

	Todo el tiempo (100%)	la mayor parte del tiempo (75%)	La mitad del tiempo (50%)	Menos de la mitad del tiempo (25%)	Nunca (0%)
Persona 5: Actualmente ¿con qué frecuencia se le exige a esta persona que trabaje fuera de su residencia?	○	○	○	○	○
Persona 5: Actualmente ¿con qué frecuencia se encuentra esta persona físicamente cerca de sus compañeros de trabajo mientras trabaja fuera de su residencia?	○	○	○	○	○
Persona 5: Actualmente ¿con qué frecuencia se encuentra esta persona físicamente cerca de sus clientes mientras trabaja fuera de su residencia?	○	○	○	○	○

Persona 5: ¿Piensa esta persona vacunarse contra la COVID-19 cuando se ofrezca una vacuna?

- ○ Sí
- ○ No
- ○ No sabe

Persona 5: En las últimas dos semanas, ¿ha tenido esta persona algún síntoma de COVID-19 (tos, fiebre, dificultad para respirar, fatiga, dolores de cuerpo, diarrea, goteo nasal o pérdida del sentido del olfato o del gusto)?	◯ Sí<br>◯ No<br>◯ No sabe
Persona 5: ¿Cuándo le comenzaron los síntomas de COVID-19 a esta persona?	________________________
Persona 5: En vista de sus síntomas, ¿le preocupa a esta persona la posibilidad de tener COVID-19?	◯ Sí<br>◯ No<br>◯ No sabe
Persona 5: ¿Fue esta persona objeto de prejuicio o discriminación debido a sus síntomas?	◯ Sí<br>◯ No<br>◯ No sabe
Persona 5: ¿Qué hizo esta persona en vista de sus síntomas?	☐ Nada<br>☐ Tomó medicamentos sin receta (ibuprofeno, acetaminofén, etc.)<br>☐ Consultó por teléfono a un proveedor de asistencia médica<br>☐ Fue al consultorio de un proveedor de asistencia médica<br>☐ Fue a una clínica o una farmacia minorista<br>☐ Fue a un centro de urgencias (FASTMed, etc.)<br>☐ Fue a la sala de emergencias<br>☐ Fue ingresado al hospital<br>☐ Otra cosa<br>☐ No sabe<br>((Seleccione todo lo que corresponda.))
Persona 5: ¿Qué otra cosa hizo esta persona en vista de sus síntomas?	________________________
Persona 5: ¿Le dijo un proveedor de asistencia médica a esta persona que era posible que tuviera COVID-19?	◯ Sí<br>◯ No<br>◯ No sabe
Persona 5: Si a esta persona le hicieron una prueba de COVID-19 en vista de sus síntomas, ¿cuál fue el resultado?	◯ Pendiente<br>◯ Positivo<br>◯ Negativo<br>◯ No concluyente<br>◯ No se hizo la prueba<br>◯ No sabe
Persona 5: ¿Cuántos días estuvo hospitalizada esta persona?	________________________
Persona 5: ¿Se le hicieron a esta persona las siguientes intervenciones durante su hospitalización?	☐ Oxígeno adicional por la nariz<br>☐ Tratamiento en la Unidad de Cuidados Intensivos (Intensive Care Unit, ICU)<br>☐ Ventilación mecánica (intubación o tubo de respiración)<br>☐ No sabe
Persona 5: ¿Ha vuelto esta persona a su salud normal?	◯ Sí<br>◯ No<br>◯ No sabe

Persona 5: ¿Cuáles de las siguientes medidas tomó esta persona para proteger a sus amigos y familiares después de que comenzaron sus síntomas?	☐ Ponerse mascarilla con mayor frecuencia<br>☐ Lavarse las manos con agua y jabón con mayor frecuencia<br>☐ Limpiarse las manos con desinfectante con mayor frecuencia<br>☐ Aislarse en su casa con mayor frecuencia<br>☐ Quedarse en casa con mayor frecuencia<br>☐ Ponerse guantes desechables con mayor frecuencia<br>☐ No sabe

Por cada persona adicional en su hogar, proporcione la siguiente informacion.

Persona 6: ¿Cuál es su relación con esta persona?	◯ Pareja o cónyuge<br>◯ Hijo o hija<br>◯ Padre o madre<br>◯ Hermano o hermana<br>◯ Otra familiar<br>◯ Proveedor de cuidado infantil u otros cuidados en casa<br>◯ Otra
Persona 6: Especifique su relación con esta persona.	__________________________________
Persona 6: ¿Qué edad tiene esta persona?	__________________________________<br>((Especifique la edad en años))
Persona 6: ¿Cuál es el sexo de esta persona?	◯ Femenino<br>◯ Masculino
Persona 6: ¿Cuál es la raza de esta persona?	☐ Indio americano o nativo de Alaska<br>☐ Asiático<br>☐ Negro o afroestadounidense<br>☐ Nativo de Hawái o de las islas del Pacífico<br>☐ Blanco<br>☐ Otro<br>☐ No sabe<br>((Seleccione todo lo que corresponda.))
Persona 6: ¿Cuál es la identidad étnica de esta persona?	◯ Hispano o latino<br>◯ Ni hispano ni latino<br>◯ Otra<br>◯ No sabe
Persona 6: ¿Cuál es el nivel de educación de esta persona?	◯ No tiene educación formal<br>◯ Kínder a 8° grado<br>◯ Estudios de educación secundaria<br>◯ Equivalencia de educación secundaria (GED)<br>◯ Diploma de educación secundaria<br>◯ Estudios de educación superior<br>◯ Título universitario<br>◯ Estudios de posgrado o más<br>◯ No sabe

Persona 6: ¿Cuál de las siguientes es la mejor descripción de la situación laboral actual de esta persona?	◯ Empleo a tiempo completo<br>◯ Empleo a tiempo parcial<br>◯ En busca de empleo<br>◯ Jubilado<br>◯ Ama de casa<br>◯ Estudiante<br>◯ Permiso de maternidad o paternidad<br>◯ Permiso por enfermedad<br>◯ Desempleado por discapacidad<br>◯ Otra<br>◯ No sabe
Persona 6: ¿Se considera esta persona actualmente empleado por cuenta propia (contratista independiente, trabajador esporádico (gig), etc.)?	◯ Sí<br>◯ No<br>◯ No sabe
Persona 6: ¿Trabaja esta persona actualmente en alguno de los siguientes ambientes de alto riesgo de contagio de COVID-19?	☐ Ambiente de asistencia médica (hospital, clínica, centro de urgencias, etc.)<br>☐ Ambiente residencial denso (hogar de ancianos, otro centro de asistencia de larga duración)<br>☐ Prisión o cárcel<br>☐ Establecimiento de envasado de carne<br>☐ Establecimiento de envío o distribución<br>☐ Establecimiento minorista de alto volumen (tienda de provisiones, etc.)<br>☐ No sabe
Persona 6: ¿Le ofrece el empleador a esta persona alguno de los siguientes beneficios en su empleo principal actual?	☐ Permiso por enfermedad con goce de sueldo<br>☐ Vacaciones o permiso personal con goce de sueldo<br>☐ Seguro de salud<br>☐ Seguro de discapacidad<br>☐ Plan de jubilación<br>☐ Otro<br>☐ No sabe<br>((Seleccione todo lo que corresponda.))
Persona 6: En una escala de 0 (definitivamente no va a suceder) a 10 (definitivamente va a suceder), ¿qué tan probable es que esta persona pierda su empleo debido a la pandemia de COVID-19?	__________________________________
Persona 6: En una escala de 0 (definitivamente no va a suceder) a 10 (definitivamente va a suceder), ¿qué tan probable es que a esta persona se le asignen menos horas de trabajo debido a la pandemia de COVID-19?	__________________________________

	Todo el tiempo (100%)	la mayor parte del tiempo (75%)	La mitad del tiempo (50%)	Menos de la mitad del tiempo (25%)	Nunca (0%)
Persona 6: Actualmente ¿con qué frecuencia se le exige a esta persona que trabaje fuera de su residencia?	◯	◯	◯	◯	◯

Persona 6: Actualmente ¿con qué frecuencia se encuentra esta persona físicamente cerca de sus compañeros de trabajo mientras trabaja fuera de su residencia? ○ ○ ○ ○ ○

Persona 6: Actualmente ¿con qué frecuencia se encuentra esta persona físicamente cerca de sus clientes mientras trabaja fuera de su residencia? ○ ○ ○ ○ ○

Persona 6: ¿Piensa esta persona vacunarse contra la COVID-19 cuando se ofrezca una vacuna?

○ Sí
○ No
○ No sabe

Persona 6: En las últimas dos semanas, ¿ha tenido esta persona algún síntoma de COVID-19 (tos, fiebre, dificultad para respirar, fatiga, dolores de cuerpo, diarrea, goteo nasal o pérdida del sentido del olfato o del gusto)?

○ Sí
○ No
○ No sabe

Persona 6: ¿Cuándo le comenzaron los síntomas de COVID-19 a esta persona?

____

Persona 6: En vista de sus síntomas, ¿le preocupa a esta persona la posibilidad de tener COVID-19?

○ Sí
○ No
○ No sabe

Persona 6: ¿Fue esta persona objeto de prejuicio o discriminación debido a sus síntomas?

○ Sí
○ No
○ No sabe

Persona 6: ¿Qué hizo esta persona en vista de sus síntomas?

☐ Nada
☐ Tomó medicamentos sin receta (ibuprofeno, acetaminofén, etc.)
☐ Consultó por teléfono a un proveedor de asistencia médica
☐ Fue al consultorio de un proveedor de asistencia médica
☐ Fue a una clínica o una farmacia minorista
☐ Fue a un centro de urgencias (FASTMed, etc.)
☐ Fue a la sala de emergencias
☐ Fue ingresado al hospital
☐ Otra cosa
☐ No sabe
((Seleccione todo lo que corresponda.))

Persona 6 ¿Qué otra cosa hizo esta persona en vista de sus síntomas?

____

Persona 6: ¿Le dijo un proveedor de asistencia médica a esta persona que era posible que tuviera COVID-19?

○ Sí
○ No
○ No sabe

Persona 6: Si a esta persona le hicieron una prueba de COVID-19 en vista de sus síntomas, ¿cuál fue el resultado?

◯ Pendiente
◯ Positivo
◯ Negativo
◯ No concluyente
◯ No se hizo la prueba
◯ No sabe

Persona 6: ¿Cuántos días estuvo hospitalizada esta persona?

____

Persona 6: ¿Se le hicieron a esta persona las siguientes intervenciones durante su hospitalización?

☐ Oxígeno adicional por la nariz
☐ Tratamiento en la Unidad de Cuidados Intensivos (Intensive Care Unit, ICU)
☐ Ventilación mecánica (intubación o tubo de respiración)
☐ No sabe

Persona 6: ¿Ha vuelto esta persona a su salud normal?

◯ Sí
◯ No
◯ No sabe

Persona 6: ¿Cuáles de las siguientes medidas tomó esta persona para proteger a sus amigos y familiares después de que comenzaron sus síntomas?

☐ Ponerse mascarilla con mayor frecuencia
☐ Lavarse las manos con agua y jabón con mayor frecuencia
☐ Limpiarse las manos con desinfectante con mayor frecuencia
☐ Aislarse en su casa con mayor frecuencia
☐ Quedarse en casa con mayor frecuencia
☐ Ponerse guantes desechables con mayor frecuencia
☐ No sabe

Por cada persona adicional en su hogar, proporcione la siguiente informacion.

Persona 7: ¿Cuál es su relación con esta persona?

◯ Pareja o cónyuge
◯ Hijo o hija
◯ Padre o madre
◯ Hermano o hermana
◯ Otra familiar
◯ Proveedor de cuidado infantil u otros cuidados en casa
◯ Otra

Persona 7: Especifique su relación con esta persona.

____

Persona 7: ¿Qué edad tiene esta persona?

____
((Especifique la edad en años))

Persona 7: ¿Cuál es el sexo de esta persona?

◯ Femenino
◯ Masculino

Persona 7: ¿Cuál es la raza de esta persona?	☐ Indio americano o nativo de Alaska<br>☐ Asiático<br>☐ Negro o afroestadounidense<br>☐ Nativo de Hawái o de las islas del Pacífico<br>☐ Blanco<br>☐ Otro<br>☐ No sabe<br>((Seleccione todo lo que corresponda.))
Persona 7: ¿Cuál es la identidad étnica de esta persona?	◯ Hispano o latino<br>◯ Ni hispano ni latino<br>◯ Otra<br>◯ No sabe
Persona 7: ¿Cuál es el nivel de educación de esta persona?	◯ No tiene educación formal<br>◯ Kínder a 8° grado<br>◯ Estudios de educación secundaria<br>◯ Equivalencia de educación secundaria (GED)<br>◯ Diploma de educación secundaria<br>◯ Estudios de educación superior<br>◯ Título universitario<br>◯ Estudios de posgrado o más<br>◯ No sabe
Persona 7: ¿Cuál de las siguientes es la mejor descripción de la situación laboral actual de esta persona?	◯ Empleo a tiempo completo<br>◯ Empleo a tiempo parcial<br>◯ En busca de empleo<br>◯ Jubilado<br>◯ Ama de casa<br>◯ Estudiante<br>◯ Permiso de maternidad o paternidad<br>◯ Permiso por enfermedad<br>◯ Desempleado por discapacidad<br>◯ Otra<br>◯ No sabe
Persona 7: ¿Se considera esta persona actualmente empleado por cuenta propia (contratista independiente, trabajador esporádico (gig), etc.)?	◯ Sí<br>◯ No<br>◯ No sabe
Persona 7: ¿Trabaja esta persona actualmente en alguno de los siguientes ambientes de alto riesgo de contagio de COVID-19?	☐ Ambiente de asistencia médica (hospital, clínica, centro de urgencias, etc.)<br>☐ Ambiente residencial denso (hogar de ancianos, otro centro de asistencia de larga duración)<br>☐ Prisión o cárcel<br>☐ Establecimiento de envasado de carne<br>☐ Establecimiento de envío o distribución<br>☐ Establecimiento minorista de alto volumen (tienda de provisiones, etc.)<br>☐ No sabe
Persona 7: ¿Le ofrece el empleador a esta persona alguno de los siguientes beneficios en su empleo principal actual?	☐ Permiso por enfermedad con goce de sueldo<br>☐ Vacaciones o permiso personal con goce de sueldo<br>☐ Seguro de salud<br>☐ Seguro de discapacidad<br>☐ Plan de jubilación<br>☐ Otro<br>☐ No sabe<br>((Seleccione todo lo que corresponda.))

Persona 7: En una escala de 0 (definitivamente no va a suceder) a 10 (definitivamente va a suceder), ¿qué tan probable es que esta persona pierda su empleo debido a la pandemia de COVID-19? __________________________________

Persona 7: En una escala de 0 (definitivamente no va a suceder) a 10 (definitivamente va a suceder), ¿qué tan probable es que a esta persona se le asignen menos horas de trabajo debido a la pandemia de COVID-19? __________________________________

	Todo el tiempo (100%)	la mayor parte del tiempo (75%)	La mitad del tiempo (50%)	Menos de la mitad del tiempo (25%)	Nunca (0%)
Persona 7: Actualmente ¿con qué frecuencia se le exige a esta persona que trabaje fuera de su residencia?	◯	◯	◯	◯	◯
Persona 7: Actualmente ¿con qué frecuencia se encuentra esta persona físicamente cerca de sus compañeros de trabajo mientras trabaja fuera de su residencia?	◯	◯	◯	◯	◯
Persona 7: Actualmente ¿con qué frecuencia se encuentra esta persona físicamente cerca de sus clientes mientras trabaja fuera de su residencia?	◯	◯	◯	◯	◯

Persona 7: ¿Piensa esta persona vacunarse contra la COVID-19 cuando se ofrezca una vacuna?
◯ Sí
◯ No
◯ No sabe

Persona 7: En las últimas dos semanas, ¿ha tenido esta persona algún síntoma de COVID-19 (tos, fiebre, dificultad para respirar, fatiga, dolores de cuerpo, diarrea, goteo nasal o pérdida del sentido del olfato o del gusto)?
◯ Sí
◯ No
◯ No sabe

Persona 7: ¿Cuándo le comenzaron los síntomas de COVID-19 a esta persona? __________________________________

Persona 7: En vista de sus síntomas, ¿le preocupa a esta persona la posibilidad de tener COVID-19?
◯ Sí
◯ No
◯ No sabe

Persona 7: ¿Fue esta persona objeto de prejuicio o discriminación debido a sus síntomas?
◯ Sí
◯ No
◯ No sabe

Persona 7: ¿Qué hizo esta persona en vista de sus síntomas?	☐ Nada<br>☐ Tomó medicamentos sin receta (ibuprofeno, acetaminofén, etc.)<br>☐ Consultó por teléfono a un proveedor de asistencia médica<br>☐ Fue al consultorio de un proveedor de asistencia médica<br>☐ Fue a una clínica o una farmacia minorista<br>☐ Fue a un centro de urgencias (FASTMed, etc.)<br>☐ Fue a la sala de emergencias<br>☐ Fue ingresado al hospital<br>☐ Otra cosa<br>☐ No sabe<br>((Seleccione todo lo que corresponda.))
Persona 7 ¿Qué otra cosa hizo esta persona en vista de sus síntomas?	__________________________________
Persona 7: ¿Le dijo un proveedor de asistencia médica a esta persona que era posible que tuviera COVID-19?	◯ Sí<br>◯ No<br>◯ No sabe
Persona 7: Si a esta persona le hicieron una prueba de COVID-19 en vista de sus síntomas, ¿cuál fue el resultado?	◯ Pendiente<br>◯ Positivo<br>◯ Negativo<br>◯ No concluyente<br>◯ No se hizo la prueba<br>◯ No sabe
Persona 7: ¿Cuántos días estuvo hospitalizada esta persona?	__________________________________
Persona 7: ¿Se le hicieron a esta persona las siguientes intervenciones durante su hospitalización?	☐ Oxígeno adicional por la nariz<br>☐ Tratamiento en la Unidad de Cuidados Intensivos (Intensive Care Unit, ICU)<br>☐ Ventilación mecánica (intubación o tubo de respiración)<br>☐ No sabe
Persona 7: ¿Ha vuelto esta persona a su salud normal?	◯ Sí<br>◯ No<br>◯ No sabe
Persona 7: ¿Cuáles de las siguientes medidas tomó esta persona para proteger a sus amigos y familiares después de que comenzaron sus síntomas?	☐ Ponerse mascarilla con mayor frecuencia<br>☐ Lavarse las manos con agua y jabón con mayor frecuencia<br>☐ Limpiarse las manos con desinfectante con mayor frecuencia<br>☐ Aislarse en su casa con mayor frecuencia<br>☐ Quedarse en casa con mayor frecuencia<br>☐ Ponerse guantes desechables con mayor frecuencia<br>☐ No sabe

Por cada persona adicional en su hogar, proporcione la siguiente informacion.

Persona 8: ¿Cuál es su relación con esta persona?

- ◯ Pareja o cónyuge
- ◯ Hijo o hija
- ◯ Padre o madre
- ◯ Hermano o hermana
- ◯ Otra familiar
- ◯ Proveedor de cuidado infantil u otros cuidados en casa
- ◯ Otra

Persona 8: Especifique su relación con esta persona.

____

Persona 8: ¿Qué edad tiene esta persona?

____
((Especifique la edad en años))

Persona 8: ¿Cuál es el sexo de esta persona?

- ◯ Femenino
- ◯ Masculino

Persona 8: ¿Cuál es la raza de esta persona?

- ☐ Indio americano o nativo de Alaska
- ☐ Asiático
- ☐ Negro o afroestadounidense
- ☐ Nativo de Hawái o de las islas del Pacífico
- ☐ Blanco
- ☐ Otro
- ☐ No sabe

((Seleccione todo lo que corresponda.))

Persona 8: ¿Cuál es la identidad étnica de esta persona?

- ◯ Hispano o latino
- ◯ Ni hispano ni latino
- ◯ Otra
- ◯ No sabe

Persona 8: ¿Cuál es el nivel de educación de esta persona?

- ◯ No tiene educación formal
- ◯ Kínder a 8° grado
- ◯ Estudios de educación secundaria
- ◯ Equivalencia de educación secundaria (GED)
- ◯ Diploma de educación secundaria
- ◯ Estudios de educación superior
- ◯ Título universitario
- ◯ Estudios de posgrado o más
- ◯ No sabe

Persona 8: ¿Cuál de las siguientes es la mejor descripción de la situación laboral actual de esta persona?

- ◯ Empleo a tiempo completo
- ◯ Empleo a tiempo parcial
- ◯ En busca de empleo
- ◯ Jubilado
- ◯ Ama de casa
- ◯ Estudiante
- ◯ Permiso de maternidad o paternidad
- ◯ Permiso por enfermedad
- ◯ Desempleado por discapacidad
- ◯ Otra
- ◯ No sabe

Persona 8: ¿Se considera esta persona actualmente empleado por cuenta propia (contratista independiente, trabajador esporádico (gig), etc.)?

- ◯ Sí
- ◯ No
- ◯ No sabe

Persona 8: ¿Trabaja esta persona actualmente en alguno de los siguientes ambientes de alto riesgo de contagio de COVID-19?	☐ Ambiente de asistencia médica (hospital, clínica, centro de urgencias, etc.)<br>☐ Ambiente residencial denso (hogar de ancianos, otro centro de asistencia de larga duración)<br>☐ Prisión o cárcel<br>☐ Establecimiento de envasado de carne<br>☐ Establecimiento de envío o distribución<br>☐ Establecimiento minorista de alto volumen (tienda de provisiones, etc.)<br>☐ No sabe
Persona 8: ¿Le ofrece el empleador a esta persona alguno de los siguientes beneficios en su empleo principal actual?	☐ Permiso por enfermedad con goce de sueldo<br>☐ Vacaciones o permiso personal con goce de sueldo<br>☐ Seguro de salud<br>☐ Seguro de discapacidad<br>☐ Plan de jubilación<br>☐ Otro<br>☐ No sabe<br>((Seleccione todo lo que corresponda.))
Persona 8: En una escala de 0 (definitivamente no va a suceder) a 10 (definitivamente va a suceder), ¿qué tan probable es que esta persona pierda su empleo debido a la pandemia de COVID-19?	__________________________________
Persona 8: En una escala de 0 (definitivamente no va a suceder) a 10 (definitivamente va a suceder), ¿qué tan probable es que a esta persona se le asignen menos horas de trabajo debido a la pandemia de COVID-19?	__________________________________

	Todo el tiempo (100%)	la mayor parte del tiempo (75%)	La mitad del tiempo (50%)	Menos de la mitad del tiempo (25%)	Nunca (0%)
Persona 8: Actualmente ¿con qué frecuencia se le exige a esta persona que trabaje fuera de su residencia?	○	○	○	○	○
Persona 8: Actualmente ¿con qué frecuencia se encuentra esta persona físicamente cerca de sus compañeros de trabajo mientras trabaja fuera de su residencia?	○	○	○	○	○
Persona 8: Actualmente ¿con qué frecuencia se encuentra esta persona físicamente cerca de sus clientes mientras trabaja fuera de su residencia?	○	○	○	○	○

Persona 8: ¿Piensa esta persona vacunarse contra la COVID-19 cuando se ofrezca una vacuna?	○ Sí<br>○ No<br>○ No sabe

Persona 8: En las últimas dos semanas, ¿ha tenido esta persona algún síntoma de COVID-19 (tos, fiebre, dificultad para respirar, fatiga, dolores de cuerpo, diarrea, goteo nasal o pérdida del sentido del olfato o del gusto)?	◯ Sí<br>◯ No<br>◯ No sabe
Persona 8: ¿Cuándo le comenzaron los síntomas de COVID-19 a esta persona?	______________________
Persona 8: En vista de sus síntomas, ¿le preocupa a esta persona la posibilidad de tener COVID-19?	◯ Sí<br>◯ No<br>◯ No sabe
Persona 8: ¿Fue esta persona objeto de prejuicio o discriminación debido a sus síntomas?	◯ Sí<br>◯ No<br>◯ No sabe
Persona 8: ¿Qué hizo esta persona en vista de sus síntomas?	☐ Nada<br>☐ Tomó medicamentos sin receta (ibuprofeno, acetaminofén, etc.)<br>☐ Consultó por teléfono a un proveedor de asistencia médica<br>☐ Fue al consultorio de un proveedor de asistencia médica<br>☐ Fue a una clínica o una farmacia minorista<br>☐ Fue a un centro de urgencias (FASTMed, etc.)<br>☐ Fue a la sala de emergencias<br>☐ Fue ingresado al hospital<br>☐ Otra cosa<br>☐ No sabe<br>((Seleccione todo lo que corresponda.))
Persona 8 ¿Qué otra cosa hizo esta persona en vista de sus síntomas?	______________________
Persona 8: ¿Le dijo un proveedor de asistencia médica a esta persona que era posible que tuviera COVID-19?	◯ Sí<br>◯ No<br>◯ No sabe
Persona 8: Si a esta persona le hicieron una prueba de COVID-19 en vista de sus síntomas, ¿cuál fue el resultado?	◯ Pendiente<br>◯ Positivo<br>◯ Negativo<br>◯ No concluyente<br>◯ No se hizo la prueba<br>◯ No sabe
Persona 8: ¿Cuántos días estuvo hospitalizada esta persona?	______________________
Persona 8: ¿Se le hicieron a esta persona las siguientes intervenciones durante su hospitalización?	☐ Oxígeno adicional por la nariz<br>☐ Tratamiento en la Unidad de Cuidados Intensivos (Intensive Care Unit, ICU)<br>☐ Ventilación mecánica (intubación o tubo de respiración)<br>☐ No sabe
Persona 8: ¿Ha vuelto esta persona a su salud normal?	◯ Sí<br>◯ No<br>◯ No sabe

Persona 8: ¿Cuáles de las siguientes medidas tomó esta persona para proteger a sus amigos y familiares después de que comenzaron sus síntomas?

- ☐ Ponerse mascarilla con mayor frecuencia
- ☐ Lavarse las manos con agua y jabón con mayor frecuencia
- ☐ Limpiarse las manos con desinfectante con mayor frecuencia
- ☐ Aislarse en su casa con mayor frecuencia
- ☐ Quedarse en casa con mayor frecuencia
- ☐ Ponerse guantes desechables con mayor frecuencia
- ☐ No sabe

Por cada persona adicional en su hogar, proporcione la siguiente informacion.

Persona 9: ¿Cuál es su relación con esta persona?

- ○ Pareja o cónyuge
- ○ Hijo o hija
- ○ Padre o madre
- ○ Hermano o hermana
- ○ Otra familiar
- ○ Proveedor de cuidado infantil u otros cuidados en casa
- ○ Otra

Persona 9: Especifique su relación con esta persona.

____

Persona 9: ¿Qué edad tiene esta persona?

____
((Especifique la edad en años))

Persona 9: ¿Cuál es el sexo de esta persona?

- ○ Femenino
- ○ Masculino

Persona 9: ¿Cuál es la raza de esta persona?

- ☐ Indio americano o nativo de Alaska
- ☐ Asiático
- ☐ Negro o afroestadounidense
- ☐ Nativo de Hawái o de las islas del Pacífico
- ☐ Blanco
- ☐ Otro
- ☐ No sabe

((Seleccione todo lo que corresponda.))

Persona 9: ¿Cuál es la identidad étnica de esta persona?

- ○ Hispano o latino
- ○ Ni hispano ni latino
- ○ Otra
- ○ No sabe

Persona 9: ¿Cuál es el nivel de educación de esta persona?

- ○ No tiene educación formal
- ○ Kínder a 8° grado
- ○ Estudios de educación secundaria
- ○ Equivalencia de educación secundaria (GED)
- ○ Diploma de educación secundaria
- ○ Estudios de educación superior
- ○ Título universitario
- ○ Estudios de posgrado o más
- ○ No sabe

Persona 9: ¿Cuál de las siguientes es la mejor descripción de la situación laboral actual de esta persona?	◯ Empleo a tiempo completo<br>◯ Empleo a tiempo parcial<br>◯ En busca de empleo<br>◯ Jubilado<br>◯ Ama de casa<br>◯ Estudiante<br>◯ Permiso de maternidad o paternidad<br>◯ Permiso por enfermedad<br>◯ Desempleado por discapacidad<br>◯ Otra<br>◯ No sabe
Persona 9: ¿Se considera esta persona actualmente empleado por cuenta propia (contratista independiente, trabajador esporádico (gig), etc.)?	◯ Sí<br>◯ No<br>◯ No sabe
Persona 9: ¿Trabaja esta persona actualmente en alguno de los siguientes ambientes de alto riesgo de contagio de COVID-19?	☐ Ambiente de asistencia médica (hospital, clínica, centro de urgencias, etc.)<br>☐ Ambiente residencial denso (hogar de ancianos, otro centro de asistencia de larga duración)<br>☐ Prisión o cárcel<br>☐ Establecimiento de envasado de carne<br>☐ Establecimiento de envío o distribución<br>☐ Establecimiento minorista de alto volumen (tienda de provisiones, etc.)<br>☐ No sabe
Persona 9: ¿Le ofrece el empleador a esta persona alguno de los siguientes beneficios en su empleo principal actual?	☐ Permiso por enfermedad con goce de sueldo<br>☐ Vacaciones o permiso personal con goce de sueldo<br>☐ Seguro de salud<br>☐ Seguro de discapacidad<br>☐ Plan de jubilación<br>☐ Otro<br>☐ No sabe<br>((Seleccione todo lo que corresponda.))
Persona 9: En una escala de 0 (definitivamente no va a suceder) a 10 (definitivamente va a suceder), ¿qué tan probable es que esta persona pierda su empleo debido a la pandemia de COVID-19?	______________________
Persona 9: En una escala de 0 (definitivamente no va a suceder) a 10 (definitivamente va a suceder), ¿qué tan probable es que a esta persona se le asignen menos horas de trabajo debido a la pandemia de COVID-19?	______________________

	Todo el tiempo (100%)	la mayor parte del tiempo (75%)	La mitad del tiempo (50%)	Menos de la mitad del tiempo (25%)	Nunca (0%)
Persona 9: Actualmente ¿con qué frecuencia se le exige a esta persona que trabaje fuera de su residencia?	◯	◯	◯	◯	◯

Persona 9: Actualmente ¿con qué frecuencia se encuentra esta persona físicamente cerca de sus compañeros de trabajo mientras trabaja fuera de su residencia?

○ ○ ○ ○ ○

Persona 9: Actualmente ¿con qué frecuencia se encuentra esta persona físicamente cerca de sus clientes mientras trabaja fuera de su residencia?

○ ○ ○ ○ ○

Persona 9: ¿Piensa esta persona vacunarse contra la COVID-19 cuando se ofrezca una vacuna?

- ○ Sí
- ○ No
- ○ No sabe

Persona 9: En las últimas dos semanas, ¿ha tenido esta persona algún síntoma de COVID-19 (tos, fiebre, dificultad para respirar, fatiga, dolores de cuerpo, diarrea, goteo nasal o pérdida del sentido del olfato o del gusto)?

- ○ Sí
- ○ No
- ○ No sabe

Persona 9: ¿Cuándo le comenzaron los síntomas de COVID-19 a esta persona?

____

Persona 9: En vista de sus síntomas, ¿le preocupa a esta persona la posibilidad de tener COVID-19?

- ○ Sí
- ○ No
- ○ No sabe

Persona 9: ¿Fue esta persona objeto de prejuicio o discriminación debido a sus síntomas?

- ○ Sí
- ○ No
- ○ No sabe

Persona 9: ¿Qué hizo esta persona en vista de sus síntomas?

- ☐ Nada
- ☐ Tomó medicamentos sin receta (ibuprofeno, acetaminofén, etc.)
- ☐ Consultó por teléfono a un proveedor de asistencia médica
- ☐ Fue al consultorio de un proveedor de asistencia médica
- ☐ Fue a una clínica o una farmacia minorista
- ☐ Fue a un centro de urgencias (FASTMed, etc.)
- ☐ Fue a la sala de emergencias
- ☐ Fue ingresado al hospital
- ☐ Otra cosa
- ☐ No sabe

((Seleccione todo lo que corresponda.))

Persona 9 ¿Qué otra cosa hizo esta persona en vista de sus síntomas?

____

Persona 9: ¿Le dijo un proveedor de asistencia médica a esta persona que era posible que tuviera COVID-19?

- ○ Sí
- ○ No
- ○ No sabe

Persona 9: Si a esta persona le hicieron una prueba de COVID-19 en vista de sus síntomas, ¿cuál fue el resultado?

- ◯ Pendiente
- ◯ Positivo
- ◯ Negativo
- ◯ No concluyente
- ◯ No se hizo la prueba
- ◯ No sabe

Persona 9: ¿Cuántos días estuvo hospitalizada esta persona?

____

Persona 9: ¿Se le hicieron a esta persona las siguientes intervenciones durante su hospitalización?

- ☐ Oxígeno adicional por la nariz
- ☐ Tratamiento en la Unidad de Cuidados Intensivos (Intensive Care Unit, ICU)
- ☐ Ventilación mecánica (intubación o tubo de respiración)
- ☐ No sabe

Persona 9: ¿Ha vuelto esta persona a su salud normal?

- ◯ Sí
- ◯ No
- ◯ No sabe

Persona 9: ¿Cuáles de las siguientes medidas tomó esta persona para proteger a sus amigos y familiares después de que comenzaron sus síntomas?

- ☐ Ponerse mascarilla con mayor frecuencia
- ☐ Lavarse las manos con agua y jabón con mayor frecuencia
- ☐ Limpiarse las manos con desinfectante con mayor frecuencia
- ☐ Aislarse en su casa con mayor frecuencia
- ☐ Quedarse en casa con mayor frecuencia
- ☐ Ponerse guantes desechables con mayor frecuencia
- ☐ No sabe

Por cada persona adicional en su hogar, proporcione la siguiente informacion.

Persona 10: ¿Cuál es su relación con esta persona?

- ◯ Pareja o cónyuge
- ◯ Hijo o hija
- ◯ Padre o madre
- ◯ Hermano o hermana
- ◯ Otra familiar
- ◯ Proveedor de cuidado infantil u otros cuidados en casa
- ◯ Otra

Persona 10: Especifique su relación con esta persona.

____

Persona 10: ¿Qué edad tiene esta persona?

____
((Especifique la edad en años))

Persona 10: ¿Cuál es el sexo de esta persona?

- ◯ Femenino
- ◯ Masculino

Persona 10: ¿Cuál es la raza de esta persona?	☐ Indio americano o nativo de Alaska<br>☐ Asiático<br>☐ Negro o afroestadounidense<br>☐ Nativo de Hawái o de las islas del Pacífico<br>☐ Blanco<br>☐ Otro<br>☐ No sabe<br>((Seleccione todo lo que corresponda.))
Persona 10: ¿Cuál es la identidad étnica de esta persona?	○ Hispano o latino<br>○ Ni hispano ni latino<br>○ Otra<br>○ No sabe
Persona 10: ¿Cuál es el nivel de educación de esta persona?	○ No tiene educación formal<br>○ Kínder a 8° grado<br>○ Estudios de educación secundaria<br>○ Equivalencia de educación secundaria (GED)<br>○ Diploma de educación secundaria<br>○ Estudios de educación superior<br>○ Título universitario<br>○ Estudios de posgrado o más<br>○ No sabe
Persona 10: ¿Cuál de las siguientes es la mejor descripción de la situación laboral actual de esta persona?	○ Empleo a tiempo completo<br>○ Empleo a tiempo parcial<br>○ En busca de empleo<br>○ Jubilado<br>○ Ama de casa<br>○ Estudiante<br>○ Permiso de maternidad o paternidad<br>○ Permiso por enfermedad<br>○ Desempleado por discapacidad<br>○ Otra<br>○ No sabe
Persona 10: ¿Se considera esta persona actualmente empleado por cuenta propia (contratista independiente, trabajador esporádico (gig), etc.)?	○ Sí<br>○ No<br>○ No sabe
Persona 10: ¿Trabaja esta persona actualmente en alguno de los siguientes ambientes de alto riesgo de contagio de COVID-19?	☐ Ambiente de asistencia médica (hospital, clínica, centro de urgencias, etc.)<br>☐ Ambiente residencial denso (hogar de ancianos, otro centro de asistencia de larga duración)<br>☐ Prisión o cárcel<br>☐ Establecimiento de envasado de carne<br>☐ Establecimiento de envío o distribución<br>☐ Establecimiento minorista de alto volumen (tienda de provisiones, etc.)<br>☐ No sabe
Persona 10: ¿Le ofrece el empleador a esta persona alguno de los siguientes beneficios en su empleo principal actual?	☐ Permiso por enfermedad con goce de sueldo<br>☐ Vacaciones o permiso personal con goce de sueldo<br>☐ Seguro de salud<br>☐ Seguro de discapacidad<br>☐ Plan de jubilación<br>☐ Otro<br>☐ No sabe<br>((Seleccione todo lo que corresponda.))

Persona 10: En una escala de 0 (definitivamente no va a suceder) a 10 (definitivamente va a suceder), ¿qué tan probable es que esta persona pierda su empleo debido a la pandemia de COVID-19? ______________________________

Persona 10: En una escala de 0 (definitivamente no va a suceder) a 10 (definitivamente va a suceder), ¿qué tan probable es que a esta persona se le asignen menos horas de trabajo debido a la pandemia de COVID-19? ______________________________

	Todo el tiempo (100%)	la mayor parte del tiempo (75%)	La mitad del tiempo (50%)	Menos de la mitad del tiempo (25%)	Nunca (0%)
Persona 10: Actualmente ¿con qué frecuencia se le exige a esta persona que trabaje fuera de su residencia?	○	○	○	○	○
Persona 10: Actualmente ¿con qué frecuencia se encuentra esta persona físicamente cerca de sus compañeros de trabajo mientras trabaja fuera de su residencia?	○	○	○	○	○
Persona 10: Actualmente ¿con qué frecuencia se encuentra esta persona físicamente cerca de sus clientes mientras trabaja fuera de su residencia?	○	○	○	○	○

Persona 10: ¿Piensa esta persona vacunarse contra la COVID-19 cuando se ofrezca una vacuna?

○ Sí
○ No
○ No sabe

Persona 10: En las últimas dos semanas, ¿ha tenido esta persona algún síntoma de COVID-19 (tos, fiebre, dificultad para respirar, fatiga, dolores de cuerpo, diarrea, goteo nasal o pérdida del sentido del olfato o del gusto)?

○ Sí
○ No
○ No sabe

Persona 10: ¿Cuándo le comenzaron los síntomas de COVID-19 a esta persona? ______________________________

Persona 10: En vista de sus síntomas, ¿le preocupa a esta persona la posibilidad de tener COVID-19?

○ Sí
○ No
○ No sabe

Persona 10: ¿Fue esta persona objeto de prejuicio o discriminación debido a sus síntomas?

○ Sí
○ No
○ No sabe

Persona 10: ¿Qué hizo esta persona en vista de sus síntomas?	☐ Nada<br>☐ Tomó medicamentos sin receta (ibuprofeno, acetaminofén, etc.)<br>☐ Consultó por teléfono a un proveedor de asistencia médica<br>☐ Fue al consultorio de un proveedor de asistencia médica<br>☐ Fue a una clínica o una farmacia minorista<br>☐ Fue a un centro de urgencias (FASTMed, etc.)<br>☐ Fue a la sala de emergencias<br>☐ Fue ingresado al hospital<br>☐ Otra cosa<br>☐ No sabe<br>((Seleccione todo lo que corresponda.))
Persona 10 ¿Qué otra cosa hizo esta persona en vista de sus síntomas?	________________________
Persona 10: ¿Le dijo un proveedor de asistencia médica a esta persona que era posible que tuviera COVID-19?	◯ Sí<br>◯ No<br>◯ No sabe
Persona 10: Si a esta persona le hicieron una prueba de COVID-19 en vista de sus síntomas, ¿cuál fue el resultado?	◯ Pendiente<br>◯ Positivo<br>◯ Negativo<br>◯ No concluyente<br>◯ No se hizo la prueba<br>◯ No sabe
Persona 10: ¿Cuántos días estuvo hospitalizada esta persona?	________________________
Persona 10: ¿Se le hicieron a esta persona las siguientes intervenciones durante su hospitalización?	☐ Oxígeno adicional por la nariz<br>☐ Tratamiento en la Unidad de Cuidados Intensivos (Intensive Care Unit, ICU)<br>☐ Ventilación mecánica (intubación o tubo de respiración)<br>☐ No sabe
Persona 10: ¿Ha vuelto esta persona a su salud normal?	◯ Sí<br>◯ No<br>◯ No sabe
Persona 10: ¿Cuáles de las siguientes medidas tomó esta persona para proteger a sus amigos y familiares después de que comenzaron sus síntomas?	☐ Ponerse mascarilla con mayor frecuencia<br>☐ Lavarse las manos con agua y jabón con mayor frecuencia<br>☐ Limpiarse las manos con desinfectante con mayor frecuencia<br>☐ Aislarse en su casa con mayor frecuencia<br>☐ Quedarse en casa con mayor frecuencia<br>☐ Ponerse guantes desechables con mayor frecuencia<br>☐ No sabe

Por cada persona adicional en su hogar, proporcione la siguiente informacion.

Persona 11: ¿Cuál es su relación con esta persona?	◯ Pareja o cónyuge<br>◯ Hijo o hija<br>◯ Padre o madre<br>◯ Hermano o hermana<br>◯ Otra familiar<br>◯ Proveedor de cuidado infantil u otros cuidados en casa<br>◯ Otra
Persona 11: Especifique su relación con esta persona.	__________________________________
Persona 11: ¿Qué edad tiene esta persona?	__________________________________<br>((Especifique la edad en años))
Persona 11: ¿Cuál es el sexo de esta persona?	◯ Femenino<br>◯ Masculino
Persona 11: ¿Cuál es la raza de esta persona?	☐ Indio americano o nativo de Alaska<br>☐ Asiático<br>☐ Negro o afroestadounidense<br>☐ Nativo de Hawái o de las islas del Pacífico<br>☐ Blanco<br>☐ Otro<br>☐ No sabe<br>((Seleccione todo lo que corresponda.))
Persona 11: ¿Cuál es la identidad étnica de esta persona?	◯ Hispano o latino<br>◯ Ni hispano ni latino<br>◯ Otra<br>◯ No sabe
Persona 11: ¿Cuál es el nivel de educación de esta persona?	◯ No tiene educación formal<br>◯ Kínder a 8° grado<br>◯ Estudios de educación secundaria<br>◯ Equivalencia de educación secundaria (GED)<br>◯ Diploma de educación secundaria<br>◯ Estudios de educación superior<br>◯ Título universitario<br>◯ Estudios de posgrado o más<br>◯ No sabe
Persona 11: ¿Cuál de las siguientes es la mejor descripción de la situación laboral actual de esta persona?	◯ Empleo a tiempo completo<br>◯ Empleo a tiempo parcial<br>◯ En busca de empleo<br>◯ Jubilado<br>◯ Ama de casa<br>◯ Estudiante<br>◯ Permiso de maternidad o paternidad<br>◯ Permiso por enfermedad<br>◯ Desempleado por discapacidad<br>◯ Otra<br>◯ No sabe
Persona 11: ¿Se considera esta persona actualmente empleado por cuenta propia (contratista independiente, trabajador esporádico (gig), etc.)?	◯ Sí<br>◯ No<br>◯ No sabe

Persona 11: ¿Trabaja esta persona actualmente en alguno de los siguientes ambientes de alto riesgo de contagio de COVID-19?

- ☐ Ambiente de asistencia médica (hospital, clínica, centro de urgencias, etc.)
- ☐ Ambiente residencial denso (hogar de ancianos, otro centro de asistencia de larga duración)
- ☐ Prisión o cárcel
- ☐ Establecimiento de envasado de carne
- ☐ Establecimiento de envío o distribución
- ☐ Establecimiento minorista de alto volumen (tienda de provisiones, etc.)
- ☐ No sabe

Persona 11: ¿Le ofrece el empleador a esta persona alguno de los siguientes beneficios en su empleo principal actual?

- ☐ Permiso por enfermedad con goce de sueldo
- ☐ Vacaciones o permiso personal con goce de sueldo
- ☐ Seguro de salud
- ☐ Seguro de discapacidad
- ☐ Plan de jubilación
- ☐ Otro
- ☐ No sabe

((Seleccione todo lo que corresponda.))

Persona 11: En una escala de 0 (definitivamente no va a suceder) a 10 (definitivamente va a suceder), ¿qué tan probable es que esta persona pierda su empleo debido a la pandemia de COVID-19?

____

Persona 11: En una escala de 0 (definitivamente no va a suceder) a 10 (definitivamente va a suceder), ¿qué tan probable es que a esta persona se le asignen menos horas de trabajo debido a la pandemia de COVID-19?

____

	Todo el tiempo (100%)	la mayor parte del tiempo (75%)	La mitad del tiempo (50%)	Menos de la mitad del tiempo (25%)	Nunca (0%)
Persona 11: Actualmente ¿con qué frecuencia se le exige a esta persona que trabaje fuera de su residencia?	○	○	○	○	○
Persona 11: Actualmente ¿con qué frecuencia se encuentra esta persona físicamente cerca de sus compañeros de trabajo mientras trabaja fuera de su residencia?	○	○	○	○	○
Persona 11: Actualmente ¿con qué frecuencia se encuentra esta persona físicamente cerca de sus clientes mientras trabaja fuera de su residencia?	○	○	○	○	○

Persona 11: ¿Piensa esta persona vacunarse contra la COVID-19 cuando se ofrezca una vacuna?

- ○ Sí
- ○ No
- ○ No sabe

Persona 11: En las últimas dos semanas, ¿ha tenido esta persona algún síntoma de COVID-19 (tos, fiebre, dificultad para respirar, fatiga, dolores de cuerpo, diarrea, goteo nasal o pérdida del sentido del olfato o del gusto)?	◯ Sí<br>◯ No<br>◯ No sabe
Persona 11: ¿Cuándo le comenzaron los síntomas de COVID-19 a esta persona?	________________________
Persona 11: En vista de sus síntomas, ¿le preocupa a esta persona la posibilidad de tener COVID-19?	◯ Sí<br>◯ No<br>◯ No sabe
Persona 11: ¿Fue esta persona objeto de prejuicio o discriminación debido a sus síntomas?	◯ Sí<br>◯ No<br>◯ No sabe
Persona 11: ¿Qué hizo esta persona en vista de sus síntomas?	☐ Nada<br>☐ Tomó medicamentos sin receta (ibuprofeno, acetaminofén, etc.)<br>☐ Consultó por teléfono a un proveedor de asistencia médica<br>☐ Fue al consultorio de un proveedor de asistencia médica<br>☐ Fue a una clínica o una farmacia minorista<br>☐ Fue a un centro de urgencias (FASTMed, etc.)<br>☐ Fue a la sala de emergencias<br>☐ Fue ingresado al hospital<br>☐ Otra cosa<br>☐ No sabe<br>((Seleccione todo lo que corresponda.))
Persona 11 ¿Qué otra cosa hizo esta persona en vista de sus síntomas?	________________________
Persona 11: ¿Le dijo un proveedor de asistencia médica a esta persona que era posible que tuviera COVID-19?	◯ Sí<br>◯ No<br>◯ No sabe
Persona 11: Si a esta persona le hicieron una prueba de COVID-19 en vista de sus síntomas, ¿cuál fue el resultado?	◯ Pendiente<br>◯ Positivo<br>◯ Negativo<br>◯ No concluyente<br>◯ No se hizo la prueba<br>◯ No sabe
Persona 11: ¿Cuántos días estuvo hospitalizada esta persona?	________________________
Persona 11: ¿Se le hicieron a esta persona las siguientes intervenciones durante su hospitalización?	☐ Oxígeno adicional por la nariz<br>☐ Tratamiento en la Unidad de Cuidados Intensivos (Intensive Care Unit, ICU)<br>☐ Ventilación mecánica (intubación o tubo de respiración)<br>☐ No sabe
Persona 11: ¿Ha vuelto esta persona a su salud normal?	◯ Sí<br>◯ No<br>◯ No sabe

Persona 11: ¿Cuáles de las siguientes medidas tomó esta persona para proteger a sus amigos y familiares después de que comenzaron sus síntomas?	☐ Ponerse mascarilla con mayor frecuencia<br>☐ Lavarse las manos con agua y jabón con mayor frecuencia<br>☐ Limpiarse las manos con desinfectante con mayor frecuencia<br>☐ Aislarse en su casa con mayor frecuencia<br>☐ Quedarse en casa con mayor frecuencia<br>☐ Ponerse guantes desechables con mayor frecuencia<br>☐ No sabe

Por cada persona adicional en su hogar, proporcione la siguiente informacion.

Persona 12: ¿Cuál es su relación con esta persona?	◯ Pareja o cónyuge<br>◯ Hijo o hija<br>◯ Padre o madre<br>◯ Hermano o hermana<br>◯ Otra familiar<br>◯ Proveedor de cuidado infantil u otros cuidados en casa<br>◯ Otra
Persona 12: Especifique su relación con esta persona.	__________________________________
Persona 12: ¿Qué edad tiene esta persona?	__________________________________<br>((Especifique la edad en años))
Persona 12: ¿Cuál es el sexo de esta persona?	◯ Femenino<br>◯ Masculino
Persona 12: ¿Cuál es la raza de esta persona?	☐ Indio americano o nativo de Alaska<br>☐ Asiático<br>☐ Negro o afroestadounidense<br>☐ Nativo de Hawái o de las islas del Pacífico<br>☐ Blanco<br>☐ Otro<br>☐ No sabe<br>((Seleccione todo lo que corresponda.))
Persona 12: ¿Cuál es la identidad étnica de esta persona?	◯ Hispano o latino<br>◯ Ni hispano ni latino<br>◯ Otra<br>◯ No sabe
Persona 12: ¿Cuál es el nivel de educación de esta persona?	◯ No tiene educación formal<br>◯ Kínder a 8° grado<br>◯ Estudios de educación secundaria<br>◯ Equivalencia de educación secundaria (GED)<br>◯ Diploma de educación secundaria<br>◯ Estudios de educación superior<br>◯ Título universitario<br>◯ Estudios de posgrado o más<br>◯ No sabe

Persona 12: ¿Cuál de las siguientes es la mejor descripción de la situación laboral actual de esta persona?	◯ Empleo a tiempo completo<br>◯ Empleo a tiempo parcial<br>◯ En busca de empleo<br>◯ Jubilado<br>◯ Ama de casa<br>◯ Estudiante<br>◯ Permiso de maternidad o paternidad<br>◯ Permiso por enfermedad<br>◯ Desempleado por discapacidad<br>◯ Otra<br>◯ No sabe
Persona 12: ¿Se considera esta persona actualmente empleado por cuenta propia (contratista independiente, trabajador esporádico (gig), etc.)?	◯ Sí<br>◯ No<br>◯ No sabe
Persona 12: ¿Trabaja esta persona actualmente en alguno de los siguientes ambientes de alto riesgo de contagio de COVID-19?	☐ Ambiente de asistencia médica (hospital, clínica, centro de urgencias, etc.)<br>☐ Ambiente residencial denso (hogar de ancianos, otro centro de asistencia de larga duración)<br>☐ Prisión o cárcel<br>☐ Establecimiento de envasado de carne<br>☐ Establecimiento de envío o distribución<br>☐ Establecimiento minorista de alto volumen (tienda de provisiones, etc.)<br>☐ No sabe
Persona 12: ¿Le ofrece el empleador a esta persona alguno de los siguientes beneficios en su empleo principal actual?	☐ Permiso por enfermedad con goce de sueldo<br>☐ Vacaciones o permiso personal con goce de sueldo<br>☐ Seguro de salud<br>☐ Seguro de discapacidad<br>☐ Plan de jubilación<br>☐ Otro<br>☐ No sabe<br>((Seleccione todo lo que corresponda.))
Persona 12: En una escala de 0 (definitivamente no va a suceder) a 10 (definitivamente va a suceder), ¿qué tan probable es que esta persona pierda su empleo debido a la pandemia de COVID-19?	______________________
Persona 12: En una escala de 0 (definitivamente no va a suceder) a 10 (definitivamente va a suceder), ¿qué tan probable es que a esta persona se le asignen menos horas de trabajo debido a la pandemia de COVID-19?	______________________

	Todo el tiempo (100%)	la mayor parte del tiempo (75%)	La mitad del tiempo (50%)	Menos de la mitad del tiempo (25%)	Nunca (0%)
Persona 12: Actualmente ¿con qué frecuencia se le exige a esta persona que trabaje fuera de su residencia?	◯	◯	◯	◯	◯

Persona 12: Actualmente ¿con qué frecuencia se encuentra esta persona físicamente cerca de sus compañeros de trabajo mientras trabaja fuera de su residencia?

◯ ◯ ◯ ◯ ◯

Persona 12: Actualmente ¿con qué frecuencia se encuentra esta persona físicamente cerca de sus clientes mientras trabaja fuera de su residencia?

◯ ◯ ◯ ◯ ◯

Persona 12: ¿Piensa esta persona vacunarse contra la COVID-19 cuando se ofrezca una vacuna?

◯ Sí
◯ No
◯ No sabe

Persona 12: En las últimas dos semanas, ¿ha tenido esta persona algún síntoma de COVID-19 (tos, fiebre, dificultad para respirar, fatiga, dolores de cuerpo, diarrea, goteo nasal o pérdida del sentido del olfato o del gusto)?

◯ Sí
◯ No
◯ No sabe

Persona 12: ¿Cuándo le comenzaron los síntomas de COVID-19 a esta persona?

____

Persona 12: En vista de sus síntomas, ¿le preocupa a esta persona la posibilidad de tener COVID-19?

◯ Sí
◯ No
◯ No sabe

Persona 12: ¿Fue esta persona objeto de prejuicio o discriminación debido a sus síntomas?

◯ Sí
◯ No
◯ No sabe

Persona 12: ¿Qué hizo esta persona en vista de sus síntomas?

☐ Nada
☐ Tomó medicamentos sin receta (ibuprofeno, acetaminofén, etc.)
☐ Consultó por teléfono a un proveedor de asistencia médica
☐ Fue al consultorio de un proveedor de asistencia médica
☐ Fue a una clínica o una farmacia minorista
☐ Fue a un centro de urgencias (FASTMed, etc.)
☐ Fue a la sala de emergencias
☐ Fue ingresado al hospital
☐ Otra cosa
☐ No sabe
((Seleccione todo lo que corresponda.))

Persona 12 ¿Qué otra cosa hizo esta persona en vista de sus síntomas?

____

Persona 12: ¿Le dijo un proveedor de asistencia médica a esta persona que era posible que tuviera COVID-19?

◯ Sí
◯ No
◯ No sabe

Persona 12: Si a esta persona le hicieron una prueba de COVID-19 en vista de sus síntomas, ¿cuál fue el resultado?	◯ Pendiente<br>◯ Positivo<br>◯ Negativo<br>◯ No concluyente<br>◯ No se hizo la prueba<br>◯ No sabe
Persona 12: ¿Cuántos días estuvo hospitalizada esta persona?	______________________
Persona 12: ¿Se le hicieron a esta persona las siguientes intervenciones durante su hospitalización?	☐ Oxígeno adicional por la nariz<br>☐ Tratamiento en la Unidad de Cuidados Intensivos (Intensive Care Unit, ICU)<br>☐ Ventilación mecánica (intubación o tubo de respiración)<br>☐ No sabe
Persona 12: ¿Ha vuelto esta persona a su salud normal?	◯ Sí<br>◯ No<br>◯ No sabe
Persona 12: ¿Cuáles de las siguientes medidas tomó esta persona para proteger a sus amigos y familiares después de que comenzaron sus síntomas?	☐ Ponerse mascarilla con mayor frecuencia<br>☐ Lavarse las manos con agua y jabón con mayor frecuencia<br>☐ Limpiarse las manos con desinfectante con mayor frecuencia<br>☐ Aislarse en su casa con mayor frecuencia<br>☐ Quedarse en casa con mayor frecuencia<br>☐ Ponerse guantes desechables con mayor frecuencia<br>☐ No sabe

Complete la siguiente informacion sobre su salud mental y bienestar

A su juicio, ¿qué tan grave es para usted personalmente la pandemia de COVID-19 en este momento?	◯ Muy grave<br>◯ Más o menos grave<br>◯ No demasiado grave<br>◯ Nada grave
A su juicio, ¿qué tan grave es actualmente la pandemia de COVID-19 para los integrantes de su comunidad?	◯ Muy grave<br>◯ Más o menos grave<br>◯ No demasiado grave<br>◯ Nada grave
A su juicio, ¿qué tan grave es actualmente la pandemia de COVID-19 para los habitantes de Estados Unidos?	◯ Muy grave<br>◯ Más o menos grave<br>◯ No demasiado grave<br>◯ Nada grave
A su juicio, ¿qué tan grave es actualmente la pandemia de COVID-19 para los habitantes de todo el mundo?	◯ Muy grave<br>◯ Más o menos grave<br>◯ No demasiado grave<br>◯ Nada grave

La pandemia de COVID-19 puede cuasar desafios por algunas personas sin importar a que esten infectados. Que tan preocupado/a esta usted sobre cada una de la siguiente?

	Nada	No mucho	Un poco	Mucho
Recibir la asistencia médica que necesita (incluyendo la asistencia de salud mental)	○	○	○	○
Tener un lugar para vivir	○	○	○	○
Poder de interactuar con otras personas	○	○	○	○
Conseguir comida, agua y otros suministros domésticos	○	○	○	○
Conseguir medicamentos	○	○	○	○
Tener transporte para llegar adonde tiene que ir	○	○	○	○
Cuidar de su familia y a sus amigos	○	○	○	○

Durante las ultimas 2 semanas, que tan seguido ha tenido molestias debido a los siguientes problemas?

	Nunca	Varios días	Más de la mitad de los días	Casi todos los días
Nerviosisimo o ansiedad	○	○	○	○
Incapacidad de dejar de preocuparse o controlar las preocupaciones	○	○	○	○
Exceso de preocupación por diferentes cosas	○	○	○	○
Dificultad para tranquilizarse	○	○	○	○
Tanta inquietud que le es difícil estarse quieto	○	○	○	○
Facilidad para molestarse o irritarse	○	○	○	○
Temor de que algo horrible podría pasar	○	○	○	○

¿Cuánta dificultad le han causado estos problemas para hacer su trabajo, ocuparse de las cosas de su casa o llevarse bien con los demás?

- ○ Nada de dificultad
- ○ Un poco de dificultad
- ○ Bastante dificultad
- ○ Mucha dificultad

Durante las ultimas 2 semanas, qu_ tan seguido ha tenido los siguentes sentimientos y comportamientos?

	Rara vez o nunca (menos de 1 día)	Pocas veces (1-2 días)	De vez en cuando (3-4 días)	La mayor parte del tiempo o todo el tiempo (5-7 días)
Me ha molestado lo que normalmente no me molesta.	◯	◯	◯	◯
No he tenido ganas de comer; he tenido poco apetito.	◯	◯	◯	◯
He sentido que no podía librarme de la tristeza, ni siquiera con la ayuda de mis familiares o amigos.	◯	◯	◯	◯
He sentido que yo soy tan bueno como otras personas.	◯	◯	◯	◯
Me ha costado concentrarme en lo que hacía.	◯	◯	◯	◯
Me he sentido deprimido.	◯	◯	◯	◯
He sentido que todo lo que hacía implicaba un gran esfuerzo.	◯	◯	◯	◯
He tenido esperanza para el futuro.	◯	◯	◯	◯
He pensado que mi vida es un fracaso.	◯	◯	◯	◯
He tenido miedo.	◯	◯	◯	◯
No he dormido bien.	◯	◯	◯	◯
He sido feliz.	◯	◯	◯	◯
He hablado menos de lo habitual.	◯	◯	◯	◯
Me he sentido solo.	◯	◯	◯	◯
Las personas han sido poco amistosas.	◯	◯	◯	◯
He disfrutado de la vida.	◯	◯	◯	◯
He tenido episodios de llanto.	◯	◯	◯	◯
Me he sentido triste.	◯	◯	◯	◯
He sentido que no les agrado a los demás.	◯	◯	◯	◯
No he podido "ponerme en marcha".	◯	◯	◯	◯

¿Está usted dispuesto a dar una muestra nasal con hisopo indolora y una muestra de sangre indolora para este estudio de COVID-19 en el condado de Chatham?

◯ Sí
◯ No

¿Cómo llenó esta encuesta?

- ◯ En una computadora (portátil o de escritorio)
- ◯ En un dispositivo móvil (tableta o teléfono celular)
- ◯ Por teléfono con un entrevistador
- ◯ Otra manera

Chatham County COVID-19 Cohort Study

Biweekly Survey

Bi-weekly Survey

This Chatham County COVID-19 Cohort Study is being conducted by researchers from the University of North Carolina at Chapel Hill Schools of Medicine and Public Health. The purpose of this study is to learn how the new coronavirus disease, called COVID-19, is spreading and how it affects health. The results may help uncover new ways to prevent COVID-19 in the future.

Please complete the survey below.

Thank you!

Would you prefer to take this survey in English or Spanish? / Preferie responder esta encuesta en ingles o espanol?

- ○ English / ingles
- ○ Spanish / espanol

Which of the following best fits your current work situation?

- ○ works full time
- ○ works part time
- ○ is looking for work/employment
- ○ retired
- ○ homemaker
- ○ student
- ○ on maternity/paternity leave
- ○ on illness/sick leave
- ○ on disability
- ○ other

Do you currently consider yourself self-employed (including as an independent contractor or gig-economy worker)?

- ○ yes
- ○ no
- ○ don't know

On a scale of 0 (definitely not going to happen) to 10 (definitely going to happen), how likely is it that you will lose your job because of the COVID-19 pandemic?

____

On a scale of 0 (definitely not going to happen) to 10 (definitely going to happen), how likely is it that you will receive fewer work hours at your job because of the COVID-19 pandemic?

____

On a scale of 0 (definitely not going to happen) to 10 (definitely going to happen), how likely do you think it is that your household will run out of money in the next 3 months?

____

---

	always (100%)	most of the time (75%)	half of the time (50%)	less than half of the time (25%)	never (0%)
How often are you required to work from outside of the home currently?	○	○	○	○	○

How regularly are you in close physical contact with co-workers during your work outside of the home currently?	○	○	○	○	○
How regularly are you in close physical contact with clients during your work outside of the home currently?	○	○	○	○	○
How often do you have access to disposable gloves during your work outside of the home currently?	○	○	○	○	○
How often do you have access to a face mask during your work outside of the home currently?	○	○	○	○	○
How often do you use disposable gloves during your work outside of the home currently?	○	○	○	○	○
How often do you use a face mask during your work outside of the home currently?	○	○	○	○	○
How often do you wash your hands with soap and water during your work outside of the home currently?	○	○	○	○	○
How often do you sanitize your hands with hand sanitizer during your work outside of the home currently?	○	○	○	○	○
How worried are you that you will be exposed to COVID-19 during your work outside of the home currently?	○	○	○	○	○

Do you currently work in any of the following high-risk settings for COVID-19 transmission?

- ☐ healthcare setting (hospital, clinic, urgent care, etc.)
- ☐ dense residential setting (nursing home, other long-term care facility)
- ☐ prison or jail
- ☐ meatpacking facility
- ☐ shipping or distribution facility
- ☐ high-volume retail facility (grocery store, etc.)

Are you covered by any type of medical or health insurance (including private insurance, insurance your purchased, Medicare, Medicaid, or any other health insurance program)?

- ○ yes
- ○ no
- ○ don't know

What is the primary health insurance coverage that you have?

- ◯ Private health insurance through a job or school
- ◯ Insurance purchased through a state or federal health insurance exchange, such as healthcare.gov
- ◯ Insurance purchased directly through a health plan or insurance company
- ◯ Medicare
- ◯ Medi-Gap
- ◯ Medicaid
- ◯ Military health care (TRICARE, VA, CHAMP-VA, etc.)
- ◯ Indian Health Service
- ◯ Other

(Select one (your primary insurance).)

Please specify your other source of health insurance

____

Are you currently pregnant?

- ◯ Yes
- ◯ No

	excellent	very good	good	fair	poor
In general, how would you rate your health over the last two weeks?	◯	◯	◯	◯	◯

How often have you done the following things to protect yourself from infection during the last two weeks?

	always (100%)	most of the time (75%)	half of the time (50%)	less than half of the time (25%)	never (0%)
Worn a face mask	◯	◯	◯	◯	◯
Washed hands and/or used sanitizer frequently	◯	◯	◯	◯	◯
Stayed at least 6 feet away from others	◯	◯	◯	◯	◯
Avoided large gatherings, public spaces, or crowds	◯	◯	◯	◯	◯
Avoided contact with people who could be high risk	◯	◯	◯	◯	◯
Avoided food from restaurants, including takeout	◯	◯	◯	◯	◯
Worked or studied at home instead of going into an office/classroom	◯	◯	◯	◯	◯
Avoided shaking hands or touching people	◯	◯	◯	◯	◯
Stayed home when you were sick	◯	◯	◯	◯	◯
Wiped down surfaces with disinfectant	◯	◯	◯	◯	◯

Cancelled or postponed planned travel for work	○	○	○	○	○
Cancelled or postponed travel for pleasure	○	○	○	○	○
Cancelled or postponed personal or social activities	○	○	○	○	○
Cancelled a doctor's appointment	○	○	○	○	○
Stockpiled food or water	○	○	○	○	○
Followed government guidelines or rules to shelter in place (staying at home, limiting contacts with other people, etc.)	○	○	○	○	○

During the last two weeks, have you experienced any of the following symptoms?

	yes	no
Fever (measured by thermometer or self-diagnosed)	○	○
Cough (new or worsening)	○	○
Shortness of breath (new or worsening)	○	○
Fatigue (new tiredness doing normal activities)	○	○
Body aches	○	○
Headache	○	○
Diarrhea	○	○
Sore throat	○	○
Itchy, pink, or painful eyes	○	○
Runny nose or congestion	○	○
Changes in your sense of smell or taste	○	○
New rash	○	○
Repeated shaking with chills	○	○

When did the symptoms reported above first start?

____

Did you experience any bias or discrimination due to the symptoms you reported?

○ yes
○ no
○ don't know

Which of the following did you do to protect your friends and family after your symptoms began?

☐ wore a mask more frequently
☐ washed your hands with soap and water more frequently
☐ used hand sanitizer more frequently
☐ isolated yourself in your home more frequently
☐ stayed home more frequently
☐ wore disposable gloves more frequently

What did you do in response to the symptoms reported above?	☐ nothing<br>☐ took over the counter medication (ibuprofen, acetaminophen, etc.)<br>☐ communicated with a health care provider over the phone<br>☐ visited a health care provider's office<br>☐ visited a retail clinic or pharmacy<br>☐ visited urgent care (FASTMed, etc.)<br>☐ visited the emergency room<br>☐ was admitted to the hospital<br>☐ other<br>(Select all that apply.)
Please specify what other action you took in response to your symptoms.	______________________
If you were able to talk with a health care provider, were you told that you may have COVID-19?	◯ yes<br>◯ no<br>◯ don't know
If you received a COVID-19 test due to the symptoms you reported, what was the result?	◯ pending<br>◯ positive<br>◯ negative<br>◯ inconclusive<br>◯ did not receive a test
How many days were you admitted to the hospital?	______________________
Did you receive the following interventions during your hospital admission?	☐ extra oxygen in your nose<br>☐ treatment in the intensive care unit (ICU)<br>☐ mechanical ventilation (intubation or a breathing tube)
Have you returned to your normal health at this time?	◯ yes<br>◯ no<br>◯ don't know
Have you ever been, or are currently, in a COVID vaccine trial?	◯ Yes<br>◯ No
Where was/is the primary site for the COVID vaccine trial? (For example: UNC, Duke, a specific hospital?)	______________________
Have you received a COVID vaccine outside of a clinical trial?	◯ Yes<br>◯ No
Where did you receive the COVID vaccine?	◯ Doctors Office<br>◯ Work/Employment<br>◯ Retail (e.g. Walgreens, CVS)<br>◯ Vaccine site<br>◯ Other
Please specify where you received the COVID vaccine.	______________________
Please specify the city/town in NC of the vaccine site:	______________________

Please specify who was the hosting organization/institution of the vaccine site:	__________________________________
Which COVID vaccine did you receive?	◯ Pfizer<br>◯ Moderna<br>◯ AstraZeneca<br>◯ Novavax<br>◯ Johnson & Johnson<br>◯ Other<br>◯ Unsure/Unknown
Please specify which other COVID vaccine you received.	__________________________________
How many doses of the COVID vaccine have you received?	◯ 1<br>◯ 2
Which day did you receive the first dose of the vaccine?	__________________________________
Which day did you receive the second dose of the vaccine?	__________________________________

Did you experience any of the following side effects after vaccination?

		No	Mild (you notice symptoms, but they aren't a problem)	Moderate (symptoms limit your normal daily activities)	Severe (symptoms make normal daily activities difficult or impossible)
1	pain at or around the injection site	◯	◯	◯	◯
2	redness at or around the injection site	◯	◯	◯	◯
3	swelling at or around the injection site	◯	◯	◯	◯
4	rash at or around the injection site	◯	◯	◯	◯
5	headache	◯	◯	◯	◯
6	fatigue	◯	◯	◯	◯
7	fever (temperature >100.4°F or >38°C)	◯	◯	◯	◯
8	chills	◯	◯	◯	◯
9	joint pain	◯	◯	◯	◯
10	muscle pain	◯	◯	◯	◯
11	nausea	◯	◯	◯	◯

How long did these side effects last?	◯ Less than 12 hours<br>◯ 12 to 24 hours<br>◯ more than 24 hours
Did you take any medication for these side effects?	◯ Yes<br>◯ No

What medication(s) did you take for the side effects?

____
(Please list all medications.)

Did you consult a physician or other health care provider for the side effects?
- ◯ Yes
- ◯ No

How did you experience the side effects after the second dose of the vaccination as compared to those after the first dose of the vaccination?
- ◯ More severe
- ◯ Less severe
- ◯ Equally severe
- ◯ Not applicable/Haven't received second dose yet

Please provide the following information about your household.

What is your permanent address?

____

How long have you lived at this address?
- ◯ 0-3 years
- ◯ 4-6 years
- ◯ 7-10 years
- ◯ more than 10 years

How many additional people (not including yourself) live or spend a significant amount of time (greater than 40 hours a week) in this household?
- ◯ 0
- ◯ 1
- ◯ 2
- ◯ 3
- ◯ 4
- ◯ 5
- ◯ 6
- ◯ 7
- ◯ 8
- ◯ 9
- ◯ 10
- ◯ 11
- ◯ 12

How many of the people in your household are below the age of 18?
- ◯ 0
- ◯ 1
- ◯ 2
- ◯ 3
- ◯ 4
- ◯ 5
- ◯ 6
- ◯ 7
- ◯ 8
- ◯ 9
- ◯ 10
- ◯ 11
- ◯ 12

For each additional person in the your household, please provide the following information.

Person 1: What is your relationship to this person?

◯ partner or spouse
◯ child
◯ parent
◯ sibling
◯ other family member
◯ in-home childcare provider or other caregiver
◯ other

Person 1: Please specify your relationship with this person.

____

Person 1: What is this person's age?

____
(Please specify their age in years)

Person 1: What is this person's sex?

◯ Female
◯ Male
◯ Other

Person 1: What is this person's race?

☐ American Indian or Alaska Native
☐ Asian
☐ Black or African American
☐ Native Hawaiian or Pacific Islander
☐ White
☐ Other
☐ don't know
(Select all that apply.)

Person 1: What is this person's ethnicity?

◯ Hispanic or Latino
◯ Not Hispanic or Latino
◯ Other
◯ don't know

Person 1: What is the highest level of education or schooling this person has completed?

◯ never attended school
◯ kindergarten - 8th grade
◯ some high school
◯ high school equivalency (GED)
◯ high school graduate
◯ some college
◯ college graduate
◯ graduate school or more
◯ don't know

Person 1: Which of the following best fit this person's current work situation?

◯ works full time
◯ works part time
◯ is looking for work/employment
◯ retired
◯ homemaker
◯ student
◯ on maternity/paternity leave
◯ on illness/sick leave
◯ on disability
◯ other
◯ don't know

Person 1: Does this person currently consider themselves self-employed (including as an independent contractor or gig-economy worker)?

◯ yes
◯ no
◯ don't know

Person 1: Does this person currently work in any of the following high-risk settings for COVID-19 transmission?	☐ healthcare setting (hospital, clinic, urgent care, etc.)<br>☐ dense residential setting (nursing home, other long-term care facility)<br>☐ prison or jail<br>☐ meatpacking facility<br>☐ shipping or distribution facility<br>☐ high-volume retail facility (grocery store, etc.)<br>☐ don't know
Person 1: Does this person's employer offer them any of the following benefits at their current main job?	☐ paid sick leave<br>☐ paid vacation/personal leave<br>☐ health insurance<br>☐ disability insurance<br>☐ retirement plan<br>☐ other<br>☐ don't know<br>(Select all that apply.)
Person 1: On a scale of 0 (definitely not going to happen) to 10 (definitely going to happen), how likely is it that this person will lose their job because of the COVID-19 pandemic?	________________________
Person 1: On a scale of 0 (definitely not going to happen) to 10 (definitely going to happen), how likely is it that this person will receive fewer work hours at their job because of the COVID-19 pandemic?	________________________

	always (100%)	most of the time (75%)	half of the time (50%)	less than half of the time (25%)	never (0%)	don't know
Person 1: How often is this person required to work from outside of the home currently?	○	○	○	○	○	○
Person 1: How regularly is this person in close physical contact with co-workers during their work outside of the home currently?	○	○	○	○	○	○
Person 1: How regularly is this person in close physical contact with clients during their work outside of the home currently?	○	○	○	○	○	○

Person 1: Does this person plan to get a vaccine for COVID-19 when one becomes available?	○ yes<br>○ no<br>○ don't know
Person 1: Has this person had any symptoms (cough, fever, difficulty breathing, fatigue, body aches, diarrhea, runny nose, loss of smell or taste) consistent with COVID-19 in the last two weeks?	○ yes<br>○ no<br>○ don't know
Person 1: When did this person's symptoms begin?	________________________

Person 1: Is this person worried that they may have had COVID-19 because of their symptoms?	◯ yes<br>◯ no<br>◯ don't know
Person 1: Did this person experience any bias or discrimination because of their symptoms?	◯ yes<br>◯ no<br>◯ don't know
Person 1: What did this person do in response to their symptoms?	☐ nothing<br>☐ took over the counter medication (ibuprofen, acetaminophen, etc.)<br>☐ communicated with a health care provider over the phone<br>☐ visited a health care provider's office<br>☐ visited a retail clinic or pharmacy<br>☐ visited urgent care (FASTMed, etc.)<br>☐ visited the emergency room<br>☐ was admitted to the hospital<br>☐ other<br>☐ don't know<br>(Select all that apply.)
Person 1: Please specify what other action this person took in response to their symptoms.	______________________
Person 1: Did a health care provider tell this person that they may have COVID-19?	◯ yes<br>◯ no<br>◯ don't know
Person 1: If this person received a COVID-19 test due to their symptoms, what was the result?	◯ pending<br>◯ positive<br>◯ negative<br>◯ inconclusive<br>◯ did not receive a test<br>◯ don't know
Person 1: How many days was this person admitted to the hospital?	______________________
Person 1: Did this person receive any of the following interventions during their hospital admission?	☐ extra oxygen in your nose<br>☐ treatment in the intensive care unit (ICU)<br>☐ mechanical ventilation (intubation or a breathing tube)<br>☐ don't know
Person 1: Has this person returned to their normal health at this time?	◯ yes<br>◯ no<br>◯ don't know
Person 1: Which of the following did this person do to protect their friends and family after their symptoms began?	☐ wore a mask more frequently<br>☐ washed your hands with soap and water more frequently<br>☐ used hand sanitizer more frequently<br>☐ isolated yourself in your home more frequently<br>☐ stayed home more frequently<br>☐ wore disposable gloves more frequently<br>☐ don't know

For each additional person in the your household, please provide the following information.

Person 2: What is your relationship to this person?	◯ partner or spouse<br>◯ child<br>◯ parent<br>◯ sibling<br>◯ other family member<br>◯ in-home childcare provider or other caregiver<br>◯ other
Person 2: Please specify your relationship with this person.	______________________________
Person 2: What is this person's age?	______________________________<br>(Please specify their age in years)
Person 2: What is this person's sex?	◯ Female<br>◯ Male<br>◯ Other
Person 2: What is this person's race?	☐ American Indian or Alaska Native<br>☐ Asian<br>☐ Black or African American<br>☐ Native Hawaiian or Pacific Islander<br>☐ White<br>☐ Other<br>☐ don't know<br>(Select all that apply.)
Person 2: What is this person's ethnicity?	◯ Hispanic or Latino<br>◯ Not Hispanic or Latino<br>◯ Other<br>◯ don't know
Person 2: What is the highest level of education or schooling this person has completed?	◯ never attended school<br>◯ kindergarten - 8th grade<br>◯ some high school<br>◯ high school equivalency (GED)<br>◯ high school graduate<br>◯ some college<br>◯ college graduate<br>◯ graduate school or more<br>◯ don't know
Person 2: Which of the following best fit this person's current work situation?	◯ works full time<br>◯ works part time<br>◯ is looking for work/employment<br>◯ retired<br>◯ homemaker<br>◯ student<br>◯ on maternity/paternity leave<br>◯ on illness/sick leave<br>◯ on disability<br>◯ other<br>◯ don't know
Person 2: Does this person currently consider themselves self-employed (including as an independent contractor or gig-economy worker)?	◯ yes<br>◯ no<br>◯ don't know

Person 2: Does this person currently work in any of the following high-risk settings for COVID-19 transmission?	☐ healthcare setting (hospital, clinic, urgent care, etc.)<br>☐ dense residential setting (nursing home, other long-term care facility)<br>☐ prison or jail<br>☐ meatpacking facility<br>☐ shipping or distribution facility<br>☐ high-volume retail facility (grocery store, etc.)<br>☐ don't know
Person 2: Does this person's employer offer them any of the following benefits at their current main job?	☐ paid sick leave<br>☐ paid vacation/personal leave<br>☐ health insurance<br>☐ disability insurance<br>☐ retirement plan<br>☐ other<br>☐ don't know<br>(Select all that apply.)
Person 2: On a scale of 0 (definitely not going to happen) to 10 (definitely going to happen), how likely is it that this person will lose their job because of the COVID-19 pandemic?	______________________
Person 2: On a scale of 0 (definitely not going to happen) to 10 (definitely going to happen), how likely is it that this person will receive fewer work hours at their job because of the COVID-19 pandemic?	______________________

	always (100%)	most of the time (75%)	half of the time (50%)	less than half of the time (25%)	never (0%)	don't know
Person 2: How often is this person required to work from outside of the home currently?	○	○	○	○	○	○
Person 2: How regularly is this person in close physical contact with co-workers during their work outside of the home currently?	○	○	○	○	○	○
Person 2: How regularly is this person in close physical contact with clients during their work outside of the home currently?	○	○	○	○	○	○

Person 2: Does this person plan to get a vaccine for COVID-19 when one becomes available?	○ yes<br>○ no<br>○ don't know
Person 2: Has this person had any symptoms (cough, fever, difficulty breathing, fatigue, body aches, diarrhea, runny nose, loss of smell or taste) consistent with COVID-19 in the last two weeks?	○ yes<br>○ no<br>○ don't know
Person 2: When did this person's symptoms begin?	______________________

Person 2: Is this person worried that they may have had COVID-19 because of their symptoms?	◯ yes<br>◯ no<br>◯ don't know
Person 2: Did this person experience any bias or discrimination because of their symptoms?	◯ yes<br>◯ no<br>◯ don't know
Person 2: What did this person do in response to their symptoms?	☐ nothing<br>☐ took over the counter medication (ibuprofen, acetaminophen, etc.)<br>☐ communicated with a health care provider over the phone<br>☐ visited a health care provider's office<br>☐ visited a retail clinic or pharmacy<br>☐ visited urgent care (FASTMed, etc.)<br>☐ visited the emergency room<br>☐ was admitted to the hospital<br>☐ other<br>☐ don't know<br>(Select all that apply.)
Person 2: Please specify what other action this person took in response to their symptoms.	__________________________________
Person 2: Did a health care provider tell this person that they may have COVID-19?	◯ yes<br>◯ no<br>◯ don't know
Person 2: If this person received a COVID-19 test due to their symptoms, what was the result?	◯ pending<br>◯ positive<br>◯ negative<br>◯ inconclusive<br>◯ did not receive a test<br>◯ don't know
Person 2: How many days was this person admitted to the hospital?	__________________________________
Person 2: Did this person receive any of the following interventions during their hospital admission?	☐ extra oxygen in your nose<br>☐ treatment in the intensive care unit (ICU)<br>☐ mechanical ventilation (intubation or a breathing tube)<br>☐ don't know
Person 2: Has this person returned to their normal health at this time?	◯ yes<br>◯ no<br>◯ don't know
Person 2: Which of the following did this person do to protect their friends and family after their symptoms began?	☐ wore a mask more frequently<br>☐ washed your hands with soap and water more frequently<br>☐ used hand sanitizer more frequently<br>☐ isolated yourself in your home more frequently<br>☐ stayed home more frequently<br>☐ wore disposable gloves more frequently<br>☐ don't know

For each additional person in the your household, please provide the following information.

Person 3: What is your relationship to this person?

- ◯ partner or spouse
- ◯ child
- ◯ parent
- ◯ sibling
- ◯ other family member
- ◯ in-home childcare provider or other caregiver
- ◯ other

Person 3: Please specify your relationship with this person.

____

Person 3: What is this person's age?

____
(Please specify their age in years)

Person 3: What is this person's sex?

- ◯ Female
- ◯ Male
- ◯ Other

Person 3: What is this person's race?

- ☐ American Indian or Alaska Native
- ☐ Asian
- ☐ Black or African American
- ☐ Native Hawaiian or Pacific Islander
- ☐ White
- ☐ Other
- ☐ don't know

(Select all that apply.)

Person 3: What is this person's ethnicity?

- ◯ Hispanic or Latino
- ◯ Not Hispanic or Latino
- ◯ Other
- ◯ don't know

Person 3: What is the highest level of education or schooling this person has completed?

- ◯ never attended school
- ◯ kindergarten - 8th grade
- ◯ some high school
- ◯ high school equivalency (GED)
- ◯ high school graduate
- ◯ some college
- ◯ college graduate
- ◯ graduate school or more
- ◯ don't know

Person 3: Which of the following best fit this person's current work situation?

- ◯ works full time
- ◯ works part time
- ◯ is looking for work/employment
- ◯ retired
- ◯ homemaker
- ◯ student
- ◯ on maternity/paternity leave
- ◯ on illness/sick leave
- ◯ on disability
- ◯ other
- ◯ don't know

Person 3: Does this person currently consider themselves self-employed (including as an independent contractor or gig-economy worker)?

- ◯ yes
- ◯ no
- ◯ don't know

Person 3: Does this person currently work in any of the following high-risk settings for COVID-19 transmission?	☐ healthcare setting (hospital, clinic, urgent care, etc.)<br>☐ dense residential setting (nursing home, other long-term care facility)<br>☐ prison or jail<br>☐ meatpacking facility<br>☐ shipping or distribution facility<br>☐ high-volume retail facility (grocery store, etc.)<br>☐ don't know
Person 3: Does this person's employer offer them any of the following benefits at their current main job?	☐ paid sick leave<br>☐ paid vacation/personal leave<br>☐ health insurance<br>☐ disability insurance<br>☐ retirement plan<br>☐ other<br>☐ don't know<br>(Select all that apply.)
Person 3: On a scale of 0 (definitely not going to happen) to 10 (definitely going to happen), how likely is it that this person will lose their job because of the COVID-19 pandemic?	________________________
Person 3: On a scale of 0 (definitely not going to happen) to 10 (definitely going to happen), how likely is it that this person will receive fewer work hours at their job because of the COVID-19 pandemic?	________________________

	always (100%)	most of the time (75%)	half of the time (50%)	less than half of the time (25%)	never (0%)	don't know
Person 3: How often is this person required to work from outside of the home currently?	◯	◯	◯	◯	◯	◯
Person 3: How regularly is this person in close physical contact with co-workers during their work outside of the home currently?	◯	◯	◯	◯	◯	◯
Person 3: How regularly is this person in close physical contact with clients during their work outside of the home currently?	◯	◯	◯	◯	◯	◯

Person 3: Does this person plan to get a vaccine for COVID-19 when one becomes available?	◯ yes<br>◯ no<br>◯ don't know
Person 3: Has this person had any symptoms (cough, fever, difficulty breathing, fatigue, body aches, diarrhea, runny nose, loss of smell or taste) consistent with COVID-19 in the last two weeks?	◯ yes<br>◯ no<br>◯ don't know
Person 3: When did this person's symptoms begin?	________________________

Person 3: Is this person worried that they may have had COVID-19 because of their symptoms?	◯ yes<br>◯ no<br>◯ don't know
Person 3: Did this person experience any bias or discrimination because of their symptoms?	◯ yes<br>◯ no<br>◯ don't know
Person 3: What did this person do in response to their symptoms?	☐ nothing<br>☐ took over the counter medication (ibuprofen, acetaminophen, etc.)<br>☐ communicated with a health care provider over the phone<br>☐ visited a health care provider's office<br>☐ visited a retail clinic or pharmacy<br>☐ visited urgent care (FASTMed, etc.)<br>☐ visited the emergency room<br>☐ was admitted to the hospital<br>☐ other<br>☐ don't know<br>(Select all that apply.)
Person 3: Please specify what other action this person took in response to their symptoms.	__________________________________
Person 3: Did a health care provider tell this person that they may have COVID-19?	◯ yes<br>◯ no<br>◯ don't know
Person 3: If this person received a COVID-19 test due to their symptoms, what was the result?	◯ pending<br>◯ positive<br>◯ negative<br>◯ inconclusive<br>◯ did not receive a test<br>◯ don't know
Person 3: How many days was this person admitted to the hospital?	__________________________________
Person 3: Did this person receive any of the following interventions during their hospital admission?	☐ extra oxygen in your nose<br>☐ treatment in the intensive care unit (ICU)<br>☐ mechanical ventilation (intubation or a breathing tube)<br>☐ don't know
Person 3: Has this person returned to their normal health at this time?	◯ yes<br>◯ no<br>◯ don't know
Person 3: Which of the following did this person do to protect their friends and family after their symptoms began?	☐ wore a mask more frequently<br>☐ washed your hands with soap and water more frequently<br>☐ used hand sanitizer more frequently<br>☐ isolated yourself in your home more frequently<br>☐ stayed home more frequently<br>☐ wore disposable gloves more frequently<br>☐ don't know

For each additional person in the your household, please provide the following information.

Question	Response
Person 4: What is your relationship to this person?	◯ partner or spouse<br>◯ child<br>◯ parent<br>◯ sibling<br>◯ other family member<br>◯ in-home childcare provider or other caregiver<br>◯ other
Person 4: Please specify your relationship with this person.	__________________________________
Person 4: What is this person's age?	__________________________________<br>(Please specify their age in years)
Person 4: What is this person's sex?	◯ Female<br>◯ Male<br>◯ Other
Person 4: What is this person's race?	☐ American Indian or Alaska Native<br>☐ Asian<br>☐ Black or African American<br>☐ Native Hawaiian or Pacific Islander<br>☐ White<br>☐ Other<br>☐ don't know<br>(Select all that apply.)
Person 4: What is this person's ethnicity?	◯ Hispanic or Latino<br>◯ Not Hispanic or Latino<br>◯ Other<br>◯ don't know
Person 4: What is the highest level of education or schooling this person has completed?	◯ never attended school<br>◯ kindergarten - 8th grade<br>◯ some high school<br>◯ high school equivalency (GED)<br>◯ high school graduate<br>◯ some college<br>◯ college graduate<br>◯ graduate school or more<br>◯ don't know
Person 4: Which of the following best fit this person's current work situation?	◯ works full time<br>◯ works part time<br>◯ is looking for work/employment<br>◯ retired<br>◯ homemaker<br>◯ student<br>◯ on maternity/paternity leave<br>◯ on illness/sick leave<br>◯ on disability<br>◯ other<br>◯ don't know
Person 4: Does this person currently consider themselves self-employed (including as an independent contractor or gig-economy worker)?	◯ yes<br>◯ no<br>◯ don't know

Person 4: Does this person currently work in any of the following high-risk settings for COVID-19 transmission?	☐ healthcare setting (hospital, clinic, urgent care, etc.)<br>☐ dense residential setting (nursing home, other long-term care facility)<br>☐ prison or jail<br>☐ meatpacking facility<br>☐ shipping or distribution facility<br>☐ high-volume retail facility (grocery store, etc.)<br>☐ don't know
Person 4: Does this person's employer offer them any of the following benefits at their current main job?	☐ paid sick leave<br>☐ paid vacation/personal leave<br>☐ health insurance<br>☐ disability insurance<br>☐ retirement plan<br>☐ other<br>☐ don't know<br>(Select all that apply.)
Person 4: On a scale of 0 (definitely not going to happen) to 10 (definitely going to happen), how likely is it that this person will lose their job because of the COVID-19 pandemic?	______________________
Person 4: On a scale of 0 (definitely not going to happen) to 10 (definitely going to happen), how likely is it that this person will receive fewer work hours at their job because of the COVID-19 pandemic?	______________________

	always (100%)	most of the time (75%)	half of the time (50%)	less than half of the time (25%)	never (0%)	don't know
Person 4: How often is this person required to work from outside of the home currently?	○	○	○	○	○	○
Person 4: How regularly is this person in close physical contact with co-workers during their work outside of the home currently?	○	○	○	○	○	○
Person 4: How regularly is this person in close physical contact with clients during their work outside of the home currently?	○	○	○	○	○	○

Person 4: Does this person plan to get a vaccine for COVID-19 when one becomes available?	○ yes<br>○ no<br>○ don't know
Person 4: Has this person had any symptoms (cough, fever, difficulty breathing, fatigue, body aches, diarrhea, runny nose, loss of smell or taste) consistent with COVID-19 in the last two weeks?	○ yes<br>○ no<br>○ don't know
Person 4: When did this person's symptoms begin?	______________________

Person 4: Is this person worried that they may have had COVID-19 because of their symptoms?	◯ yes<br>◯ no<br>◯ don't know
Person 4: Did this person experience any bias or discrimination because of their symptoms?	◯ yes<br>◯ no<br>◯ don't know
Person 4: What did this person do in response to their symptoms?	☐ nothing<br>☐ took over the counter medication (ibuprofen, acetaminophen, etc.)<br>☐ communicated with a health care provider over the phone<br>☐ visited a health care provider's office<br>☐ visited a retail clinic or pharmacy<br>☐ visited urgent care (FASTMed, etc.)<br>☐ visited the emergency room<br>☐ was admitted to the hospital<br>☐ other<br>☐ don't know<br>(Select all that apply.)
Person 4: Please specify what other action this person took in response to their symptoms.	__________________________________
Person 4: Did a health care provider tell this person that they may have COVID-19?	◯ yes<br>◯ no<br>◯ don't know
Person 4: If this person received a COVID-19 test due to their symptoms, what was the result?	◯ pending<br>◯ positive<br>◯ negative<br>◯ inconclusive<br>◯ did not receive a test<br>◯ don't know
Person 4: How many days was this person admitted to the hospital?	__________________________________
Person 4: Did this person receive any of the following interventions during their hospital admission?	☐ extra oxygen in your nose<br>☐ treatment in the intensive care unit (ICU)<br>☐ mechanical ventilation (intubation or a breathing tube)<br>☐ don't know
Person 4: Has this person returned to their normal health at this time?	◯ yes<br>◯ no<br>◯ don't know
Person 4: Which of the following did this person do to protect their friends and family after their symptoms began?	☐ wore a mask more frequently<br>☐ washed your hands with soap and water more frequently<br>☐ used hand sanitizer more frequently<br>☐ isolated yourself in your home more frequently<br>☐ stayed home more frequently<br>☐ wore disposable gloves more frequently<br>☐ don't know

For each additional person in the your household, please provide the following information.

Person 5: What is your relationship to this person?	◯ partner or spouse<br>◯ child<br>◯ parent<br>◯ sibling<br>◯ other family member<br>◯ in-home childcare provider or other caregiver<br>◯ other
Person 5: Please specify your relationship with this person.	__________________________________
Person 5: What is this person's age?	__________________________________<br>(Please specify their age in years)
Person 5: What is this person's sex?	◯ Female<br>◯ Male<br>◯ Other
Person 5: What is this person's race?	☐ American Indian or Alaska Native<br>☐ Asian<br>☐ Black or African American<br>☐ Native Hawaiian or Pacific Islander<br>☐ White<br>☐ Other<br>☐ don't know<br>(Select all that apply.)
Person 5: What is this person's ethnicity?	◯ Hispanic or Latino<br>◯ Not Hispanic or Latino<br>◯ Other<br>◯ don't know
Person 5: What is the highest level of education or schooling this person has completed?	◯ never attended school<br>◯ kindergarten - 8th grade<br>◯ some high school<br>◯ high school equivalency (GED)<br>◯ high school graduate<br>◯ some college<br>◯ college graduate<br>◯ graduate school or more<br>◯ don't know
Person 5: Which of the following best fit this person's current work situation?	◯ works full time<br>◯ works part time<br>◯ is looking for work/employment<br>◯ retired<br>◯ homemaker<br>◯ student<br>◯ on maternity/paternity leave<br>◯ on illness/sick leave<br>◯ on disability<br>◯ other<br>◯ don't know
Person 5: Does this person currently consider themselves self-employed (including as an independent contractor or gig-economy worker)?	◯ yes<br>◯ no<br>◯ don't know

Person 5: Does this person currently work in any of the following high-risk settings for COVID-19 transmission?	☐ healthcare setting (hospital, clinic, urgent care, etc.)<br>☐ dense residential setting (nursing home, other long-term care facility)<br>☐ prison or jail<br>☐ meatpacking facility<br>☐ shipping or distribution facility<br>☐ high-volume retail facility (grocery store, etc.)<br>☐ don't know
Person 5: Does this person's employer offer them any of the following benefits at their current main job?	☐ paid sick leave<br>☐ paid vacation/personal leave<br>☐ health insurance<br>☐ disability insurance<br>☐ retirement plan<br>☐ other<br>☐ don't know<br>(Select all that apply.)
Person 5: On a scale of 0 (definitely not going to happen) to 10 (definitely going to happen), how likely is it that this person will lose their job because of the COVID-19 pandemic?	______________________
Person 5: On a scale of 0 (definitely not going to happen) to 10 (definitely going to happen), how likely is it that this person will receive fewer work hours at their job because of the COVID-19 pandemic?	______________________

	always (100%)	most of the time (75%)	half of the time (50%)	less than half of the time (25%)	never (0%)	don't know
Person 5: How often is this person required to work from outside of the home currently?	◯	◯	◯	◯	◯	◯
Person 5: How regularly is this person in close physical contact with co-workers during their work outside of the home currently?	◯	◯	◯	◯	◯	◯
Person 5: How regularly is this person in close physical contact with clients during their work outside of the home currently?	◯	◯	◯	◯	◯	◯

Person 5: Does this person plan to get a vaccine for COVID-19 when one becomes available?	◯ yes<br>◯ no<br>◯ don't know
Person 5: Has this person had any symptoms (cough, fever, difficulty breathing, fatigue, body aches, diarrhea, runny nose, loss of smell or taste) consistent with COVID-19 in the last two weeks?	◯ yes<br>◯ no<br>◯ don't know
Person 5: When did this person's symptoms begin?	______________________

Person 5: Is this person worried that they may have had COVID-19 because of their symptoms?	◯ yes<br>◯ no<br>◯ don't know
Person 5: Did this person experience any bias or discrimination because of their symptoms?	◯ yes<br>◯ no<br>◯ don't know
Person 5: What did this person do in response to their symptoms?	☐ nothing<br>☐ took over the counter medication (ibuprofen, acetaminophen, etc.)<br>☐ communicated with a health care provider over the phone<br>☐ visited a health care provider's office<br>☐ visited a retail clinic or pharmacy<br>☐ visited urgent care (FASTMed, etc.)<br>☐ visited the emergency room<br>☐ was admitted to the hospital<br>☐ other<br>☐ don't know<br>(Select all that apply.)
Person 5: Please specify what other action this person took in response to their symptoms.	__________________________
Person 5: Did a health care provider tell this person that they may have COVID-19?	◯ yes<br>◯ no<br>◯ don't know
Person 5: If this person received a COVID-19 test due to their symptoms, what was the result?	◯ pending<br>◯ positive<br>◯ negative<br>◯ inconclusive<br>◯ did not receive a test<br>◯ don't know
Person 5: How many days was this person admitted to the hospital?	__________________________
Person 5: Did this person receive any of the following interventions during their hospital admission?	☐ extra oxygen in your nose<br>☐ treatment in the intensive care unit (ICU)<br>☐ mechanical ventilation (intubation or a breathing tube)<br>☐ don't know
Person 5: Has this person returned to their normal health at this time?	◯ yes<br>◯ no<br>◯ don't know
Person 5: Which of the following did this person do to protect their friends and family after their symptoms began?	☐ wore a mask more frequently<br>☐ washed your hands with soap and water more frequently<br>☐ used hand sanitizer more frequently<br>☐ isolated yourself in your home more frequently<br>☐ stayed home more frequently<br>☐ wore disposable gloves more frequently<br>☐ don't know

For each additional person in the your household, please provide the following information.

Person 6: What is your relationship to this person?	◯ partner or spouse<br>◯ child<br>◯ parent<br>◯ sibling<br>◯ other family member<br>◯ in-home childcare provider or other caregiver<br>◯ other
Person 6: Please specify your relationship with this person.	__________________________________
Person 6: What is this person's age?	__________________________________<br>(Please specify their age in years)
Person 6: What is this person's sex?	◯ Female<br>◯ Male<br>◯ Other
Person 6: What is this person's race?	☐ American Indian or Alaska Native<br>☐ Asian<br>☐ Black or African American<br>☐ Native Hawaiian or Pacific Islander<br>☐ White<br>☐ Other<br>☐ don't know<br>(Select all that apply.)
Person 6: What is this person's ethnicity?	◯ Hispanic or Latino<br>◯ Not Hispanic or Latino<br>◯ Other<br>◯ don't know
Person 6: What is the highest level of education or schooling this person has completed?	◯ never attended school<br>◯ kindergarten - 8th grade<br>◯ some high school<br>◯ high school equivalency (GED)<br>◯ high school graduate<br>◯ some college<br>◯ college graduate<br>◯ graduate school or more<br>◯ don't know
Person 6: Which of the following best fit this person's current work situation?	◯ works full time<br>◯ works part time<br>◯ is looking for work/employment<br>◯ retired<br>◯ homemaker<br>◯ student<br>◯ on maternity/paternity leave<br>◯ on illness/sick leave<br>◯ on disability<br>◯ other<br>◯ don't know
Person 6: Does this person currently consider themselves self-employed (including as an independent contractor or gig-economy worker)?	◯ yes<br>◯ no<br>◯ don't know

Person 6: Does this person currently work in any of the following high-risk settings for COVID-19 transmission?	☐ healthcare setting (hospital, clinic, urgent care, etc.)<br>☐ dense residential setting (nursing home, other long-term care facility)<br>☐ prison or jail<br>☐ meatpacking facility<br>☐ shipping or distribution facility<br>☐ high-volume retail facility (grocery store, etc.)<br>☐ don't know
Person 6: Does this person's employer offer them any of the following benefits at their current main job?	☐ paid sick leave<br>☐ paid vacation/personal leave<br>☐ health insurance<br>☐ disability insurance<br>☐ retirement plan<br>☐ other<br>☐ don't know<br>(Select all that apply.)
Person 6: On a scale of 0 (definitely not going to happen) to 10 (definitely going to happen), how likely is it that this person will lose their job because of the COVID-19 pandemic?	______________________________
Person 6: On a scale of 0 (definitely not going to happen) to 10 (definitely going to happen), how likely is it that this person will receive fewer work hours at their job because of the COVID-19 pandemic?	______________________________

	always (100%)	most of the time (75%)	half of the time (50%)	less than half of the time (25%)	never (0%)	don't know
Person 6: How often is this person required to work from outside of the home currently?	○	○	○	○	○	○
Person 6: How regularly is this person in close physical contact with co-workers during their work outside of the home currently?	○	○	○	○	○	○
Person 6: How regularly is this person in close physical contact with clients during their work outside of the home currently?	○	○	○	○	○	○

Person 6: Does this person plan to get a vaccine for COVID-19 when one becomes available?	○ yes<br>○ no<br>○ don't know
Person 6: Has this person had any symptoms (cough, fever, difficulty breathing, fatigue, body aches, diarrhea, runny nose, loss of smell or taste) consistent with COVID-19 in the last two weeks?	○ yes<br>○ no<br>○ don't know
Person 6: When did this person's symptoms begin?	______________________________

Person 6: Is this person worried that they may have had COVID-19 because of their symptoms?	◯ yes<br>◯ no<br>◯ don't know
Person 6: Did this person experience any bias or discrimination because of their symptoms?	◯ yes<br>◯ no<br>◯ don't know
Person 6: What did this person do in response to their symptoms?	☐ nothing<br>☐ took over the counter medication (ibuprofen, acetaminophen, etc.)<br>☐ communicated with a health care provider over the phone<br>☐ visited a health care provider's office<br>☐ visited a retail clinic or pharmacy<br>☐ visited urgent care (FASTMed, etc.)<br>☐ visited the emergency room<br>☐ was admitted to the hospital<br>☐ other<br>☐ don't know<br>(Select all that apply.)
Person 6: Please specify what other action this person took in response to their symptoms.	______________________
Person 6: Did a health care provider tell this person that they may have COVID-19?	◯ yes<br>◯ no<br>◯ don't know
Person 6: If this person received a COVID-19 test due to their symptoms, what was the result?	◯ pending<br>◯ positive<br>◯ negative<br>◯ inconclusive<br>◯ did not receive a test<br>◯ don't know
Person 6: How many days was this person admitted to the hospital?	______________________
Person 6: Did this person receive any of the following interventions during their hospital admission?	☐ extra oxygen in your nose<br>☐ treatment in the intensive care unit (ICU)<br>☐ mechanical ventilation (intubation or a breathing tube)<br>☐ don't know
Person 6: Has this person returned to their normal health at this time?	◯ yes<br>◯ no<br>◯ don't know
Person 6: Which of the following did this person do to protect their friends and family after their symptoms began?	☐ wore a mask more frequently<br>☐ washed your hands with soap and water more frequently<br>☐ used hand sanitizer more frequently<br>☐ isolated yourself in your home more frequently<br>☐ stayed home more frequently<br>☐ wore disposable gloves more frequently<br>☐ don't know

For each additional person in the your household, please provide the following information.

Person 7: What is your relationship to this person?	◯ partner or spouse<br>◯ child<br>◯ parent<br>◯ sibling<br>◯ other family member<br>◯ in-home childcare provider or other caregiver<br>◯ other
Person 7: Please specify your relationship with this person.	__________________________
Person 7: What is this person's age?	__________________________<br>(Please specify their age in years)
Person 7: What is this person's sex?	◯ Female<br>◯ Male<br>◯ Other
Person 7: What is this person's race?	☐ American Indian or Alaska Native<br>☐ Asian<br>☐ Black or African American<br>☐ Native Hawaiian or Pacific Islander<br>☐ White<br>☐ Other<br>☐ don't know<br>(Select all that apply.)
Person 7: What is this person's ethnicity?	◯ Hispanic or Latino<br>◯ Not Hispanic or Latino<br>◯ Other<br>◯ don't know
Person 7: What is the highest level of education or schooling this person has completed?	◯ never attended school<br>◯ kindergarten - 8th grade<br>◯ some high school<br>◯ high school equivalency (GED)<br>◯ high school graduate<br>◯ some college<br>◯ college graduate<br>◯ graduate school or more<br>◯ don't know
Person 7: Which of the following best fit this person's current work situation?	◯ works full time<br>◯ works part time<br>◯ is looking for work/employment<br>◯ retired<br>◯ homemaker<br>◯ student<br>◯ on maternity/paternity leave<br>◯ on illness/sick leave<br>◯ on disability<br>◯ other<br>◯ don't know
Person 7: Does this person currently consider themselves self-employed (including as an independent contractor or gig-economy worker)?	◯ yes<br>◯ no<br>◯ don't know

Person 7: Does this person currently work in any of the following high-risk settings for COVID-19 transmission?

- ☐ healthcare setting (hospital, clinic, urgent care, etc.)
- ☐ dense residential setting (nursing home, other long-term care facility)
- ☐ prison or jail
- ☐ meatpacking facility
- ☐ shipping or distribution facility
- ☐ high-volume retail facility (grocery store, etc.)
- ☐ don't know

Person 7: Does this person's employer offer them any of the following benefits at their current main job?

- ☐ paid sick leave
- ☐ paid vacation/personal leave
- ☐ health insurance
- ☐ disability insurance
- ☐ retirement plan
- ☐ other
- ☐ don't know

(Select all that apply.)

Person 7: On a scale of 0 (definitely not going to happen) to 10 (definitely going to happen), how likely is it that this person will lose their job because of the COVID-19 pandemic?

____

Person 7: On a scale of 0 (definitely not going to happen) to 10 (definitely going to happen), how likely is it that this person will receive fewer work hours at their job because of the COVID-19 pandemic?

____

	always (100%)	most of the time (75%)	half of the time (50%)	less than half of the time (25%)	never (0%)	don't know
Person 7: How often is this person required to work from outside of the home currently?	○	○	○	○	○	○
Person 7: How regularly is this person in close physical contact with co-workers during their work outside of the home currently?	○	○	○	○	○	○
Person 7: How regularly is this person in close physical contact with clients during their work outside of the home currently?	○	○	○	○	○	○

Person 7: Does this person plan to get a vaccine for COVID-19 when one becomes available?

- ○ yes
- ○ no
- ○ don't know

Person 7: Has this person had any symptoms (cough, fever, difficulty breathing, fatigue, body aches, diarrhea, runny nose, loss of smell or taste) consistent with COVID-19 in the last two weeks?

- ○ yes
- ○ no
- ○ don't know

Person 7: When did this person's symptoms begin?

____

Person 7: Is this person worried that they may have had COVID-19 because of their symptoms?	◯ yes<br>◯ no<br>◯ don't know
Person 7: Did this person experience any bias or discrimination because of their symptoms?	◯ yes<br>◯ no<br>◯ don't know
Person 7: What did this person do in response to their symptoms?	☐ nothing<br>☐ took over the counter medication (ibuprofen, acetaminophen, etc.)<br>☐ communicated with a health care provider over the phone<br>☐ visited a health care provider's office<br>☐ visited a retail clinic or pharmacy<br>☐ visited urgent care (FASTMed, etc.)<br>☐ visited the emergency room<br>☐ was admitted to the hospital<br>☐ other<br>☐ don't know<br>(Select all that apply.)
Person 7: Please specify what other action this person took in response to their symptoms.	__________________________________
Person 7: Did a health care provider tell this person that they may have COVID-19?	◯ yes<br>◯ no<br>◯ don't know
Person 7: If this person received a COVID-19 test due to their symptoms, what was the result?	◯ pending<br>◯ positive<br>◯ negative<br>◯ inconclusive<br>◯ did not receive a test<br>◯ don't know
Person 7: How many days was this person admitted to the hospital?	__________________________________
Person 7: Did this person receive any of the following interventions during their hospital admission?	☐ extra oxygen in your nose<br>☐ treatment in the intensive care unit (ICU)<br>☐ mechanical ventilation (intubation or a breathing tube)<br>☐ don't know
Person 7: Has this person returned to their normal health at this time?	◯ yes<br>◯ no<br>◯ don't know
Person 7: Which of the following did this person do to protect their friends and family after their symptoms began?	☐ wore a mask more frequently<br>☐ washed your hands with soap and water more frequently<br>☐ used hand sanitizer more frequently<br>☐ isolated yourself in your home more frequently<br>☐ stayed home more frequently<br>☐ wore disposable gloves more frequently<br>☐ don't know

For each additional person in the your household, please provide the following information.

Person 8: What is your relationship to this person?	◯ partner or spouse<br>◯ child<br>◯ parent<br>◯ sibling<br>◯ other family member<br>◯ in-home childcare provider or other caregiver<br>◯ other
Person 8: Please specify your relationship with this person.	__________________________
Person 8: What is this person's age?	__________________________<br>(Please specify their age in years)
Person 8: What is this person's sex?	◯ Female<br>◯ Male<br>◯ Other
Person 8: What is this person's race?	☐ American Indian or Alaska Native<br>☐ Asian<br>☐ Black or African American<br>☐ Native Hawaiian or Pacific Islander<br>☐ White<br>☐ Other<br>☐ don't know<br>(Select all that apply.)
Person 8: What is this person's ethnicity?	◯ Hispanic or Latino<br>◯ Not Hispanic or Latino<br>◯ Other<br>◯ don't know
Person 8: What is the highest level of education or schooling this person has completed?	◯ never attended school<br>◯ kindergarten - 8th grade<br>◯ some high school<br>◯ high school equivalency (GED)<br>◯ high school graduate<br>◯ some college<br>◯ college graduate<br>◯ graduate school or more<br>◯ don't know
Person 8: Which of the following best fit this person's current work situation?	◯ works full time<br>◯ works part time<br>◯ is looking for work/employment<br>◯ retired<br>◯ homemaker<br>◯ student<br>◯ on maternity/paternity leave<br>◯ on illness/sick leave<br>◯ on disability<br>◯ other<br>◯ don't know
Person 8: Does this person currently consider themselves self-employed (including as an independent contractor or gig-economy worker)?	◯ yes<br>◯ no<br>◯ don't know

Person 8: Does this person currently work in any of the following high-risk settings for COVID-19 transmission?	☐ healthcare setting (hospital, clinic, urgent care, etc.)<br>☐ dense residential setting (nursing home, other long-term care facility)<br>☐ prison or jail<br>☐ meatpacking facility<br>☐ shipping or distribution facility<br>☐ high-volume retail facility (grocery store, etc.)<br>☐ don't know
Person 8: Does this person's employer offer them any of the following benefits at their current main job?	☐ paid sick leave<br>☐ paid vacation/personal leave<br>☐ health insurance<br>☐ disability insurance<br>☐ retirement plan<br>☐ other<br>☐ don't know<br>(Select all that apply.)
Person 8: On a scale of 0 (definitely not going to happen) to 10 (definitely going to happen), how likely is it that this person will lose their job because of the COVID-19 pandemic?	__________________________________
Person 8: On a scale of 0 (definitely not going to happen) to 10 (definitely going to happen), how likely is it that this person will receive fewer work hours at their job because of the COVID-19 pandemic?	__________________________________

	always (100%)	most of the time (75%)	half of the time (50%)	less than half of the time (25%)	never (0%)	don't know
Person 8: How often is this person required to work from outside of the home currently?	○	○	○	○	○	○
Person 8: How regularly is this person in close physical contact with co-workers during their work outside of the home currently?	○	○	○	○	○	○
Person 8: How regularly is this person in close physical contact with clients during their work outside of the home currently?	○	○	○	○	○	○

Person 8: Does this person plan to get a vaccine for COVID-19 when one becomes available?	○ yes<br>○ no<br>○ don't know
Person 8: Has this person had any symptoms (cough, fever, difficulty breathing, fatigue, body aches, diarrhea, runny nose, loss of smell or taste) consistent with COVID-19 in the last two weeks?	○ yes<br>○ no<br>○ don't know
Person 8: When did this person's symptoms begin?	__________________________________

Person 8: Is this person worried that they may have had COVID-19 because of their symptoms?	◯ yes<br>◯ no<br>◯ don't know
Person 8: Did this person experience any bias or discrimination because of their symptoms?	◯ yes<br>◯ no<br>◯ don't know
Person 8: What did this person do in response to their symptoms?	☐ nothing<br>☐ took over the counter medication (ibuprofen, acetaminophen, etc.)<br>☐ communicated with a health care provider over the phone<br>☐ visited a health care provider's office<br>☐ visited a retail clinic or pharmacy<br>☐ visited urgent care (FASTMed, etc.)<br>☐ visited the emergency room<br>☐ was admitted to the hospital<br>☐ other<br>☐ don't know<br>(Select all that apply.)
Person 8: Please specify what other action this person took in response to their symptoms.	__________________________________
Person 8: Did a health care provider tell this person that they may have COVID-19?	◯ yes<br>◯ no<br>◯ don't know
Person 8: If this person received a COVID-19 test due to their symptoms, what was the result?	◯ pending<br>◯ positive<br>◯ negative<br>◯ inconclusive<br>◯ did not receive a test<br>◯ don't know
Person 8: How many days was this person admitted to the hospital?	__________________________________
Person 8: Did this person receive any of the following interventions during their hospital admission?	☐ extra oxygen in your nose<br>☐ treatment in the intensive care unit (ICU)<br>☐ mechanical ventilation (intubation or a breathing tube)<br>☐ don't know
Person 8: Has this person returned to their normal health at this time?	◯ yes<br>◯ no<br>◯ don't know
Person 8: Which of the following did this person do to protect their friends and family after their symptoms began?	☐ wore a mask more frequently<br>☐ washed your hands with soap and water more frequently<br>☐ used hand sanitizer more frequently<br>☐ isolated yourself in your home more frequently<br>☐ stayed home more frequently<br>☐ wore disposable gloves more frequently<br>☐ don't know

For each additional person in the your household, please provide the following information.

Person 9: What is your relationship to this person?

- ◯ partner or spouse
- ◯ child
- ◯ parent
- ◯ sibling
- ◯ other family member
- ◯ in-home childcare provider or other caregiver
- ◯ other

Person 9: Please specify your relationship with this person.

____

Person 9: What is this person's age?

____
(Please specify their age in years)

Person 9: What is this person's sex?

- ◯ Female
- ◯ Male
- ◯ Other

Person 9: What is this person's race?

- ☐ American Indian or Alaska Native
- ☐ Asian
- ☐ Black or African American
- ☐ Native Hawaiian or Pacific Islander
- ☐ White
- ☐ Other
- ☐ don't know

(Select all that apply.)

Person 9: What is this person's ethnicity?

- ◯ Hispanic or Latino
- ◯ Not Hispanic or Latino
- ◯ Other
- ◯ don't know

Person 9: What is the highest level of education or schooling this person has completed?

- ◯ never attended school
- ◯ kindergarten - 8th grade
- ◯ some high school
- ◯ high school equivalency (GED)
- ◯ high school graduate
- ◯ some college
- ◯ college graduate
- ◯ graduate school or more
- ◯ don't know

Person 9: Which of the following best fit this person's current work situation?

- ◯ works full time
- ◯ works part time
- ◯ is looking for work/employment
- ◯ retired
- ◯ homemaker
- ◯ student
- ◯ on maternity/paternity leave
- ◯ on illness/sick leave
- ◯ on disability
- ◯ other
- ◯ don't know

Person 9: Does this person currently consider themselves self-employed (including as an independent contractor or gig-economy worker)?

- ◯ yes
- ◯ no
- ◯ don't know

Person 9: Does this person currently work in any of the following high-risk settings for COVID-19 transmission?	☐ healthcare setting (hospital, clinic, urgent care, etc.)<br>☐ dense residential setting (nursing home, other long-term care facility)<br>☐ prison or jail<br>☐ meatpacking facility<br>☐ shipping or distribution facility<br>☐ high-volume retail facility (grocery store, etc.)<br>☐ don't know
Person 9: Does this person's employer offer them any of the following benefits at their current main job?	☐ paid sick leave<br>☐ paid vacation/personal leave<br>☐ health insurance<br>☐ disability insurance<br>☐ retirement plan<br>☐ other<br>☐ don't know<br>(Select all that apply.)
Person 9: On a scale of 0 (definitely not going to happen) to 10 (definitely going to happen), how likely is it that this person will lose their job because of the COVID-19 pandemic?	__________________________________
Person 9: On a scale of 0 (definitely not going to happen) to 10 (definitely going to happen), how likely is it that this person will receive fewer work hours at their job because of the COVID-19 pandemic?	__________________________________

	always (100%)	most of the time (75%)	half of the time (50%)	less than half of the time (25%)	never (0%)	don't know
Person 9: How often is this person required to work from outside of the home currently?	◯	◯	◯	◯	◯	◯
Person 9: How regularly is this person in close physical contact with co-workers during their work outside of the home currently?	◯	◯	◯	◯	◯	◯
Person 9: How regularly is this person in close physical contact with clients during their work outside of the home currently?	◯	◯	◯	◯	◯	◯

Person 9: Does this person plan to get a vaccine for COVID-19 when one becomes available?	◯ yes<br>◯ no<br>◯ don't know
Person 9: Has this person had any symptoms (cough, fever, difficulty breathing, fatigue, body aches, diarrhea, runny nose, loss of smell or taste) consistent with COVID-19 in the last two weeks?	◯ yes<br>◯ no<br>◯ don't know
Person 9: When did this person's symptoms begin?	__________________________________

Person 9: Is this person worried that they may have had COVID-19 because of their symptoms?	◯ yes<br>◯ no<br>◯ don't know
Person 9: Did this person experience any bias or discrimination because of their symptoms?	◯ yes<br>◯ no<br>◯ don't know
Person 9: What did this person do in response to their symptoms?	☐ nothing<br>☐ took over the counter medication (ibuprofen, acetaminophen, etc.)<br>☐ communicated with a health care provider over the phone<br>☐ visited a health care provider's office<br>☐ visited a retail clinic or pharmacy<br>☐ visited urgent care (FASTMed, etc.)<br>☐ visited the emergency room<br>☐ was admitted to the hospital<br>☐ other<br>☐ don't know<br>(Select all that apply.)
Person 9: Please specify what other action this person took in response to their symptoms.	__________________________________
Person 9: Did a health care provider tell this person that they may have COVID-19?	◯ yes<br>◯ no<br>◯ don't know
Person 9: If this person received a COVID-19 test due to their symptoms, what was the result?	◯ pending<br>◯ positive<br>◯ negative<br>◯ inconclusive<br>◯ did not receive a test<br>◯ don't know
Person 9: How many days was this person admitted to the hospital?	__________________________________
Person 9: Did this person receive any of the following interventions during their hospital admission?	☐ extra oxygen in your nose<br>☐ treatment in the intensive care unit (ICU)<br>☐ mechanical ventilation (intubation or a breathing tube)<br>☐ don't know
Person 9: Has this person returned to their normal health at this time?	◯ yes<br>◯ no<br>◯ don't know
Person 9: Which of the following did this person do to protect their friends and family after their symptoms began?	☐ wore a mask more frequently<br>☐ washed your hands with soap and water more frequently<br>☐ used hand sanitizer more frequently<br>☐ isolated yourself in your home more frequently<br>☐ stayed home more frequently<br>☐ wore disposable gloves more frequently<br>☐ don't know

For each additional person in the your household, please provide the following information.

Person 10: What is your relationship to this person?

- ◯ partner or spouse
- ◯ child
- ◯ parent
- ◯ sibling
- ◯ other family member
- ◯ in-home childcare provider or other caregiver
- ◯ other

Person 10: Please specify your relationship with this person.

____

Person 10: What is this person's age?

____
(Please specify their age in years)

Person 10: What is this person's sex?

- ◯ Female
- ◯ Male
- ◯ Other

Person 10: What is this person's race?

- ☐ American Indian or Alaska Native
- ☐ Asian
- ☐ Black or African American
- ☐ Native Hawaiian or Pacific Islander
- ☐ White
- ☐ Other
- ☐ don't know

(Select all that apply.)

Person 10: What is this person's ethnicity?

- ◯ Hispanic or Latino
- ◯ Not Hispanic or Latino
- ◯ Other
- ◯ don't know

Person 10: What is the highest level of education or schooling this person has completed?

- ◯ never attended school
- ◯ kindergarten - 8th grade
- ◯ some high school
- ◯ high school equivalency (GED)
- ◯ high school graduate
- ◯ some college
- ◯ college graduate
- ◯ graduate school or more
- ◯ don't know

Person 10: Which of the following best fit this person's current work situation?

- ◯ works full time
- ◯ works part time
- ◯ is looking for work/employment
- ◯ retired
- ◯ homemaker
- ◯ student
- ◯ on maternity/paternity leave
- ◯ on illness/sick leave
- ◯ on disability
- ◯ other
- ◯ don't know

Person 10: Does this person currently consider themselves self-employed (including as an independent contractor or gig-economy worker)?

- ◯ yes
- ◯ no
- ◯ don't know

Person 10: Does this person currently work in any of the following high-risk settings for COVID-19 transmission?	☐ healthcare setting (hospital, clinic, urgent care, etc.)<br>☐ dense residential setting (nursing home, other long-term care facility)<br>☐ prison or jail<br>☐ meatpacking facility<br>☐ shipping or distribution facility<br>☐ high-volume retail facility (grocery store, etc.)<br>☐ don't know
Person 10: Does this person's employer offer them any of the following benefits at their current main job?	☐ paid sick leave<br>☐ paid vacation/personal leave<br>☐ health insurance<br>☐ disability insurance<br>☐ retirement plan<br>☐ other<br>☐ don't know<br>(Select all that apply.)
Person 10: On a scale of 0 (definitely not going to happen) to 10 (definitely going to happen), how likely is it that this person will lose their job because of the COVID-19 pandemic?	______________________________
Person 10: On a scale of 0 (definitely not going to happen) to 10 (definitely going to happen), how likely is it that this person will receive fewer work hours at their job because of the COVID-19 pandemic?	______________________________

	always (100%)	most of the time (75%)	half of the time (50%)	less than half of the time (25%)	never (0%)	don't know
Person 10: How often is this person required to work from outside of the home currently?	○	○	○	○	○	○
Person 10: How regularly is this person in close physical contact with co-workers during their work outside of the home currently?	○	○	○	○	○	○
Person 10: How regularly is this person in close physical contact with clients during their work outside of the home currently?	○	○	○	○	○	○

Person 10: Does this person plan to get a vaccine for COVID-19 when one becomes available?	○ yes<br>○ no<br>○ don't know
Person 10: Has this person had any symptoms (cough, fever, difficulty breathing, fatigue, body aches, diarrhea, runny nose, loss of smell or taste) consistent with COVID-19 in the last two weeks?	○ yes<br>○ no<br>○ don't know
Person 10: When did this person's symptoms begin?	______________________________

Person 10: Is this person worried that they may have had COVID-19 because of their symptoms?	◯ yes<br>◯ no<br>◯ don't know
Person 10: Did this person experience any bias or discrimination because of their symptoms?	◯ yes<br>◯ no<br>◯ don't know
Person 10: What did this person do in response to their symptoms?	☐ nothing<br>☐ took over the counter medication (ibuprofen, acetaminophen, etc.)<br>☐ communicated with a health care provider over the phone<br>☐ visited a health care provider's office<br>☐ visited a retail clinic or pharmacy<br>☐ visited urgent care (FASTMed, etc.)<br>☐ visited the emergency room<br>☐ was admitted to the hospital<br>☐ other<br>☐ don't know<br>(Select all that apply.)
Person 10: Please specify what other action this person took in response to their symptoms.	__________________________________
Person 10: Did a health care provider tell this person that they may have COVID-19?	◯ yes<br>◯ no<br>◯ don't know
Person 10: If this person received a COVID-19 test due to their symptoms, what was the result?	◯ pending<br>◯ positive<br>◯ negative<br>◯ inconclusive<br>◯ did not receive a test<br>◯ don't know
Person 10: How many days was this person admitted to the hospital?	__________________________________
Person 10: Did this person receive any of the following interventions during their hospital admission?	☐ extra oxygen in your nose<br>☐ treatment in the intensive care unit (ICU)<br>☐ mechanical ventilation (intubation or a breathing tube)<br>☐ don't know
Person 10: Has this person returned to their normal health at this time?	◯ yes<br>◯ no<br>◯ don't know
Person 10: Which of the following did this person do to protect their friends and family after their symptoms began?	☐ wore a mask more frequently<br>☐ washed your hands with soap and water more frequently<br>☐ used hand sanitizer more frequently<br>☐ isolated yourself in your home more frequently<br>☐ stayed home more frequently<br>☐ wore disposable gloves more frequently<br>☐ don't know

For each additional person in the your household, please provide the following information.

Person 11: What is your relationship to this person?

◯ partner or spouse
◯ child
◯ parent
◯ sibling
◯ other family member
◯ in-home childcare provider or other caregiver
◯ other

Person 11: Please specify your relationship with this person.

____

Person 11: What is this person's age?

____
(Please specify their age in years)

Person 11: What is this person's sex?

◯ Female
◯ Male
◯ Other

Person 11: What is this person's race?

☐ American Indian or Alaska Native
☐ Asian
☐ Black or African American
☐ Native Hawaiian or Pacific Islander
☐ White
☐ Other
☐ don't know
(Select all that apply.)

Person 11: What is this person's ethnicity?

◯ Hispanic or Latino
◯ Not Hispanic or Latino
◯ Other
◯ don't know

Person 11: What is the highest level of education or schooling this person has completed?

◯ never attended school
◯ kindergarten - 8th grade
◯ some high school
◯ high school equivalency (GED)
◯ high school graduate
◯ some college
◯ college graduate
◯ graduate school or more
◯ don't know

Person 11: Which of the following best fit this person's current work situation?

◯ works full time
◯ works part time
◯ is looking for work/employment
◯ retired
◯ homemaker
◯ student
◯ on maternity/paternity leave
◯ on illness/sick leave
◯ on disability
◯ other
◯ don't know

Person 11: Does this person currently consider themselves self-employed (including as an independent contractor or gig-economy worker)?

◯ yes
◯ no
◯ don't know

Person 11: Does this person currently work in any of the following high-risk settings for COVID-19 transmission?	☐ healthcare setting (hospital, clinic, urgent care, etc.)<br>☐ dense residential setting (nursing home, other long-term care facility)<br>☐ prison or jail<br>☐ meatpacking facility<br>☐ shipping or distribution facility<br>☐ high-volume retail facility (grocery store, etc.)<br>☐ don't know
Person 11: Does this person's employer offer them any of the following benefits at their current main job?	☐ paid sick leave<br>☐ paid vacation/personal leave<br>☐ health insurance<br>☐ disability insurance<br>☐ retirement plan<br>☐ other<br>☐ don't know<br>(Select all that apply.)
Person 11: On a scale of 0 (definitely not going to happen) to 10 (definitely going to happen), how likely is it that this person will lose their job because of the COVID-19 pandemic?	______________________
Person 11: On a scale of 0 (definitely not going to happen) to 10 (definitely going to happen), how likely is it that this person will receive fewer work hours at their job because of the COVID-19 pandemic?	______________________

	always (100%)	most of the time (75%)	half of the time (50%)	less than half of the time (25%)	never (0%)	don't know
Person 11: How often is this person required to work from outside of the home currently?	○	○	○	○	○	○
Person 11: How regularly is this person in close physical contact with co-workers during their work outside of the home currently?	○	○	○	○	○	○
Person 11: How regularly is this person in close physical contact with clients during their work outside of the home currently?	○	○	○	○	○	○

Person 11: Does this person plan to get a vaccine for COVID-19 when one becomes available?	○ yes<br>○ no<br>○ don't know
Person 11: Has this person had any symptoms (cough, fever, difficulty breathing, fatigue, body aches, diarrhea, runny nose, loss of smell or taste) consistent with COVID-19 in the last two weeks?	○ yes<br>○ no<br>○ don't know
Person 11: When did this person's symptoms begin?	______________________

Person 11: Is this person worried that they may have had COVID-19 because of their symptoms?	◯ yes<br>◯ no<br>◯ don't know
Person 11: Did this person experience any bias or discrimination because of their symptoms?	◯ yes<br>◯ no<br>◯ don't know
Person 11: What did this person do in response to their symptoms?	☐ nothing<br>☐ took over the counter medication (ibuprofen, acetaminophen, etc.)<br>☐ communicated with a health care provider over the phone<br>☐ visited a health care provider's office<br>☐ visited a retail clinic or pharmacy<br>☐ visited urgent care (FASTMed, etc.)<br>☐ visited the emergency room<br>☐ was admitted to the hospital<br>☐ other<br>☐ don't know<br>(Select all that apply.)
Person 11: Please specify what other action this person took in response to their symptoms.	__________________________
Person 11: Did a health care provider tell this person that they may have COVID-19?	◯ yes<br>◯ no<br>◯ don't know
Person 11: If this person received a COVID-19 test due to their symptoms, what was the result?	◯ pending<br>◯ positive<br>◯ negative<br>◯ inconclusive<br>◯ did not receive a test<br>◯ don't know
Person 11: How many days was this person admitted to the hospital?	__________________________
Person 11: Did this person receive any of the following interventions during their hospital admission?	☐ extra oxygen in your nose<br>☐ treatment in the intensive care unit (ICU)<br>☐ mechanical ventilation (intubation or a breathing tube)<br>☐ don't know
Person 11: Has this person returned to their normal health at this time?	◯ yes<br>◯ no<br>◯ don't know
Person 11: Which of the following did this person do to protect their friends and family after their symptoms began?	☐ wore a mask more frequently<br>☐ washed your hands with soap and water more frequently<br>☐ used hand sanitizer more frequently<br>☐ isolated yourself in your home more frequently<br>☐ stayed home more frequently<br>☐ wore disposable gloves more frequently<br>☐ don't know

For each additional person in the your household, please provide the following information.

Person 12: What is your relationship to this person?

- ◯ partner or spouse
- ◯ child
- ◯ parent
- ◯ sibling
- ◯ other family member
- ◯ in-home childcare provider or other caregiver
- ◯ other

Person 12: Please specify your relationship with this person.

____

Person 12: What is this person's age?

____
(Please specify their age in years)

Person 12: What is this person's sex?

- ◯ Female
- ◯ Male
- ◯ Other

Person 12: What is this person's race?

- ☐ American Indian or Alaska Native
- ☐ Asian
- ☐ Black or African American
- ☐ Native Hawaiian or Pacific Islander
- ☐ White
- ☐ Other
- ☐ don't know

(Select all that apply.)

Person 12: What is this person's ethnicity?

- ◯ Hispanic or Latino
- ◯ Not Hispanic or Latino
- ◯ Other
- ◯ don't know

Person 12: What is the highest level of education or schooling this person has completed?

- ◯ never attended school
- ◯ kindergarten - 8th grade
- ◯ some high school
- ◯ high school equivalency (GED)
- ◯ high school graduate
- ◯ some college
- ◯ college graduate
- ◯ graduate school or more
- ◯ don't know

Person 12: Which of the following best fit this person's current work situation?

- ◯ works full time
- ◯ works part time
- ◯ is looking for work/employment
- ◯ retired
- ◯ homemaker
- ◯ student
- ◯ on maternity/paternity leave
- ◯ on illness/sick leave
- ◯ on disability
- ◯ other
- ◯ don't know

Person 12: Does this person currently consider themselves self-employed (including as an independent contractor or gig-economy worker)?

- ◯ yes
- ◯ no
- ◯ don't know

Person 12: Does this person currently work in any of the following high-risk settings for COVID-19 transmission?	☐ healthcare setting (hospital, clinic, urgent care, etc.)<br>☐ dense residential setting (nursing home, other long-term care facility)<br>☐ prison or jail<br>☐ meatpacking facility<br>☐ shipping or distribution facility<br>☐ high-volume retail facility (grocery store, etc.)<br>☐ don't know
Person 12: Does this person's employer offer them any of the following benefits at their current main job?	☐ paid sick leave<br>☐ paid vacation/personal leave<br>☐ health insurance<br>☐ disability insurance<br>☐ retirement plan<br>☐ other<br>☐ don't know<br>(Select all that apply.)
Person 12: On a scale of 0 (definitely not going to happen) to 10 (definitely going to happen), how likely is it that this person will lose their job because of the COVID-19 pandemic?	________________________
Person 12: On a scale of 0 (definitely not going to happen) to 10 (definitely going to happen), how likely is it that this person will receive fewer work hours at their job because of the COVID-19 pandemic?	________________________

	always (100%)	most of the time (75%)	half of the time (50%)	less than half of the time (25%)	never (0%)	don't know
Person 12: How often is this person required to work from outside of the home currently?	○	○	○	○	○	○
Person 12: How regularly is this person in close physical contact with co-workers during their work outside of the home currently?	○	○	○	○	○	○
Person 12: How regularly is this person in close physical contact with clients during their work outside of the home currently?	○	○	○	○	○	○

Person 12: Does this person plan to get a vaccine for COVID-19 when one becomes available?	○ yes<br>○ no<br>○ don't know
Person 12: Has this person had any symptoms (cough, fever, difficulty breathing, fatigue, body aches, diarrhea, runny nose, loss of smell or taste) consistent with COVID-19 in the last two weeks?	○ yes<br>○ no<br>○ don't know
Person 12: When did this person's symptoms begin?	________________________

Person 12: Is this person worried that they may have had COVID-19 because of their symptoms?	◯ yes<br>◯ no<br>◯ don't know
Person 12: Did this person experience any bias or discrimination because of their symptoms?	◯ yes<br>◯ no<br>◯ don't know
Person 12: What did this person do in response to their symptoms?	☐ nothing<br>☐ took over the counter medication (ibuprofen, acetaminophen, etc.)<br>☐ communicated with a health care provider over the phone<br>☐ visited a health care provider's office<br>☐ visited a retail clinic or pharmacy<br>☐ visited urgent care (FASTMed, etc.)<br>☐ visited the emergency room<br>☐ was admitted to the hospital<br>☐ other<br>☐ don't know<br>(Select all that apply.)
Person 12: Please specify what other action this person took in response to their symptoms.	________________________________
Person 12: Did a health care provider tell this person that they may have COVID-19?	◯ yes<br>◯ no<br>◯ don't know
Person 12: If this person received a COVID-19 test due to their symptoms, what was the result?	◯ pending<br>◯ positive<br>◯ negative<br>◯ inconclusive<br>◯ did not receive a test<br>◯ don't know
Person 12: How many days was this person admitted to the hospital?	________________________________
Person 12: Did this person receive any of the following interventions during their hospital admission?	☐ extra oxygen in your nose<br>☐ treatment in the intensive care unit (ICU)<br>☐ mechanical ventilation (intubation or a breathing tube)<br>☐ don't know
Person 12: Has this person returned to their normal health at this time?	◯ yes<br>◯ no<br>◯ don't know
Person 12: Which of the following did this person do to protect their friends and family after their symptoms began?	☐ wore a mask more frequently<br>☐ washed your hands with soap and water more frequently<br>☐ used hand sanitizer more frequently<br>☐ isolated yourself in your home more frequently<br>☐ stayed home more frequently<br>☐ wore disposable gloves more frequently<br>☐ don't know

Please provide the following information about your mental health and wellness.

How serious a problem would you say the COVID-19 pandemic is for you personally right now?
- ◯ very serious
- ◯ somewhat serious
- ◯ not too serious
- ◯ not at all serious

How serious a problem would you say the COVID-19 pandemic is for people in your community right now?
- ◯ very serious
- ◯ somewhat serious
- ◯ not too serious
- ◯ not at all serious

How serious a problem would you say the COVID-19 pandemic is for people in the United States right now?
- ◯ very serious
- ◯ somewhat serious
- ◯ not too serious
- ◯ not at all serious

How serious a problem would you say the COVID-19 pandemic is for people around the world right now?
- ◯ very serious
- ◯ somewhat serious
- ◯ not too serious
- ◯ not at all serious

In the last two weeks, how often have you encountered news media, a viral/trending event, online videos, online discussions, or photos in which someone like you was threatened or harmed?
- ◯ Never
- ◯ About once a month
- ◯ About once a week
- ◯ About once a day
- ◯ Several times a day

In the last two weeks, how often have you encountered news media, a viral/trending event, online videos, online discussions, or photos in which negative stereotypes of people like you were promoted as if they were true?
- ◯ Never
- ◯ About once a month
- ◯ About once a week
- ◯ About once a day
- ◯ Several times a day

The COVID-19 pandemic may cause challenges for some people regardless of whether they are infected. How concerned are you about each of the following things?

	not at all concerned	not too concerned	somewhat concerned	very concerned
Getting the healthcare you need (including care for mental health)	◯	◯	◯	◯
Having a place to live	◯	◯	◯	◯
Being able to interact with other people	◯	◯	◯	◯
Getting food, water, and other household supplies	◯	◯	◯	◯
Getting medication	◯	◯	◯	◯
Having transportation to get where you need to go	◯	◯	◯	◯
Caring for your family and friends	◯	◯	◯	◯

During the last two weeks, how often have you been bothered by the following problems?

	not at all	several days	more than half of the days	nearly every day
Feeling nervous, anxious, or on edge	◯	◯	◯	◯
Not being able to stop or control worrying	◯	◯	◯	◯
Worrying too much about different things	◯	◯	◯	◯
Trouble relaxing	◯	◯	◯	◯
Being so restless that it's hard to sit still	◯	◯	◯	◯
Becoming easily annoyed or irritable	◯	◯	◯	◯
Feeling afraid as if something awful might happen	◯	◯	◯	◯

How difficult have these problems made it for you to do your work, take care of things at home, or get along with other people?

- ◯ not difficult
- ◯ somewhat difficult
- ◯ very difficult
- ◯ extremely difficult

In the last two weeks, how often have you experienced the following feelings and behaviors?

	Rarely or none of the time (< 1 day)	Some or a little of the time (1-2 days)	Occasionally or a moderate amount of the time (3-4 days)	Most or all of the time (5-7 days)
I was bothered by things that usually don't bother me.	◯	◯	◯	◯
I did not feel like eating; my appetite was poor.	◯	◯	◯	◯
I felt that I could not shake off the blues even with the help from my family and friends.	◯	◯	◯	◯
I felt that I was not as good as other people.	◯	◯	◯	◯
I had trouble keeping my mind on what I was doing.	◯	◯	◯	◯
I felt depressed.	◯	◯	◯	◯
I felt that everything I did was an effort.	◯	◯	◯	◯
I felt hopeless about the future.	◯	◯	◯	◯
I thought my life had been a failure.	◯	◯	◯	◯

I felt fearful.	◯	◯	◯	◯
My sleep was restless.	◯	◯	◯	◯
I was unhappy.	◯	◯	◯	◯
I talked less than usual.	◯	◯	◯	◯
I felt lonely.	◯	◯	◯	◯
People were unfriendly.	◯	◯	◯	◯
I did not enjoy life.	◯	◯	◯	◯
I had crying spells.	◯	◯	◯	◯
I felt sad.	◯	◯	◯	◯
I felt that people disliked me.	◯	◯	◯	◯
I could not get "going".	◯	◯	◯	◯

How did you complete this survey?

- ◯ on a computer (laptop or desktop)
- ◯ on a mobile device (cell phone or tablet)
- ◯ on a phone with an interviewer
- ◯ other

¿Cuál de las siguientes es la mejor descripción de su situación laboral actual?

- ◯ Empleo a tiempo completo
- ◯ Empleo a tiempo parcial
- ◯ En busca de empleo
- ◯ Jubilado
- ◯ Ama de casa
- ◯ Estudiante
- ◯ Permiso de maternidad o paternidad
- ◯ Permiso por enfermedad
- ◯ Desempleado por discapacidad
- ◯ Otra

¿Se considera usted actualmente empleado por cuenta propia (contratista independiente, trabajador esporádico (gig), etc.)?

- ◯ Sí
- ◯ No
- ◯ No sabe

En una escala de 0 (definitivamente no va a suceder) a 10 (definitivamente va a suceder), ¿qué tan probable es que usted pierda su empleo debido a la pandemia de COVID-19?

____

En una escala de 0 (definitivamente no va a suceder) a 10 (definitivamente va a suceder), ¿qué tan probable es que le asignen menos horas de trabajo debido a la pandemia de COVID-19?

____

En su opinión ¿qué tan probable es que su grupo familiar se quede sin dinero en los próximos 3 meses? Dé su respuesta en una escala de 0 (definitivamente no va a suceder) a 10 (definitivamente va a suceder).

____

	Todo el tiempo (100%)	La mayor parte del tiempo (75%)	La mitad del tiempo (50%)	Menos de la mitad del tiempo (25%)	Nunca (0%)

Actualmente, ¿con qué frecuencia se le exige que trabaje fuera de su residencia?	○	○	○	○	○
Actualmente, ¿con qué frecuencia se encuentra usted físicamente cerca de sus compañeros de trabajo mientras trabaja fuera de su residencia?	○	○	○	○	○
Actualmente, ¿con qué frecuencia se encuentra usted físicamente cerca de los clientes mientras trabaja fuera de su residencia?	○	○	○	○	○
Actualmente, ¿con qué frecuencia tiene acceso a guantes desechables mientras trabaja fuera de su residencia?	○	○	○	○	○
Actualmente, ¿con qué frecuencia tiene acceso a una mascarilla mientras trabaja fuera de su residencia?	○	○	○	○	○
Actualmente, ¿con qué frecuencia lleva usted guantes desechables mientras trabaja fuera de su residencia?	○	○	○	○	○
Actualmente, ¿con qué frecuencia lleva usted mascarilla mientras trabaja fuera de su residencia?	○	○	○	○	○
Actualmente, ¿con qué frecuencia se lava las manos con agua y jabón mientras trabaja fuera de su residencia?	○	○	○	○	○
Actualmente, ¿con qué frecuencia se desinfecta las manos con desinfectante de manos mientras trabaja fuera de su residencia?	○	○	○	○	○
Actualmente, ¿cuánto le preocupa exponerse a la COVID-19 mientras trabaja fuera de su residencia?	○	○	○	○	○

¿Trabaja usted actualmente en alguno de los siguientes ambientes de alto riesgo de contagio de COVID-19?	☐ Ambiente de asistencia médica (hospital, clínica, centro de urgencias)<br>☐ Ambiente residencial denso (hogar de ancianos, otro centro de asistencia de larga duración)<br>☐ Prisión o cárcel<br>☐ Establecimiento de envasado de carne<br>☐ Establecimiento de envío o distribución<br>☐ Establecimiento minorista de alto volumen (tienda de provisiones, etc.)
¿Tiene usted seguro médico o de salud (seguro privado, seguro que usted ha comprado, Medicare, Medicaid o algún otro tipo de seguro)?	◯ Sí<br>◯ No<br>◯ No sabe
¿Cuál es su principal seguro de salud?	◯ Seguro médico privado por empleo o establecimiento educacional<br>◯ Seguro comprado en un intercambio de seguros de salud estatal o federal, tal como healthcare.gov<br>◯ Seguro comprado directamente a una compañía de seguros.<br>◯ Medicare<br>◯ Medi-Gap<br>◯ Medicaid<br>◯ Asistencia médica militar (TRICARE, VA, CHAMP-VA, etc.)<br>◯ Servicio de Salud Indígena<br>◯ Otro<br>((Seleccione uno (su seguro principal).))
Si marcó "Otro", especifique la fuente de seguro médico.	__________________________________
¿Está usted actualmente embarazada?	◯ Sí<br>◯ No

	Excelente	Muy buena	Buena	Regular	Mala
En general, ¿cómo calificaría usted su salud en las últimas dos semanas?	◯	◯	◯	◯	◯

Con que frequencia ha hecho lo siguiente durante las ultimas dos semanas para protegerse contra la infeccion?

	Siempre (100%)	La mayor parte del tiempo (75%)	La mitad del tiempo (50%)	Menos de la mitad del tiempo (25%)	Nunca (0%)
Llevar mascarilla	◯	◯	◯	◯	◯
Lavarse las manos y/o limpiárselas con desinfectante a menudo	◯	◯	◯	◯	◯
Mantenerse al menos a 6 pies de distancia de los demás	◯	◯	◯	◯	◯

Evitar grandes reuniones, espacios públicos o multitudes	◯	◯	◯	◯	◯
Evitar el contacto con personas de alto riesgo	◯	◯	◯	◯	◯
No comer en restaurante, ni siquiera comprar comida para llevar	◯	◯	◯	◯	◯
Trabajar o estudiar en casa en lugar de ir a una oficina o a un aula	◯	◯	◯	◯	◯
No dar la mano ni tocar a otras personas.	◯	◯	◯	◯	◯
Quedarse en casa cuando se está enfermo	◯	◯	◯	◯	◯
Limpiar las superficies con desinfectante	◯	◯	◯	◯	◯
Cancelar o postergar viajes de trabajo	◯	◯	◯	◯	◯
Cancelar o postergar viajes de placer	◯	◯	◯	◯	◯
Cancelar o postergar viajes las actividades personales o sociales	◯	◯	◯	◯	◯
Cancelar las consultas médicas en persona	◯	◯	◯	◯	◯
Almacenar comida o agua	◯	◯	◯	◯	◯
Seguir las recomendaciones o reglas del gobierno sobre refugiarse donde se esté (es decir, quedarse en casa, limitar el contacto con otras personas, etc.)	◯	◯	◯	◯	◯

Durante los ultimos dos semanas, ha tenido alguno de los siguientes sintomas?

	Sí	No
Fiebre (medida por termómetro o autodiagnóstico)	◯	◯
Tos (nueva o que empeora)	◯	◯
Dificultad para respirar (nueva o que empeora)	◯	◯
Fatiga (nuevo cansancio al hacer actividades normales)	◯	◯
Dolores de cuerpo	◯	◯
Dolor de cabeza	◯	◯

Diarrea	○	○
Dolor de garganta	○	○
Picazón, enrojecimiento o dolor de ojos	○	○
Goteo o congestión nasal	○	○
Cambios en el sentido del olfato o del gusto	○	○
Nuevo sarpullido	○	○
Temblores y escalofríos reiterados	○	○

¿Cuándo comenzaron estos síntomas?

____

¿Fue usted objeto de prejuicio o discriminación debido a sus síntomas?

- ○ Sí
- ○ No
- ○ No sabe

¿Cuáles de las siguientes medidas tomó usted para proteger a sus amigos y familiares después de que comenzaron sus síntomas?

- ☐ Ponerse mascarilla con mayor frecuencia
- ☐ Lavarse las manos con agua y jabón con mayor frecuencia
- ☐ Limpiarse las manos con desinfectante con mayor frecuencia
- ☐ Aislarse en su casa con mayor frecuencia
- ☐ Quedarse en casa con mayor frecuencia
- ☐ Ponerse guantes desechables con mayor frecuencia

¿Qué hizo usted en vista de sus síntomas?

- ☐ Nada
- ☐ Tomó medicamentos sin receta (ibuprofeno, acetaminofén, etc.)
- ☐ Consultó por teléfono a un proveedor de asistencia médica
- ☐ Fue al consultorio de un proveedor de asistencia
- ☐ Fue a una clínica o una farmacia minorista
- ☐ Fue a un centro de urgencias (FASTmed, etc.)
- ☐ Fue a la sala de emergencias
- ☐ Fue ingresado al hospital
- ☐ Otra cosa

((Seleccione todo lo que corresponda.))

¿Qué otra cosa hizo usted en vista de sus síntomas?

____

Si pudo hablar con un proveedor de asistencia médica, ¿le dijo el proveedor que era posible que tuviera COVID-19?

- ○ Sí
- ○ No
- ○ No sabe

Si le hicieron una prueba de COVID-19 en vista de los síntomas que declaró, ¿cuál fue el resultado?

- ○ Pendiente
- ○ Positivo
- ○ Negativo
- ○ No concluyente
- ○ No se hizo la prueba

¿Cuántos días estuvo usted hospitalizado?

____

¿Le hicieron a usted las siguientes intervenciones durante su hospitalización?	☐ Oxígeno adicional por la nariz<br>☐ Tratamiento en la Unidad de Cuidados Intensivos (Intensive Care Unit, ICU)<br>☐ Ventilación mecánica (intubación o tubo de respiración)
¿Ha vuelto usted a su salud normal?	◯ Sí<br>◯ No<br>◯ No sabe
¿Ha participado o está participando actualmente en un ensayo de la vacuna de COVID?	◯ Yes<br>◯ No
¿Dónde fue o es el sitio principal para el ensayo de la vacuna de COVID? (Por ejemplo, ¿UNC, Duke o un hospital específico?)	________________________________
¿Ha recibido una vacuna de COVID-19 fuera de un ensayo clínico?	◯ Sí<br>◯ No
¿Dónde recibió la vacuna de COVID-19?	◯ Oficina del doctor/medico<br>◯ Trabajo/Empleo<br>◯ Minorista (por ejemplo Walgreens, CVS)<br>◯ Sitio de la vacuna<br>◯ Otro
Especifique donde recibió la vacuna de COVID-19.	________________________________
Especifique la cuidad/el pueblo en Carolina del Norte del sitio de la vacuna:	________________________________
Especifique quien fue la organización o instituto que organizo el sitio de la vacuna:	________________________________
¿Cuál vacuna de COVID-19 recibió?	◯ Pfizer<br>◯ Moderna<br>◯ AstraZeneca<br>◯ Novavax<br>◯ Johnson & Johnson<br>◯ Otro<br>◯ No sabe
Especifique que otra vacuna de COVID recibió.	________________________________
¿Cuántas dosis de la vacuna ha recibido?	◯ 1<br>◯ 2
¿Qué día recibió la primera dosis de la vacuna?	________________________________
¿Qué día recibió la segunda dosis de la vacuna?	________________________________

Did you experience any of the following side effects after vaccination?

		No	Leve (nota síntomas, pero no son un problema)	Moderado (los síntomas limitan sus actividades diarias normales)	Grave (los síntomas hacen que las actividades normales sean difícil o imposible)
1	dolor en o alrededor del lugar de la inyección	◯	◯	◯	◯
2	enrojecimiento en o alrededor del lugar de la inyección	◯	◯	◯	◯
3	hinchazón en o alrededor del lugar de la inyección	◯	◯	◯	◯
4	erupción en o alrededor del lugar de la inyección	◯	◯	◯	◯
5	dolor de cabeza	◯	◯	◯	◯
6	fatiga/cansancio	◯	◯	◯	◯
7	fiebre (temperatura >100.4°F o >38°C)	◯	◯	◯	◯
8	escalofríos	◯	◯	◯	◯
9	dolor en las articulaciones	◯	◯	◯	◯
10	dolor muscular	◯	◯	◯	◯
11	nausea	◯	◯	◯	◯

¿Cuánto tiempo duraron estos efectos secundarios?

◯ menos de 12 horas
◯ 12 a 24 horas
◯ más de 24 horas

¿Tomo algún medicamento para estos efectos secundarios?

◯ Sí
◯ No

¿Que medicamento(s) tomo para los efectos secundarios?

____
(Por favor enumere todos los medicamentos.)

¿Consultó a un médico u otro proveedor de atención médica para los efectos secundarios?

◯ Sí
◯ No

¿Como experimento los efectos secundarios después de la secunda dosis de la vacuna en comparación con los efectos secundarios después de la primera dosis de la vacuna?

◯ más grave
◯ menos grave
◯ igualmenta grave
◯ no corresponde/aún no he recibido la segunda dosis

Proporcione la siguiente informacion sobre su hogar.

¿Cuál es su dirección permanente?

____

¿Cuanto tiempo ha vivido en esta dirección?

- ◯ 0-3 años
- ◯ 4-6 años
- ◯ 7-10 años
- ◯ Más de 10 años

¿Cuántas personas (sin contarse usted) viven o pasan una cantidad considerable de tiempo en esta vivienda (más de 40 horas por semana)?

- ◯ 0
- ◯ 1
- ◯ 2
- ◯ 3
- ◯ 4
- ◯ 5
- ◯ 6
- ◯ 7
- ◯ 8
- ◯ 9
- ◯ 10
- ◯ 11
- ◯ 12

¿Cuántas personas de su grupo familiar son menores de 18 años?

- ◯ 0
- ◯ 1
- ◯ 2
- ◯ 3
- ◯ 4
- ◯ 5
- ◯ 6
- ◯ 7
- ◯ 8
- ◯ 9
- ◯ 10
- ◯ 11
- ◯ 12

Por cada persona adicional en su hogar, proporcione la siguiente informacion.

Persona 1: ¿Cuál es su relación con esta persona?

- ◯ Pareja o cónyuge
- ◯ Hijo o hija
- ◯ Padre o madre
- ◯ Hermano o hermana
- ◯ Otro familiar
- ◯ Proveedor de cuidado infantil u otros cuidados en casa
- ◯ Otra

Persona 1: Especifique su relación con esta persona.

____

Persona 1: ¿Qué edad tiene esta persona?

____
((Especifique la edad en años))

Persona 1: ¿Cuál es el sexo de esta persona?

- ◯ Femenino
- ◯ Masculino

Persona 1: ¿Cuál es la raza de esta persona?	☐ Indio americano o nativo de Alaska<br>☐ Asiático<br>☐ Negro o afroestadounidense<br>☐ Nativo de Hawái o de las islas del Pacífico<br>☐ Blanco<br>☐ Otra<br>☐ No sabe<br>((Seleccione todo lo que corresponda.))
Persona 1: ¿Cuál es la identidad étnica de esta persona?	◯ Hispano o latino<br>◯ Ni hispano ni latino<br>◯ Otra<br>◯ No sabe
Persona 1: ¿Cuál es el nivel de educación de esta persona?	◯ No tiene educación formal<br>◯ Kínder a 8° grado<br>◯ Estudios de educación secundaria<br>◯ Equivalencia de educación secundaria (GED)<br>◯ Diploma de educación secundaria<br>◯ Estudios de educación superior<br>◯ Título universitario<br>◯ Estudios de posgrado o más<br>◯ No sabe
Persona 1: ¿Cuál de las siguientes es la mejor descripción de la situación laboral actual de esta persona?	◯ Empleo a tiempo completo<br>◯ Empleo a tiempo parcial<br>◯ En busca de empleo<br>◯ Jubilado<br>◯ Ama de casa<br>◯ Estudiante<br>◯ Permiso de maternidad o paternidad<br>◯ Permiso por enfermedad<br>◯ Desempleado por discapacidad<br>◯ Otra<br>◯ No sabe
Persona 1: ¿Se considera esta persona actualmente empleado por cuenta propia (contratista independiente, trabajador esporádico (gig), etc.)?	◯ Sí<br>◯ No<br>◯ No sabe
Persona 1: ¿Trabaja esta persona actualmente en alguno de los siguientes ambientes de alto riesgo de contagio de COVID-19?	☐ Ambiente de asistencia médica (hospital, clínica, centro de urgencias, etc.)<br>☐ Ambiente residencial denso (hogar de ancianos, otro centro de asistencia de larga duración)<br>☐ Prisión o cárcel<br>☐ Establecimiento de envasado de carne<br>☐ Establecimiento de envío o distribución<br>☐ Establecimiento minorista de alto volumen (tienda de provisiones, etc.)<br>☐ No sabe
Persona 1: ¿Le ofrece el empleador a esta persona alguno de los siguientes beneficios en su empleo principal actual?	☐ Permiso por enfermedad con goce de sueldo<br>☐ Vacaciones o permiso personal con goce de sueldo<br>☐ Seguro de salud<br>☐ Seguro de discapacidad<br>☐ Plan de jubilación<br>☐ Otro<br>☐ No sabe<br>((Seleccione todo lo que corresponda.))

Persona 1: En una escala de 0 (definitivamente no va a suceder) a 10 (definitivamente va a suceder), ¿qué tan probable es que esta persona pierda su empleo debido a la pandemia de COVID-19? __________________________________

Persona 1: En una escala de 0 (definitivamente no va a suceder) a 10 (definitivamente va a suceder), ¿qué tan probable es que a esta persona se le asignen menos horas de trabajo debido a la pandemia de COVID-19? __________________________________

	Todo el tiempo (100%)	La mayor parte del tiempo (75%)	La mitad del tiempo (50%)	Menos de la mitad del tiempo (25%)	nunca (0%)	Nunca
Persona 1: Actualmente ¿con qué frecuencia se le exige a esta persona que trabaje fuera de su residencia?	◯	◯	◯	◯	◯	◯
Persona 1: Actualmente, ¿con qué frecuencia se encuentra esta persona físicamente cerca de sus compañeros de trabajo mientras trabaja fuera de su residencia?	◯	◯	◯	◯	◯	◯
Persona 1: Actualmente, ¿con qué frecuencia se encuentra esta persona físicamente cerca de sus clientes mientras trabaja fuera de su residencia	◯	◯	◯	◯	◯	◯

Persona 1: ¿Piensa esta persona vacunarse contra la COVID-19 cuando se ofrezca una vacuna?

◯ Sí
◯ No
◯ No sabe

Persona 1: En las últimas dos semanas, ¿ha tenido esta persona algún síntoma de COVID-19 (tos, fiebre, dificultad para respirar, fatiga, dolores de cuerpo, diarrea, goteo nasal o pérdida del sentido del olfato o del gusto)?

◯ Sí
◯ No
◯ No sabe

Persona 1: ¿Cuándo le comenzaron los síntomas de COVID-19 a esta persona? __________________________________

Persona 1: En vista de sus síntomas, ¿le preocupa a esta persona la posibilidad de tener COVID-19?

◯ Sí
◯ No
◯ No sabe

Persona 1: ¿Fue esta persona objeto de prejuicio o discriminación debido a sus síntomas?

◯ Sí
◯ No
◯ No sabe

Persona 1: ¿Qué hizo esta persona en vista de sus síntomas?	☐ Nada<br>☐ Tomó medicamentos sin receta (ibuprofeno, acetaminofén, etc.)<br>☐ Consultó por teléfono a un proveedor de asistencia médica<br>☐ Fue al consultorio de un proveedor de asistencia médica<br>☐ Fue a una clínica o una farmacia minorista<br>☐ Fue a un centro de urgencias (FASTMed, etc.)<br>☐ Fue a la sala de emergencias<br>☐ Fue ingresado al hospital<br>☐ Otra cosa<br>☐ No sabe<br>((Seleccione todo lo que corresponda.))
Persona 1: ¿Qué otra cosa hizo esta persona en vista de sus síntomas?	______________________________
Persona 1: ¿Le dijo un proveedor de asistencia médica a esta persona que era posible que tuviera COVID-19?	◯ Sí<br>◯ No<br>◯ No sabe
Persona 1: Si a esta persona le hicieron una prueba de COVID-19 en vista de sus síntomas, ¿cuál fue el resultado?	◯ Pendiente<br>◯ Positivo<br>◯ Negativo<br>◯ No concluyente<br>◯ No se hizo la prueba<br>◯ No sabe
Persona 1: ¿Cuántos días estuvo hospitalizada esta persona?	______________________________
Persona 1: ¿Se le hicieron a esta persona las siguientes intervenciones durante su hospitalización?	☐ Oxígeno adicional por la nariz<br>☐ Tratamiento en la Unidad de Cuidados Intensivos (Intensive Care Unit, ICU)<br>☐ Ventilación mecánica (intubación o tubo de respiración)<br>☐ No sabe
Persona 1: ¿Ha vuelto esta persona a su salud normal?	◯ Sí<br>◯ No<br>◯ No sabe
Persona 1: ¿Cuáles de las siguientes medidas tomó esta persona para proteger a sus amigos y familiares después de que comenzaron sus síntomas?	☐ Ponerse mascarilla con mayor frecuencia<br>☐ Lavarse las manos con agua y jabón con mayor frecuencia<br>☐ Limpiarse las manos con desinfectante con mayor frecuencia<br>☐ Aislarse en su casa con mayor frecuencia<br>☐ Quedarse en casa con mayor frecuencia<br>☐ Ponerse guantes desechables con mayor frecuencia<br>☐ No sabe

Por cada persona adicional en su hogar, proporcione la siguiente informacion.

Persona 2: ¿Cuál es su relación con esta persona?	◯ Pareja o cónyuge<br>◯ Hijo o hija<br>◯ Padre o madre<br>◯ Hermano o hermana<br>◯ Otro familiar<br>◯ Proveedor de cuidado infantil u otros cuidados en casa<br>◯ Otra
Persona 2: Especifique su relación con esta persona.	__________________________________
Persona 2: ¿Qué edad tiene esta persona?	__________________________________<br>((Especifique la edad en años))
Persona 2: ¿Cuál es el sexo de esta persona?	◯ Femenino<br>◯ Masculino
Persona 2: ¿Cuál es la raza de esta persona?	☐ Indio americano o nativo de Alaska<br>☐ Asiático<br>☐ Negro o afroestadounidense<br>☐ Nativo de Hawái o de las islas del Pacífico<br>☐ Blanco<br>☐ Otra<br>☐ No sabe<br>((Seleccione todo lo que corresponda.))
Persona 2: ¿Cuál es la identidad étnica de esta persona?	◯ Hispano o latino<br>◯ Ni hispano ni latino<br>◯ Otra<br>◯ No sabe
Persona 2: ¿Cuál es el nivel de educación de esta persona?	◯ No tiene educación formal<br>◯ Kínder a 8° grado<br>◯ Estudios de educación secundaria<br>◯ Equivalencia de educación secundaria (GED)<br>◯ Diploma de educación secundaria<br>◯ Estudios de educación superior<br>◯ Título universitario<br>◯ Estudios de posgrado o más<br>◯ No sabe
Persona 2: ¿Cuál de las siguientes es la mejor descripción de la situación laboral actual de esta persona?	◯ Empleo a tiempo completo<br>◯ Empleo a tiempo parcial<br>◯ En busca de empleo<br>◯ Jubilado<br>◯ Ama de casa<br>◯ Estudiante<br>◯ Permiso de maternidad o paternidad<br>◯ Permiso por enfermedad<br>◯ Desempleado por discapacidad<br>◯ Otra<br>◯ No sabe
Persona 2: ¿Se considera esta persona actualmente empleado por cuenta propia (contratista independiente, trabajador esporádico (gig), etc.)?	◯ Sí<br>◯ No<br>◯ No sabe

Persona 2: ¿Trabaja esta persona actualmente en alguno de los siguientes ambientes de alto riesgo de contagio de COVID-19?	☐ Ambiente de asistencia médica (hospital, clínica, centro de urgencias, etc.)<br>☐ Ambiente residencial denso (hogar de ancianos, otro centro de asistencia de larga duración)<br>☐ Prisión o cárcel<br>☐ Establecimiento de envasado de carne<br>☐ Establecimiento de envío o distribución<br>☐ Establecimiento minorista de alto volumen (tienda de provisiones, etc.)<br>☐ No sabe
Persona 2: ¿Le ofrece el empleador a esta persona alguno de los siguientes beneficios en su empleo principal actual?	☐ Permiso por enfermedad con goce de sueldo<br>☐ Vacaciones o permiso personal con goce de sueldo<br>☐ Seguro de salud<br>☐ Seguro de discapacidad<br>☐ Plan de jubilación<br>☐ Otro<br>☐ No sabe<br>((Seleccione todo lo que corresponda.))
Persona 2: En una escala de 0 (definitivamente no va a suceder) a 10 (definitivamente va a suceder), ¿qué tan probable es que esta persona pierda su empleo debido a la pandemia de COVID-19?	__________________________
Persona 2: En una escala de 0 (definitivamente no va a suceder) a 10 (definitivamente va a suceder), ¿qué tan probable es que a esta persona se le asignen menos horas de trabajo debido a la pandemia de COVID-19?	__________________________

	Todo el tiempo (100%)	La mayor parte del tiempo (75%)	La mitad del tiempo (50%)	Menos de la mitad del tiempo (25%)	nunca (0%)	Nunca
Persona 2: Actualmente ¿con qué frecuencia se le exige a esta persona que trabaje fuera de su residencia?	○	○	○	○	○	○
Persona 2: Actualmente, ¿con qué frecuencia se encuentra esta persona físicamente cerca de sus compañeros de trabajo mientras trabaja fuera de su residencia?	○	○	○	○	○	○
Persona 2: Actualmente, ¿con qué frecuencia se encuentra esta persona físicamente cerca de sus clientes mientras trabaja fuera de su residencia	○	○	○	○	○	○

Persona 2: ¿Piensa esta persona vacunarse contra la COVID-19 cuando se ofrezca una vacuna?	○ Sí<br>○ No<br>○ No sabe

Persona 2: En las últimas dos semanas, ¿ha tenido esta persona algún síntoma de COVID-19 (tos, fiebre, dificultad para respirar, fatiga, dolores de cuerpo, diarrea, goteo nasal o pérdida del sentido del olfato o del gusto)?	◯ Sí<br>◯ No<br>◯ No sabe
Persona 2: ¿Cuándo le comenzaron los síntomas de COVID-19 a esta persona?	______________________
Persona 2: En vista de sus síntomas, ¿le preocupa a esta persona la posibilidad de tener COVID-19?	◯ Sí<br>◯ No<br>◯ No sabe
Persona 2: ¿Fue esta persona objeto de prejuicio o discriminación debido a sus síntomas?	◯ Sí<br>◯ No<br>◯ No sabe
Persona 2: ¿Qué hizo esta persona en vista de sus síntomas?	☐ Nada<br>☐ Tomó medicamentos sin receta (ibuprofeno, acetaminofén, etc.)<br>☐ Consultó por teléfono a un proveedor de asistencia médica<br>☐ Fue al consultorio de un proveedor de asistencia médica<br>☐ Fue a una clínica o una farmacia minorista<br>☐ Fue a un centro de urgencias (FASTMed, etc.)<br>☐ Fue a la sala de emergencias<br>☐ Fue ingresado al hospital<br>☐ Otra cosa<br>☐ No sabe<br>((Seleccione todo lo que corresponda.))
Persona 2: ¿Qué otra cosa hizo esta persona en vista de sus síntomas?	______________________
Persona 2: ¿Le dijo un proveedor de asistencia médica a esta persona que era posible que tuviera COVID-19?	◯ Sí<br>◯ No<br>◯ No sabe
Persona 2: Si a esta persona le hicieron una prueba de COVID-19 en vista de sus síntomas, ¿cuál fue el resultado?	◯ Pendiente<br>◯ Positivo<br>◯ Negativo<br>◯ No concluyente<br>◯ No se hizo la prueba<br>◯ No sabe
Persona 2: ¿Cuántos días estuvo hospitalizada esta persona?	______________________
Persona 2: ¿Se le hicieron a esta persona las siguientes intervenciones durante su hospitalización?	☐ Oxígeno adicional por la nariz<br>☐ Tratamiento en la Unidad de Cuidados Intensivos (Intensive Care Unit, ICU)<br>☐ Ventilación mecánica (intubación o tubo de respiración)<br>☐ No sabe
Persona 2: ¿Ha vuelto esta persona a su salud normal?	◯ Sí<br>◯ No<br>◯ No sabe

Persona 2: ¿Cuáles de las siguientes medidas tomó esta persona para proteger a sus amigos y familiares después de que comenzaron sus síntomas?

- ☐ Ponerse mascarilla con mayor frecuencia
- ☐ Lavarse las manos con agua y jabón con mayor frecuencia
- ☐ Limpiarse las manos con desinfectante con mayor frecuencia
- ☐ Aislarse en su casa con mayor frecuencia
- ☐ Quedarse en casa con mayor frecuencia
- ☐ Ponerse guantes desechables con mayor frecuencia
- ☐ No sabe

Por cada persona adicional en su hogar, proporcione la siguiente informacion.

Persona 3: ¿Cuál es su relación con esta persona?

- ◯ Pareja o cónyuge
- ◯ Hijo o hija
- ◯ Padre o madre
- ◯ Hermano o hermana
- ◯ Otro familiar
- ◯ Proveedor de cuidado infantil u otros cuidados en casa
- ◯ Otra

Persona 3: Especifique su relación con esta persona.

____

Persona 3: ¿Qué edad tiene esta persona?

____
((Especifique la edad en años))

Persona 3: ¿Cuál es el sexo de esta persona?

- ◯ Femenino
- ◯ Masculino

Persona 3: ¿Cuál es la raza de esta persona?

- ☐ Indio americano o nativo de Alaska
- ☐ Asiático
- ☐ Negro o afroestadounidense
- ☐ Nativo de Hawái o de las islas del Pacífico
- ☐ Blanco
- ☐ Otra
- ☐ No sabe

((Seleccione todo lo que corresponda.))

Persona 3: ¿Cuál es la identidad étnica de esta persona?

- ◯ Hispano o latino
- ◯ Ni hispano ni latino
- ◯ Otra
- ◯ No sabe

Persona 3: ¿Cuál es el nivel de educación de esta persona?

- ◯ No tiene educación formal
- ◯ Kínder a 8° grado
- ◯ Estudios de educación secundaria
- ◯ Equivalencia de educación secundaria (GED)
- ◯ Diploma de educación secundaria
- ◯ Estudios de educación superior
- ◯ Título universitario
- ◯ Estudios de posgrado o más
- ◯ No sabe

Persona 3: ¿Cuál de las siguientes es la mejor descripción de la situación laboral actual de esta persona?	◯ Empleo a tiempo completo<br>◯ Empleo a tiempo parcial<br>◯ En busca de empleo<br>◯ Jubilado<br>◯ Ama de casa<br>◯ Estudiante<br>◯ Permiso de maternidad o paternidad<br>◯ Permiso por enfermedad<br>◯ Desempleado por discapacidad<br>◯ Otra<br>◯ No sabe
Persona 3: ¿Se considera esta persona actualmente empleado por cuenta propia (contratista independiente, trabajador esporádico (gig), etc.)?	◯ Sí<br>◯ No<br>◯ No sabe
Persona 3: ¿Trabaja esta persona actualmente en alguno de los siguientes ambientes de alto riesgo de contagio de COVID-19?	☐ Ambiente de asistencia médica (hospital, clínica, centro de urgencias, etc.)<br>☐ Ambiente residencial denso (hogar de ancianos, otro centro de asistencia de larga duración)<br>☐ Prisión o cárcel<br>☐ Establecimiento de envasado de carne<br>☐ Establecimiento de envío o distribución<br>☐ Establecimiento minorista de alto volumen (tienda de provisiones, etc.)<br>☐ No sabe
Persona 3: ¿Le ofrece el empleador a esta persona alguno de los siguientes beneficios en su empleo principal actual?	☐ Permiso por enfermedad con goce de sueldo<br>☐ Vacaciones o permiso personal con goce de sueldo<br>☐ Seguro de salud<br>☐ Seguro de discapacidad<br>☐ Plan de jubilación<br>☐ Otro<br>☐ No sabe<br>((Seleccione todo lo que corresponda.))
Persona 3: En una escala de 0 (definitivamente no va a suceder) a 10 (definitivamente va a suceder), ¿qué tan probable es que esta persona pierda su empleo debido a la pandemia de COVID-19?	__________________________________
Persona 3: En una escala de 0 (definitivamente no va a suceder) a 10 (definitivamente va a suceder), ¿qué tan probable es que a esta persona se le asignen menos horas de trabajo debido a la pandemia de COVID-19?	__________________________________

	Todo el tiempo (100%)	La mayor parte del tiempo (75%)	La mitad del tiempo (50%)	Menos de la mitad del tiempo (25%)	nunca (0%)	Nunca
Persona 3: Actualmente ¿con qué frecuencia se le exige a esta persona que trabaje fuera de su residencia?	◯	◯	◯	◯	◯	◯

Persona 3: Actualmente, ¿con qué frecuencia se encuentra esta persona físicamente cerca de sus compañeros de trabajo mientras trabaja fuera de su residencia? ◯ ◯ ◯ ◯ ◯ ◯

Persona 3: Actualmente, ¿con qué frecuencia se encuentra esta persona físicamente cerca de sus clientes mientras trabaja fuera de su residencia ◯ ◯ ◯ ◯ ◯ ◯

Persona 3: ¿Piensa esta persona vacunarse contra la COVID-19 cuando se ofrezca una vacuna?

◯ Sí
◯ No
◯ No sabe

Persona 3: En las últimas dos semanas, ¿ha tenido esta persona algún síntoma de COVID-19 (tos, fiebre, dificultad para respirar, fatiga, dolores de cuerpo, diarrea, goteo nasal o pérdida del sentido del olfato o del gusto)?

◯ Sí
◯ No
◯ No sabe

Persona 3: ¿Cuándo le comenzaron los síntomas de COVID-19 a esta persona?

____

Persona 3: En vista de sus síntomas, ¿le preocupa a esta persona la posibilidad de tener COVID-19?

◯ Sí
◯ No
◯ No sabe

Persona 3: ¿Fue esta persona objeto de prejuicio o discriminación debido a sus síntomas?

◯ Sí
◯ No
◯ No sabe

Persona 3: ¿Qué hizo esta persona en vista de sus síntomas?

☐ Nada
☐ Tomó medicamentos sin receta (ibuprofeno, acetaminofén, etc.)
☐ Consultó por teléfono a un proveedor de asistencia médica
☐ Fue al consultorio de un proveedor de asistencia médica
☐ Fue a una clínica o una farmacia minorista
☐ Fue a un centro de urgencias (FASTMed, etc.)
☐ Fue a la sala de emergencias
☐ Fue ingresado al hospital
☐ Otra cosa
☐ No sabe
((Seleccione todo lo que corresponda.))

Persona 3: ¿Qué otra cosa hizo esta persona en vista de sus síntomas?

____

Persona 3: ¿Le dijo un proveedor de asistencia médica a esta persona que era posible que tuviera COVID-19?

◯ Sí
◯ No
◯ No sabe

Persona 3: Si a esta persona le hicieron una prueba de COVID-19 en vista de sus síntomas, ¿cuál fue el resultado?	◯ Pendiente<br>◯ Positivo<br>◯ Negativo<br>◯ No concluyente<br>◯ No se hizo la prueba<br>◯ No sabe
Persona 3: ¿Cuántos días estuvo hospitalizada esta persona?	________________________________
Persona 3: ¿Se le hicieron a esta persona las siguientes intervenciones durante su hospitalización?	☐ Oxígeno adicional por la nariz<br>☐ Tratamiento en la Unidad de Cuidados Intensivos (Intensive Care Unit, ICU)<br>☐ Ventilación mecánica (intubación o tubo de respiración)<br>☐ No sabe
Persona 3: ¿Ha vuelto esta persona a su salud normal?	◯ Sí<br>◯ No<br>◯ No sabe
Persona 3: ¿Cuáles de las siguientes medidas tomó esta persona para proteger a sus amigos y familiares después de que comenzaron sus síntomas?	☐ Ponerse mascarilla con mayor frecuencia<br>☐ Lavarse las manos con agua y jabón con mayor frecuencia<br>☐ Limpiarse las manos con desinfectante con mayor frecuencia<br>☐ Aislarse en su casa con mayor frecuencia<br>☐ Quedarse en casa con mayor frecuencia<br>☐ Ponerse guantes desechables con mayor frecuencia<br>☐ No sabe

Por cada persona adicional en su hogar, proporcione la siguiente informacion.

Persona 4: ¿Cuál es su relación con esta persona?	◯ Pareja o cónyuge<br>◯ Hijo o hija<br>◯ Padre o madre<br>◯ Hermano o hermana<br>◯ Otro familiar<br>◯ Proveedor de cuidado infantil u otros cuidados en casa<br>◯ Otra
Persona 4: Especifique su relación con esta persona.	________________________________
Persona 4: ¿Qué edad tiene esta persona?	________________________________<br>((Especifique la edad en años))
Persona 4: ¿Cuál es el sexo de esta persona?	◯ Femenino<br>◯ Masculino

Persona 4: ¿Cuál es la raza de esta persona?	☐ Indio americano o nativo de Alaska<br>☐ Asiático<br>☐ Negro o afroestadounidense<br>☐ Nativo de Hawái o de las islas del Pacífico<br>☐ Blanco<br>☐ Otra<br>☐ No sabe<br>((Seleccione todo lo que corresponda.))
Persona 4: ¿Cuál es la identidad étnica de esta persona?	◯ Hispano o latino<br>◯ Ni hispano ni latino<br>◯ Otra<br>◯ No sabe
Persona 4: ¿Cuál es el nivel de educación de esta persona?	◯ No tiene educación formal<br>◯ Kínder a 8° grado<br>◯ Estudios de educación secundaria<br>◯ Equivalencia de educación secundaria (GED)<br>◯ Diploma de educación secundaria<br>◯ Estudios de educación superior<br>◯ Título universitario<br>◯ Estudios de posgrado o más<br>◯ No sabe
Persona 4: ¿Cuál de las siguientes es la mejor descripción de la situación laboral actual de esta persona?	◯ Empleo a tiempo completo<br>◯ Empleo a tiempo parcial<br>◯ En busca de empleo<br>◯ Jubilado<br>◯ Ama de casa<br>◯ Estudiante<br>◯ Permiso de maternidad o paternidad<br>◯ Permiso por enfermedad<br>◯ Desempleado por discapacidad<br>◯ Otra<br>◯ No sabe
Persona 4: ¿Se considera esta persona actualmente empleado por cuenta propia (contratista independiente, trabajador esporádico (gig), etc.)?	◯ Sí<br>◯ No<br>◯ No sabe
Persona 4: ¿Trabaja esta persona actualmente en alguno de los siguientes ambientes de alto riesgo de contagio de COVID-19?	☐ Ambiente de asistencia médica (hospital, clínica, centro de urgencias, etc.)<br>☐ Ambiente residencial denso (hogar de ancianos, otro centro de asistencia de larga duración)<br>☐ Prisión o cárcel<br>☐ Establecimiento de envasado de carne<br>☐ Establecimiento de envío o distribución<br>☐ Establecimiento minorista de alto volumen (tienda de provisiones, etc.)<br>☐ No sabe
Persona 4: ¿Le ofrece el empleador a esta persona alguno de los siguientes beneficios en su empleo principal actual?	☐ Permiso por enfermedad con goce de sueldo<br>☐ Vacaciones o permiso personal con goce de sueldo<br>☐ Seguro de salud<br>☐ Seguro de discapacidad<br>☐ Plan de jubilación<br>☐ Otro<br>☐ No sabe<br>((Seleccione todo lo que corresponda.))

Persona 4: En una escala de 0 (definitivamente no va a suceder) a 10 (definitivamente va a suceder), ¿qué tan probable es que esta persona pierda su empleo debido a la pandemia de COVID-19? __________________________________

Persona 4: En una escala de 0 (definitivamente no va a suceder) a 10 (definitivamente va a suceder), ¿qué tan probable es que a esta persona se le asignen menos horas de trabajo debido a la pandemia de COVID-19? __________________________________

	Todo el tiempo (100%)	La mayor parte del tiempo (75%)	La mitad del tiempo (50%)	Menos de la mitad del tiempo (25%)	nunca (0%)	Nunca
Persona 4: Actualmente ¿con qué frecuencia se le exige a esta persona que trabaje fuera de su residencia?	◯	◯	◯	◯	◯	◯
Persona 4: Actualmente, ¿con qué frecuencia se encuentra esta persona físicamente cerca de sus compañeros de trabajo mientras trabaja fuera de su residencia?	◯	◯	◯	◯	◯	◯
Persona 4: Actualmente, ¿con qué frecuencia se encuentra esta persona físicamente cerca de sus clientes mientras trabaja fuera de su residencia	◯	◯	◯	◯	◯	◯

Persona 4: ¿Piensa esta persona vacunarse contra la COVID-19 cuando se ofrezca una vacuna?
- ◯ Sí
- ◯ No
- ◯ No sabe

Persona 4: En las últimas dos semanas, ¿ha tenido esta persona algún síntoma de COVID-19 (tos, fiebre, dificultad para respirar, fatiga, dolores de cuerpo, diarrea, goteo nasal o pérdida del sentido del olfato o del gusto)?
- ◯ Sí
- ◯ No
- ◯ No sabe

Persona 4: ¿Cuándo le comenzaron los síntomas de COVID-19 a esta persona? __________________________________

Persona 4: En vista de sus síntomas, ¿le preocupa a esta persona la posibilidad de tener COVID-19?
- ◯ Sí
- ◯ No
- ◯ No sabe

Persona 4: ¿Fue esta persona objeto de prejuicio o discriminación debido a sus síntomas?
- ◯ Sí
- ◯ No
- ◯ No sabe

Persona 4: ¿Qué hizo esta persona en vista de sus síntomas?	☐ Nada<br>☐ Tomó medicamentos sin receta (ibuprofeno, acetaminofén, etc.)<br>☐ Consultó por teléfono a un proveedor de asistencia médica<br>☐ Fue al consultorio de un proveedor de asistencia médica<br>☐ Fue a una clínica o una farmacia minorista<br>☐ Fue a un centro de urgencias (FASTMed, etc.)<br>☐ Fue a la sala de emergencias<br>☐ Fue ingresado al hospital<br>☐ Otra cosa<br>☐ No sabe<br>((Seleccione todo lo que corresponda.))
Persona 4: ¿Qué otra cosa hizo esta persona en vista de sus síntomas?	________________________________
Persona 4: ¿Le dijo un proveedor de asistencia médica a esta persona que era posible que tuviera COVID-19?	◯ Sí<br>◯ No<br>◯ No sabe
Persona 4: Si a esta persona le hicieron una prueba de COVID-19 en vista de sus síntomas, ¿cuál fue el resultado?	◯ Pendiente<br>◯ Positivo<br>◯ Negativo<br>◯ No concluyente<br>◯ No se hizo la prueba<br>◯ No sabe
Persona 4: ¿Cuántos días estuvo hospitalizada esta persona?	________________________________
Persona 4: ¿Se le hicieron a esta persona las siguientes intervenciones durante su hospitalización?	☐ Oxígeno adicional por la nariz<br>☐ Tratamiento en la Unidad de Cuidados Intensivos (Intensive Care Unit, ICU)<br>☐ Ventilación mecánica (intubación o tubo de respiración)<br>☐ No sabe
Persona 4: ¿Ha vuelto esta persona a su salud normal?	◯ Sí<br>◯ No<br>◯ No sabe
Persona 4: ¿Cuáles de las siguientes medidas tomó esta persona para proteger a sus amigos y familiares después de que comenzaron sus síntomas?	☐ Ponerse mascarilla con mayor frecuencia<br>☐ Lavarse las manos con agua y jabón con mayor frecuencia<br>☐ Limpiarse las manos con desinfectante con mayor frecuencia<br>☐ Aislarse en su casa con mayor frecuencia<br>☐ Quedarse en casa con mayor frecuencia<br>☐ Ponerse guantes desechables con mayor frecuencia<br>☐ No sabe

Por cada persona adicional en su hogar, proporcione la siguiente informacion.

Persona 5: ¿Cuál es su relación con esta persona?	◯ Pareja o cónyuge<br>◯ Hijo o hija<br>◯ Padre o madre<br>◯ Hermano o hermana<br>◯ Otro familiar<br>◯ Proveedor de cuidado infantil u otros cuidados en casa<br>◯ Otra
Persona 5: Especifique su relación con esta persona.	__________________________
Persona 5: ¿Qué edad tiene esta persona?	__________________________<br>((Especifique la edad en años))
Persona 5: ¿Cuál es el sexo de esta persona?	◯ Femenino<br>◯ Masculino
Persona 5: ¿Cuál es la raza de esta persona?	☐ Indio americano o nativo de Alaska<br>☐ Asiático<br>☐ Negro o afroestadounidense<br>☐ Nativo de Hawái o de las islas del Pacífico<br>☐ Blanco<br>☐ Otra<br>☐ No sabe<br>((Seleccione todo lo que corresponda.))
Persona 5: ¿Cuál es la identidad étnica de esta persona?	◯ Hispano o latino<br>◯ Ni hispano ni latino<br>◯ Otra<br>◯ No sabe
Persona 5: ¿Cuál es el nivel de educación de esta persona?	◯ No tiene educación formal<br>◯ Kínder a 8° grado<br>◯ Estudios de educación secundaria<br>◯ Equivalencia de educación secundaria (GED)<br>◯ Diploma de educación secundaria<br>◯ Estudios de educación superior<br>◯ Título universitario<br>◯ Estudios de posgrado o más<br>◯ No sabe
Persona 5: ¿Cuál de las siguientes es la mejor descripción de la situación laboral actual de esta persona?	◯ Empleo a tiempo completo<br>◯ Empleo a tiempo parcial<br>◯ En busca de empleo<br>◯ Jubilado<br>◯ Ama de casa<br>◯ Estudiante<br>◯ Permiso de maternidad o paternidad<br>◯ Permiso por enfermedad<br>◯ Desempleado por discapacidad<br>◯ Otra<br>◯ No sabe
Persona 5: ¿Se considera esta persona actualmente empleado por cuenta propia (contratista independiente, trabajador esporádico (gig), etc.)?	◯ Sí<br>◯ No<br>◯ No sabe

Persona 5: ¿Trabaja esta persona actualmente en alguno de los siguientes ambientes de alto riesgo de contagio de COVID-19?	☐ Ambiente de asistencia médica (hospital, clínica, centro de urgencias, etc.)<br>☐ Ambiente residencial denso (hogar de ancianos, otro centro de asistencia de larga duración)<br>☐ Prisión o cárcel<br>☐ Establecimiento de envasado de carne<br>☐ Establecimiento de envío o distribución<br>☐ Establecimiento minorista de alto volumen (tienda de provisiones, etc.)<br>☐ No sabe
Persona 5: ¿Le ofrece el empleador a esta persona alguno de los siguientes beneficios en su empleo principal actual?	☐ Permiso por enfermedad con goce de sueldo<br>☐ Vacaciones o permiso personal con goce de sueldo<br>☐ Seguro de salud<br>☐ Seguro de discapacidad<br>☐ Plan de jubilación<br>☐ Otro<br>☐ No sabe<br>((Seleccione todo lo que corresponda.))
Persona 5: En una escala de 0 (definitivamente no va a suceder) a 10 (definitivamente va a suceder), ¿qué tan probable es que esta persona pierda su empleo debido a la pandemia de COVID-19?	__________________________________
Persona 5: En una escala de 0 (definitivamente no va a suceder) a 10 (definitivamente va a suceder), ¿qué tan probable es que a esta persona se le asignen menos horas de trabajo debido a la pandemia de COVID-19?	__________________________________

	Todo el tiempo (100%)	La mayor parte del tiempo (75%)	La mitad del tiempo (50%)	Menos de la mitad del tiempo (25%)	nunca (0%)	Nunca
Persona 5: Actualmente ¿con qué frecuencia se le exige a esta persona que trabaje fuera de su residencia?	◯	◯	◯	◯	◯	◯
Persona 5: Actualmente, ¿con qué frecuencia se encuentra esta persona físicamente cerca de sus compañeros de trabajo mientras trabaja fuera de su residencia?	◯	◯	◯	◯	◯	◯
Persona 5: Actualmente, ¿con qué frecuencia se encuentra esta persona físicamente cerca de sus clientes mientras trabaja fuera de su residencia	◯	◯	◯	◯	◯	◯

Persona 5: ¿Piensa esta persona vacunarse contra la COVID-19 cuando se ofrezca una vacuna?	◯ Sí<br>◯ No<br>◯ No sabe

Persona 5: En las últimas dos semanas, ¿ha tenido esta persona algún síntoma de COVID-19 (tos, fiebre, dificultad para respirar, fatiga, dolores de cuerpo, diarrea, goteo nasal o pérdida del sentido del olfato o del gusto)?	◯ Sí<br>◯ No<br>◯ No sabe
Persona 5: ¿Cuándo le comenzaron los síntomas de COVID-19 a esta persona?	______________________
Persona 5: En vista de sus síntomas, ¿le preocupa a esta persona la posibilidad de tener COVID-19?	◯ Sí<br>◯ No<br>◯ No sabe
Persona 5: ¿Fue esta persona objeto de prejuicio o discriminación debido a sus síntomas?	◯ Sí<br>◯ No<br>◯ No sabe
Persona 5: ¿Qué hizo esta persona en vista de sus síntomas?	☐ Nada<br>☐ Tomó medicamentos sin receta (ibuprofeno, acetaminofén, etc.)<br>☐ Consultó por teléfono a un proveedor de asistencia médica<br>☐ Fue al consultorio de un proveedor de asistencia médica<br>☐ Fue a una clínica o una farmacia minorista<br>☐ Fue a un centro de urgencias (FASTMed, etc.)<br>☐ Fue a la sala de emergencias<br>☐ Fue ingresado al hospital<br>☐ Otra cosa<br>☐ No sabe<br>((Seleccione todo lo que corresponda.))
Persona 5: ¿Qué otra cosa hizo esta persona en vista de sus síntomas?	______________________
Persona 5: ¿Le dijo un proveedor de asistencia médica a esta persona que era posible que tuviera COVID-19?	◯ Sí<br>◯ No<br>◯ No sabe
Persona 5: Si a esta persona le hicieron una prueba de COVID-19 en vista de sus síntomas, ¿cuál fue el resultado?	◯ Pendiente<br>◯ Positivo<br>◯ Negativo<br>◯ No concluyente<br>◯ No se hizo la prueba<br>◯ No sabe
Persona 5: ¿Cuántos días estuvo hospitalizada esta persona?	______________________
Persona 5: ¿Se le hicieron a esta persona las siguientes intervenciones durante su hospitalización?	☐ Oxígeno adicional por la nariz<br>☐ Tratamiento en la Unidad de Cuidados Intensivos (Intensive Care Unit, ICU)<br>☐ Ventilación mecánica (intubación o tubo de respiración)<br>☐ No sabe
Persona 5: ¿Ha vuelto esta persona a su salud normal?	◯ Sí<br>◯ No<br>◯ No sabe

Persona 5: ¿Cuáles de las siguientes medidas tomó esta persona para proteger a sus amigos y familiares después de que comenzaron sus síntomas?	☐ Ponerse mascarilla con mayor frecuencia<br>☐ Lavarse las manos con agua y jabón con mayor frecuencia<br>☐ Limpiarse las manos con desinfectante con mayor frecuencia<br>☐ Aislarse en su casa con mayor frecuencia<br>☐ Quedarse en casa con mayor frecuencia<br>☐ Ponerse guantes desechables con mayor frecuencia<br>☐ No sabe

Por cada persona adicional en su hogar, proporcione la siguiente informacion.

Persona 6: ¿Cuál es su relación con esta persona?	◯ Pareja o cónyuge<br>◯ Hijo o hija<br>◯ Padre o madre<br>◯ Hermano o hermana<br>◯ Otro familiar<br>◯ Proveedor de cuidado infantil u otros cuidados en casa<br>◯ Otra
Persona 6: Especifique su relación con esta persona.	__________________________________
Persona 6: ¿Qué edad tiene esta persona?	__________________________________<br>((Especifique la edad en años))
Persona 6: ¿Cuál es el sexo de esta persona?	◯ Femenino<br>◯ Masculino
Persona 6: ¿Cuál es la raza de esta persona?	☐ Indio americano o nativo de Alaska<br>☐ Asiático<br>☐ Negro o afroestadounidense<br>☐ Nativo de Hawái o de las islas del Pacífico<br>☐ Blanco<br>☐ Otra<br>☐ No sabe<br>((Seleccione todo lo que corresponda.))
Persona 6: ¿Cuál es la identidad étnica de esta persona?	◯ Hispano o latino<br>◯ Ni hispano ni latino<br>◯ Otra<br>◯ No sabe
Persona 6: ¿Cuál es el nivel de educación de esta persona?	◯ No tiene educación formal<br>◯ Kínder a 8° grado<br>◯ Estudios de educación secundaria<br>◯ Equivalencia de educación secundaria (GED)<br>◯ Diploma de educación secundaria<br>◯ Estudios de educación superior<br>◯ Título universitario<br>◯ Estudios de posgrado o más<br>◯ No sabe

Persona 6: ¿Cuál de las siguientes es la mejor descripción de la situación laboral actual de esta persona?	◯ Empleo a tiempo completo<br>◯ Empleo a tiempo parcial<br>◯ En busca de empleo<br>◯ Jubilado<br>◯ Ama de casa<br>◯ Estudiante<br>◯ Permiso de maternidad o paternidad<br>◯ Permiso por enfermedad<br>◯ Desempleado por discapacidad<br>◯ Otra<br>◯ No sabe
Persona 6: ¿Se considera esta persona actualmente empleado por cuenta propia (contratista independiente, trabajador esporádico (gig), etc.)?	◯ Sí<br>◯ No<br>◯ No sabe
Persona 6: ¿Trabaja esta persona actualmente en alguno de los siguientes ambientes de alto riesgo de contagio de COVID-19?	☐ Ambiente de asistencia médica (hospital, clínica, centro de urgencias, etc.)<br>☐ Ambiente residencial denso (hogar de ancianos, otro centro de asistencia de larga duración)<br>☐ Prisión o cárcel<br>☐ Establecimiento de envasado de carne<br>☐ Establecimiento de envío o distribución<br>☐ Establecimiento minorista de alto volumen (tienda de provisiones, etc.)<br>☐ No sabe
Persona 6: ¿Le ofrece el empleador a esta persona alguno de los siguientes beneficios en su empleo principal actual?	☐ Permiso por enfermedad con goce de sueldo<br>☐ Vacaciones o permiso personal con goce de sueldo<br>☐ Seguro de salud<br>☐ Seguro de discapacidad<br>☐ Plan de jubilación<br>☐ Otro<br>☐ No sabe<br>((Seleccione todo lo que corresponda.))
Persona 6: En una escala de 0 (definitivamente no va a suceder) a 10 (definitivamente va a suceder), ¿qué tan probable es que esta persona pierda su empleo debido a la pandemia de COVID-19?	________________________
Persona 6: En una escala de 0 (definitivamente no va a suceder) a 10 (definitivamente va a suceder), ¿qué tan probable es que a esta persona se le asignen menos horas de trabajo debido a la pandemia de COVID-19?	________________________

	Todo el tiempo (100%)	La mayor parte del tiempo (75%)	La mitad del tiempo (50%)	Menos de la mitad del tiempo (25%)	nunca (0%)	Nunca
Persona 6: Actualmente ¿con qué frecuencia se le exige a esta persona que trabaje fuera de su residencia?	◯	◯	◯	◯	◯	◯

Persona 6: Actualmente, ¿con qué frecuencia se encuentra esta persona físicamente cerca de sus compañeros de trabajo mientras trabaja fuera de su residencia? ○ ○ ○ ○ ○ ○

Persona 6: Actualmente, ¿con qué frecuencia se encuentra esta persona físicamente cerca de sus clientes mientras trabaja fuera de su residencia ○ ○ ○ ○ ○ ○

Persona 6: ¿Piensa esta persona vacunarse contra la COVID-19 cuando se ofrezca una vacuna?

○ Sí
○ No
○ No sabe

Persona 6: En las últimas dos semanas, ¿ha tenido esta persona algún síntoma de COVID-19 (tos, fiebre, dificultad para respirar, fatiga, dolores de cuerpo, diarrea, goteo nasal o pérdida del sentido del olfato o del gusto)?

○ Sí
○ No
○ No sabe

Persona 6: ¿Cuándo le comenzaron los síntomas de COVID-19 a esta persona?

____

Persona 6: En vista de sus síntomas, ¿le preocupa a esta persona la posibilidad de tener COVID-19?

○ Sí
○ No
○ No sabe

Persona 6: ¿Fue esta persona objeto de prejuicio o discriminación debido a sus síntomas?

○ Sí
○ No
○ No sabe

Persona 6: ¿Qué hizo esta persona en vista de sus síntomas?

☐ Nada
☐ Tomó medicamentos sin receta (ibuprofeno, acetaminofén, etc.)
☐ Consultó por teléfono a un proveedor de asistencia médica
☐ Fue al consultorio de un proveedor de asistencia médica
☐ Fue a una clínica o una farmacia minorista
☐ Fue a un centro de urgencias (FASTMed, etc.)
☐ Fue a la sala de emergencias
☐ Fue ingresado al hospital
☐ Otra cosa
☐ No sabe
((Seleccione todo lo que corresponda.))

Persona 6: ¿Qué otra cosa hizo esta persona en vista de sus síntomas?

____

Persona 6: ¿Le dijo un proveedor de asistencia médica a esta persona que era posible que tuviera COVID-19?

○ Sí
○ No
○ No sabe

Persona 6: Si a esta persona le hicieron una prueba de COVID-19 en vista de sus síntomas, ¿cuál fue el resultado?	◯ Pendiente<br>◯ Positivo<br>◯ Negativo<br>◯ No concluyente<br>◯ No se hizo la prueba<br>◯ No sabe
Persona 6: ¿Cuántos días estuvo hospitalizada esta persona?	__________________________
Persona 6: ¿Se le hicieron a esta persona las siguientes intervenciones durante su hospitalización?	☐ Oxígeno adicional por la nariz<br>☐ Tratamiento en la Unidad de Cuidados Intensivos (Intensive Care Unit, ICU)<br>☐ Ventilación mecánica (intubación o tubo de respiración)<br>☐ No sabe
Persona 6: ¿Ha vuelto esta persona a su salud normal?	◯ Sí<br>◯ No<br>◯ No sabe
Persona 6: ¿Cuáles de las siguientes medidas tomó esta persona para proteger a sus amigos y familiares después de que comenzaron sus síntomas?	☐ Ponerse mascarilla con mayor frecuencia<br>☐ Lavarse las manos con agua y jabón con mayor frecuencia<br>☐ Limpiarse las manos con desinfectante con mayor frecuencia<br>☐ Aislarse en su casa con mayor frecuencia<br>☐ Quedarse en casa con mayor frecuencia<br>☐ Ponerse guantes desechables con mayor frecuencia<br>☐ No sabe

Por cada persona adicional en su hogar, proporcione la siguiente informacion.

Persona 7: ¿Cuál es su relación con esta persona?	◯ Pareja o cónyuge<br>◯ Hijo o hija<br>◯ Padre o madre<br>◯ Hermano o hermana<br>◯ Otro familiar<br>◯ Proveedor de cuidado infantil u otros cuidados en casa<br>◯ Otra
Persona 7: Especifique su relación con esta persona.	__________________________
Persona 7: ¿Qué edad tiene esta persona?	__________________________<br>((Especifique la edad en años))
Persona 7: ¿Cuál es el sexo de esta persona?	◯ Femenino<br>◯ Masculino

Persona 7: ¿Cuál es la raza de esta persona?	☐ Indio americano o nativo de Alaska<br>☐ Asiático<br>☐ Negro o afroestadounidense<br>☐ Nativo de Hawái o de las islas del Pacífico<br>☐ Blanco<br>☐ Otra<br>☐ No sabe<br>((Seleccione todo lo que corresponda.))
Persona 7: ¿Cuál es la identidad étnica de esta persona?	◯ Hispano o latino<br>◯ Ni hispano ni latino<br>◯ Otra<br>◯ No sabe
Persona 7: ¿Cuál es el nivel de educación de esta persona?	◯ No tiene educación formal<br>◯ Kínder a 8° grado<br>◯ Estudios de educación secundaria<br>◯ Equivalencia de educación secundaria (GED)<br>◯ Diploma de educación secundaria<br>◯ Estudios de educación superior<br>◯ Título universitario<br>◯ Estudios de posgrado o más<br>◯ No sabe
Persona 7: ¿Cuál de las siguientes es la mejor descripción de la situación laboral actual de esta persona?	◯ Empleo a tiempo completo<br>◯ Empleo a tiempo parcial<br>◯ En busca de empleo<br>◯ Jubilado<br>◯ Ama de casa<br>◯ Estudiante<br>◯ Permiso de maternidad o paternidad<br>◯ Permiso por enfermedad<br>◯ Desempleado por discapacidad<br>◯ Otra<br>◯ No sabe
Persona 7: ¿Se considera esta persona actualmente empleado por cuenta propia (contratista independiente, trabajador esporádico (gig), etc.)?	◯ Sí<br>◯ No<br>◯ No sabe
Persona 7: ¿Trabaja esta persona actualmente en alguno de los siguientes ambientes de alto riesgo de contagio de COVID-19?	☐ Ambiente de asistencia médica (hospital, clínica, centro de urgencias, etc.)<br>☐ Ambiente residencial denso (hogar de ancianos, otro centro de asistencia de larga duración)<br>☐ Prisión o cárcel<br>☐ Establecimiento de envasado de carne<br>☐ Establecimiento de envío o distribución<br>☐ Establecimiento minorista de alto volumen (tienda de provisiones, etc.)<br>☐ No sabe
Persona 7: ¿Le ofrece el empleador a esta persona alguno de los siguientes beneficios en su empleo principal actual?	☐ Permiso por enfermedad con goce de sueldo<br>☐ Vacaciones o permiso personal con goce de sueldo<br>☐ Seguro de salud<br>☐ Seguro de discapacidad<br>☐ Plan de jubilación<br>☐ Otro<br>☐ No sabe<br>((Seleccione todo lo que corresponda.))

Persona 7: En una escala de 0 (definitivamente no va a suceder) a 10 (definitivamente va a suceder), ¿qué tan probable es que esta persona pierda su empleo debido a la pandemia de COVID-19?

____

Persona 7: En una escala de 0 (definitivamente no va a suceder) a 10 (definitivamente va a suceder), ¿qué tan probable es que a esta persona se le asignen menos horas de trabajo debido a la pandemia de COVID-19?

____

	Todo el tiempo (100%)	La mayor parte del tiempo (75%)	La mitad del tiempo (50%)	Menos de la mitad del tiempo (25%)	nunca (0%)	Nunca
Persona 7: Actualmente ¿con qué frecuencia se le exige a esta persona que trabaje fuera de su residencia?	◯	◯	◯	◯	◯	◯
Persona 7: Actualmente, ¿con qué frecuencia se encuentra esta persona físicamente cerca de sus compañeros de trabajo mientras trabaja fuera de su residencia?	◯	◯	◯	◯	◯	◯
Persona 7: Actualmente, ¿con qué frecuencia se encuentra esta persona físicamente cerca de sus clientes mientras trabaja fuera de su residencia	◯	◯	◯	◯	◯	◯

Persona 7: ¿Piensa esta persona vacunarse contra la COVID-19 cuando se ofrezca una vacuna?

◯ Sí
◯ No
◯ No sabe

Persona 7: En las últimas dos semanas, ¿ha tenido esta persona algún síntoma de COVID-19 (tos, fiebre, dificultad para respirar, fatiga, dolores de cuerpo, diarrea, goteo nasal o pérdida del sentido del olfato o del gusto)?

◯ Sí
◯ No
◯ No sabe

Persona 7: ¿Cuándo le comenzaron los síntomas de COVID-19 a esta persona?

____

Persona 7: En vista de sus síntomas, ¿le preocupa a esta persona la posibilidad de tener COVID-19?

◯ Sí
◯ No
◯ No sabe

Persona 7: ¿Fue esta persona objeto de prejuicio o discriminación debido a sus síntomas?

◯ Sí
◯ No
◯ No sabe

Persona 7: ¿Qué hizo esta persona en vista de sus síntomas?	☐ Nada<br>☐ Tomó medicamentos sin receta (ibuprofeno, acetaminofén, etc.)<br>☐ Consultó por teléfono a un proveedor de asistencia médica<br>☐ Fue al consultorio de un proveedor de asistencia médica<br>☐ Fue a una clínica o una farmacia minorista<br>☐ Fue a un centro de urgencias (FASTMed, etc.)<br>☐ Fue a la sala de emergencias<br>☐ Fue ingresado al hospital<br>☐ Otra cosa<br>☐ No sabe<br>((Seleccione todo lo que corresponda.))
Persona 7: ¿Qué otra cosa hizo esta persona en vista de sus síntomas?	__________________________________
Persona 7: ¿Le dijo un proveedor de asistencia médica a esta persona que era posible que tuviera COVID-19?	◯ Sí<br>◯ No<br>◯ No sabe
Persona 7: Si a esta persona le hicieron una prueba de COVID-19 en vista de sus síntomas, ¿cuál fue el resultado?	◯ Pendiente<br>◯ Positivo<br>◯ Negativo<br>◯ No concluyente<br>◯ No se hizo la prueba<br>◯ No sabe
Persona 7: ¿Cuántos días estuvo hospitalizada esta persona?	__________________________________
Persona 7: ¿Se le hicieron a esta persona las siguientes intervenciones durante su hospitalización?	☐ Oxígeno adicional por la nariz<br>☐ Tratamiento en la Unidad de Cuidados Intensivos (Intensive Care Unit, ICU)<br>☐ Ventilación mecánica (intubación o tubo de respiración)<br>☐ No sabe
Persona 7: ¿Ha vuelto esta persona a su salud normal?	◯ Sí<br>◯ No<br>◯ No sabe
Persona 7: ¿Cuáles de las siguientes medidas tomó esta persona para proteger a sus amigos y familiares después de que comenzaron sus síntomas?	☐ Ponerse mascarilla con mayor frecuencia<br>☐ Lavarse las manos con agua y jabón con mayor frecuencia<br>☐ Limpiarse las manos con desinfectante con mayor frecuencia<br>☐ Aislarse en su casa con mayor frecuencia<br>☐ Quedarse en casa con mayor frecuencia<br>☐ Ponerse guantes desechables con mayor frecuencia<br>☐ No sabe

Por cada persona adicional en su hogar, proporcione la siguiente informacion.

Persona 8: ¿Cuál es su relación con esta persona?	◯ Pareja o cónyuge<br>◯ Hijo o hija<br>◯ Padre o madre<br>◯ Hermano o hermana<br>◯ Otro familiar<br>◯ Proveedor de cuidado infantil u otros cuidados en casa<br>◯ Otra
Persona 8: Especifique su relación con esta persona.	__________________________________
Persona 8: ¿Qué edad tiene esta persona?	__________________________________<br>((Especifique la edad en años))
Persona 8: ¿Cuál es el sexo de esta persona?	◯ Femenino<br>◯ Masculino
Persona 8: ¿Cuál es la raza de esta persona?	☐ Indio americano o nativo de Alaska<br>☐ Asiático<br>☐ Negro o afroestadounidense<br>☐ Nativo de Hawái o de las islas del Pacífico<br>☐ Blanco<br>☐ Otra<br>☐ No sabe<br>((Seleccione todo lo que corresponda.))
Persona 8: ¿Cuál es la identidad étnica de esta persona?	◯ Hispano o latino<br>◯ Ni hispano ni latino<br>◯ Otra<br>◯ No sabe
Persona 8: ¿Cuál es el nivel de educación de esta persona?	◯ No tiene educación formal<br>◯ Kínder a 8° grado<br>◯ Estudios de educación secundaria<br>◯ Equivalencia de educación secundaria (GED)<br>◯ Diploma de educación secundaria<br>◯ Estudios de educación superior<br>◯ Título universitario<br>◯ Estudios de posgrado o más<br>◯ No sabe
Persona 8: ¿Cuál de las siguientes es la mejor descripción de la situación laboral actual de esta persona?	◯ Empleo a tiempo completo<br>◯ Empleo a tiempo parcial<br>◯ En busca de empleo<br>◯ Jubilado<br>◯ Ama de casa<br>◯ Estudiante<br>◯ Permiso de maternidad o paternidad<br>◯ Permiso por enfermedad<br>◯ Desempleado por discapacidad<br>◯ Otra<br>◯ No sabe
Persona 8: ¿Se considera esta persona actualmente empleado por cuenta propia (contratista independiente, trabajador esporádico (gig), etc.)?	◯ Sí<br>◯ No<br>◯ No sabe

Persona 8: ¿Trabaja esta persona actualmente en alguno de los siguientes ambientes de alto riesgo de contagio de COVID-19?	☐ Ambiente de asistencia médica (hospital, clínica, centro de urgencias, etc.)<br>☐ Ambiente residencial denso (hogar de ancianos, otro centro de asistencia de larga duración)<br>☐ Prisión o cárcel<br>☐ Establecimiento de envasado de carne<br>☐ Establecimiento de envío o distribución<br>☐ Establecimiento minorista de alto volumen (tienda de provisiones, etc.)<br>☐ No sabe
Persona 8: ¿Le ofrece el empleador a esta persona alguno de los siguientes beneficios en su empleo principal actual?	☐ Permiso por enfermedad con goce de sueldo<br>☐ Vacaciones o permiso personal con goce de sueldo<br>☐ Seguro de salud<br>☐ Seguro de discapacidad<br>☐ Plan de jubilación<br>☐ Otro<br>☐ No sabe<br>((Seleccione todo lo que corresponda.))
Persona 8: En una escala de 0 (definitivamente no va a suceder) a 10 (definitivamente va a suceder), ¿qué tan probable es que esta persona pierda su empleo debido a la pandemia de COVID-19?	______________________
Persona 8: En una escala de 0 (definitivamente no va a suceder) a 10 (definitivamente va a suceder), ¿qué tan probable es que a esta persona se le asignen menos horas de trabajo debido a la pandemia de COVID-19?	______________________

	Todo el tiempo (100%)	La mayor parte del tiempo (75%)	La mitad del tiempo (50%)	Menos de la mitad del tiempo (25%)	nunca (0%)	Nunca
Persona 8: Actualmente ¿con qué frecuencia se le exige a esta persona que trabaje fuera de su residencia?	○	○	○	○	○	○
Persona 8: Actualmente, ¿con qué frecuencia se encuentra esta persona físicamente cerca de sus compañeros de trabajo mientras trabaja fuera de su residencia?	○	○	○	○	○	○
Persona 8: Actualmente, ¿con qué frecuencia se encuentra esta persona físicamente cerca de sus clientes mientras trabaja fuera de su residencia	○	○	○	○	○	○

Persona 8: ¿Piensa esta persona vacunarse contra la COVID-19 cuando se ofrezca una vacuna?	○ Sí<br>○ No<br>○ No sabe

Persona 8: En las últimas dos semanas, ¿ha tenido esta persona algún síntoma de COVID-19 (tos, fiebre, dificultad para respirar, fatiga, dolores de cuerpo, diarrea, goteo nasal o pérdida del sentido del olfato o del gusto)?	◯ Sí<br>◯ No<br>◯ No sabe
Persona 8: ¿Cuándo le comenzaron los síntomas de COVID-19 a esta persona?	__________________________
Persona 8: En vista de sus síntomas, ¿le preocupa a esta persona la posibilidad de tener COVID-19?	◯ Sí<br>◯ No<br>◯ No sabe
Persona 8: ¿Fue esta persona objeto de prejuicio o discriminación debido a sus síntomas?	◯ Sí<br>◯ No<br>◯ No sabe
Persona 8: ¿Qué hizo esta persona en vista de sus síntomas?	☐ Nada<br>☐ Tomó medicamentos sin receta (ibuprofeno, acetaminofén, etc.)<br>☐ Consultó por teléfono a un proveedor de asistencia médica<br>☐ Fue al consultorio de un proveedor de asistencia médica<br>☐ Fue a una clínica o una farmacia minorista<br>☐ Fue a un centro de urgencias (FASTMed, etc.)<br>☐ Fue a la sala de emergencias<br>☐ Fue ingresado al hospital<br>☐ Otra cosa<br>☐ No sabe<br>((Seleccione todo lo que corresponda.))
Persona 8: ¿Qué otra cosa hizo esta persona en vista de sus síntomas?	__________________________
Persona 8: ¿Le dijo un proveedor de asistencia médica a esta persona que era posible que tuviera COVID-19?	◯ Sí<br>◯ No<br>◯ No sabe
Persona 8: Si a esta persona le hicieron una prueba de COVID-19 en vista de sus síntomas, ¿cuál fue el resultado?	◯ Pendiente<br>◯ Positivo<br>◯ Negativo<br>◯ No concluyente<br>◯ No se hizo la prueba<br>◯ No sabe
Persona 8: ¿Cuántos días estuvo hospitalizada esta persona?	__________________________
Persona 8: ¿Se le hicieron a esta persona las siguientes intervenciones durante su hospitalización?	☐ Oxígeno adicional por la nariz<br>☐ Tratamiento en la Unidad de Cuidados Intensivos (Intensive Care Unit, ICU)<br>☐ Ventilación mecánica (intubación o tubo de respiración)<br>☐ No sabe
Persona 8: ¿Ha vuelto esta persona a su salud normal?	◯ Sí<br>◯ No<br>◯ No sabe

Persona 8: ¿Cuáles de las siguientes medidas tomó esta persona para proteger a sus amigos y familiares después de que comenzaron sus síntomas?

- ☐ Ponerse mascarilla con mayor frecuencia
- ☐ Lavarse las manos con agua y jabón con mayor frecuencia
- ☐ Limpiarse las manos con desinfectante con mayor frecuencia
- ☐ Aislarse en su casa con mayor frecuencia
- ☐ Quedarse en casa con mayor frecuencia
- ☐ Ponerse guantes desechables con mayor frecuencia
- ☐ No sabe

Por cada persona adicional en su hogar, proporcione la siguiente informacion.

Persona 9: ¿Cuál es su relación con esta persona?

- ◯ Pareja o cónyuge
- ◯ Hijo o hija
- ◯ Padre o madre
- ◯ Hermano o hermana
- ◯ Otro familiar
- ◯ Proveedor de cuidado infantil u otros cuidados en casa
- ◯ Otra

Persona 9: Especifique su relación con esta persona.

____

Persona 9: ¿Qué edad tiene esta persona?

____
((Especifique la edad en años))

Persona 9: ¿Cuál es el sexo de esta persona?

- ◯ Femenino
- ◯ Masculino

Persona 9: ¿Cuál es la raza de esta persona?

- ☐ Indio americano o nativo de Alaska
- ☐ Asiático
- ☐ Negro o afroestadounidense
- ☐ Nativo de Hawái o de las islas del Pacífico
- ☐ Blanco
- ☐ Otra
- ☐ No sabe

((Seleccione todo lo que corresponda.))

Persona 9: ¿Cuál es la identidad étnica de esta persona?

- ◯ Hispano o latino
- ◯ Ni hispano ni latino
- ◯ Otra
- ◯ No sabe

Persona 9: ¿Cuál es el nivel de educación de esta persona?

- ◯ No tiene educación formal
- ◯ Kínder a 8° grado
- ◯ Estudios de educación secundaria
- ◯ Equivalencia de educación secundaria (GED)
- ◯ Diploma de educación secundaria
- ◯ Estudios de educación superior
- ◯ Título universitario
- ◯ Estudios de posgrado o más
- ◯ No sabe

Persona 9: ¿Cuál de las siguientes es la mejor descripción de la situación laboral actual de esta persona?

- ◯ Empleo a tiempo completo
- ◯ Empleo a tiempo parcial
- ◯ En busca de empleo
- ◯ Jubilado
- ◯ Ama de casa
- ◯ Estudiante
- ◯ Permiso de maternidad o paternidad
- ◯ Permiso por enfermedad
- ◯ Desempleado por discapacidad
- ◯ Otra
- ◯ No sabe

Persona 9: ¿Se considera esta persona actualmente empleado por cuenta propia (contratista independiente, trabajador esporádico (gig), etc.)?

- ◯ Sí
- ◯ No
- ◯ No sabe

Persona 9: ¿Trabaja esta persona actualmente en alguno de los siguientes ambientes de alto riesgo de contagio de COVID-19?

- ☐ Ambiente de asistencia médica (hospital, clínica, centro de urgencias, etc.)
- ☐ Ambiente residencial denso (hogar de ancianos, otro centro de asistencia de larga duración)
- ☐ Prisión o cárcel
- ☐ Establecimiento de envasado de carne
- ☐ Establecimiento de envío o distribución
- ☐ Establecimiento minorista de alto volumen (tienda de provisiones, etc.)
- ☐ No sabe

Persona 9: ¿Le ofrece el empleador a esta persona alguno de los siguientes beneficios en su empleo principal actual?

- ☐ Permiso por enfermedad con goce de sueldo
- ☐ Vacaciones o permiso personal con goce de sueldo
- ☐ Seguro de salud
- ☐ Seguro de discapacidad
- ☐ Plan de jubilación
- ☐ Otro
- ☐ No sabe

((Seleccione todo lo que corresponda.))

Persona 9: En una escala de 0 (definitivamente no va a suceder) a 10 (definitivamente va a suceder), ¿qué tan probable es que esta persona pierda su empleo debido a la pandemia de COVID-19?

____

Persona 9: En una escala de 0 (definitivamente no va a suceder) a 10 (definitivamente va a suceder), ¿qué tan probable es que a esta persona se le asignen menos horas de trabajo debido a la pandemia de COVID-19?

____

	Todo el tiempo (100%)	La mayor parte del tiempo (75%)	La mitad del tiempo (50%)	Menos de la mitad del tiempo (25%)	nunca (0%)	Nunca
Persona 9: Actualmente ¿con qué frecuencia se le exige a esta persona que trabaje fuera de su residencia?	◯	◯	◯	◯	◯	◯

Persona 9: Actualmente, ¿con qué frecuencia se encuentra esta persona físicamente cerca de sus compañeros de trabajo mientras trabaja fuera de su residencia?

○ ○ ○ ○ ○ ○

Persona 9: Actualmente, ¿con qué frecuencia se encuentra esta persona físicamente cerca de sus clientes mientras trabaja fuera de su residencia

○ ○ ○ ○ ○ ○

Persona 9: ¿Piensa esta persona vacunarse contra la COVID-19 cuando se ofrezca una vacuna?

○ Sí
○ No
○ No sabe

Persona 9: En las últimas dos semanas, ¿ha tenido esta persona algún síntoma de COVID-19 (tos, fiebre, dificultad para respirar, fatiga, dolores de cuerpo, diarrea, goteo nasal o pérdida del sentido del olfato o del gusto)?

○ Sí
○ No
○ No sabe

Persona 9: ¿Cuándo le comenzaron los síntomas de COVID-19 a esta persona?

____

Persona 9: En vista de sus síntomas, ¿le preocupa a esta persona la posibilidad de tener COVID-19?

○ Sí
○ No
○ No sabe

Persona 9: ¿Fue esta persona objeto de prejuicio o discriminación debido a sus síntomas?

○ Sí
○ No
○ No sabe

Persona 9: ¿Qué hizo esta persona en vista de sus síntomas?

☐ Nada
☐ Tomó medicamentos sin receta (ibuprofeno, acetaminofén, etc.)
☐ Consultó por teléfono a un proveedor de asistencia médica
☐ Fue al consultorio de un proveedor de asistencia médica
☐ Fue a una clínica o una farmacia minorista
☐ Fue a un centro de urgencias (FASTMed, etc.)
☐ Fue a la sala de emergencias
☐ Fue ingresado al hospital
☐ Otra cosa
☐ No sabe
((Seleccione todo lo que corresponda.))

Persona 9: ¿Qué otra cosa hizo esta persona en vista de sus síntomas?

____

Persona 9: ¿Le dijo un proveedor de asistencia médica a esta persona que era posible que tuviera COVID-19?

○ Sí
○ No
○ No sabe

Persona 9: Si a esta persona le hicieron una prueba de COVID-19 en vista de sus síntomas, ¿cuál fue el resultado?	◯ Pendiente<br>◯ Positivo<br>◯ Negativo<br>◯ No concluyente<br>◯ No se hizo la prueba<br>◯ No sabe
Persona 9: ¿Cuántos días estuvo hospitalizada esta persona?	______________________
Persona 9: ¿Se le hicieron a esta persona las siguientes intervenciones durante su hospitalización?	☐ Oxígeno adicional por la nariz<br>☐ Tratamiento en la Unidad de Cuidados Intensivos (Intensive Care Unit, ICU)<br>☐ Ventilación mecánica (intubación o tubo de respiración)<br>☐ No sabe
Persona 9: ¿Ha vuelto esta persona a su salud normal?	◯ Sí<br>◯ No<br>◯ No sabe
Persona 9: ¿Cuáles de las siguientes medidas tomó esta persona para proteger a sus amigos y familiares después de que comenzaron sus síntomas?	☐ Ponerse mascarilla con mayor frecuencia<br>☐ Lavarse las manos con agua y jabón con mayor frecuencia<br>☐ Limpiarse las manos con desinfectante con mayor frecuencia<br>☐ Aislarse en su casa con mayor frecuencia<br>☐ Quedarse en casa con mayor frecuencia<br>☐ Ponerse guantes desechables con mayor frecuencia<br>☐ No sabe

Por cada persona adicional en su hogar, proporcione la siguiente informacion.

Persona 10: ¿Cuál es su relación con esta persona?	◯ Pareja o cónyuge<br>◯ Hijo o hija<br>◯ Padre o madre<br>◯ Hermano o hermana<br>◯ Otro familiar<br>◯ Proveedor de cuidado infantil u otros cuidados en casa<br>◯ Otra
Persona 10: Especifique su relación con esta persona.	______________________
Persona 10: ¿Qué edad tiene esta persona?	______________________<br>((Especifique la edad en años))
Persona 10: ¿Cuál es el sexo de esta persona?	◯ Femenino<br>◯ Masculino

Persona 10: ¿Cuál es la raza de esta persona?	☐ Indio americano o nativo de Alaska<br>☐ Asiático<br>☐ Negro o afroestadounidense<br>☐ Nativo de Hawái o de las islas del Pacífico<br>☐ Blanco<br>☐ Otra<br>☐ No sabe<br>((Seleccione todo lo que corresponda.))
Persona 10: ¿Cuál es la identidad étnica de esta persona?	◯ Hispano o latino<br>◯ Ni hispano ni latino<br>◯ Otra<br>◯ No sabe
Persona 10: ¿Cuál es el nivel de educación de esta persona?	◯ No tiene educación formal<br>◯ Kínder a 8° grado<br>◯ Estudios de educación secundaria<br>◯ Equivalencia de educación secundaria (GED)<br>◯ Diploma de educación secundaria<br>◯ Estudios de educación superior<br>◯ Título universitario<br>◯ Estudios de posgrado o más<br>◯ No sabe
Persona 10: ¿Cuál de las siguientes es la mejor descripción de la situación laboral actual de esta persona?	◯ Empleo a tiempo completo<br>◯ Empleo a tiempo parcial<br>◯ En busca de empleo<br>◯ Jubilado<br>◯ Ama de casa<br>◯ Estudiante<br>◯ Permiso de maternidad o paternidad<br>◯ Permiso por enfermedad<br>◯ Desempleado por discapacidad<br>◯ Otra<br>◯ No sabe
Persona 10: ¿Se considera esta persona actualmente empleado por cuenta propia (contratista independiente, trabajador esporádico (gig), etc.)?	◯ Sí<br>◯ No<br>◯ No sabe
Persona 10: ¿Trabaja esta persona actualmente en alguno de los siguientes ambientes de alto riesgo de contagio de COVID-19?	☐ Ambiente de asistencia médica (hospital, clínica, centro de urgencias, etc.)<br>☐ Ambiente residencial denso (hogar de ancianos, otro centro de asistencia de larga duración)<br>☐ Prisión o cárcel<br>☐ Establecimiento de envasado de carne<br>☐ Establecimiento de envío o distribución<br>☐ Establecimiento minorista de alto volumen (tienda de provisiones, etc.)<br>☐ No sabe
Persona 10: ¿Le ofrece el empleador a esta persona alguno de los siguientes beneficios en su empleo principal actual?	☐ Permiso por enfermedad con goce de sueldo<br>☐ Vacaciones o permiso personal con goce de sueldo<br>☐ Seguro de salud<br>☐ Seguro de discapacidad<br>☐ Plan de jubilación<br>☐ Otro<br>☐ No sabe<br>((Seleccione todo lo que corresponda.))

Persona 10: En una escala de 0 (definitivamente no va a suceder) a 10 (definitivamente va a suceder), ¿qué tan probable es que esta persona pierda su empleo debido a la pandemia de COVID-19? __________________________________

Persona 10: En una escala de 0 (definitivamente no va a suceder) a 10 (definitivamente va a suceder), ¿qué tan probable es que a esta persona se le asignen menos horas de trabajo debido a la pandemia de COVID-19? __________________________________

	Todo el tiempo (100%)	La mayor parte del tiempo (75%)	La mitad del tiempo (50%)	Menos de la mitad del tiempo (25%)	nunca (0%)	Nunca
Persona 10: Actualmente ¿con qué frecuencia se le exige a esta persona que trabaje fuera de su residencia?	◯	◯	◯	◯	◯	◯
Persona 10: Actualmente, ¿con qué frecuencia se encuentra esta persona físicamente cerca de sus compañeros de trabajo mientras trabaja fuera de su residencia?	◯	◯	◯	◯	◯	◯
Persona 10: Actualmente, ¿con qué frecuencia se encuentra esta persona físicamente cerca de sus clientes mientras trabaja fuera de su residencia	◯	◯	◯	◯	◯	◯

Persona 10: ¿Piensa esta persona vacunarse contra la COVID-19 cuando se ofrezca una vacuna?

◯ Sí
◯ No
◯ No sabe

Persona 10: En las últimas dos semanas, ¿ha tenido esta persona algún síntoma de COVID-19 (tos, fiebre, dificultad para respirar, fatiga, dolores de cuerpo, diarrea, goteo nasal o pérdida del sentido del olfato o del gusto)?

◯ Sí
◯ No
◯ No sabe

Persona 10: ¿Cuándo le comenzaron los síntomas de COVID-19 a esta persona? __________________________________

Persona 10: En vista de sus síntomas, ¿le preocupa a esta persona la posibilidad de tener COVID-19?

◯ Sí
◯ No
◯ No sabe

Persona 10: ¿Fue esta persona objeto de prejuicio o discriminación debido a sus síntomas?

◯ Sí
◯ No
◯ No sabe

Persona 10: ¿Qué hizo esta persona en vista de sus síntomas?	☐ Nada<br>☐ Tomó medicamentos sin receta (ibuprofeno, acetaminofén, etc.)<br>☐ Consultó por teléfono a un proveedor de asistencia médica<br>☐ Fue al consultorio de un proveedor de asistencia médica<br>☐ Fue a una clínica o una farmacia minorista<br>☐ Fue a un centro de urgencias (FASTMed, etc.)<br>☐ Fue a la sala de emergencias<br>☐ Fue ingresado al hospital<br>☐ Otra cosa<br>☐ No sabe<br>((Seleccione todo lo que corresponda.))
Persona 10: ¿Qué otra cosa hizo esta persona en vista de sus síntomas?	__________________________
Persona 10: ¿Le dijo un proveedor de asistencia médica a esta persona que era posible que tuviera COVID-19?	◯ Sí<br>◯ No<br>◯ No sabe
Persona 10: Si a esta persona le hicieron una prueba de COVID-19 en vista de sus síntomas, ¿cuál fue el resultado?	◯ Pendiente<br>◯ Positivo<br>◯ Negativo<br>◯ No concluyente<br>◯ No se hizo la prueba<br>◯ No sabe
Persona 10: ¿Cuántos días estuvo hospitalizada esta persona?	__________________________
Persona 10: ¿Se le hicieron a esta persona las siguientes intervenciones durante su hospitalización?	☐ Oxígeno adicional por la nariz<br>☐ Tratamiento en la Unidad de Cuidados Intensivos (Intensive Care Unit, ICU)<br>☐ Ventilación mecánica (intubación o tubo de respiración)<br>☐ No sabe
Persona 10: ¿Ha vuelto esta persona a su salud normal?	◯ Sí<br>◯ No<br>◯ No sabe
Persona 10: ¿Cuáles de las siguientes medidas tomó esta persona para proteger a sus amigos y familiares después de que comenzaron sus síntomas?	☐ Ponerse mascarilla con mayor frecuencia<br>☐ Lavarse las manos con agua y jabón con mayor frecuencia<br>☐ Limpiarse las manos con desinfectante con mayor frecuencia<br>☐ Aislarse en su casa con mayor frecuencia<br>☐ Quedarse en casa con mayor frecuencia<br>☐ Ponerse guantes desechables con mayor frecuencia<br>☐ No sabe

Por cada persona adicional en su hogar, proporcione la siguiente informacion.

Persona 11: ¿Cuál es su relación con esta persona?
- ◯ Pareja o cónyuge
- ◯ Hijo o hija
- ◯ Padre o madre
- ◯ Hermano o hermana
- ◯ Otro familiar
- ◯ Proveedor de cuidado infantil u otros cuidados en casa
- ◯ Otra

Persona 11: Especifique su relación con esta persona.

____

Persona 11: ¿Qué edad tiene esta persona?

____
((Especifique la edad en años))

Persona 11: ¿Cuál es el sexo de esta persona?
- ◯ Femenino
- ◯ Masculino

Persona 11: ¿Cuál es la raza de esta persona?
- ☐ Indio americano o nativo de Alaska
- ☐ Asiático
- ☐ Negro o afroestadounidense
- ☐ Nativo de Hawái o de las islas del Pacífico
- ☐ Blanco
- ☐ Otra
- ☐ No sabe

((Seleccione todo lo que corresponda.))

Persona 11: ¿Cuál es la identidad étnica de esta persona?
- ◯ Hispano o latino
- ◯ Ni hispano ni latino
- ◯ Otra
- ◯ No sabe

Persona 11: ¿Cuál es el nivel de educación de esta persona?
- ◯ No tiene educación formal
- ◯ Kínder a 8° grado
- ◯ Estudios de educación secundaria
- ◯ Equivalencia de educación secundaria (GED)
- ◯ Diploma de educación secundaria
- ◯ Estudios de educación superior
- ◯ Título universitario
- ◯ Estudios de posgrado o más
- ◯ No sabe

Persona 11: ¿Cuál de las siguientes es la mejor descripción de la situación laboral actual de esta persona?
- ◯ Empleo a tiempo completo
- ◯ Empleo a tiempo parcial
- ◯ En busca de empleo
- ◯ Jubilado
- ◯ Ama de casa
- ◯ Estudiante
- ◯ Permiso de maternidad o paternidad
- ◯ Permiso por enfermedad
- ◯ Desempleado por discapacidad
- ◯ Otra
- ◯ No sabe

Persona 11: ¿Se considera esta persona actualmente empleado por cuenta propia (contratista independiente, trabajador esporádico (gig), etc.)?
- ◯ Sí
- ◯ No
- ◯ No sabe

Persona 11: ¿Trabaja esta persona actualmente en alguno de los siguientes ambientes de alto riesgo de contagio de COVID-19?

- ☐ Ambiente de asistencia médica (hospital, clínica, centro de urgencias, etc.)
- ☐ Ambiente residencial denso (hogar de ancianos, otro centro de asistencia de larga duración)
- ☐ Prisión o cárcel
- ☐ Establecimiento de envasado de carne
- ☐ Establecimiento de envío o distribución
- ☐ Establecimiento minorista de alto volumen (tienda de provisiones, etc.)
- ☐ No sabe

Persona 11: ¿Le ofrece el empleador a esta persona alguno de los siguientes beneficios en su empleo principal actual?

- ☐ Permiso por enfermedad con goce de sueldo
- ☐ Vacaciones o permiso personal con goce de sueldo
- ☐ Seguro de salud
- ☐ Seguro de discapacidad
- ☐ Plan de jubilación
- ☐ Otro
- ☐ No sabe

((Seleccione todo lo que corresponda.))

Persona 11: En una escala de 0 (definitivamente no va a suceder) a 10 (definitivamente va a suceder), ¿qué tan probable es que esta persona pierda su empleo debido a la pandemia de COVID-19?

____

Persona 11: En una escala de 0 (definitivamente no va a suceder) a 10 (definitivamente va a suceder), ¿qué tan probable es que a esta persona se le asignen menos horas de trabajo debido a la pandemia de COVID-19?

____

	Todo el tiempo (100%)	La mayor parte del tiempo (75%)	La mitad del tiempo (50%)	Menos de la mitad del tiempo (25%)	nunca (0%)	Nunca
Persona 11: Actualmente ¿con qué frecuencia se le exige a esta persona que trabaje fuera de su residencia?	○	○	○	○	○	○
Persona 11: Actualmente, ¿con qué frecuencia se encuentra esta persona físicamente cerca de sus compañeros de trabajo mientras trabaja fuera de su residencia?	○	○	○	○	○	○
Persona 11: Actualmente, ¿con qué frecuencia se encuentra esta persona físicamente cerca de sus clientes mientras trabaja fuera de su residencia	○	○	○	○	○	○

Persona 11: ¿Piensa esta persona vacunarse contra la COVID-19 cuando se ofrezca una vacuna?

- ○ Sí
- ○ No
- ○ No sabe

Persona 11: En las últimas dos semanas, ¿ha tenido esta persona algún síntoma de COVID-19 (tos, fiebre, dificultad para respirar, fatiga, dolores de cuerpo, diarrea, goteo nasal o pérdida del sentido del olfato o del gusto)?	◯ Sí<br>◯ No<br>◯ No sabe
Persona 11: ¿Cuándo le comenzaron los síntomas de COVID-19 a esta persona?	______________________
Persona 11: En vista de sus síntomas, ¿le preocupa a esta persona la posibilidad de tener COVID-19?	◯ Sí<br>◯ No<br>◯ No sabe
Persona 11: ¿Fue esta persona objeto de prejuicio o discriminación debido a sus síntomas?	◯ Sí<br>◯ No<br>◯ No sabe
Persona 11: ¿Qué hizo esta persona en vista de sus síntomas?	☐ Nada<br>☐ Tomó medicamentos sin receta (ibuprofeno, acetaminofén, etc.)<br>☐ Consultó por teléfono a un proveedor de asistencia médica<br>☐ Fue al consultorio de un proveedor de asistencia médica<br>☐ Fue a una clínica o una farmacia minorista<br>☐ Fue a un centro de urgencias (FASTMed, etc.)<br>☐ Fue a la sala de emergencias<br>☐ Fue ingresado al hospital<br>☐ Otra cosa<br>☐ No sabe<br>((Seleccione todo lo que corresponda.))
Persona 11: ¿Qué otra cosa hizo esta persona en vista de sus síntomas?	______________________
Persona 11: ¿Le dijo un proveedor de asistencia médica a esta persona que era posible que tuviera COVID-19?	◯ Sí<br>◯ No<br>◯ No sabe
Persona 11: Si a esta persona le hicieron una prueba de COVID-19 en vista de sus síntomas, ¿cuál fue el resultado?	◯ Pendiente<br>◯ Positivo<br>◯ Negativo<br>◯ No concluyente<br>◯ No se hizo la prueba<br>◯ No sabe
Persona 11: ¿Cuántos días estuvo hospitalizada esta persona?	______________________
Persona 11: ¿Se le hicieron a esta persona las siguientes intervenciones durante su hospitalización?	☐ Oxígeno adicional por la nariz<br>☐ Tratamiento en la Unidad de Cuidados Intensivos (Intensive Care Unit, ICU)<br>☐ Ventilación mecánica (intubación o tubo de respiración)<br>☐ No sabe
Persona 11: ¿Ha vuelto esta persona a su salud normal?	◯ Sí<br>◯ No<br>◯ No sabe

Persona 11: ¿Cuáles de las siguientes medidas tomó esta persona para proteger a sus amigos y familiares después de que comenzaron sus síntomas?

- ☐ Ponerse mascarilla con mayor frecuencia
- ☐ Lavarse las manos con agua y jabón con mayor frecuencia
- ☐ Limpiarse las manos con desinfectante con mayor frecuencia
- ☐ Aislarse en su casa con mayor frecuencia
- ☐ Quedarse en casa con mayor frecuencia
- ☐ Ponerse guantes desechables con mayor frecuencia
- ☐ No sabe

Por cada persona adicional en su hogar, proporcione la siguiente informacion.

Persona 12: ¿Cuál es su relación con esta persona?

- ◯ Pareja o cónyuge
- ◯ Hijo o hija
- ◯ Padre o madre
- ◯ Hermano o hermana
- ◯ Otro familiar
- ◯ Proveedor de cuidado infantil u otros cuidados en casa
- ◯ Otra

Persona 12: Especifique su relación con esta persona.

____

Persona 12: ¿Qué edad tiene esta persona?

____
((Especifique la edad en años))

Persona 12: ¿Cuál es el sexo de esta persona?

- ◯ Femenino
- ◯ Masculino

Persona 12: ¿Cuál es la raza de esta persona?

- ☐ Indio americano o nativo de Alaska
- ☐ Asiático
- ☐ Negro o afroestadounidense
- ☐ Nativo de Hawái o de las islas del Pacífico
- ☐ Blanco
- ☐ Otra
- ☐ No sabe

((Seleccione todo lo que corresponda.))

Persona 12: ¿Cuál es la identidad étnica de esta persona?

- ◯ Hispano o latino
- ◯ Ni hispano ni latino
- ◯ Otra
- ◯ No sabe

Persona 12: ¿Cuál es el nivel de educación de esta persona?

- ◯ No tiene educación formal
- ◯ Kínder a 8° grado
- ◯ Estudios de educación secundaria
- ◯ Equivalencia de educación secundaria (GED)
- ◯ Diploma de educación secundaria
- ◯ Estudios de educación superior
- ◯ Título universitario
- ◯ Estudios de posgrado o más
- ◯ No sabe

Persona 12: ¿Cuál de las siguientes es la mejor descripción de la situación laboral actual de esta persona?	◯ Empleo a tiempo completo<br>◯ Empleo a tiempo parcial<br>◯ En busca de empleo<br>◯ Jubilado<br>◯ Ama de casa<br>◯ Estudiante<br>◯ Permiso de maternidad o paternidad<br>◯ Permiso por enfermedad<br>◯ Desempleado por discapacidad<br>◯ Otra<br>◯ No sabe
Persona 12: ¿Se considera esta persona actualmente empleado por cuenta propia (contratista independiente, trabajador esporádico (gig), etc.)?	◯ Sí<br>◯ No<br>◯ No sabe
Persona 12: ¿Trabaja esta persona actualmente en alguno de los siguientes ambientes de alto riesgo de contagio de COVID-19?	☐ Ambiente de asistencia médica (hospital, clínica, centro de urgencias, etc.)<br>☐ Ambiente residencial denso (hogar de ancianos, otro centro de asistencia de larga duración)<br>☐ Prisión o cárcel<br>☐ Establecimiento de envasado de carne<br>☐ Establecimiento de envío o distribución<br>☐ Establecimiento minorista de alto volumen (tienda de provisiones, etc.)<br>☐ No sabe
Persona 12: ¿Le ofrece el empleador a esta persona alguno de los siguientes beneficios en su empleo principal actual?	☐ Permiso por enfermedad con goce de sueldo<br>☐ Vacaciones o permiso personal con goce de sueldo<br>☐ Seguro de salud<br>☐ Seguro de discapacidad<br>☐ Plan de jubilación<br>☐ Otro<br>☐ No sabe<br>((Seleccione todo lo que corresponda.))
Persona 12: En una escala de 0 (definitivamente no va a suceder) a 10 (definitivamente va a suceder), ¿qué tan probable es que esta persona pierda su empleo debido a la pandemia de COVID-19?	______________________
Persona 12: En una escala de 0 (definitivamente no va a suceder) a 10 (definitivamente va a suceder), ¿qué tan probable es que a esta persona se le asignen menos horas de trabajo debido a la pandemia de COVID-19?	______________________

	Todo el tiempo (100%)	La mayor parte del tiempo (75%)	La mitad del tiempo (50%)	Menos de la mitad del tiempo (25%)	nunca (0%)	Nunca
Persona 12: Actualmente ¿con qué frecuencia se le exige a esta persona que trabaje fuera de su residencia?	◯	◯	◯	◯	◯	◯

Persona 12: Actualmente, ¿con qué frecuencia se encuentra esta persona físicamente cerca de sus compañeros de trabajo mientras trabaja fuera de su residencia? ◯ ◯ ◯ ◯ ◯ ◯

Persona 12: Actualmente, ¿con qué frecuencia se encuentra esta persona físicamente cerca de sus clientes mientras trabaja fuera de su residencia ◯ ◯ ◯ ◯ ◯ ◯

Persona 12: ¿Piensa esta persona vacunarse contra la COVID-19 cuando se ofrezca una vacuna?

◯ Sí
◯ No
◯ No sabe

Persona 12: En las últimas dos semanas, ¿ha tenido esta persona algún síntoma de COVID-19 (tos, fiebre, dificultad para respirar, fatiga, dolores de cuerpo, diarrea, goteo nasal o pérdida del sentido del olfato o del gusto)?

◯ Sí
◯ No
◯ No sabe

Persona 12: ¿Cuándo le comenzaron los síntomas de COVID-19 a esta persona?

____

Persona 12: En vista de sus síntomas, ¿le preocupa a esta persona la posibilidad de tener COVID-19?

◯ Sí
◯ No
◯ No sabe

Persona 12: ¿Fue esta persona objeto de prejuicio o discriminación debido a sus síntomas?

◯ Sí
◯ No
◯ No sabe

Persona 12: ¿Qué hizo esta persona en vista de sus síntomas?

☐ Nada
☐ Tomó medicamentos sin receta (ibuprofeno, acetaminofén, etc.)
☐ Consultó por teléfono a un proveedor de asistencia médica
☐ Fue al consultorio de un proveedor de asistencia médica
☐ Fue a una clínica o una farmacia minorista
☐ Fue a un centro de urgencias (FASTMed, etc.)
☐ Fue a la sala de emergencias
☐ Fue ingresado al hospital
☐ Otra cosa
☐ No sabe
((Seleccione todo lo que corresponda.))

Persona 12: ¿Qué otra cosa hizo esta persona en vista de sus síntomas?

____

Persona 12: ¿Le dijo un proveedor de asistencia médica a esta persona que era posible que tuviera COVID-19?

◯ Sí
◯ No
◯ No sabe

Persona 12: Si a esta persona le hicieron una prueba de COVID-19 en vista de sus síntomas, ¿cuál fue el resultado?

- ◯ Pendiente
- ◯ Positivo
- ◯ Negativo
- ◯ No concluyente
- ◯ No se hizo la prueba
- ◯ No sabe

Persona 12: ¿Cuántos días estuvo hospitalizada esta persona?

____

Persona 12: ¿Se le hicieron a esta persona las siguientes intervenciones durante su hospitalización?

- ☐ Oxígeno adicional por la nariz
- ☐ Tratamiento en la Unidad de Cuidados Intensivos (Intensive Care Unit, ICU)
- ☐ Ventilación mecánica (intubación o tubo de respiración)
- ☐ No sabe

Persona 12: ¿Ha vuelto esta persona a su salud normal?

- ◯ Sí
- ◯ No
- ◯ No sabe

Persona 12: ¿Cuáles de las siguientes medidas tomó esta persona para proteger a sus amigos y familiares después de que comenzaron sus síntomas?

- ☐ Ponerse mascarilla con mayor frecuencia
- ☐ Lavarse las manos con agua y jabón con mayor frecuencia
- ☐ Limpiarse las manos con desinfectante con mayor frecuencia
- ☐ Aislarse en su casa con mayor frecuencia
- ☐ Quedarse en casa con mayor frecuencia
- ☐ Ponerse guantes desechables con mayor frecuencia
- ☐ No sabe

Complete la siguiente informacion sobre su salud mental y bienestar

A su juicio, ¿qué tan grave es para usted personalmente la pandemia de COVID-19 en este momento?

- ◯ Muy grave
- ◯ Más o menos grave
- ◯ No demasiado grave
- ◯ Nada grave

A su juicio, ¿qué tan grave es actualmente la pandemia de COVID-19 para los integrantes de su comunidad?

- ◯ Muy grave
- ◯ Más o menos grave
- ◯ No demasiado grave
- ◯ Nada grave

A su juicio, ¿qué tan grave es actualmente la pandemia de COVID-19 para los habitantes de Estados Unidos?

- ◯ Muy grave
- ◯ Más o menos grave
- ◯ No demasiado grave
- ◯ Nada grave

A su juicio, ¿qué tan grave es actualmente la pandemia de COVID-19 para los habitantes de todo el mundo?

- ◯ Muy grave
- ◯ Más o menos grave
- ◯ No demasiado grave
- ◯ Nada grave

En las últimas dos semanas, ¿con qué frecuencia ha encontrado usted medios de comunicación, hechos o tendencias virales, conversaciones en línea, fotos o videos en línea en los cuales una persona como usted es amenazada o lastimada?

- ◯ Nunca
- ◯ Aproximadamente una vez al mes
- ◯ Aproximadamente una vez a la semana
- ◯ Aproximadamente una vez al día
- ◯ Varias veces al día

En las últimas dos semanas, ¿con qué frecuencia ha encontrado usted medios de comunicación, hechos o tendencias virales, conversaciones en línea, fotos o videos en línea en los cuales se presentan estereotipos negativos de personas como usted como si fueran verdaderos?

- ◯ Nunca
- ◯ Aproximadamente una vez al mes
- ◯ Aproximadamente una vez a la semana
- ◯ Aproximadamente una vez al día
- ◯ Varias veces al día

La pandemia de COVID-19 puede cuasar desafios por algunas personas sin importar a que esten infectados. Que tan preocupado/a esta usted sobre cada una de la siguiente?

	Nada	No mucho	Un poco	Mucho
Recibir la asistencia médica que necesita (incluyendo la asistencia de salud mental)	◯	◯	◯	◯
Tener un lugar para vivir	◯	◯	◯	◯
Poder interactuar con otras personas	◯	◯	◯	◯
Conseguir comida, agua y otros suministros domésticos	◯	◯	◯	◯
Conseguir medicamentos	◯	◯	◯	◯
Tener transporte para llegar adonde tiene que ir	◯	◯	◯	◯
Cuidar de su familia y a sus amigos	◯	◯	◯	◯

Durante las ultimas 2 semanas, que tan seguido ha tenido molestias debido a los siguientes problemas?

	Nunca	Varios días	Más de la mitad de los días	Casi todos los días
Nerviosismo o ansiedad	◯	◯	◯	◯
Incapacidad de dejar de preocuparse o controlar las preocupaciones	◯	◯	◯	◯
Exceso de preocupación por diferentes cosas	◯	◯	◯	◯
Dificultad para tranquilizarse	◯	◯	◯	◯
Tanta inquietud que le es difícil estarse quieto	◯	◯	◯	◯
Facilidad para molestarse o irritarse	◯	◯	◯	◯
Temor de que algo horrible podría pasar	◯	◯	◯	◯

¿Cuánta dificultad le han causado estos problemas para hacer su trabajo, ocuparse de las cosas de su casa o llevarse bien con los demás?

- ◯ Nada de dificultad
- ◯ Un poco de dificultad
- ◯ Bastante dificultad
- ◯ Mucha dificultad

Durante las ultimas 2 semanas, que tan seguido ha tenido los siguentes sentimientos y comportamientos?

	Rara vez o nunca (menos de 1 día)	Pocas veces (1-2 días)	De vez en cuando (3-4 días)	La mayor parte del tiempo o todo el tiempo (5-7 días)
Me ha molestado lo que normalmente no me molesta.	◯	◯	◯	◯
No he tenido ganas de comer; he tenido poco apetito.	◯	◯	◯	◯
He sentido que no podía librarme de la tristeza, ni siquiera con la ayuda de mis familiares o amigos.	◯	◯	◯	◯
He sentido que yo no soy tan bueno como otras personas.	◯	◯	◯	◯
Me ha costado concentrarme en lo que hacía.	◯	◯	◯	◯
Me he sentido deprimido.	◯	◯	◯	◯
He sentido que todo lo que hacía implicaba un gran esfuerzo.	◯	◯	◯	◯
He sentido desesperanza respecto al futuro.	◯	◯	◯	◯
He pensado que mi vida es un fracaso.	◯	◯	◯	◯
He tenido miedo.	◯	◯	◯	◯
No he dormido bien.	◯	◯	◯	◯
No he sido feliz.	◯	◯	◯	◯
He hablado menos de lo habitual.	◯	◯	◯	◯
Me he sentido solo.	◯	◯	◯	◯
Las personas han sido poco amistosas.	◯	◯	◯	◯
No he disfrutado de la vida.	◯	◯	◯	◯
He tenido episodios de llanto.	◯	◯	◯	◯
Me he sentido triste.	◯	◯	◯	◯
He sentido que no les agrado a los demás.	◯	◯	◯	◯
No he podido "ponerme en marcha".	◯	◯	◯	◯

¿Cómo llenó esta encuesta?

- ◯ En una computadora (portátil o de escritorio)
- ◯ En un dispositivo móvil (tableta o teléfono celular)
- ◯ Por teléfono con un entrevistador
- ◯ Otra manera

Community Prevention and COVID-19 Testing (ComPACT) Study

Baseline Survey

Baseline Questionnaire

Please complete the survey below.

Thank you!

This first set of questions asks about your household information.

What is your sex?
- ◯ Male
- ◯ Female
- ◯ Other

How old are you?

____

What is your primary race?
- ◯ White/Caucasian
- ◯ Black or African American
- ◯ Asian
- ◯ American Indian or Alaska Native
- ◯ Native Hawaiian or other Pacific Islander
- ◯ Other

What is your ethnicity?
- ◯ Hispanic or Latinx
- ◯ Not Hispanic or Latinx

What is the highest level of education or schooling you have completed?
- ◯ Never attended school
- ◯ Kindergarten through 8th grade
- ◯ Some high school
- ◯ High school equivalency (GED)
- ◯ High school graduate
- ◯ Some college
- ◯ College graduate
- ◯ Graduate school
- ◯ Prefer not to answer

What was your approximate total household income last year from all sources, before taxes?
- ◯ Less than $10,000
- ◯ $10,000-$19,999
- ◯ $20,000-$29,999
- ◯ $30,000-$49,999
- ◯ $50,000-$74,999
- ◯ $75,000 or more
- ◯ Prefer not to answer
- ◯ Don't know

How many total people (including yourself) CURRENTLY live in your household?

____

How has the number of people living in your household changed since March 1, 2020 when the COVID-19 pandemic began in North Carolina?
- ◯ Increased by more than two people
- ◯ Increased by one or two people
- ◯ The number has not changed
- ◯ Decreased by one or two people
- ◯ Decreased by more than two people

How many of the people in your household are below the age of 18?

____

How many of the people in your household are below the age of 10?

____

How long have you lived at your current address?

- ◯ 0-3 years
- ◯ 4-6 years
- ◯ 7-10 years
- ◯ 10+ years

How long have you lived in Pitt county?

- ◯ 0-3 years
- ◯ 4-6 years
- ◯ 7-10 years
- ◯ 10+ years

Which of the following best describes your home's wastewater disposal system?

- ◯ Greenville Utilities Commission sewer system
- ◯ Other sewer system
- ◯ Septic system
- ◯ Other
- ◯ Don't know

Which utility company provides your home with sewer services?

____

Briefly describe your wastewater system

____

Section 2. COVID-19 impact

This next section asks about how the COVID-19 pandemic has impacted you. We will ask about employment, food availability, income, and savings.

	0	1	2	3	4	5	6	7	8	9	10
On a scale of 0 (not at all informed) to 10 (very well informed), how well informed are you about the COVID-19 pandemic?	◯	◯	◯	◯	◯	◯	◯	◯	◯	◯	◯

Which of the following best describes your current employment?

- ◯ Working full time (30 or more hours/week)
- ◯ Working part time (< 30 hours/week)
- ◯ On furlough or administrative leave
- ◯ Looking for work/employment
- ◯ Unemployed but not looking for work
- ◯ Retired
- ◯ Homemaker
- ◯ Student
- ◯ On maternity/paternity leave
- ◯ On illness/sick leave
- ◯ On disability
- ◯ Other

Do you currently consider yourself self-employed (including as an independent contractor or gig-economy worker)?

- ◯ Yes
- ◯ No

Of the job (or jobs) that you currently have, which description best describes your main job (i.e. the job you spend the most hours at, or the job at which you have worked the longest)?

- ◯ Management, business, or finance
- ◯ Computer, mathematics, data science, architecture, or engineering
- ◯ Life, physical, or social science (including research)
- ◯ Community or social services
- ◯ Legal services
- ◯ Education, training, or library
- ◯ Art, design, entertainment, sports, or media
- ◯ Health care
- ◯ Protective services (including first responders)
- ◯ Food preparation or service
- ◯ Building and grounds cleaning or maintenance
- ◯ Personal care or service
- ◯ Non-food sales or retail
- ◯ Farming, fishing, or forestry
- ◯ Construction or extraction
- ◯ Installation, maintenance, or repair
- ◯ Factory production, assembly, or processing, including food
- ◯ Transportation or material moving
- ◯ Military
- ◯ Other

How would you describe your main job?

____

Do you currently work in any of the following high-risk settings for COVID-19 transmission

- ◯ Do not work in high-risk setting
- ◯ Health care setting (hospital, clinic, urgent care, etc.)
- ◯ Dense residential setting (nursing home, other long-term care facility)
- ◯ Prison or Jail
- ◯ Meatpacking facility
- ◯ Shipping or distribution facility
- ◯ High-volume retail facility (grocery store, etc)

How often are you currently required to work from outside of the home?

- ◯ Always
- ◯ Often
- ◯ Sometimes
- ◯ Hardly ever
- ◯ Never

When you go to work, where is the primary location of the work you are doing?

- ◯ Greenville
- ◯ Outside of Greenville
- ◯ Both

In which town outside of Greenville do you primarily work?

____

Approximately what proportion of your work is in Greenville?

- ◯ 0% to 10%
- ◯ 11% to 20%
- ◯ 21% to 30%
- ◯ 31% to 40%
- ◯ 41% to 50%
- ◯ 51% to 60%
- ◯ 61% to 70%
- ◯ 71% to 80%
- ◯ 81% to 90%
- ◯ 91% to 100%

In your main job, does your current employer offer you any of the following benefits? (select all that apply)

- ☐ Paid sick leave
- ☐ Paid vacation/personal leave
- ☐ Health insurance
- ☐ Disability insurance
- ☐ Retirement plan

	0	1	2	3	4	5	6	7	8	9	10
On a scale of 0 (definitely not going to happen) to 10 (definitely going to happen), how likely is it that you will lose your job because of the COVID-19 pandemic?	○	○	○	○	○	○	○	○	○	○	○

Since March 1, 2020, have you received fewer work hours due to COVID-19?

- ○ Yes
- ○ No

	0	1	2	3	4	5	6	7	8	9	10
On a scale of 0 (definitely not going to happen) to 10 (definitely going to happen), how likely is it that you will receive fewer works hours at your job because of the COVID-19 pandemic?	○	○	○	○	○	○	○	○	○	○	○

This next set of questions asks about other changes that have occurred because of the COVID-19 pandemic.

	Often True	Sometimes true	Never true	I don't know
In the past two weeks, the food your household bought just didn't last (not enough food), and you didn't have money to get more.	○	○	○	○
In the past two weeks, you couldn't afford to eat balanced meals.	○	○	○	○
In the past two weeks, you or others in your household cut the size of your meals or skip meals because there wasn't enough money for food	○	○	○	○
In the past two weeks, you ate less than you felt you should because there wasn't enough money for food	○	○	○	○

In the past two weeks, you were hungry but didn't eat because there wasn't enough money for food	◯	◯	◯	◯
In the past two weeks, you were worried or stressed about having enough money to buy nutritious meals	◯	◯	◯	◯

How do you think your total household income will change this year due to the COVID-19 crisis?

- ◯ Decrease significantly
- ◯ Decrease slightly
- ◯ Stay the same
- ◯ Increase slightly
- ◯ Increase significantly

	0	1	2	3	4	5	6	7	8	9	10
On a scale of 0 (definitely not going to happen) to 10 (definitely going to happen), how likely do you think it is that your household will run out of money in the next 3 months?	◯	◯	◯	◯	◯	◯	◯	◯	◯	◯	◯

How often is your family getting help with running necessary errands, such as getting groceries or medications?

- ◯ Always
- ◯ most of the time
- ◯ half of the time
- ◯ less than half of the time
- ◯ never

How difficult has it been for your household to adjust to changes in child care or having to home school?

- ◯ not difficult
- ◯ somewhat difficult
- ◯ very difficult
- ◯ extremely difficult

Before the COVID-19 pandemic, did you have regular caregiving responsibilities for someone who didn't live in your household (ex. elderly parent or sibling who you regularly visited and supported, etc.)?

- ◯ Yes
- ◯ No

How difficult has it been to continue providing this care due to the COVID-19 pandemic?

- ◯ not difficult
- ◯ somewhat difficult
- ◯ very difficult
- ◯ extremely difficult

Section 3. Mental health and wellness

This next set of questions ask about your mental health and wellness since March 1, 2020 when the COVID-19 pandemic began in North Carolina.

How serious a problem would you say the COVID-19 pandemic is right now for you personally?

- ◯ Very serious
- ◯ Somewhat serious
- ◯ Not too serious
- ◯ Not at all serious
- ◯ Don't know

How serious a problem would you say the COVID-19 pandemic is right now for people in your community?

- ◯ Very serious
- ◯ Somewhat serious
- ◯ Not too serious
- ◯ Not at all serious
- ◯ Don't know

How serious a problem would you say the COVID-19 pandemic is right now for people in the United States?

- ◯ Very serious
- ◯ Somewhat serious
- ◯ Not too serious
- ◯ Not at all serious
- ◯ Don't know

How serious a problem would you say the COVID-19 pandemic is right now for people around the world?

- ◯ Very serious
- ◯ Somewhat serious
- ◯ Not too serious
- ◯ Not at all serious
- ◯ Don't know

The COVID-19 pandemic may cause challenges for some people regardless of whether they are infected. How concerned are you about each of the following things?

	Very concerned	Somewhat concerned	Not at all concerned	Don't know
Getting the healthcare I need (including care for mental health)	◯	◯	◯	◯
Having a place to live	◯	◯	◯	◯
Being able to interact with other people	◯	◯	◯	◯
Getting food, water, and other household supplies	◯	◯	◯	◯
Getting medication	◯	◯	◯	◯
Having transportation to get where I need to go	◯	◯	◯	◯
Caring for my family and friends	◯	◯	◯	◯

How significant of a source of stress is the COVID-19 pandemic in your life right now?

- ◯ Very significant
- ◯ Somewhat significant
- ◯ Not very significant
- ◯ Not at all significant

During the last two weeks, how often have you been bothered by the following problems?

	Not at all	Several days	More than half the days	Nearly every day
Feeling nervous, anxious, or on edge	◯	◯	◯	◯
Not being able to stop or control worrying	◯	◯	◯	◯
Worrying too much about different things	◯	◯	◯	◯
Trouble relaxing	◯	◯	◯	◯
Being so restless that it's hard to sit still	◯	◯	◯	◯
Becoming easily annoyed or irritable	◯	◯	◯	◯
Feeling afraid as if something awful might happen	◯	◯	◯	◯

GAD corrected sum score ______________________________

GAD score dichotomized (1 for >=10; 0 for < 10) ______________________________

How difficult have these made it for you to do your work, take care of things at home, or get along with other people?

◯ Not difficult
◯ Somewhat difficult
◯ Very difficult
◯ Extremely difficult

During the last two weeks, how often have you experienced the following behaviors and feelings?

	Rarely or none of the time (less than 1 day)	Some or a little of the time (1-2 days)	Occasionally or a moderate amount of the time (3-4 days)	Most or all of the time (5-7 days)
I was bothered by things that usually don't bother me	◯	◯	◯	◯
I did not feel like eating; my appetite was poor	◯	◯	◯	◯
I felt that I could not shake off the blues even with help from my family or friends	◯	◯	◯	◯
I felt I was just as good as other people	◯	◯	◯	◯
I had trouble keeping my mind on what I was doing	◯	◯	◯	◯

I felt depressed	○	○	○	○
I felt that everything I did was an effort	○	○	○	○
I felt hopeful about the future	○	○	○	○
I thought my life had been a failure	○	○	○	○
I felt fearful	○	○	○	○
My sleep was restless	○	○	○	○
I was happy	○	○	○	○
I talked less than usual	○	○	○	○
I felt lonely	○	○	○	○
People were unfriendly	○	○	○	○
I enjoyed life	○	○	○	○
I had crying spells	○	○	○	○
I felt sad	○	○	○	○
I felt that people disliked me	○	○	○	○
I could not "get going"	○	○	○	○

CES-D score (negative items) ______________________

CES-D (positive items) ______________________

CES-D score, corrected ______________________

CES-D score dichotomized (1 for >=16; 0 for < 16) ______________________

During the last two weeks, how often have you experienced the following due to the COVID-19 pandemic?

I believed that my job was putting me at great risk
○ Yes
○ No
○ I was not working during the last two weeks

I felt extra stress at work
○ Yes
○ No
○ I was not working during the last two weeks

I was afraid of falling ill with COVID-19
○ Yes
○ No

I felt I had little control over whether I would get infected or not
○ Yes
○ No

I thought I would be unlikely to survive if I were to get COVID-19
○ Yes
○ No

I thought about resigning because of COVID-19	◯ Yes<br>◯ No<br>◯ I was not working during the last two weeks
I was afraid I would pass COVID-19 on to others	◯ Yes<br>◯ No
My family and friends were worried that they might get infected through me	◯ Yes<br>◯ No
People avoided my family because of my work	◯ Yes<br>◯ No
I was willing to accept the risks at work because I wanted to help COVID-19 patients	◯ Yes<br>◯ No<br>◯ I have not been working with COVID-19 patients

Section 4. COVID-19 prevention measures

The next set of questions asks you about different activities you may do to protect yourself or others from COVID-19. Depending on your responses to prior questions, this may include your work or your daily life.

	Always	Often	Sometimes	Hardly ever	Never
How regularly are you in close physical contact with co-workers in your current work outside of the home?	◯	◯	◯	◯	◯
How regularly are you in close physical contact with clients in your current work outside of the home?	◯	◯	◯	◯	◯
How often do you have access to disposable gloves during your current work outside of the home?	◯	◯	◯	◯	◯
How often do you use disposable gloves during your current work outside of the home?	◯	◯	◯	◯	◯
How often do you have access to a face mask during your current work outside of the home?	◯	◯	◯	◯	◯
How often do you use a face mask during your current work outside of the home?	◯	◯	◯	◯	◯

How often do you wash your hands with soap and water at your current work outside of the home?	◯	◯	◯	◯	◯
How often do you sanitize your hands with hand sanitizer at your current work outside of the home?	◯	◯	◯	◯	◯

How worried are you that you will be exposed to COVID-19 at your current work outside of the home?

◯ Extremely worried
◯ Moderately worried
◯ Slightly worried
◯ Not at all worried

In the last two weeks, how often have you done the following things to protect yourself and others from COVID-19?

	Always	Most of the time	Half of the time	Less than half of the time	Never
Worn a face mask when out in public	◯	◯	◯	◯	◯
Washed hands and/or used sanitizer frequently	◯	◯	◯	◯	◯
Stayed at least 6 feet away from others	◯	◯	◯	◯	◯
Avoided large gatherings, public spaces, or crowds	◯	◯	◯	◯	◯
Avoided contact with people who could be high risk	◯	◯	◯	◯	◯
Avoided eating at restaurants	◯	◯	◯	◯	◯
Avoided getting take-out from restaurants	◯	◯	◯	◯	◯
Worked or studied at home instead of going into an office/classroom	◯	◯	◯	◯	◯
Avoided shaking hands or touching people	◯	◯	◯	◯	◯
Stayed home when I am sick	◯	◯	◯	◯	◯
Wiped down surfaces with disinfectant	◯	◯	◯	◯	◯
Cancelled or postponed planned travel for work	◯	◯	◯	◯	◯
Cancelled or postponed travel for pleasure	◯	◯	◯	◯	◯

Cancelled or postponed personal or social activities	○	○	○	○	○
Cancelled a doctor's appointment	○	○	○	○	○
Stockpiled food or water	○	○	○	○	○
Followed government guidelines or rules to shelter in place. Specifically, staying at home and limiting contacts with other people	○	○	○	○	○

Over the past 30 days, have you traveled outside of Pitt County?
○ Yes
○ No

Where did you travel?
____

Between the period November 21, 2020 and January 4, 2021, did you spend time with friends or relatives outside your immediate family for the holidays?
○ Yes
○ No

Did you travel outside of Pitt county to spend time with friends or relatives outside your immediate family for the holidays?
○ Yes
○ No

Did you spend at least one night at the home or other location of friends or relatives outside your immediate family for the holidays?
○ Yes
○ No

How long was your trip to spend time with friends or relatives outside your immediate family for the holiday? If you took multiple trips during the period November 21, 2020 and January 4, 2021, indicate the total amount of time spent with relatives outside your immediate family or friends.
○ Just one overnight trip
○ Two or three nights
○ Four or five nights
○ Six or seven nights
○ Eight or more nights

During the time spent with relatives outside your extended family or friends for the holidays, how often have you done the following things to protect yourself and others from COVID-19?

	Always	Most of the time	Half of the time	Less than half of the time	Never
Worn a face mask	○	○	○	○	○
Washed hands and/or used sanitizer frequently	○	○	○	○	○
Stayed at least 6 feet away from others	○	○	○	○	○
Avoided large gatherings, public spaces, or crowds	○	○	○	○	○

Avoided contact with people who could be high risk	◯	◯	◯	◯	◯
Avoided eating at restaurants	◯	◯	◯	◯	◯
Avoided getting take-out from restaurants	◯	◯	◯	◯	◯

In general, which of the following best describes what you have done differently to protect yourself and others from COVID-19 over the last two weeks?

- ◯ I've done nothing differently
- ◯ I've done things differently less than half the time
- ◯ I've done things differently about half the time
- ◯ I've done things differently most of the time
- ◯ I've done things differently all of the time

Thinking about your household as a whole over the last two weeks, how often has everyone been staying at home and avoiding interacting with others aside from getting groceries?

- ◯ Always
- ◯ most of the time
- ◯ half of the time
- ◯ less than half of the time
- ◯ never

Do you plan to get a vaccine for COVID-19 when one becomes available?

- ◯ Yes
- ◯ Maybe
- ◯ No

Have you received one or more of the COVID-19 vaccine doses?

- ◯ Yes
- ◯ No
- ◯ Don't know

Have you received one or two doses of the vaccine?

- ◯ One dose only
- ◯ Two doses
- ◯ Don't know

What was the date of your first dose?

____

What was the date of your second dose?

____

Do you know which COVID-19 vaccine you received?

- ◯ Pfizer/BioNTech (mRNA type vaccine) - 2 doses given 3 weeks apart
- ◯ Moderna (mRNA type vaccine) - 2 doses given 4 weeks apart
- ◯ Don't know

Section 5. Health Care and Physical Health

This section asks about having health insurance, any chronic conditions you have, and smoking, alcohol, and exercise behaviors.

Are you covered by any type of medical or health insurance (including private insurance, insurance you purchased, Medicare, Medicaid, or any other health insurance program)?

- ◯ Yes
- ◯ No

What is the primary health insurance coverage that you have?	◯ Private health insurance through a job or school<br>◯ Insurance purchased through a state or federal health insurance exchange such as healthcare.gov<br>◯ Insurance purchased directly through a health plan or insurance company<br>◯ Medicare<br>◯ Medi-Gap<br>◯ Medicaid<br>◯ Military health care (TRICARE/VA/CHAMP-VA)<br>◯ Indian Health Service<br>◯ Other
Please specify what other health insurance you have	__________________________________
About how long have you had your current health insurance coverage?	◯ Less than 6 months<br>◯ More than 6 months but less than 1 year<br>◯ More than 1 year but less than 3 years<br>◯ More than 3 years<br>◯ Don't know
Was there any time in past 12 month that you did NOT have ANY health insurance coverage?	◯ Yes<br>◯ No<br>◯ Don't know
About how long have you been without health insurance coverage	◯ Less than 6 months<br>◯ More than 6 months but less than 1 year<br>◯ More than 1 year but less than 3 years<br>◯ More than 3 years<br>◯ Don't know
Are you currently pregnant?	◯ Yes<br>◯ No<br>◯ Don't know
Have you ever been tested for COVID-19 (do not include being tested for this study)?	◯ Yes<br>◯ No<br>◯ Don't know
What was the reason for being testing for COVID-19?	◯ Had symptoms of COVID-19<br>◯ Close contact of person who had COVID-19<br>◯ Employer provided testing<br>◯ Other
What was the reason you were tested for COVID-19?	__________________________________
Was one (or more) of these tests positive for COVID-19?	◯ Yes<br>◯ No<br>◯ Don't know
What was the date of the most recent positive COVID-19 test?	__________________________________
What was the date of your most recent COVID-19 test?	__________________________________

What was the result of your most recent COVID-19 test?

- ◯ Positive
- ◯ Negative
- ◯ Inconclusive
- ◯ Still waiting on result

This next section asks about symptoms you may have experienced.

During the last two weeks, have you experienced any of the following symptoms?

	Yes	No	Don't know
Fever (measured by thermometer/self-diagnosed)	◯	◯	◯
Cough (new or worsening)	◯	◯	◯
Shortness of breath (new or worsening)	◯	◯	◯
Fatigue (new tiredness doing normal activities)	◯	◯	◯
Body aches	◯	◯	◯
Headache	◯	◯	◯
Diarrhea	◯	◯	◯
Sore throat	◯	◯	◯
Itchy, pink, or painful eyes	◯	◯	◯
Runny nose or congestion	◯	◯	◯
Changes in your sense of smell or taste	◯	◯	◯
New rash	◯	◯	◯
Repeated shaking with chills	◯	◯	◯

When did these symptoms first start to occur?

____

Because of these symptoms, were you worried that you may have COVID-19?

- ◯ Yes
- ◯ No
- ◯ Don't know

Because of these symptoms, did you experience any bias or discrimination due to your symptoms?

- ◯ Yes
- ◯ No
- ◯ Don't know

Which of the following did you do to protect your friends and family afteryour symptoms began? (select all that apply)

- ☐ Wore a mask more frequently
- ☐ Washed hands with soap and water more frequently
- ☐ Used hand sanitizer more frequently
- ☐ Isolated yourself in your home more frequently
- ☐ Stayed home more frequently
- ☐ Wore disposable gloves more frequently

What did you do in response to the symptoms reported above? (select all that apply)	☐ nothing<br>☐ took over the counter medication (ibuprofen, acetimenophen, etc.)<br>☐ communicated with a health care provider over the phone<br>☐ visited a health care provider's office<br>☐ visited a retail clinic or pharmacy<br>☐ visited urgent care (FASTMed, etc.)<br>☐ visited the emergency room<br>☐ was admitted to the hospital<br>☐ other
If you specified other, please explain	__________________________________
How many days were you admitted in the hospital?	__________________________________
Did you require any of the following interventions while admitted in the hospital? (select all that apply)	◯ Extra oxygen in your nose<br>◯ Treatment in the intensive care unit (ICU)<br>◯ Mechanical ventilation (intubation or a breathing tube)
Because of your symptoms, were you told that you may have been infected with COVID-19?	◯ Yes<br>◯ No<br>◯ Don't know
Because of your symptoms, were you tested for COVID-19 (do not include being tested as part of this study)?	◯ Yes<br>◯ No<br>◯ Don't know
Was one or more of these tests positive for COVID-19?	◯ Yes<br>◯ No<br>◯ Don't know
What was the date of the most recent positive COVID-19 test?	__________________________________
What was the date of your most recent COVID-19 test?	__________________________________
What was the result of your most recent COVID-19 test?	◯ Positive<br>◯ Negative<br>◯ Inconclusive<br>◯ Still waiting on result
Have you returned to your normal health at this time?	◯ Yes<br>◯ No<br>◯ Don't know
During the last two weeks, were you tested for COVID for any other reason?	◯ Yes<br>◯ No<br>◯ Don't know
What was the reason for being tested for COVID-19?	◯ Had symptoms of COVID-19<br>◯ Close contact of person who had COVID-19<br>◯ Employer provided testing<br>◯ Other

What was the reason for being tested for COVID-19?

____

Was one or more of these tests positive for COVID-19?

- ◯ Yes
- ◯ No
- ◯ Don't know

What was the date of the most recent positive COVID-19 test?

____

What was the date of your most recent COVID-19 test?

____

What was the result of your most recent COVID-19 test?

- ◯ Positive
- ◯ Negative
- ◯ Inconclusive
- ◯ Still waiting on result

If someone in the household became sick with COVID-19, how well would the household be able to isolate them (let them stay in their own room and limit contact with them)?

- ◯ extremely well
- ◯ very well
- ◯ pretty well
- ◯ not very well

This next section asks about smoking and alcohol use

Have you EVER smoked cigarettes, cigars, or a pipe on a daily basis?

- ◯ Yes
- ◯ No

How old were you when you first started to smoke fairly regularly?

____

Do you CURRENTLY smoke cigarettes, cigars, or a pipe on a daily basis?

- ◯ Yes
- ◯ No

What is the average number of cigarettes smoked per day since you began smoking?

____

What is the average number of cigars smoked per day since you began smoking?

____

What is the average number of bowls of tobacco smoked per day since you began smoking?

____

How many years has it been since you quit smoking?

____

When you were smoking, what was the average number of cigarettes smoked per day?

____

When you were smoking, what was the average number of cigars smoked per day?

____

When you were smoking, what was the average number of bowls of tobacco smoked per day?

____

Have you EVER used electronic cigarettes (e-cigs, vaping)?	◯ Yes<br>◯ No
How old were you when you first started to use e-cigs fairly regularly?	__________________
Do you CURRENTLY use electronic cigarettes (e-cigs, vaping)?	◯ Yes<br>◯ No
What is the average number of cartridges vaped per day since you began smoking?	__________________
What is the typical size of your e-cig cartridge?	__________________
What is the average number of e-cig (or other vaping product) puffs you inhale per day?	◯ 0-25<br>◯ 26-50<br>◯ 51-75<br>◯ 76-100<br>◯ 101-125<br>◯ 126-150<br>◯ 151-175<br>◯ 176-200<br>◯ 201-225<br>◯ 226-250<br>◯ 251 or more
How many years has it been since you quit using e-cigs?	__________________
When you were using e-cigs, what was the average number of cartridges vaped per day?	__________________
When you were using e-cigs, what was the typical size of your e-cig cartridge?	__________________
When you were using e-cigs, what was the average number of e-cig (or other vaping product) puffs you inhaled per day?	◯ 0-25<br>◯ 26-50<br>◯ 51-75<br>◯ 76-100<br>◯ 101-125<br>◯ 126-150<br>◯ 151-175<br>◯ 176-200<br>◯ 201-225<br>◯ 226-250<br>◯ 251 or more
Did you EVER drink alcohol at least once a week?	◯ Yes<br>◯ No
How old were when you first started to drink alcohol fairly regularly?	__________________
Do you CURRENTLY drink alcohol a least once a week?	◯ Yes<br>◯ No

Think specifically about the last 30 days, including today. During the last 30 days, on how many days did you drink on or more drinks of an alcoholic beverage?	________________________
On the days that you drank during the past 30 days, how many drinks did you usually have each day?	________________________
How many years has it been since you quit drinking alcohol?	________________________
Think specifically about the last 30 days, including today. During the last 30 days, on how many days did you drink one or more drinks of an alcoholic beverage?	________________________
On the days that you drank during the past 30 days, how many drinks did you usually have each day?	________________________

This section asks about other behaviors

Did you receive a flu vaccine yet this flu season (2020-2021)?	◯ Yes<br>◯ No<br>◯ Don't know
At least once a week, do you engage in regular activity like walking, jogging, bicycling, swimming, etc. long enough to work up a sweat, get your heart thumping, or get out of breath?	◯ Yes<br>◯ No
When you are exercising in your usual fashion, how would you rate your average level of exertion (degree of effort)?	◯ Easy / Warm-up<br>◯ Medium (can hold a conversation) / Aerobic Development<br>◯ Hard (but you can push yourself to continue) / Aerobic Endurance<br>◯ Very Hard (cannot hold a conversation) / Anaerobic Endurance<br>◯ Extremely Hard (out of breath, your body wants to stop the exercise) / Speed, Power
On average, how many days per week do you engage in this kind of exercise?	________________________
On average, how many minutes per day do you engage in this kind of exercise?	________________________
During the past MONTH, how often did you have MILK, either to drink or on cereal? This may include skim, no-fat, low-fat, whole milk, buttermilk, lactose-free milk, and chocolate or other flavored milks. Do NOT include small amounts of milk in coffee or tea, cream or soy milk.	◯ Never<br>◯ 1-3 times last month<br>◯ 1-2 times per week<br>◯ 3-4 times per week<br>◯ 5-6 times per week<br>◯ 1 time per day<br>◯ 2 times per day<br>◯ 3 times per day<br>◯ 4 times per day<br>◯ 5 or more times per day<br>◯ Don't know

In the past MONTH, think about how much Green leafy vegetables you have eaten (Cooked dark green vegetables like spinach, collards, mustard greens, kale, turnip greens, okra, broccoli, cabbage, and raw cabbage slaw). About how many servings did you have?	◯ None<br>◯ Only a few servings last month<br>◯ 1-2 servings per week<br>◯ 3-4 servings per week<br>◯ 5-6 servings per week<br>◯ 1 serving per day<br>◯ 2 serving per day<br>◯ 3 serving per day<br>◯ 4 serving per day<br>◯ 5 or more servings per day<br>◯ Don't know
What size were the servings, on average?	◯ Small<br>◯ Medium<br>◯ Large<br>◯ Don't know
Do you currently take a multivitamin supplement that contains Vitamin D?	◯ Yes<br>◯ No<br>◯ Don't know
How often do you take the multivitamin?	◯ Every day or almost every day<br>◯ 3 to 5 days each week<br>◯ 1 or 2 days each week<br>◯ A few times each month<br>◯ Don't know
Do you currently take a Vitamin D supplement (not in a multivitamin)?	◯ Yes<br>◯ No<br>◯ Don't know
How often do you take the Vitamin D supplement?	◯ Every day or almost every day<br>◯ 3 tp 5 days each week<br>◯ 1 or 2 days each week<br>◯ A few times each month<br>◯ Don't know
In the past MONTH, on average, how many hours were you outside per day between 10:00am and 4:00pm on WEEKDAYS (Monday through Friday)	◯ 30 minutes or less<br>◯ 31 minutes to 1 hour<br>◯ More than 1 hour but less than 2 hours<br>◯ More than 2 hour but less than 3 hours<br>◯ More than 3 hour but less than 4 hours<br>◯ More than 4 hour but less than 5 hours<br>◯ More than 5 hour but less than 6 hours<br>◯ Don't know
In the past MONTH, on average, how many hours were you outside per day between 10:00am and 4:00p on WEEKEND days (Saturday and Sunday)	◯ 30 minutes or less<br>◯ 31 minutes to 1 hour<br>◯ More than 1 hour but less than 2 hours<br>◯ More than 2 hour but less than 3 hours<br>◯ More than 3 hour but less than 4 hours<br>◯ More than 4 hour but less than 5 hours<br>◯ More than 5 hour but less than 6 hours<br>◯ Don't know

Section 6. Other individuals in your household.

This final section briefly asks about COVID-19 exposure and symptoms of any one else in your household

Does anyone else in your household currently work outside of the home?
- ○ Yes
- ○ No

How often do others in your household work from outside of the home?
- ○ Always
- ○ Often
- ○ Sometimes
- ○ Hardly ever
- ○ Never

Does anyone else in your household currently work in any of the following high-risk settings for COVID-19 transmission?
- ○ Does not work in high-risk setting
- ○ Health care setting (hospital, clinic, urgent care, etc.)
- ○ Dense residential setting (nursing home, other long-term care facility)
- ○ Prison or Jail
- ○ Meatpacking facility
- ○ Shipping or distribution facility
- ○ High-volume retail facility (grocery store, etc)

During the last two weeks, has anyone in your household (not including you) experienced any of the following symptoms ? (select all that apply)
- ☐ Fever (measured by thermometer/self-diagnosed)
- ☐ Cough (new or worsening)
- ☐ Shortness of breath (new or worsening)
- ☐ Fatigue (new tiredness doing normal activities)
- ☐ Body aches
- ☐ Headache
- ☐ Diarrhea
- ☐ Sore throat
- ☐ Itchy, pink, or painful eyes
- ☐ Runny nose or congestion
- ☐ Changes in sense of smell or taste
- ☐ New rash
- ☐ Repeated shaking with chills
- ☐ No one else had any of these symptoms

How many individuals in your household (not including you) experienced one or more of these symptoms?

____

How old is/are the individual(s) who experienced symptoms (not including you)? (select all that apply)
- ☐ 0 to 4 years old
- ☐ 5 to 9 years old
- ☐ 10 to 17 years old
- ☐ 18 to 24 years old
- ☐ 25 to 49 years old
- ☐ 50 to 64 years old
- ☐ 65 to 74 years old
- ☐ 75 years or older

When did the earliest symptoms begin?

____

Because of these symptoms, were you worried that they may have COVID-19?
- ○ Yes
- ○ No
- ○ Don't know

Which of the following did the individual(s) with symptoms do to protect others after the symptoms began? (select all that apply)	☐ Wore a mask more frequently<br>☐ Washed hands with soap and water more frequently<br>☐ Used hand sanitizer more frequently<br>☐ Isolated yourself in your home more frequently<br>☐ Stayed home more frequently<br>☐ Wore disposable gloves more frequently
Which of the following did the individual(s) with symptoms do in response to the symptoms reported above? (select all that apply)	☐ nothing<br>☐ took over the counter medication (ibuprofen, acetimenophen, etc.)<br>☐ communicated with a health care provider over the phone<br>☐ visited a health care provider's office<br>☐ visited a retail clinic or pharmacy<br>☐ visited urgent care (FASTMed, etc.)<br>☐ visited the emergency room<br>☐ was admitted to the hospital<br>☐ other
If you other, please explain	__________________________________
How many days was the individual(s) with symptoms admitted in the hospital?	__________________________________
Did the individual(s) with symptoms require any of the following interventions while admitted in the hospital? (select all that apply)	◯ Extra oxygen in your nose<br>◯ Treatment in the intensive care unit (ICU)<br>◯ Mechanical ventilation (intubation or a breathing tube)
Was the individual(s) with symptoms ever told that they may have been infected with COVID-19?	◯ Yes<br>◯ No<br>◯ Don't know
Was the individual(s) with symptoms ever tested for COVID-19?	◯ Yes<br>◯ No<br>◯ Don't know
Was one or more of the individual's tests positive for COVID-19?	◯ Yes<br>◯ No<br>◯ Don't know
What was the date of the individual's most recent positive COVID-19 test?	__________________________________
What was the date of the individual's most recent COVID-19 test?	__________________________________
What was the result of the individual's most recent COVID-19 test?	◯ Positive<br>◯ Negative<br>◯ Inconclusive<br>◯ Still waiting on result
Have the individual(s) with symptoms returned to their normal health at this time?	◯ Yes<br>◯ No<br>◯ Don't know

During the last two weeks, was the individual tested for COVID for any other reason?	◯ Yes<br>◯ No<br>◯ Don't know
What was the reason for the individual being tested for COVID-19?	◯ Had symptoms of COVID-19<br>◯ Close contact of person who had COVID-19<br>◯ Employer provided testing<br>◯ Other
What was the reason for the individual being tested for COVID-19?	__________________________________
Was one or more of the individual's tests positive for COVID-19?	◯ Yes<br>◯ No<br>◯ Don't know
What was the date of the individual's most recent positive COVID-19 test?	__________________________________
What was the date of the individual's most recent COVID-19 test?	__________________________________
What was the result of the individual's most recent COVID-19 test?	◯ Positive<br>◯ Negative<br>◯ Inconclusive<br>◯ Still waiting on result

Please click the 'Submit' button to finish the survey

Community Prevention and COVID-19 Testing (ComPACT) Study

Biweekly Survey

Follow-Up Survey

Thank you for taking the time to complete this follow-up survey.

If completing on a smartphone, some questions may be easier to read if the phone is turned sideways.

Has your address changed in the last 14 days?	◯ Yes<br>◯ No
What is your new address (including city and ZIP code)?	__________________________
Remind us, how many total people (including yourself) CURRENTLY live in your household?	__________________________
How many of the people in your household are below the age of 18?	__________________________
How many of the people in your household are below the age of 10?	__________________________
Which of the following best describes your home's wastewater disposal system?	◯ Greenville Utilities Commission sewer system<br>◯ Other sewer system<br>◯ Septic system<br>◯ Other<br>◯ Don't know
Which utility company provides your home with sewer services?	__________________________
Briefly describe your wastewater system	__________________________
Which of the following best describes your current employment?	◯ Working full time (30 or more hours/week)<br>◯ Working part time (< 30 hours/week)<br>◯ On furlough or administrative leave<br>◯ Looking for work/employment<br>◯ Unemployed but not looking for work<br>◯ Retired<br>◯ Homemaker<br>◯ Student<br>◯ On maternity/paternity leave<br>◯ On illness/sick leave<br>◯ On disability<br>◯ Other
How often are you currently required to work from outside of the home?	◯ Always<br>◯ Often<br>◯ Sometimes<br>◯ Hardly ever<br>◯ Never
Has your employment situation changed in the last 14 days (new job, lost job, or major change in hours or duties)?	◯ Yes<br>◯ No

Do you currently consider yourself self-employed (including as an independent contractor or gig-economy worker)?

- ◯ Yes
- ◯ No

Of the job (or jobs) that you currently have, which description best describes your main job (i.e. the job you spend the most hours at, or the job at which you have worked the longest)?

- ◯ Management, business, or finance
- ◯ Computer, mathematics, data science, architecture, or engineering
- ◯ Life, physical, or social science (including research)
- ◯ Community or social services
- ◯ Legal services
- ◯ Education, training, or library
- ◯ Art, design, entertainment, sports, or media
- ◯ Health care
- ◯ Protective services (including first responders)
- ◯ Food preparation or service
- ◯ Building and grounds cleaning or maintenance
- ◯ Personal care or service
- ◯ Non-food sales or retail
- ◯ Farming, fishing, or forestry
- ◯ Construction or extraction
- ◯ Installation, maintenance, or repair
- ◯ Factory production, assembly, or processing, including food
- ◯ Transportation or material moving
- ◯ Military
- ◯ Other

How would you describe your main job?

____

Do you currently work in any of the following high-risk settings for COVID-19 transmission

- ◯ Do not work in high-risk setting
- ◯ Health care setting (hospital, clinic, urgent care, etc.)
- ◯ Dense residential setting (nursing home, other long-term care facility)
- ◯ Prison or Jail
- ◯ Meatpacking facility
- ◯ Shipping or distribution facility
- ◯ High-volume retail facility (grocery store, etc)

When you go to work, where is the primary location of the work you are doing?

- ◯ Greenville
- ◯ Outside of Greenville
- ◯ Both

In which town outside of Greenville do you primarily work?

____

Approximately what proportion of your work is in Greenville?

- ◯ 0% to 10%
- ◯ 11% to 20%
- ◯ 21% to 30%
- ◯ 31% to 40%
- ◯ 41% to 50%
- ◯ 51% to 60%
- ◯ 61% to 70%
- ◯ 71% to 80%
- ◯ 81% to 90%
- ◯ 91% to 100%

In your main job, does your current employer offer you any of the following benefits? (select all that apply)	☐ Paid sick leave<br>☐ Paid vacation/personal leave<br>☐ Health insurance<br>☐ Disability insurance<br>☐ Retirement plan

	0	1	2	3	4	5	6	7	8	9	10
On a scale of 0 (definitely not going to happen) to 10 (definitely going to happen), how likely is it that you will lose your job because of the COVID-19 pandemic?	○	○	○	○	○	○	○	○	○	○	○

	0	1	2	3	4	5	6	7	8	9	10
On a scale of 0 (definitely not going to happen) to 10 (definitely going to happen), how likely is it that you will receive fewer works hours at your job because of the COVID-19 pandemic?	○	○	○	○	○	○	○	○	○	○	○

	0	1	2	3	4	5	6	7	8	9	10
On a scale of 0 (definitely not going to happen) to 10 (definitely going to happen), how likely do you think it is that your household will run out of money in the next 3 months?	○	○	○	○	○	○	○	○	○	○	○

How often is your family getting help with running necessary errands, such as getting groceries or medications?	○ Always<br>○ most of the time<br>○ half of the time<br>○ less than half of the time<br>○ never

Section 3. Mental health and wellness

This next set of questions ask about your mental health and wellness since March 1, 2020 when the COVID-19 pandemic began in North Carolina.

How serious a problem would you say the COVID-19 pandemic is right now for you personally?

- ◯ Very serious
- ◯ Somewhat serious
- ◯ Not too serious
- ◯ Not at all serious
- ◯ Don't know

How serious a problem would you say the COVID-19 pandemic is right now for people in your community?

- ◯ Very serious
- ◯ Somewhat serious
- ◯ Not too serious
- ◯ Not at all serious
- ◯ Don't know

How serious a problem would you say the COVID-19 pandemic is right now for people in the United States?

- ◯ Very serious
- ◯ Somewhat serious
- ◯ Not too serious
- ◯ Not at all serious
- ◯ Don't know

How serious a problem would you say the COVID-19 pandemic is right now for people around the world?

- ◯ Very serious
- ◯ Somewhat serious
- ◯ Not too serious
- ◯ Not at all serious
- ◯ Don't know

The COVID-19 pandemic may cause challenges for some people regardless of whether they are infected. How concerned are you about each of the following things?

	Very concerned	Somewhat concerned	Not at all concerned	Don't know
Getting the healthcare I need (including care for mental health)	◯	◯	◯	◯
Having a place to live	◯	◯	◯	◯
Being able to interact with other people	◯	◯	◯	◯
Getting food, water, and other household supplies	◯	◯	◯	◯
Getting medication	◯	◯	◯	◯
Having transportation to get where I need to go	◯	◯	◯	◯
Caring for my family and friends	◯	◯	◯	◯

How significant of a source of stress is the COVID-19 pandemic in your life right now?

◯ Very significant
◯ Somewhat significant
◯ Not very significant
◯ Not at all significant

During the last two weeks, how often have you been bothered by the following problems?

	Not at all	Several days	More than half the days	Nearly every day
Feeling nervous, anxious, or on edge	○	○	○	○
Not being able to stop or control worrying	○	○	○	○
Worrying too much about different things	○	○	○	○
Trouble relaxing	○	○	○	○
Being so restless that it's hard to sit still	○	○	○	○
Becoming easily annoyed or irritable	○	○	○	○
Feeling afraid as if something awful might happen	○	○	○	○

GAD score, corrected

____

GAD score dichotomized (1 for >=10; 0 for < 10)

____

How difficult have these made it for you to do your work, take care of things at home, or get along with other people?

○ Not difficult
○ Somewhat difficult
○ Very difficult
○ Extremely difficult

During the last two weeks, how often have you experienced the following behaviors and feelings?

	Rarely or none of the time (less than 1 day)	Some or a little of the time (1-2 days)	Occasionally or a moderate amount of the time (3-4 days)	Most or all of the time (5-7 days)
I was bothered by things that usually don't bother me	◯	◯	◯	◯
I did not feel like eating; my appetite was poor	◯	◯	◯	◯
I felt that I could not shake off the blues even with help from my family or friends	◯	◯	◯	◯
I felt I was just as good as other people	◯	◯	◯	◯
I had trouble keeping my mind on what I was doing	◯	◯	◯	◯
I felt depressed	◯	◯	◯	◯
I felt that everything I did was an effort	◯	◯	◯	◯
I felt hopeful about the future	◯	◯	◯	◯
I thought my life had been a failure	◯	◯	◯	◯
I felt fearful	◯	◯	◯	◯
My sleep was restless	◯	◯	◯	◯
I was happy	◯	◯	◯	◯
I talked less than usual	◯	◯	◯	◯
I felt lonely	◯	◯	◯	◯
People were unfriendly	◯	◯	◯	◯
I enjoyed life	◯	◯	◯	◯
I had crying spells	◯	◯	◯	◯
I felt sad	◯	◯	◯	◯
I felt that people disliked me	◯	◯	◯	◯
I could not "get going"	◯	◯	◯	◯

CES-D score (negative items) ______________________

CES-D (positive items) ______________________

CES-D score, corrected ______________________

CES-D score dichotomized (1 for >=16; 0 for < 16) ______________________

During the last two weeks, how often have you experienced the following due to the COVID-19 pandemic?

I believed that my job was putting me at great risk	◯ Yes<br>◯ No<br>◯ I was not working during the last two weeks
I felt extra stress at work	◯ Yes<br>◯ No<br>◯ I was not working during the last two weeks
I was afraid of falling ill with COVID-19	◯ Yes<br>◯ No
I felt I had little control over whether I would get infected or not	◯ Yes<br>◯ No
I thought I would be unlikely to survive if I were to get COVID-19	◯ Yes<br>◯ No
I thought about resigning because of COVID-19	◯ Yes<br>◯ No<br>◯ I was not working during the last two weeks
I was afraid I would pass COVID-19 on to others	◯ Yes<br>◯ No
My family and friends were worried that they might get infected through me	◯ Yes<br>◯ No
People avoided my family because of my work	◯ Yes<br>◯ No
I was willing to accept the risks at work because I wanted to help COVID-19 patients	◯ Yes<br>◯ No<br>◯ I have not been working with COVID-19 patients

Section 4. COVID-19 prevention measures

The next set of questions asks you about different activities you may do to protect yourself or others from COVID-19. Depending on your responses to prior questions, this may include your work or your daily life.

	Always	Often	Sometimes	Hardly ever	Never
How regularly are you in close physical contact with co-workers in your current work outside of the home?	◯	◯	◯	◯	◯
How regularly are you in close physical contact with clients in your current work outside of the home?	◯	◯	◯	◯	◯
How often do you have access to disposable gloves during your current work outside of the home?	◯	◯	◯	◯	◯
How often do you use disposable gloves during your current work outside of the home?	◯	◯	◯	◯	◯
How often do you have access to a face mask during your current work outside of the home?	◯	◯	◯	◯	◯
How often do you use a face mask during your current work outside of the home?	◯	◯	◯	◯	◯
How often do you wash your hands with soap and water at your current work outside of the home?	◯	◯	◯	◯	◯
How often do you sanitize your hands with hand sanitizer at your current work outside of the home?	◯	◯	◯	◯	◯

How worried are you that you will be exposed to COVID-19 at your current work outside of the home?

◯ Extremely worried
◯ Moderately worried
◯ Slightly worried
◯ Not at all worried

In the last two weeks, how often have you done the following things to protect yourself and others from COVID-19?

	Always	Most of the time	Half of the time	Less than half of the time	Never
Worn a face mask when out in public	○	○	○	○	○
Washed hands and/or used sanitizer frequently	○	○	○	○	○
Stayed at least 6 feet away from others	○	○	○	○	○
Avoided large gatherings, public spaces, or crowds	○	○	○	○	○
Avoided contact with people who could be high risk	○	○	○	○	○
Avoided eating at restaurants	○	○	○	○	○
Avoided getting take-out from restaurants	○	○	○	○	○
Worked or studied at home instead of going into an office/classroom	○	○	○	○	○
Avoided shaking hands or touching people	○	○	○	○	○
Stayed home when I am sick	○	○	○	○	○
Wiped down surfaces with disinfectant	○	○	○	○	○
Cancelled or postponed planned travel for work	○	○	○	○	○
Cancelled or postponed travel for pleasure	○	○	○	○	○
Cancelled or postponed personal or social activities	○	○	○	○	○
Cancelled a doctor's appointment	○	○	○	○	○
Stockpiled food or water	○	○	○	○	○
Followed government guidelines or rules to shelter in place. Specifically, staying at home and limiting contacts with other people	○	○	○	○	○

Over the past 30 days, have you traveled outside of Pitt County?

○ Yes
○ No

Where did you travel?

____

In general, which of the following best describes what you have done differently to protect yourself and others from COVID-19 over the last two weeks?	◯ I've done nothing differently<br>◯ I've done things differently less than half the time<br>◯ I've done things differently about half the time<br>◯ I've done things differently most of the time<br>◯ I've done things differently all of the time
Thinking about your household as a whole over the last two weeks, how often has everyone been staying at home and avoiding interacting with others aside from getting groceries?	◯ Always<br>◯ most of the time<br>◯ half of the time<br>◯ less than half of the time<br>◯ never
Do you plan to get a vaccine for COVID-19 when one becomes available?	◯ Yes<br>◯ Maybe<br>◯ No<br>◯ Already received one or more vaccine doses
In the last 2 weeks, have you received one or more of the COVID-19 vaccine doses?	◯ Yes<br>◯ No<br>◯ Don't know
Have you received one or two doses of the vaccine?	◯ One dose only<br>◯ Two doses<br>◯ Don't know
What was the date of your first dose?	________________________
What was the date of your second dose?	________________________
Do you know which COVID-19 vaccine you received?	◯ Pfizer/BioNTech (mRNA type vaccine) - 2 doses given 3 weeks apart<br>◯ Moderna (mRNA type vaccine) - 2 doses given 4 weeks apart<br>◯ Johnson & Johnson/Janssen (Adenovirus type vaccine), 1 dose<br>◯ Don't know

Section 5. Health Care and Physical Health

This section asks about having health insurance, any chronic conditions you have, and smoking, alcohol, and exercise behaviors.

Has your medical or health insurance changed in the last 14 days (changed provider, lost coverage, or gained coverage)

- ◯ Yes
- ◯ No

Are you covered by any type of medical or health insurance (including private insurance, insurance you purchased, Medicare, Medicaid, or any other health insurance program)?

- ◯ Yes
- ◯ No

What is the primary health insurance coverage that you have?

- ◯ Private health insurance through a job or school
- ◯ Insurance purchased through a state or federal health insurance exchange such as healthcare.gov
- ◯ Insurance purchased directly through a health plan or insurance company
- ◯ Medicare
- ◯ Medi-Gap
- ◯ Medicaid
- ◯ Military health care (TRICARE/VA/CHAMP-VA)
- ◯ Indian Health Service
- ◯ Other

Please specify what other health insurance you have

____

About how long have you had your current health insurance coverage?

- ◯ Less than 6 months
- ◯ More than 6 months but less than 1 year
- ◯ More than 1 year but less than 3 years
- ◯ More than 3 years
- ◯ Don't know

Was there any time in past 12 month that you did NOT have ANY health insurance coverage?

- ◯ Yes
- ◯ No
- ◯ Don't know

About how long have you been without health insurance coverage

- ◯ Less than 6 months
- ◯ More than 6 months but less than 1 year
- ◯ More than 1 year but less than 3 years
- ◯ More than 3 years
- ◯ Don't know

For women: Are you currently pregnant?

- ◯ Yes
- ◯ No
- ◯ Don't know
- ◯ Not applicable

This next section asks about symptoms you may have experienced.

During the last two weeks, have you experienced any of the following symptoms?

	Yes	No	Don't know
Fever (measured by thermometer/self-diagnosed)	◯	◯	◯
Cough (new or worsening)	◯	◯	◯
Shortness of breath (new or worsening)	◯	◯	◯
Fatigue (new tiredness doing normal activities)	◯	◯	◯
Body aches	◯	◯	◯
Headache	◯	◯	◯
Diarrhea	◯	◯	◯
Sore throat	◯	◯	◯
Itchy, pink, or painful eyes	◯	◯	◯
Runny nose or congestion	◯	◯	◯
Changes in your sense of smell or taste	◯	◯	◯
New rash	◯	◯	◯
Repeated shaking with chills	◯	◯	◯

When did these symptoms first start to occur?

____

Because of these symptoms, were you worried that you may have COVID-19?

◯ Yes
◯ No
◯ Don't know

Because of these symptoms, did you experience any bias or discrimination due to your symptoms?

◯ Yes
◯ No
◯ Don't know

Which of the following did you do to protect your friends and family afteryour symptoms began? (select all that apply)

☐ Wore a mask more frequently
☐ Washed hands with soap and water more frequently
☐ Used hand sanitizer more frequently
☐ Isolated yourself in your home more frequently
☐ Stayed home more frequently
☐ Wore disposable gloves more frequently

What did you do in response to the symptoms reported above? (select all that apply)

☐ nothing
☐ took over the counter medication (ibuprofen, acetimenophen, etc.)
☐ communicated with a health care provider over the phone
☐ visited a health care provider's office
☐ visited a retail clinic or pharmacy
☐ visited urgent care (FASTMed, etc.)
☐ visited the emergency room
☐ was admitted to the hospital
☐ other

If you specified other, please explain	__________________________
How many days were you admitted in the hospital?	__________________________
Did you require any of the following interventions while admitted in the hospital? (select all that apply)	◯ Extra oxygen in your nose<br>◯ Treatment in the intensive care unit (ICU)<br>◯ Mechanical ventilation (intubation or a breathing tube)
Have you returned to your normal health at this time?	◯ Yes<br>◯ No<br>◯ Don't know
Have you been tested for COVID-19 in the past 14 days (do not count being tested for this study)?	◯ Yes<br>◯ No<br>◯ Don't know
What was the reason for being testing for COVID-19?	◯ Had symptoms of COVID-19<br>◯ Close contact of person who had COVID-19<br>◯ Employer provided testing<br>◯ Other
What was the reason you were tested for COVID-19?	__________________________
Was one (or more) of these tests positive for COVID-19?	◯ Yes<br>◯ No<br>◯ Don't know
What was the date of the most recent positive COVID-19 test?	__________________________
What was the date of your most recent COVID-19 test?	__________________________
What was the result of your most recent COVID-19 test?	◯ Positive<br>◯ Negative<br>◯ Inconclusive<br>◯ Still waiting on result
If someone in the household became sick with COVID-19, how well would the household be able to isolate them (let them stay in their own room and limit contact with them)?	◯ extremely well<br>◯ very well<br>◯ pretty well<br>◯ not very well
At least once a week, do you engage in regular activity like walking, jogging, bicycling, swimming, etc. long enough to work up a sweat, get your heart thumping, or get out of breath?	◯ Yes<br>◯ No

When you are exercising in your usual fashion, how would you rate your average level of exertion (degree of effort)?	◯ Easy / Warm-up<br>◯ Medium (can hold a conversation) / Aerobic Development<br>◯ Hard (but you can push yourself to continue) / Aerobic Endurance<br>◯ Very Hard (cannot hold a conversation) / Anaerobic Endurance<br>◯ Extremely Hard (out of breath, your body wants to stop the exercise) / Speed, Power
On average, how many days per week do you engage in this kind of exercise?	______________________
On average, how many minutes per day do you engage in this kind of exercise?	______________________
During the past MONTH, how often did you have MILK, either to drink or on cereal? This may include skim, no-fat, low-fat, whole milk, buttermilk, lactose-free milk, non-dairy milk (soy, almond, etc), and chocolate or other flavored milks. Do NOT include small amounts of milk in coffee or tea, cream or soy milk.	◯ Never<br>◯ 1-3 times last month<br>◯ 1-2 times per week<br>◯ 3-4 times per week<br>◯ 5-6 times per week<br>◯ 1 time per day<br>◯ 2 times per day<br>◯ 3 times per day<br>◯ 4 times per day<br>◯ 5 or more times per day<br>◯ Don't know
In the past MONTH, think about how much Green leafy vegetables you have eaten (Cooked dark green vegetables like spinach, collards, mustard greens, kale, turnip greens, okra, broccoli, cabbage, and raw cabbage slaw). About how many servings did you have?	◯ None<br>◯ Only a few servings last month<br>◯ 1-2 servings per week<br>◯ 3-4 servings per week<br>◯ 5-6 servings per week<br>◯ 1 serving per day<br>◯ 2 serving per day<br>◯ 3 serving per day<br>◯ 4 serving per day<br>◯ 5 or more servings per day<br>◯ Don't know
What size were the servings, on average?	◯ Small<br>◯ Medium<br>◯ Large<br>◯ Don't know
Do you currently take a multivitamin supplement that contains Vitamin D?	◯ Yes<br>◯ No<br>◯ Don't know
How often do you take the multivitamin?	◯ Every day or almost every day<br>◯ 3 to 5 days each week<br>◯ 1 or 2 days each week<br>◯ A few times each month<br>◯ Don't know
Do you currently take a Vitamin D supplement (not in a multivitamin)?	◯ Yes<br>◯ No<br>◯ Don't know

How often do you take the Vitamin D supplement?	◯ Every day or almost every day<br>◯ 3 to 5 days each week<br>◯ 1 or 2 days each week<br>◯ A few times each month<br>◯ Don't know
In the past MONTH, on average, how many hours were you outside per day between 10:00am and 4:00pm on WEEKDAYS (Monday through Friday)	◯ 30 minutes or less<br>◯ 31 minutes to 1 hour<br>◯ More than 1 hour but less than 2 hours<br>◯ More than 2 hour but less than 3 hours<br>◯ More than 3 hour but less than 4 hours<br>◯ More than 4 hour but less than 5 hours<br>◯ More than 5 hour but less than 6 hours<br>◯ Don't know
In the past MONTH, on average, how many hours were you outside per day between 10:00am and 4:00p on WEEKEND days (Saturday and Sunday)	◯ 30 minutes or less<br>◯ 31 minutes to 1 hour<br>◯ More than 1 hour but less than 2 hours<br>◯ More than 2 hour but less than 3 hours<br>◯ More than 3 hour but less than 4 hours<br>◯ More than 4 hour but less than 5 hours<br>◯ More than 5 hour but less than 6 hours<br>◯ Don't know

Section 6. Other individuals in your household.

This final section briefly asks about COVID-19 exposure and symptoms of any one else in your household

Has anyone else in your household been vaccinated (not including you)?	◯ Yes<br>◯ No<br>◯ Don't know
How many other people in your household have been vaccinated?	__________________________
Does anyone else in your household currently work outside of the home?	◯ Yes<br>◯ No
How often do others in your household work from outside of the home?	◯ Always<br>◯ Often<br>◯ Sometimes<br>◯ Hardly ever<br>◯ Never
Does anyone else in your household currently work in any of the following high-risk settings for COVID-19 transmission?	◯ Does not work in high-risk setting<br>◯ Health care setting (hospital, clinic, urgent care, etc.)<br>◯ Dense residential setting (nursing home, other long-term care facility)<br>◯ Prison or Jail<br>◯ Meatpacking facility<br>◯ Shipping or distribution facility<br>◯ High-volume retail facility (grocery store, etc)
During the last two weeks, has anyone in your household (not including you) experienced any of the following symptoms ? (select all that apply)	☐ Fever (measured by thermometer/self-diagnosed)<br>☐ Cough (new or worsening)<br>☐ Shortness of breath (new or worsening)<br>☐ Fatigue (new tiredness doing normal activities)<br>☐ Body aches<br>☐ Headache<br>☐ Diarrhea<br>☐ Sore throat<br>☐ Itchy, pink, orpainful eyes<br>☐ Runny nose or congestion<br>☐ Changes in sense of smell or taste<br>☐ New rash<br>☐ Repeated shaking with chills<br>☐ No one else had any of these symptoms
How many individuals in your household (not including you) experienced one or more of these symptoms?	__________________________
How old is/are the individual(s) who experienced symptoms (not including you)? (select all that apply)	☐ 0 to 4 years old<br>☐ 5 to 9 years old<br>☐ 10 to 17 years old<br>☐ 18 to 24 years old<br>☐ 25 to 49 years old<br>☐ 50 to 64 years old<br>☐ 65 to 74 years old<br>☐ 75 years or older

When did the earliest symptoms begin? ________________________________

Because of these symptoms, were you worried that they may have COVID-19?
- ◯ Yes
- ◯ No
- ◯ Don't know

Which of the following did the individual(s) with symptoms do to protect others after the symptoms began? (select all that apply)
- ☐ Wore a mask more frequentyly
- ☐ Washed hands with soap and water more frequently
- ☐ Used hand sanitizer more frequently
- ☐ Isolated yourself in your home more frequently
- ☐ Stayed home more frequently
- ☐ Wore disposable gloves more frequently

Which of the following did the individual(s) with symptoms do in response to the symptoms reported above? (select all that apply)
- ☐ nothing
- ☐ took over the counter medication (ibuprofen, acetimenophen, etc.)
- ☐ communicated with a health care provider over the phone
- ☐ visited a health care provider's office
- ☐ visited a retail clinic or pharmacy
- ☐ visited urgent care (FASTMed, etc.)
- ☐ visited the emergency room
- ☐ was admitted to the hospital
- ☐ other

If you indicated other, please explain ________________________________

How many days was the individual(s) with symptoms admitted in the hospital? ________________________________

Did the individual(s) with symptoms require any of the following interventions while admitted in the hospital? (select all that apply)
- ◯ Extra oxygen in your nose
- ◯ Treatment in the intensive care unit (ICU)
- ◯ Mechanical ventilation (intubation or a breathing tube)

Was the individual(s) with symptoms ever told that they may have been infected with COVID-19?
- ◯ Yes
- ◯ No
- ◯ Don't know

Was the individual(s) with symptoms ever tested for COVID-19?
- ◯ Yes
- ◯ No
- ◯ Don't know

Was one or more of the individual's tests positive for COVID-19?
- ◯ Yes
- ◯ No
- ◯ Don't know

What was the date of the individual's most recent positive COVID-19 test? ________________________________

What was the date of the individual's most recent COVID-19 test? ________________________________

What was the result of the individual's most recent COVID-19 test?	◯ Positive<br>◯ Negative<br>◯ Inconclusive<br>◯ Still waiting on result
Have the individual(s) with symptoms returned to their normal health at this time?	◯ Yes<br>◯ No<br>◯ Don't know
During the last two weeks, has anyone in your household (not including you) been tested for COVID for any other reason?	◯ Yes<br>◯ No<br>◯ Don't know
What was the reason for the individual being tested for COVID-19?	◯ Had symptoms of COVID-19<br>◯ Close contact of person who had COVID-19<br>◯ Employer provided testing<br>◯ Other
What was the reason for the individual being tested for COVID-19?	______________________________
Was one or more of the individual's tests positive for COVID-19?	◯ Yes<br>◯ No<br>◯ Don't know
What was the date of the individual's most recent positive COVID-19 test?	______________________________
What was the date of the individual's most recent COVID-19 test?	______________________________
What was the result of the individual's most recent COVID-19 test?	◯ Positive<br>◯ Negative<br>◯ Inconclusive<br>◯ Still waiting on result

Please click the 'Submit' button to finish the survey

Cabarrus County COVID-19 Prevalence and Immunity Study

Baseline Survey

MURDOCK Cabarrus County COVID-19 Prevalence and Immunity (C3PI) Study

Is the participant consented?

◯ Yes
◯ No

Thank you for enrolling in the MURDOCK Cabarrus County COVID-19 Prevalence and Immunity (C3PI) Study!

This survey should take you about 30 minutes to complete.

If you do not complete in one sitting, there is an option to save your responses and return later to complete.

Page 1 of 9

Personal & Household Information

Please review and confirm the following information from your MURDOCK Study record.

First name: ______________________

Last name: ______________________

Date of birth: ______________________

Age: ______________________

Sex:

◯ Female
◯ Male

Race:

Select all that apply.

☐ White/Caucasian
☐ Black or African American
☐ American Indian or Alaska Native
☐ Asian
☐ Native Hawaiian or other Pacific Islander
☐ Other race
☐ Don't know/not sure/prefer not to answer
(Select all that apply.)

Ethnicity:

◯ Hispanic or Latino
◯ Not Hispanic or Latino
◯ Don't Know or Unknown

Please confirm or update your preferred email address for future surveys: ______________________

Current residential address

Street address: ______________________

City: __________________________________

State:
- ◯ AL
- ◯ AK
- ◯ AZ
- ◯ AR
- ◯ CA
- ◯ CO
- ◯ CT
- ◯ DC
- ◯ DE
- ◯ FL
- ◯ GA
- ◯ HI
- ◯ ID
- ◯ IL
- ◯ IN
- ◯ IA
- ◯ KS
- ◯ KY
- ◯ LA
- ◯ ME
- ◯ MD
- ◯ MA
- ◯ MI
- ◯ MN
- ◯ MS
- ◯ MO
- ◯ MT
- ◯ NE
- ◯ NV
- ◯ NH
- ◯ NJ
- ◯ NM
- ◯ NY
- ◯ NC
- ◯ ND
- ◯ OH
- ◯ OK
- ◯ OR
- ◯ PA
- ◯ RI
- ◯ SC
- ◯ SD
- ◯ TN
- ◯ TX
- ◯ UT
- ◯ VT
- ◯ VA
- ◯ WA
- ◯ WV
- ◯ WI
- ◯ WY

Zip code: __________________________________

Please answer the following questions about your household. For this study, household is defined as a residence, regarded as a unit.

How long have you lived at your current residential address?	◯ 0-3 years<br>◯ 4-6 years<br>◯ 7-10 years<br>◯ 10+ years
How many total people (including yourself) live in this household?	◯ 1<br>◯ 2<br>◯ 3<br>◯ 4<br>◯ 5<br>◯ 6<br>◯ 7<br>◯ 8<br>◯ 9<br>◯ 10<br>◯ 11<br>◯ 12<br>◯ 13+
How many of the people in your household are below the age of 18?	◯ 0<br>◯ 1<br>◯ 2<br>◯ 3<br>◯ 4<br>◯ 5<br>◯ 6<br>◯ 7<br>◯ 8<br>◯ 9<br>◯ 10<br>◯ 11
If the COVID-19 pandemic caused your household to adjust to changes in child care, or having to home school, how difficult has that been?	◯ Not difficult<br>◯ Somewhat difficult<br>◯ Very difficult<br>◯ Extremely difficult<br>◯ Not applicable, no changes
What is the primary language spoken in your household?	◯ English<br>◯ Spanish<br>◯ Other, please specify
Other language, please specify:	__________________________________
How many dogs live in this household?	__________________________________<br>(Use whole numbers (e.g. "4"), Enter 0 if you have no dogs.)
How many cats live in this household?	__________________________________<br>(Use whole numbers (e.g. "4"), Enter 0 if you have no cats.)

Question	Response
What was your approximate total household income last year from all sources, before taxes?	◯ Less than $10,000<br>◯ $10,000-$19,999<br>◯ $20,000-$29,999<br>◯ $30,000-$49,999<br>◯ $50,000-$74,999<br>◯ $75,000-$89,999<br>◯ $90,000 or more
Before the COVID-19 pandemic, did you have regular caregiving responsibilities for someone who didn't live in your household (e.g. elderly parent or sibling who you regularly visited and supported, etc.)?	◯ Yes<br>◯ No
If yes, how difficult has it been to continue providing this care due to the COVID-19 pandemic?	◯ Not difficult<br>◯ Somewhat difficult<br>◯ Very difficult<br>◯ Extremely difficult
Please answer the following questions about yourself.	
What kind of phone do you have?	◯ iOS (Apple iPhone)<br>◯ Android (Google, Samsung)<br>◯ Do not have a smart phone<br>◯ Other, please specify
Other, please specify:	______________________
Do you own any of the following wearable devices? Please select all that apply.	☐ Fitbit<br>☐ Apple Watch<br>☐ Garmin<br>☐ Samsung<br>☐ Other, please specify<br>☐ I do not own any wearable device<br>(Please select all that apply.)
Other, please specify:	______________________
What is your height?	
Feet:	______________________<br>(Use whole numbers (e.g. "4"))
Inches:	______________________<br>(Use whole numbers (e.g. "4"))
What is your weight? (Please enter your response in pounds)	______________________<br>(Use whole numbers (e.g. "4"))

What is the highest level of education or schooling you have completed?	◯ Never attended school<br>◯ Kindergarten - 8th grade<br>◯ Some high school<br>◯ High school equivalency (GED)<br>◯ High school graduate<br>◯ Some college<br>◯ College graduate<br>◯ Graduate school, or higher degree, graduate
Are you covered by any type of medical or health insurance (including private insurance, insurance you purchased, Medicare, Medicaid, or any other health insurance program)?	◯ Yes<br>◯ No<br>◯ Don't know
What is the primary health insurance coverage that you have?	◯ Private health insurance through a job or school<br>◯ Insurance purchased through a state or federal health insurance exchange such as healthcare.gov<br>◯ Insurance purchased directly through a health plan or insurance company<br>◯ Medicare<br>◯ Medi-Gap<br>◯ Medicaid<br>◯ Military health care (TRICARE/VA/CHAMP-VA)<br>◯ Indian Health Service<br>◯ Other, please specify
Other, please specify:	__________________________________
Have you ever served on active duty in the U.S. Armed Forces? Active duty includes serving in the U.S. Armed Forces as well as activation from the Reserves or National Guard.	◯ Yes, on active duty in the past, but not now<br>◯ Yes, now on active duty<br>◯ No, never on active duty except for initial/basic training<br>◯ No, never served in the U.S. Armed Forces
In which branch of the service did/do you serve? (Mark any that apply)	☐ Army<br>☐ Navy<br>☐ Air Force<br>☐ Marine Corps<br>☐ Coast Guard<br>☐ National Guard<br>☐ Merchant Marines<br>☐ National Oceanic and Atmospheric Administration (NOAA)<br>☐ Public Health Service<br>☐ None<br>(Mark any that apply.)
What time period(s) did you serve? (Mark any that apply)	☐ September 2001 or later<br>☐ August 1990 to August 2001<br>☐ May 1975 to July 1990<br>☐ August 1964 to April 1975 (Vietnam era)<br>☐ February 1955 to July 1964<br>☐ July 1950 to January 1955 (Korean War)<br>☐ January 1947 to June 1950<br>☐ December 1941 to December 1946 (WWII)<br>☐ November 1941 or earlier

Have you ever been enrolled in VA health care?	◯ Yes<br>◯ No<br>◯ Don't know
Have you ever used any VA health care benefits?	◯ Yes<br>◯ No<br>◯ Don't know
Do you currently use the VA for your health care?	◯ Yes<br>◯ No<br>◯ Don't know
Which VA location(s)?<br><br>Please select all that apply.	☐ Salisbury<br>☐ Durham<br>☐ Fayetteville<br>☐ Asheville<br>☐ Other, please specify<br>(Please select all that apply.)
Other, please specify:	__________________________
Did you receive a flu vaccine this flu season (2019-2020)?	◯ Yes<br>◯ No<br>◯ Don't know
How often do you get a flu vaccine?	◯ Every flu season<br>◯ Most flu seasons<br>◯ Half of the flu seasons<br>◯ Less than half of the flu seasons<br>◯ Never

Have you ever been given a diagnosis of any of the following?

	Yes	No	Don't know
Allergies	◯	◯	◯
Asthma	◯	◯	◯
Diabetes	◯	◯	◯
Hypertension	◯	◯	◯
Cardiovascular disease such as heart attack, heart failure, angina, etc.	◯	◯	◯
Chronic respiratory disease such as chronic obstructive pulmonary disease (COPD), emphysema, chronic bronchitis, etc.	◯	◯	◯
Chronic kidney disease	◯	◯	◯
Chronic liver disease, such as cirrhosis, etc.	◯	◯	◯

Cancer	◯	◯	◯
Weakened immune system, such as HIV, chronic corticosteroid treatment, organ transplant recipient, or on another medication that weakens the immune system	◯	◯	◯
Other chronic condition, please specify	◯	◯	◯

Other chronic condition, please specify: __________________________________

You reported having chronic respiratory disease such as chronic obstructive pulmonary disease (COPD), emphysema, or chronic bronchitis. Do you currently use home oxygen (O2)?
◯ Yes
◯ No
◯ Don't know

How much oxygen do you use, on average?
◯ 1 L/min
◯ 2 L/min
◯ 3 L/min
◯ 4 L/min
◯ 5 L/min
◯ 6 L/min
◯ 7 L/min
◯ 8 L/min
◯ 9 L/min
◯ 10 L/min

You reported having Chronic Kidney Disease, are you currently on dialysis?
◯ Yes
◯ No
◯ Don't know

You reported having cancer, are you currently on chemotherapy?
◯ Yes
◯ No

Page 2 of 9

Lifestyle

Please answer the following questions about yourself, including exercise habits, smoking history and alcohol use.

In general, how would you have rated your health before the COVID-19 pandemic?
◯ Excellent
◯ Very good
◯ Good
◯ Fair
◯ Poor

At least once a week, do you engage in regular activity like brisk walking, jogging, bicycling, swimming, etc. long enough to work up a sweat, get your heart thumping, or get out of breath?
◯ Yes
◯ No

On average, how many days per week and minutes per day do you engage in this kind of exercise?

Enter number of days per week:

____
(Use whole numbers (e.g. "4"))

Enter number of minutes per day:

____
(Use whole numbers (e.g. "4"))

When you are exercising in your usual fashion, how would you rate your average level of exertion (degree of effort)?

- ◯ Easy
- ◯ Medium (can hold a conversation)
- ◯ Hard (but you can push yourself to continue)
- ◯ Very Hard (cannot hold a conversation)
- ◯ Extremely Hard (out of breath, your body wants to stop the exercise)

Do you currently smoke cigarettes, cigars, or a pipe on a daily basis?

- ◯ Yes
- ◯ No

What year did you begin smoking?

____

What is the average number of tobacco products smoked per day since you began using tobacco products?

Enter number of cigarettes per day:

____
(Use whole numbers (e.g. "4"))

Enter number of cigars you smoke per day:

____
(Use whole numbers (e.g. "4"))

Enter number of bowls of tobacco per day:

____
(Use whole numbers (e.g. "4"))

Have you ever smoked cigarettes, cigars, or a pipe on a daily basis?

- ◯ Yes
- ◯ No

What year did you begin smoking?

____

What year did you stop smoking?

____

What is the average number of tobacco products smoked per day when you were smoking?

Enter number of cigarettes per day:

____
(Use whole numbers (e.g. "4"))

Enter number of cigars you smoked per day:

____
(Use whole numbers (e.g. "4"))

Enter number of bowls of tobacco per day:

____
(Use whole numbers (e.g. "4"))

Do you currently use electronic cigarettes (e-cigarettes, vaping)?
- ◯ Yes
- ◯ No

What year did you begin using electronic cigarettes?

____

What is the average number of cartridges vaped per day since you began using electronic cigarettes?

____
(Use whole numbers (e.g. "4"))

Have you ever used electronic cigarettes (e-cigarettes, vaping)?
- ◯ Yes
- ◯ No

What year did you begin using electronic cigarettes?

____

What year did you stop using electronic cigarettes?

____

What was the average number of cartridges vaped per day when you were using electronic cigarettes?

____
(Use whole numbers (e.g. "4"))

Do you currently drink alcohol at least once a week?
- ◯ Yes
- ◯ No

What year did you begin drinking alcohol at least once a week?

____

On how many weekdays (Monday through Friday) do you usually drink alcohol?
- ◯ 0
- ◯ 1
- ◯ 2
- ◯ 3
- ◯ 4
- ◯ 5
- ◯ Only occasionally

When you drink on a weekday, how many drinks do you usually have? (One drink is equal to 5 ounces of wine, 12 ounces of beer, or 1.5 ounces of liquor)
- ◯ 1
- ◯ 2
- ◯ 3
- ◯ 4
- ◯ 5
- ◯ 6
- ◯ >6

On how many weekend days (Saturday and Sunday) do you usually drink alcohol?
- ◯ 0
- ◯ 1
- ◯ 2
- ◯ Only occasionally

When you drink on a weekend day, how many drinks do you usually have? (One drink is equal to 5 ounces of wine, 12 ounces of beer, or 1.5 ounces of liquor)	◯ 1<br>◯ 2<br>◯ 3<br>◯ 4<br>◯ 5<br>◯ 6<br>◯ >6
Did you ever drink alcohol at least once a week?	◯ Yes<br>◯ No
What year did you begin drinking alcohol at least once a week?	__________________________________
What year did you stop drinking alcohol at least once a week?	__________________________________
On how many weekdays (Monday through Friday) did you usually drink alcohol?	◯ 0<br>◯ 1<br>◯ 2<br>◯ 3<br>◯ 4<br>◯ 5<br>◯ Only occasionally
When you drank on a weekday, how many drinks did you usually have? (One drink is equal to 5 ounces of wine, 12 ounces of beer, or 1.5 ounces of liquor)	◯ 1<br>◯ 2<br>◯ 3<br>◯ 4<br>◯ 5<br>◯ 6<br>◯ >6
On how many weekend days (Saturday and Sunday) did you usually drink alcohol?	◯ 0<br>◯ 1<br>◯ 2<br>◯ Only occasionally
When you drank on a weekend day, how many drinks did you usually have? (One drink is equal to 5 ounces of wine, 12 ounces of beer, or 1.5 ounces of liquor)	◯ 1<br>◯ 2<br>◯ 3<br>◯ 4<br>◯ 5<br>◯ 6<br>◯ >6
Page 3 of 9	
Employment	

Before the COVID-19 pandemic began in North Carolina, which of the following best described your work situation? Check all that apply.	☐ Worked full time<br>☐ Worked part time<br>☐ Unemployed<br>☐ Furloughed or temporarily laid off<br>☐ Was looking for work/employment<br>☐ Retired<br>☐ Homemaker<br>☐ Student<br>☐ On maternity/paternity leave<br>☐ On illness/sick leave<br>☐ On disability<br>☐ Other, please specify<br>(Please check all that apply.)
Other, please specify:	______________________________
Of the job (or jobs) that you held before the COVID-19 pandemic in North Carolina, which description(s) best described your work?<br><br>Select all that apply.	☐ Healthcare professional (e.g. nurse, physician, advanced practice provider)<br>☐ Other professional (e.g. lawyer, pharmacist, executive)<br>☐ Essential service worker (e.g. postal/package delivery, grocery)<br>☐ Other service worker (e.g. waitstaff, hair stylist, home cleaning)<br>☐ First responder (e.g. fire fighter, police, EMT/paramedic)<br>☐ Managerial<br>☐ Administrative support<br>☐ Educator<br>☐ Farming/forestry/fishing/landscape<br>☐ Precision production/craft/repair<br>☐ Operators/fabricators/laborers<br>☐ Military<br>☐ Other, please specify<br>(Select all that apply.)
Other, please specify:	______________________________
Before the COVID-19 pandemic began in North Carolina, did you consider yourself self-employed?	◯ Yes<br>◯ No<br>◯ Don't know
Did you consider yourself a "gig-economy" worker (e.g. like an Uber driver or Task Rabbit handyman)?	◯ Yes<br>◯ No<br>◯ Don't know
How many years had you spent at your main job prior to the COVID-19 pandemic?	______________________________<br>(Use whole numbers (e.g. "4"))
In your main job before the COVID-19 pandemic, how often were you required to work from outside of the home?	◯ Always<br>◯ Often<br>◯ Sometimes<br>◯ Hardly ever<br>◯ Never

In your main job before the COVID-19 pandemic, did your employer offer you any of the following benefits? (select all that apply)	☐ Paid sick leave<br>☐ Paid vacation/personal leave<br>☐ Health insurance<br>☐ Disability insurance<br>☐ Retirement plan<br>(Select all that apply.)

Page 4 of 9

Impact of the COVID-19 Pandemic

Please answer the following questions about your household. For this study, household is defined as a residence, and its occupants, regarded as a unit.

Has the composition of your household changed since the COVID-19 crisis began in North Carolina? (e.g., a parent moved in to quarantine with you, or your college-aged child moved back home unexpectedly)	◯ Yes<br>◯ No
Please explain how your household has changed since the COVID-19 crisis began in North Carolina?	______________________________
If someone in the household became sick with COVID-19, how well would that person be able to isolate and limit contact with the rest of the household (e.g., separate bedroom, separate bathroom, etc)?	◯ Extremely well<br>◯ Very well<br>◯ Pretty well<br>◯ Not very well
Do any members of your household (other than you) have a job that requires/has required them to leave the home for their jobs?	◯ Yes<br>◯ No<br>◯ Don't know<br>◯ Not applicable
Thinking about your household as a whole, aside from getting groceries, how often has everyone been staying at home and avoiding interacting with others outside your household?	◯ Always<br>◯ Most of the time<br>◯ Half of the time<br>◯ Less than half of the time<br>◯ Never

How often have you done the following things since the beginning of the COVID-19 pandemic in North Carolina to protect yourself from infection and to keep from spreading infection to others, in addition to what you normally did before COVID-19?

	Always	Most of the time	Half of the time	Less than half of the time	Never
Worn a face mask when in public places or at work	◯	◯	◯	◯	◯
Washed hands and/or used sanitizer frequently	◯	◯	◯	◯	◯
Stayed at least 6 feet away from others	◯	◯	◯	◯	◯

Avoided large gatherings, public spaces, or crowds	○	○	○	○	○
Avoided contact with people who could be high risk	○	○	○	○	○
Avoided food from restaurants, including takeout	○	○	○	○	○
Worked or studied at home instead of going into an office/classroom	○	○	○	○	○
Avoided shaking hands or touching people	○	○	○	○	○
Stayed home when I am sick	○	○	○	○	○
Wiped down surfaces with disinfectant	○	○	○	○	○

Since the beginning of the COVID-19 pandemic in North Carolina, have you done any of the following to protect yourself from infection and to keep from spreading infection to others? Select all that apply.

☐ Cancelled or postponed planned travel for work
☐ Cancelled or postponed travel for pleasure
☐ Cancelled or postponed personal or social activities
☐ Cancelled a doctor's appointment
☐ Stockpiled food or water
☐ Followed government guidelines or rules to shelter in place. Specifically, staying at home and limiting contacts with other people
(Please select all that apply.)

How has the COVID-19 pandemic changed how you are saving money?

○ I am saving a lot more
○ I am saving a little more
○ I am saving the same
○ I am saving a little less
○ I am saving a lot less

How has the COVID-19 pandemic changed how you are spending money?

○ I am spending a lot more
○ I am spending a little more
○ I am spending the same
○ I am spending a little less
○ I am spending a lot less

How has the COVID-19 pandemic changed how you are borrowing money?

○ I am borrowing a lot more
○ I am borrowing a little more
○ I am borrowing the same
○ I am borrowing a little less
○ I am borrowing a lot less
○ Not applicable

How has the COVID-19 pandemic changed your reliance on public assistance or charity?	◯ My use of public assistance or charity has increased a lot<br>◯ My use of public assistance or charity has increased a little<br>◯ My use of public assistance or charity has stayed the same<br>◯ My use of public assistance or charity has decreased a little<br>◯ My use of public assistance or charity has decreased a lot<br>◯ Not applicable

Page 5 of 9

Outlook on the COVID-19 Pandemic

	0	1	2	3	4	5	6	7	8	9	10
On a scale of 0 (not at all informed) to 10 (very well informed), how well informed are you about the COVID-19 pandemic?	◯	◯	◯	◯	◯	◯	◯	◯	◯	◯	◯

Do you plan to get a vaccine for COVID-19 when one becomes available?	◯ Yes<br>◯ No<br>◯ Don't know
How do you think your total household income will change this year due to the COVID-19 crisis?	◯ Decrease significantly<br>◯ Decrease slightly<br>◯ Stay the same<br>◯ Increase slightly<br>◯ Increase significantly

	0	1	2	3	4	5	6	7	8	9	10
On a scale of 0 (definitely not going to happen) to 10 (definitely going to happen), how likely do you think it is that your household will run out of money in the next 3 months?	◯	◯	◯	◯	◯	◯	◯	◯	◯	◯	◯

	0	1	2	3	4	5	6	7	8	9	10
On a scale of 0 (definitely not going to happen) to 10 (definitely going to happen), how likely is it that you could get financial support from friends or family outside of your house if you ran out of money?	◯	◯	◯	◯	◯	◯	◯	◯	◯	◯	◯

Suppose that you have a surprise medical bill of $400. Based on your current household financial situation, how will you pay for this expense? (select all that apply)	☐ Put it on my credit card and pay it in full at the next statement<br>☐ Put it on my credit card and pay it off over time<br>☐ Pay with money currently in savings/checking<br>☐ Pay with money from a bank loan<br>☐ Pay with money borrowed from family or friends<br>☐ Pay using a payday loan/deposit advance/overdraft<br>☐ Pay by selling something else<br>☐ I wouldn't be able to pay<br>☐ Other, please specify<br>(Select all that apply.)
Other, please specify:	__________________________
How often is your family getting help with running necessary errands, such as getting groceries or medications?	○ Always<br>○ Most of the time<br>○ Half of the time<br>○ Less than half of the time<br>○ Never
Page 6 of 9	
COVID-19 Symptom Monitoring	
During the last two weeks, have you experienced any of the following symptoms?	
Fever:	○ Yes<br>○ No
Date of symptom onset:	__________________________
Cough:	○ Yes<br>○ No
Date of symptom onset:	__________________________
Shortness of breath:	○ Yes<br>○ No
Date of symptom onset:	__________________________
Fatigue:	○ Yes<br>○ No
Date of symptom onset:	__________________________
Body aches:	○ Yes<br>○ No
Date of symptom onset:	__________________________

Headache:	◯ Yes<br>◯ No
Date of symptom onset:	______________________________
Diarrhea:	◯ Yes<br>◯ No
Date of symptom onset:	______________________________
Sore throat:	◯ Yes<br>◯ No
Date of symptom onset:	______________________________
Itchy pink or painful eyes:	◯ Yes<br>◯ No
Date of symptom onset:	______________________________
Runny nose or congestion:	◯ Yes<br>◯ No
Date of symptom onset:	______________________________
Changes in your sense of smell or taste	◯ Yes<br>◯ No
Date of symptom onset:	______________________________
New rash:	◯ Yes<br>◯ No
Date of symptom onset:	______________________________
Have you returned to your normal health at this time?	◯ Yes<br>◯ No<br>◯ Don't know
Did you experience any bias or discrimination due to your symptoms?	◯ Yes<br>◯ No<br>◯ Don't know
Please describe the bias or discrimination you experienced:	_____________________________________

When you experienced symptoms, were you worried that you may have COVID-19?	◯ Yes<br>◯ No<br>◯ Don't know
When you experienced symptoms, did anyone tell you that you may have been infected with COVID-19?	◯ Yes<br>◯ No<br>◯ Don't know
Did you seek out testing for COVID-19?	◯ Yes<br>◯ No<br>◯ Don't know
Were you tested for COVID-19?	◯ Yes<br>◯ No<br>◯ Don't know
What was the date of your test?	______________________
What was the result of your test?	◯ Pending<br>◯ Inconclusive<br>◯ Positive<br>◯ Negative

When you experienced symptoms, how often did you do the following things to limit the risk of potentially spreading your illness?

	Always	Most of the time	Half of the time	Less than half of the time	Never
Wore a face mask when in public or at work	◯	◯	◯	◯	◯
Washed hands and/or used sanitizer frequently	◯	◯	◯	◯	◯
Stayed at least 6 feet away from others	◯	◯	◯	◯	◯
Avoided large gatherings, public spaces, or crowds	◯	◯	◯	◯	◯
Avoided contact with people who could be high risk	◯	◯	◯	◯	◯
Avoided food from restaurants, including takeout	◯	◯	◯	◯	◯
Worked or studied at home instead of going into an office/classroom	◯	◯	◯	◯	◯
Avoided shaking hands or touching people	◯	◯	◯	◯	◯
Stayed home when I was sick	◯	◯	◯	◯	◯

Wiped down surfaces with disinfectant	○	○	○	○	○

When you experienced symptoms, did you do any of the following things to limit the risk of potentially spreading your illness? Select all that apply.	☐ Cancelled or postponed planned travel for work<br>☐ Cancelled or postponed planned travel for pleasure<br>☐ Cancelled or postponed personal or social activities<br>☐ Cancelled a doctor's appointment<br>☐ Stockpiled food or water<br>☐ Followed government guidelines or rules to shelter in place. Specifically, staying at home and limiting contacts with other people<br>(Please select all that apply.)
When you experienced symptoms, what did you do to take care of these symptoms?	
Took over-the-counter (OTC) medication	○ Yes<br>○ No
Which over the counter medications did you take? (Check all that apply)	☐ NSAIDs or NSAID-containing medications<br>☐ Acetaminophen-containing medications<br>☐ Other, please specify<br>(Check all that apply.)
Other, please specify:	______________________
Date you first took any OTC medication:	______________________
Communicated with a health care provider over the phone	○ Yes<br>○ No
Date:	______________________
Visited a health care provider's office	○ Yes<br>○ No
Date:	______________________
Visited a retail clinic or pharmacy	○ Yes<br>○ No
Date:	______________________
Visited urgent care (FASTMed, etc.)	○ Yes<br>○ No
Date:	______________________
Visited the emergency room	○ Yes<br>○ No

Date:	________________
Went to a COVID-19 testing location	◯ Yes<br>◯ No
Date:	________________
Other	◯ Yes<br>◯ No
Other, please specify	________________
Date:	________________
When you experienced symptoms, were you admitted to the hospital?	◯ Yes<br>◯ No
When were you admitted to the hospital?	________________
For how many days were you in the hospital?	________________<br>(Use whole numbers (e.g. "4"))
During your hospital stay, did you require the following interventions?	
Extra oxygen in your nose	◯ Yes<br>◯ No
How many days did you use extra oxygen in your nose?	________________<br>(Use whole numbers (e.g. "4"))
Treatment in the intensive care unit (ICU)	◯ Yes<br>◯ No
How many days were you treated in an ICU?	________________<br>(Use whole numbers (e.g. "4"))
Mechanical ventilation (intubation or a breathing tube)	◯ Yes<br>◯ No
How many days did you need mechanical ventilation?	________________<br>(Use whole numbers (e.g. "4"))
If other interventions were required, please briefly specify:	________________

General Health & Wellbeing

In general, how would you rate your health over the last two weeks?

- ◯ Excellent
- ◯ Very good
- ◯ Good
- ◯ Fair
- ◯ Poor

Are you currently pregnant?

- ◯ Yes
- ◯ No

Please indicate how significant a source of stress the COVID-19 pandemic is in your life right now.

- ◯ Very significant
- ◯ Somewhat significant
- ◯ Not very significant
- ◯ Not at all significant

How serious a problem would you say the COVID-19 pandemic is right now, for you and/or others:

	Very serious	Somewhat serious	Not too serious	Not at all serious	Don't know
For you personally	◯	◯	◯	◯	◯
For people in your community	◯	◯	◯	◯	◯
For people in the United States	◯	◯	◯	◯	◯
For people around the world	◯	◯	◯	◯	◯

The COVID-19 pandemic causes challenges for some people regardless of whether they are infected. How concerned are you about each of the following things?

	Very concerned	Somewhat concerned	Not at all concerned	Don't know	Not applicable
Getting the healthcare I need (including care for mental health)	◯	◯	◯	◯	◯
Having a place to live	◯	◯	◯	◯	◯
Being able to interact with other people	◯	◯	◯	◯	◯
Getting food, water, and other household supplies	◯	◯	◯	◯	◯
Getting medication	◯	◯	◯	◯	◯
Having transportation to get where I need to go	◯	◯	◯	◯	◯
Caring for my family and friends	◯	◯	◯	◯	◯
Losing my job	◯	◯	◯	◯	◯
Finding a job	◯	◯	◯	◯	◯
Feeding my family	◯	◯	◯	◯	◯
My children's education	◯	◯	◯	◯	◯

Page 8 of 9

Employment & Risk

Question	Response
Has your work situation changed since the COVID-19 pandemic began in North Carolina?	◯ Yes<br>◯ No<br>◯ Don't know
Which of the following best fits your current work situation? Check all that apply.	☐ Working full time<br>☐ Working part time<br>☐ Unemployed<br>☐ Furloughed or temporarily laid off<br>☐ Looking for work/employment<br>☐ Retired<br>☐ Homemaker<br>☐ Student<br>☐ On maternity/paternity leave<br>☐ On illness/sick leave<br>☐ On disability<br>☐ Other, please specify<br>(Please check all that apply.)
Other, please specify:	__________________________________
Of the job (or jobs) that you currently hold, which description(s) best describes your work? Select all that apply.	☐ Healthcare professional (e.g. nurse, physician, advanced practice provider)<br>☐ Other professional (e.g. lawyer, pharmacist, executive)<br>☐ Essential service worker (e.g. postal/package delivery, grocery)<br>☐ Other service worker (e.g. waitstaff, hair stylist, home cleaning)<br>☐ First responder (e.g. fire fighter, police, EMT/paramedic)<br>☐ Managerial<br>☐ Administrative support<br>☐ Educator<br>☐ Farming/forestry/fishing/landscape<br>☐ Precision production/craft/repair<br>☐ Operators/fabricators/laborers<br>☐ Military<br>☐ Other, please specify<br>(Please select all that apply.)
Other, please specify:	__________________________________
Do you currently consider yourself self-employed?	◯ Yes<br>◯ No<br>◯ Don't know
Do you consider yourself a "gig-economy worker" (e.g. like an Uber driver or Task Rabbit handyman)?	◯ Yes<br>◯ No<br>◯ Don't know
Does your primary employer currently offer you any of the following benefits? (select all that apply)	☐ Paid sick leave<br>☐ Paid vacation/personal leave<br>☐ Health insurance<br>☐ Disability insurance<br>☐ Retirement plan<br>(Select all that apply.)

	0	1	2	3	4	5	6	7	8	9	10
On a scale of 0 (definitely not going to happen) to 10 (definitely going to happen), how likely is it that you will lose your job because of the COVID-19 pandemic?	◯	◯	◯	◯	◯	◯	◯	◯	◯	◯	◯

What do you think is mostly likely to happen to your work hours due to the COVID-19 pandemic?

◯ Decrease substantially
◯ Decrease a little
◯ No change
◯ Increase a little
◯ Increase substantially

How often are you required to work from outside of the home currently?

◯ Always
◯ Often
◯ Sometimes
◯ Hardly ever
◯ Never

Please indicate how regularly or often the following apply to you when you do work outside of the home?

	Always	Very regularly	Somewhat regularly	Not very regularly	Never	Not applicable
I am in close physical contact with co-workers	◯	◯	◯	◯	◯	◯
I am in close physical contact with clients	◯	◯	◯	◯	◯	◯
I have access to disposable gloves	◯	◯	◯	◯	◯	◯
I use disposable gloves	◯	◯	◯	◯	◯	◯
I have access to a face mask	◯	◯	◯	◯	◯	◯
I use a face mask	◯	◯	◯	◯	◯	◯
I wash my hands with soap and water	◯	◯	◯	◯	◯	◯
I sanitize my hands with hand sanitizer	◯	◯	◯	◯	◯	◯

How worried are you that you will be exposed to COVID-19 at your place of work outside of the home currently?

◯ Extremely worried
◯ Moderately worried
◯ Slightly worried
◯ Not at all worried

How worried are you that you will bring COVID-19 home from your work place to others in your household?

◯ Extremely worried
◯ Moderately worried
◯ Slightly worried
◯ Not at all worried

During the last two weeks, how often have you experienced the following due to your work outside the home during the COVID-19 pandemic?

	Nearly every day	More than half the days	Several days	Not at all
I believed that my job was putting me at great risk	◯	◯	◯	◯
I felt extra stress at work	◯	◯	◯	◯
I was afraid of falling ill with COVID-19	◯	◯	◯	◯
I felt I had little control over whether I would get infected or not	◯	◯	◯	◯
I thought I would be unlikely to survive if I were to get COVID-19	◯	◯	◯	◯
I thought about resigning because of COVID-19	◯	◯	◯	◯
I was afraid I would pass COVID-19 on to others	◯	◯	◯	◯
My family and friends were worried that they might get infected through me	◯	◯	◯	◯
People avoided my family because of my work	◯	◯	◯	◯
I was willing to accept the risks involved because I wanted to help the COVID-19 patients or the public	◯	◯	◯	◯

Page 9 of 9

Survey Preferences

Would you like to receive future surveys, and possible other study-related communication, via text message?

Please note that message and data rates may apply.

◯ Yes
◯ No
◯ Don't know

Please provide preferred mobile number to receive text messages: ______________________________

Cabarrus County COVID-19 Prevalence and Immunity Study

Biweekly Survey

MURDOCK C3PI Study Follow Up Survey

Page 1 of 3

COVID-19 Symptom Monitoring

* You reported being tested for COVID-19 on [previous-event-name][fu_covid_test_date] on your last survey, and that the result was pending. What was the final result of your test?	◯ Inconclusive<br>◯ Positive<br>◯ Negative
* You reported being tested for COVID-19 on [previous-event-name][fu_covid_test_any_date] on your last survey, and that the result was pending. What was the final result of your test?	◯ Inconclusive<br>◯ Positive<br>◯ Negative

During the last two weeks, have you experienced any of the following symptoms?

Fever (measured by thermometer/self-diagnosed):	◯ Yes<br>◯ No
Date of symptom onset:	______________________
Cough (new or worsening):	◯ Yes<br>◯ No
Date of symptom onset:	______________________
Shortness of breath (new or worsening):	◯ Yes<br>◯ No
Date of symptom onset:	______________________
Fatigue (new tiredness doing normal activities):	◯ Yes<br>◯ No
Date of symptom onset:	______________________
Body aches:	◯ Yes<br>◯ No
Date of symptom onset:	______________________
Headache:	◯ Yes<br>◯ No
Date of symptom onset:	______________________

Diarrhea:	◯ Yes<br>◯ No
Date of symptom onset:	__________________________
Sore throat:	◯ Yes<br>◯ No
Date of symptom onset:	__________________________
Itchy pink or painful eyes:	◯ Yes<br>◯ No
Date of symptom onset:	__________________________
Runny nose or congestion:	◯ Yes<br>◯ No
Date of symptom onset:	__________________________
Changes in your sense of smell or taste	◯ Yes<br>◯ No
Date of symptom onset:	__________________________
New rash:	◯ Yes<br>◯ No
Date of symptom onset:	__________________________
Have you returned to your normal health at this time?	◯ Yes<br>◯ No<br>◯ Don't know
Did you experience any bias or discrimination due to your symptoms?	◯ Yes<br>◯ No<br>◯ Don't know
Please describe the bias or discrimination you experienced:	________________________________
When you experienced symptoms, were you worried that you may have COVID-19?	◯ Yes<br>◯ No<br>◯ Don't know
When you experienced symptoms, did anyone tell you that you may have been infected with COVID-19?	◯ Yes<br>◯ No<br>◯ Don't know

Did you seek out testing for COVID-19?	◯ Yes<br>◯ No<br>◯ Don't know
Were you tested for COVID-19?<br><br>Do not count tests required if you are part of the C3PI testing group.	◯ Yes<br>◯ No<br>◯ Don't know
What was the date of your test?	________________________
What was the result of your test?	◯ Pending<br>◯ Inconclusive<br>◯ Positive<br>◯ Negative

When you experienced symptoms, how often did you do the following things to limit the risk of potentially spreading your illness?

	Always	Most of the time	Half of the time	Less than half of the time	Never
Wore a face mask when in public or at work	◯	◯	◯	◯	◯
Washed hands and/or used sanitizer frequently	◯	◯	◯	◯	◯
Stayed at least 6 feet away from others	◯	◯	◯	◯	◯
Avoided large gatherings, public spaces, or crowds	◯	◯	◯	◯	◯
Avoided contact with people who could be high risk	◯	◯	◯	◯	◯
Avoided food from restaurants, including takeout	◯	◯	◯	◯	◯
Worked or studied at home instead of going into an office/classroom	◯	◯	◯	◯	◯
Avoided shaking hands or touching people	◯	◯	◯	◯	◯
Stayed home when I was sick	◯	◯	◯	◯	◯
Wiped down surfaces with disinfectant	◯	◯	◯	◯	◯

When you experienced symptoms, did you do any of the following things to limit the risk of potentially spreading your illness? Select all that apply.	☐ Cancelled or postponed planned travel for work<br>☐ Cancelled or postponed planned travel for pleasure<br>☐ Cancelled or postponed personal or social activities<br>☐ Cancelled a doctor's appointment<br>☐ Stockpiled food or water<br>☐ Followed government guidelines or rules to shelter in place. Specifically, staying at home and limiting contacts with other people<br>(Please select all that apply.)
When you experienced symptoms, what did you do to take care of these symptoms?	
Took over-the-counter (OTC) medication	◯ Yes<br>◯ No
Which over the counter medications did you take? (Check all that apply)	☐ NSAIDs or NSAID-containing medications<br>☐ Acetaminophen-containing medications<br>☐ Other, please specify<br>(Check all that apply.)
Other, please specify:	______________________
Date you first took any OTC medication:	______________________
Communicated with a health care provider over the phone	◯ Yes<br>◯ No
Date:	______________________
Visited a health care provider's office	◯ Yes<br>◯ No
Date:	______________________
Visited a retail clinic or pharmacy	◯ Yes<br>◯ No
Date:	______________________
Visited urgent care (FASTMed, etc.)	◯ Yes<br>◯ No
Date:	______________________
Visited the emergency room	◯ Yes<br>◯ No
Date:	______________________

Went to a COVID-19 testing location	◯ Yes<br>◯ No
Date:	__________________
Other	◯ Yes<br>◯ No
Other, please specify	__________________
Date:	__________________
When you experienced symptoms, were you admitted to the hospital?	◯ Yes<br>◯ No
When were you admitted to the hospital?	__________________
For how many days were you in the hospital?	__________________<br>(Use whole numbers (e.g. "4"))
During your hospital stay, did you require the following interventions?	
Extra oxygen in your nose	◯ Yes<br>◯ No
How many days did you use extra oxygen in your nose?	__________________<br>(Use whole numbers (e.g. "4"))
Treatment in the intensive care unit (ICU)	◯ Yes<br>◯ No
How many days were you treated in an ICU?	__________________<br>(Use whole numbers (e.g. "4"))
Mechanical ventilation (intubation or a breathing tube)	◯ Yes<br>◯ No
How many days did you need mechanical ventilation?	__________________<br>(Use whole numbers (e.g. "4"))
If other interventions were required, please briefly specify:	______________________________
During the last two weeks, have you been tested for COVID-19 for any reason, other than home testing required if you are part of the C3PI testing group?	◯ Yes<br>◯ No

What was the date of your test?	__________________________________
What was the result of your test?	◯ Pending<br>◯ Inconclusive<br>◯ Positive<br>◯ Negative
What was the date of your result?	__________________________________
Page 2 of 3	
General Health & Wellbeing	
In general, how would you rate your health over the last two weeks?	◯ Excellent<br>◯ Very good<br>◯ Good<br>◯ Fair<br>◯ Poor
Are you currently pregnant?	◯ Yes<br>◯ No
At enrollment, you reported having one or more people under the age of 18 in your household. Do you still have one or more people under the age of 18 in your household?	◯ Yes<br>◯ No
How difficult has it been for your household with persons under the age of 18 to adjust to the changes caused by the COVID-19 pandemic?	◯ Not difficult<br>◯ Somewhat difficult<br>◯ Very difficult<br>◯ Extremely difficult
To what extent has the COVID-19 pandemic disrupted plans for you and your family for the Summer of 2020?	◯ Significant disruption<br>◯ Moderate disruption<br>◯ Little disruption<br>◯ No disruption
What is the format you would prefer for your child or children for the beginning of the school year 2020?	◯ In-person attendance as would be expected prior to the COVID-19 pandemic<br>◯ In-person attendance, but with masking and social distancing guidelines in place<br>◯ Some in-person attendance with some remote learning<br>◯ All remote learning, no in-person attendance at school
What is the format that seems mostly likely for your children for the beginning of the school year 2020?	◯ In-person attendance as would be expected prior to the COVID-19 pandemic<br>◯ In-person attendance, but with masking and social distancing guidelines in place<br>◯ Some in-person attendance with some remote learning<br>◯ All remote learning, no in-person attendance at school

	0	1	2	3	4	5	6	7	8	9	10
On a scale of 0 (not at all informed) to 10 (very well informed), how well informed are you about the COVID-19 pandemic?	○	○	○	○	○	○	○	○	○	○	○

Please indicate how significant a source of stress the COVID-19 pandemic is in your life right now.

- ○ Very significant
- ○ Somewhat significant
- ○ Not very significant
- ○ Not at all significant

How serious a problem would you say the COVID-19 pandemic is right now, for you and/or others:

	Very serious	Somewhat serious	Not too serious	Not at all serious	Don't know
For you personally	○	○	○	○	○
For people in your community	○	○	○	○	○
For people in the United States	○	○	○	○	○
For people around the world	○	○	○	○	○

The COVID-19 pandemic causes challenges for some people regardless of whether they are infected. How concerned are you about each of the following things?

	Very concerned	Somewhat concerned	Not at all concerned	Don't know	Not applicable
Getting the healthcare I need (including care for mental health)	○	○	○	○	○
Having a place to live	○	○	○	○	○
Being able to interact with other people	○	○	○	○	○
Getting food, water, and other household supplies	○	○	○	○	○
Getting medication	○	○	○	○	○
Having transportation to get where I need to go	○	○	○	○	○
Caring for my family and friends	○	○	○	○	○
Losing my job	○	○	○	○	○
Finding a job	○	○	○	○	○
Feeding my family	○	○	○	○	○
My children's education	○	○	○	○	○

Have you received a flu vaccine for this season (2020-2021)?

- ○ Yes
- ○ No

Do you intend to be vaccinated for the flu this season?

- ○ Yes
- ○ No
- ○ Don't know or not sure

Have you participated in a COVID-19 vaccine clinical trial?	◯ Yes<br>◯ No<br>◯ Don't know
Have you received a vaccination for COVID-19?	◯ Yes<br>◯ No<br>◯ Don't know
What vaccine did you receive?	◯ Pfizer-BioNTech COVID-19 vaccine<br>◯ Moderna's COVID-19 vaccine<br>◯ Johnson & Johnson's COVID-19 vaccine<br>◯ Don't know or not sure<br>(Please select "don't know" if unknown)
Date of first shot:	______________________

On your last C3PI follow-up you reported receiving a COVID-19 vaccine.

You have reported that your first shot date was [previous-event-name][fu_covid_vaccine_date1] and that you had not received your second shot.

Please use the field below to report your second shot date.

Date of second shot:	______________________<br>(______)
Do you plan to get a vaccine for COVID-19 when available?	◯ Yes<br>◯ No<br>◯ Don't know

Page 3 of 3

Employment & Risk

Are you currently working for pay?	◯ Yes<br>◯ No
Has your work situation changed in the last two weeks?	◯ Yes<br>◯ No<br>◯ Don't know
Which of the following best fits your current work situation? Check all that apply.	☐ Working full time<br>☐ Working part time<br>☐ Unemployed<br>☐ Furloughed or temporarily laid off<br>☐ Looking for work/employment<br>☐ Retired<br>☐ Homemaker<br>☐ Student<br>☐ On maternity/paternity leave<br>☐ On illness/sick leave<br>☐ On disability<br>☐ Other, please specify<br>(Please check all that apply.)

Other, please specify: __________________________________

Of the job (or jobs) that you currently hold, which description(s) best describes your work? Select all that apply.

- ☐ Healthcare professional (e.g. nurse, physician, advanced practice provider)
- ☐ Other professional (e.g. lawyer, pharmacist, executive)
- ☐ Essential service worker (e.g. postal/package delivery, grocery)
- ☐ Other service worker (e.g. waitstaff, hair stylist, home cleaning)
- ☐ First responder (e.g. fire fighter, police, EMT/paramedic)
- ☐ Managerial
- ☐ Administrative support
- ☐ Educator
- ☐ Farming/forestry/fishing/landscape
- ☐ Precision production/craft/repair
- ☐ Operators/fabricators/laborers
- ☐ Military
- ☐ Other, please specify

(Please select all that apply.)

Other, please specify: __________________________________

Do you currently consider yourself self-employed?

- ◯ Yes
- ◯ No
- ◯ Don't know

Do you consider yourself a "gig-economy worker" (e.g. like an Uber driver or Task Rabbit handyman)?

- ◯ Yes
- ◯ No
- ◯ Don't know

Does your primary employer currently offer you any of the following benefits? (select all that apply)

- ☐ Paid sick leave
- ☐ Paid vacation/personal leave
- ☐ Health insurance
- ☐ Disability insurance
- ☐ Retirement plan

(Select all that apply.)

	0	1	2	3	4	5	6	7	8	9	10
On a scale of 0 (definitely not going to happen) to 10 (definitely going to happen), how likely is it that you will lose your job because of the COVID-19 pandemic?	◯	◯	◯	◯	◯	◯	◯	◯	◯	◯	◯

What do you think is mostly likely to happen to your work hours due to the COVID-19 pandemic?

- ◯ Decrease substantially
- ◯ Decrease a little
- ◯ No change
- ◯ Increase a little
- ◯ Increase substantially

How often are you required to work from outside of the home currently?

- ◯ Always
- ◯ Often
- ◯ Sometimes
- ◯ Hardly ever
- ◯ Never

Please indicate how regularly or often the following apply to you when you do work outside of the home?

	Always	Very regularly	Somewhat regularly	Not very regularly	Never	Not applicable
I am in close physical contact with co-workers	◯	◯	◯	◯	◯	◯
I am in close physical contact with clients	◯	◯	◯	◯	◯	◯
I have access to disposable gloves	◯	◯	◯	◯	◯	◯
I use disposable gloves	◯	◯	◯	◯	◯	◯
I have access to a face mask	◯	◯	◯	◯	◯	◯
I use a face mask	◯	◯	◯	◯	◯	◯
I wash my hands with soap and water	◯	◯	◯	◯	◯	◯
I sanitize my hands with hand sanitizer	◯	◯	◯	◯	◯	◯

How worried are you that you will be exposed to COVID-19 at your place of work outside of the home currently?

- ◯ Extremely worried
- ◯ Moderately worried
- ◯ Slightly worried
- ◯ Not at all worried

How worried are you that you will bring COVID-19 home from your work place to others in your household?

- ◯ Extremely worried
- ◯ Moderately worried
- ◯ Slightly worried
- ◯ Not at all worried

During the last two weeks, how often have you experienced the following due to your work outside the home during the COVID-19 pandemic?

	Nearly every day	More than half the days	Several days	Not at all
I believed that my job was putting me at great risk	◯	◯	◯	◯
I felt extra stress at work	◯	◯	◯	◯
I was afraid of falling ill with COVID-19	◯	◯	◯	◯
I felt I had little control over whether I would get infected or not	◯	◯	◯	◯

I thought I would be unlikely to survive if I were to get COVID-19	◯	◯	◯	◯
I thought about resigning because of COVID-19	◯	◯	◯	◯
I was afraid I would pass COVID-19 on to others	◯	◯	◯	◯
My family and friends were worried that they might get infected through me	◯	◯	◯	◯
People avoided my family because of my work	◯	◯	◯	◯
I was willing to accept the risks involved because I wanted to help the COVID-19 patients or the public	◯	◯	◯	◯

Do any members of your household (other than you) have a job that requires/has required them to leave the home for their jobs?

◯ Yes
◯ No
◯ Don't know
◯ Not applicable

How worried are you that the member(s) of your household who work outside the home will bring COVID-19 home into your household?

◯ Extremely worried
◯ Moderately worried
◯ Slightly worried
◯ Not at all worried

Thinking about your household as a whole, aside from getting groceries, how often has everyone been staying at home and avoiding interacting with others outside your household?

◯ Always
◯ Most of the time
◯ Half of the time
◯ Less than half of the time
◯ Never

In the last two weeks, how often have you done the following things to protect yourself from infection and to keep from spreading infection to others?

	Always	Most of the time	Half of the time	Less than half of the time	Never
Worn a face mask when in public places or at work	◯	◯	◯	◯	◯
Washed hands and/or used sanitizer frequently	◯	◯	◯	◯	◯
Stayed at least 6 feet away from others	◯	◯	◯	◯	◯
Avoided large gatherings, public spaces, or crowds	◯	◯	◯	◯	◯
Avoided contact with people who could be high risk	◯	◯	◯	◯	◯

Avoided food from restaurants, including takeout	○	○	○	○	○
Worked or studied at home instead of going into an office/classroom	○	○	○	○	○
Avoided shaking hands or touching people	○	○	○	○	○
Stayed home when I am sick	○	○	○	○	○
Wiped down surfaces with disinfectant	○	○	○	○	○

In the last two weeks, have you done any of the following to protect yourself from infection and to keep from spreading infection to others? Select all that apply.

- ☐ Cancelled or postponed planned travel for work
- ☐ Cancelled or postponed travel for pleasure
- ☐ Cancelled or postponed personal or social activities
- ☐ Cancelled a doctor's appointment
- ☐ Stockpiled food or water
- ☐ Followed government guidelines or rules to shelter in place. Specifically, staying at home and limiting contacts with other people

(Please select all that apply.)
