# Supplementary material for: Incidence of severe acute respiratory syndrome coronavirus 2 (SARS-CoV-2) infection in North Carolina from December 2020 – February 2022
Source: PLoS One. 2025 Oct 8;20(10):e0332645. doi: 10.1371/journal.pone.0332645 (PMC12507194; doi:10.1371/journal.pone.0332645)
Supplement: S1 Table — (S1 Table.PDF) [file pone.0332645.s002.pdf]

Month of Study		Cabarrus County		Chatham County		Pitt County		Overall	
		n	% (95% CI)	n	% (95% CI)	n	% (95% CI)	n	% (95% CI)
2020	December	NR[a]		103	2.9 (1.7, 4.9)	162	9.9 (8.2, 11.9)	265	7.2 (6.0, 8.5)
2021	January	241	14.1 (12.5, 15.9)	108	11.1 (8.9, 13.9)	172	25.0 (22.6, 27.6)	521	17.1 (15.9, 18.3)
	February	NR[a]		107	32.7 (29.3, 36.3)	149	53.7 (50.6, 56.8)	256	44.9 (42.6, 47.2)
	March	237	57.4 (54.9, 59.7)	117	63.2 (59.7, 66.6)	138	79.0 (76.1, 81.5)	492	64.8 (63.3, 66.4)
	April	NA[b]		117	82.9 (79.9, 85.5)	143	95.1 (93.3, 96.4)	260	89.6 (88.0, 91.0)
	May	NA[b]		112	85.7 (82.8, 88.2)	NA[b]		112	85.7 (82.8, 88.2)
	June	272	94.1 (92.9, 95.1)	129	84.5 (81.8, 86.8)	NA[b]		378	96.6 (95.7, 97.2)
	July	270	94.8 (93.6, 95.8)	108	86.1 (83.2, 88.6)	NA[b]		365	95.6 (94.7, 96.4)
	August	264	94.7 (93.5, 95.7)	65	93.8 (90.5, 96.1)	NA[b]		329	94.5 (93.4, 95.4)
	September	264	95.5 (94.3, 96.4)	46	80.4 (74.9, 84.9)	NA[b]		310	93.2 (92.0, 94.3)
	October	259	96.1 (95.0, 97.0)	54	94.4 (90.7, 96.8)	116	100.0 (98.8, 100.0)	429	97.0 (96.2, 97.6)
	November	264	95.8 (94.7, 96.7)	38	89.5 (84.0, 93.3)	124	100.0 (98.9, 100.0)	426	96.5 (95.7, 97.1)
	December	NA[c]		71	97.2 (94.6, 98.6)	121	100.0 (98.9, 100.0)	192	99.0 (98.0, 99.5)
2022	January	NA[c]		49	87.8 (83.0, 91.3)	139	100.0 (99.0, 100.0)	188	96.8 (95.5, 97.8)
	February	NA[c]		74	98.6 (96.4, 99.6)	132	100.0 (99.0, 100.0)	206	99.5 (98.7, 99.9)

CI, confidence interval; NR, not reported; NA, not applicable.
[a]Data suppressed due to small cell size.
[b]No samples tested.
[c]Data collection ended in December 2021 for the Cabarrus County study
