# Supplementary material for: Incidence of severe acute respiratory syndrome coronavirus 2 (SARS-CoV-2) infection in North Carolina from December 2020 – February 2022
Source: PLoS One. 2025 Oct 8;20(10):e0332645. doi: 10.1371/journal.pone.0332645 (PMC12507194; doi:10.1371/journal.pone.0332645)
Supplement: S2 Table — (S2 Table.PDF) [file pone.0332645.s003.pdf]

Month of Study		Cabarrus County		Chatham County		Pitt County		Overall	
		n	% (95% CI)	n	% (95% CI)	n	% (95% CI)	N	% (95% CI)
2020	December	NR[a]		94	3.2 (0.7, 9.3)	152	1.3 (0.2, 4.7)	246	2.4 (0.9, 5.2)
2021	January	273	5.9 (4.8, 7.1)	102	1.0 (0.0, 5.3)	160	3.1 (1.0, 7.1)	535	4.1 (3.5, 4.8)
	February	NR[a]		95	4.2 (1.2, 10.4)	144	1.4 (0.2, 4.9)	239	4.2 (2.0, 7.6)
	March	285	3.2 (2.4, 4.2)	103	2.9 (0.6, 8.3)	132	0.0 (0.0, 2.8)	520	2.3 (1.8, 2.9)
	April	NA[b]		103	1.0 (0.0, 5.3)	133	0.0 (0.0, 2.7)	236	0.4 (0.0, 2.3)
	May	NA[b]		101	1.0 (0.0, 5.4)	NA[b]		101	1.0 (0.0, 5.4)
	June	272	0.7 (0.4, 1.4)	118	0.0 (0.0, 3.1)	NA[b]		390	0.5 (0.2, 1.0)
	July	270	0.0 (0.0, 0.5)	95	0.0 (0.0, 3.8)	NA[b]		365	0.0 (0.0, 0.4)
	August	264	0.8 (0.4, 1.5)	61	3.3 (0.4, 11.3)	NA[b]		325	1.2 (0.8, 1.9)
	September	264	2.3 (1.6, 3.2)	43	0.0 (0.0, 8.2)	NA[b]		307	2.0 (1.4, 2.8)
	October	260	1.2 (0.7, 2.0)	52	1.9 (0.0, 10.3)	105	0.0 (0.0, 3.5)	417	1.0 (0.6, 1.5)
	November	264	0.8 (0.4,1.5)	43	0.0 (0.0, 8.2)	116	0.0 (0.0, 3.1)	423	0.5 (0.2, 1.0)
	December	NA[c]		65	1.5 (0.0, 8.3)	113	0.0 (0.0, 3.2)	178	0.6 (0.0, 3.1)
2022	January	NA[c]		43	0.0 (0.0, 8.2)	132	9.1 (4.8, 15.3)	175	6.9 (3.6, 11.7)
	February	NA[c]		67	9.0 (3.4, 18.5)	115	11.3 (6.2, 18.6)	182	10.4 (6.4, 15.8)

CI, confidence interval; NR, not reported; NA, not applicable.

[a]Data suppressed due to small cell size.

[b]No samples tested for nucleocapsid protein.

[c]Data collection ended in December 2021 for the Cabarrus County study.
