# Supplementary material for: Incidence of severe acute respiratory syndrome coronavirus 2 (SARS-CoV-2) infection in North Carolina from December 2020 – February 2022
Source: PLoS One. 2025 Oct 8;20(10):e0332645. doi: 10.1371/journal.pone.0332645 (PMC12507194; doi:10.1371/journal.pone.0332645)
Supplement: S3 Table — (S3 Table.PDF) [file pone.0332645.s004.pdf]

Month of Study		Cabarrus County		Chatham County		Pitt County		Overall	
		n	% (95% CI)	n	% (95% CI)	n	% (95% CI)	n	% (95% CI)
2020	December	567	2.5 (2.0, 3.1)	165	0.0 (0.0, 2.2)	285	1.4 (0.4, 3.6)	1,017	1.8 (1.1, 2.8)
2021	January	561	2.5 (2.0, 3.1)	180	0.6 (0.0, 3.1)	340	1.8 (0.7, 3.8)	1,081	1.9 (1.1, 2.8)
	February	562	2.0 (1.5, 2.5)	129	0.0 (0.0, 2.8)	287	0.7 (0.1, 2.5)	978	1.3 (0.7, 2.3)
	March	489	0.8 (0.5, 1.3)	233	0.4 (0.0, 2.4)	314	0.3 (0.1, 1.8)	1,036	0.6 (0.2, 1.3)
	April	8	0.0 (0.0, 36.9)	201	0.0 (0.0, 1.8)	282	0.0 (0.0, 1.3)	491	0.0 (0.0, 0.7)
	May	250	0.4 (0.1, 1.1)	217	0.0 (0.0, 1.7)	183	0.5 (0.0, 3.0)	650	0.2 (0.0, 0.9)
	June	506	0.0 (0.0, 0.7)	241	0.0 (0.0, 1.5)	134	0.0 (0.1, 2.7)	881	0.1 (0.0, 0.6)
	July	517	1.0 (0.7, 1.4)	192	0.0 (0.0, 1.9)	115	0.9 (0.0, 4.7)	824	0.7 (0.3, 1.6)
	August	523	2.3 (1.8, 2.9)	79	2.5 (0.3, 8.8)	NA[a]		602	2.3 (1.9, 2.9)
	September	522	0.8 (0.5, 1.2)	55	0.0 (0.0, 6.5)	NA[a]		577	0.7 (0.4, 1.1)
	October	514	1.0 (0.7, 1.4)	64	0.0 (0.0, 5.6)	172	0.6 (0.0, 3.2)	750	0.8 (0.6, 1.1)
	November	NR[b]		50	2.0 (0.1, 10.6)	254	0.0 (0.0, 1.4)	304	0.3 (0.1, 0.9)
	December	NA[c]		77	1.3 (0.0, 7.0)	230	0.9 (0.1, 3.1)	307	1.0 (0.6, 1.7)
2022	January	NA[c]		63	4.8 (1.0, 13.3)	255	8.2 (5.2, 12.3)	318	7.5 (6.5, 8.8)
	February	NA[c]		79	3.8 (0.8, 10.7)	252	4.4 (2.2, 7.7)	331	4.2 (3.4, 5.2)
December 2020 – February 2022		5,020	1.4 (1.3, 1.5)	2,025	0.6 (0.3, 1.0)	3,103	1.6 (1.2, 2.1)	10,148	1.3 (1.2, 1.4)

CI, confidence interval; NA, not applicable; NR, not reported.

[a]Sample collection paused.

[b]Data suppressed due to small cell size.

[c]Data collection ended in December 2021 for the Cabarrus County study.
