# Supplementary material for: Incidence of severe acute respiratory syndrome coronavirus 2 (SARS-CoV-2) infection in North Carolina from December 2020 – February 2022
Source: PLoS One. 2025 Oct 8;20(10):e0332645. doi: 10.1371/journal.pone.0332645 (PMC12507194; doi:10.1371/journal.pone.0332645)
Supplement: S4 Table — (S4 Table.PDF) [file pone.0332645.s005.pdf]

Vaccine Type	Month of Study		Cabarrus County		Chatham County		Pitt County		Overall	
			n	% (95% CI)	n	% (95% CI)	n	% (95% CI)	N	% (95% CI)
Primary series	2020	December	NR[a]		106	0.0 (0.0, 1.3)	168	0.0 (0.0, 0.8)	274	0.0 (0.0, 0.5)
	2021	January	47	6.4 (4.4, 9.3)	118	2.5 (1.7, 3.7)	173	3.5 (2.6, 4.5)	338	3.6 (2.9, 4.3)
		February	288	9.7 (8.6, 11.0)	120	12.5 (10.6, 14.7)	170	26.5 (24.3, 28.8)	578	15.2 (14.2, 16.3)
		March	288	36.1 (34.2, 38.0)	127	45.7 (42.7, 48.7)	166	51.8 (49.2, 54.4)	581	42.7 (41.3, 44.1)
		April	287	59.1 (58.0, 61.9)	130	68.5 (65.7, 71.1)	159	89.3 (87.5, 90.9)	576	70.0 (68.7, 71.2)
		May	285	84.6 (83.1, 86.0)	131	84.7 (82.5, 86.7)	136	94.1 (92.6, 95.3)	552	87.0 (86.0, 87.9)
		June	284	89.4 (88.1, 90.6)	128	86.7 (84.6, 88.6)	134	96.3 (95.0, 97.2)	546	90.5 (89.6, 91.3)
		July	283	90.1 (88.8, 91.2)	128	86.6 (84.4, 88.5)	133	97.7 (96.7, 98.5)	543	91.3 (90.5, 92.1)
		August	282	90.8 (89.6, 91.9)	125	87.2 (85.0, 89.1)	131	98.5 (97.6, 99.1)	538	92.0 (91.2, 92.8)
		September	280	91.8 (90.6, 92.8)	124	89.5 (87.5, 91.2)	131	98.5 (97.6, 99.1)	535	92.9 (92.1, 93.6)
		October	279	93.2 (92.1, 94.1)	123	91.1 (89.2, 92.6)	131	98.5 (97.6, 99.1)	533	94.0 (93.3, 94.7)
		November	279	93.5 (92.5, 94.5)	122	92.6 (90.9, 94.1)	130	98.5 (97.5, 99.0)	531	94.5 (93.8, 95.2)
		December	NA[b]		132	93.2 (91.5, 94.5)	130	99.2 (98.5, 99.6)	262	96.2 (95.3, 96.9)
	2022	January	NA[b]		136	94.1 (92.6, 95.3)	141	97.9 (96.9, 98.6)	277	96.0 (95.2, 96.7)
		February	NA[b]		140	94.3 (92.8, 95.5)	140	97.9 (96.9, 98.5)	280	96.1 (95.2, 96.8)
Booster	2020	December	292	0.0 (0.0, 0.5)	106	0.0 (0.0, 1.3)	168	0.0 (0.0, 0.8)	274	0.0 (0.0, 0.5)
	2021	January	47	0.0 (0.0, 2.9)	118	0.0 (0.0, 1.2)	173	0.0 (0.0, 0.8)	338	0.0 (0.0, 0.4)
		February	288	0.0 (0.0, 0.5)	120	0.0 (0.0, 1.1)	170	0.0 (0.0, 0.8)	578	0.0 (0.0, 0.2)
		March	288	0.0 (0.0, 0.5)	127	0.0 (0.0, 1.1)	166	0.0 (0.0, 0.8)	581	0.0 (0.0, 0.2)
		April	287	0.0 (0.0, 0.5)	130	0.0 (0.0, 1.1)	159	0.0 (0.0, 0.9)	576	0.0 (0.0, 0.2)

		May	285	0.0 (0.0, 0.5)	131	0.0 (0.0, 1.1)	136	0.0 (0.0, 1.0)	552	0.0 (0.0, 0.3)
		June	284	0.0 (0.0, 0.5)	128	0.0 (0.0, 1.1)	134	0.0 (0.0, 1.0)	546	0.0 (0.0, 0.3)
		July	283	0.4 (0.2, 0.7)	128	0.0 (0.0, 1.1)	133	0.0 (0.0, 1.0)	543	0.2 (0.1, 0.4)
		August	282	0.4 (0.2, 0.7)	125	4.0 (3.0, 5.4)	131	0.8 (0.4, 1.5)	538	1.3 (1.0, 1.7)
		September	280	4.3 (3.5, 5.2)	124	12.9 (11.0, 15.1)	131	3.1 (2.2, 4.2)	535	6.0 (5.3, 6.7)
		October	279	28.7 (26.9, 30.5)	123	37.4 (34.5, 40.4)	131	22.9 (20.5, 25.5)	533	29.3 (28.0, 30.6)
		November	279	51.3 (49.2, 53.3)	122	60.7 (57.6, 63.6)	130	23.1 (20.7, 25.7)	531	46.5 (45.1, 48.0)
		December	NA[b]		132	77.3 (74.7, 79.6)	130	23.1 (20.7, 25.7)	262	50.4 (48.3, 52.5)
	2022	January	NA[b]		136	79.4 (77.0, 81.7)	141	24.8 (22.5, 27.4)	277	51.6 (49.6, 53.6)
			NA[b]		140	82.9 (80.6, 84.9)	140	25.0 (22.6, 27.5)	280	53.9 (51.9, 55.9)

CI, confidence interval; NA, not applicable; NR, not reported.

[a]Data suppressed due to small cell size.

[b]Data collection ended in December 2021 for the Cabarrus County study.
