# Supplementary material for: Incidence of severe acute respiratory syndrome coronavirus 2 (SARS-CoV-2) infection in North Carolina from December 2020 – February 2022
Source: PLoS One. 2025 Oct 8;20(10):e0332645. doi: 10.1371/journal.pone.0332645 (PMC12507194; doi:10.1371/journal.pone.0332645)
Supplement: S5 Table — (S5 Table.PDF) [file pone.0332645.s006.pdf]

Month of Study		Cabarrus County		Chatham County		Pitt County	
		NCDHHS % (95% CI)	Study % (95% CI)	NCDHHS % (95% CI)	Study % (95% CI)	NCDHHS % (95% CI)	Study % (95% CI)
2020	December	1.5 (1.5, 1.5)	NR[a]	0.6 (0.6, 0.6)	3.2 (0.7, 9.3)	1.6 (1.6, 1.6)	1.3 (0.2, 4.7)
2021	January	2.2 (2.1, 2.2)	5.9 (4.8, 7.1)	1.2 (1.1, 1.2)	1.0 (0.0, 5.3)	2.3 (2.3, 2.3)	3.1 (1.0, 7.1)
	February	0.7 (0.7, 0.7)	NR[a]	0.4 (0.3, 0.4)	4.2 (1.2, 10.4)	0.9 (0.9, 0.9)	1.4 (0.2, 4.9)
	March	0.4 (0.4, 0.4)	3.2 (2.4, 4.2)	0.2 (0.2, 0.2)	2.9 (0.6, 8.3)	0.3 (0.3, 0.3)	0.0 (0.0, 2.8)
	April	0.6 (0.6, 0.6)	NA[b]	0.2 (0.2, 0.2)	1.0 (0.0, 5.3)	0.5 (0.5, 0.5)	0.0 (0.0, 2.7)
	May	0.3 (0.3, 0.3)	NA[b]	0.1 (0.1, 0.1)	1.0 (0.0, 5.4)	0.5 (0.5, 0.5)	NA[b]
	June	0.1 (0.1, 0.1)	0.7 (0.4, 1.4)	0.0 (0.1, 0.1)	0.0 (0.0, 3.1)	0.1 (0.1, 0.1)	NA[b]
	July	0.5 (0.5, 0.5)	0.0 (0.0, 0.5)	0.4 (0.3, 0.4)	0.0 (0.0, 3.8)	0.5 (0.5, 0.5)	NA[b]
	August	1.3 (1.3, 1.3)	0.8 (0.4, 1.5)	0.8 (0.7, 0.8)	3.3 (0.4, 11.3)	1.4 (1.3, 1.4)	NA[b]
	September	1.1 (1.1, 1.1)	2.3 (1.6, 3.2)	0.6 (0.6, 0.6)	0.0 (0.0, 8.2)	1.6 (1.5, 1.6)	NA[b]
	October	0.6 (0.6, 0.6)	1.2 (0.7, 2.0)	0.4 (0.3, 0.4)	1.9 (0.0, 10.3)	0.7 (0.7, 0.7)	0.0 (0.0, 3.5)
	November	0.4 (0.4, 0.4)	0.8 (0.4, 1.5)	0.3 (0.2, 0.3)	0.0 (0.0, 8.2)	0.3 (0.3, 0.3)	0.0 (0.0, 3.1)
	December	0.9 (0.9, 0.9)	NA[c]	0.6 (0.6, 0.6)	1.5 (0.0, 8.3)	0.8 (0.8, 0.8)	0.0 (0.0, 3.2)
2022	January	5.3 (5.3, 5.3)	NA[c]	4.3 (4.2, 4.3)	0.0 (0.0, 8.2)	8.3 (8.2, 8.3)	9.1 (4.8, 15.3)
	February	0.9 (0.9, 0.9)	NA[c]	0.8 (0.8, 0.8)	9.0 (3.4, 18.5)	1.1 (1.1, 1.1)	1.3 (6.2, 18.6)

CI, confidence interval; NA, not applicable; NR, not reported.

[a]Data suppressed due to small cell size.

[b]No samples tested for nucleocapsid protein.

[c]Data collection ended in December 2021 for the Cabarrus County study.
